# Whole genome expression profiling reveals a significant role for immune function in human abdominal aortic aneurysms

**DOI:** 10.1186/1471-2164-8-237

**Published:** 2007-07-16

**Authors:** Guy M Lenk, Gerard Tromp, Shantel Weinsheimer, Zoran Gatalica, Ramon Berguer, Helena Kuivaniemi

**Affiliations:** 1 Center for Molecular Medicine and Genetics, Wayne State University School of Medicine, Detroit, MI, USA; 2 Department of Neurology, Wayne State University School of Medicine, Detroit, MI, USA; 3 Department of Surgery, Wayne State University School of Medicine, Detroit, MI, USA; 4 Department of Pathology, Creighton University School of Medicine, Omaha, NE, USA; 5 Department of Surgery, University of Michigan, Ann Arbor, MI, USA

## Abstract

**Background:**

Abdominal aortic aneurysms are a common disorder with an incompletely understood etiology. We used Illumina and Affymetrix microarray platforms to generate global gene expression profiles for both aneurysmal (AAA) and non-aneurysmal abdominal aorta, and identified genes that were significantly differentially expressed between cases and controls.

**Results:**

Affymetrix and Illumina arrays included 18,057 genes in common; 11,542 (64%) of these genes were considered to be expressed in either aneurysmal or normal abdominal aorta. There were 3,274 differentially expressed genes with a false discovery rate (FDR) ≤ 0.05. Many of these genes were not previously known to be involved in AAA, including *SOST *and *RUNX3*, which were confirmed using Q-RT-PCR (Pearson correlation coefficient for microarray and Q-RT-PCR data = 0.89; p-values for differences in expression between AAA and controls for *SOST*: 4.87 × 10^-4 ^and for *RUNX3*: 4.33 × 10^-5^). Analysis of biological pathways, including Gene Ontology (GO) and Kyoto Encyclopedia of Genes and Genomes (KEGG), indicated extreme overrepresentation of immune related categories. The enriched categories included the GO category Immune Response (GO:0006955; FDR = 2.1 × 10^-14^), and the KEGG pathways *natural killer cell mediated cytotoxicity *(hsa04650; FDR = 5.9 × 10^-6^) and *leukocyte transendothelial migration *(hsa04670; FDR = 1.1 × 10^-5^).

**Conclusion:**

Previous studies have provided evidence for the involvement of the immune system in AAA. The current expression analysis extends these findings by demonstrating broad coordinate gene expression in immunological pathways. A large number of genes involved in immune function were differentially expressed in AAA, and the pathway analysis gave these results a biological context. The data provide valuable insight for future studies to dissect the pathogenesis of human AAA. These pathways might also be used as targets for the development of therapeutic agents for AAA.

## Background

Abdominal aortic aneurysm (AAA) is a common, late age-at-onset disorder affecting approximately 1–6 % of the population of industrialized countries, and approximately 9.5% of those 65 years and older [1,2]. Rupture of AAA has high mortality, and is the 13^th ^leading cause of death in the United States [1].

The etiology of AAA is complex, with many environmental and genetic factors contributing to the risk [1,3,4]. AAAs are characterized by signs of local chronic inflammation of the aortic wall, decreased numbers of smooth muscle cells in the aortic media layer and fragmentation of the extracellular matrix at the site of the aneurysm [4]. Risk factors have been identified, but the molecular events responsible for the initiation and progression of AAAs remain unknown. Many studies have focused on limited sets of plausible candidate genes, such as those encoding matrix metalloproteinases (MMPs) and their inhibitors, but recently microarrays have been used to elucidate a more global gene expression profile for AAA [4-8].

Previous studies have provided evidence for the involvement of the immune system in AAA formation and progression [see [9-12]]. Animal models of AAA have been used to test the contributions of components of the immune system [13,14]. Cellular involvement of neutrophils, T cells, and macrophages has been found to be important in the formation of AAA in animal models [15-17]. In addition, various immune-related molecules have been examined using the AAA models, and molecules such as NFκB, c-Jun N-terminal kinase, P47phox, IFNγ, and IL4, among others, have also been shown to be involved in the formation of an experimental aneurysm [18-21]. In humans, the cellular immune infiltrates in the AAA tissue have been characterized by several groups. The findings indicate that many types of immune cells are found in AAAs, such as macrophages, neutrophils, natural killer (NK) cells, T cells, and B cells [22-25]. Changes in the circulating immune components of AAA patients included an increase in NK cells and CD4^+^CD28^- ^T cells, as well as a decrease in the number of CD4^+^CD31^+ ^T cells [26-28]. These findings implicate the immune system in pathogenesis of AAA, and it has even been asserted that AAA is autoimmune [29-31]. The precise role of the immune system in AAA pathophysiology has yet to be elucidated.

The present study utilized two microarray platforms to generate global mRNA expression profiles for both aneurysmal and non-aneurysmal abdominal aorta. These results provide valuable information to identify functional pathways involved in the pathogenesis of AAA without biasing the data by an *a priori *selection of genes. The global approach allows identification of the most relevant pathways, and the cross-platform validation protects against platform-specific artifacts. The current study does indeed validate some genes that were previously studied; however, it also extends the findings from the previous studies, suggests involvement of genes not previously studied, and in its global aggregate suggests which components within the pathways are the active components.

## Results

Aortic wall tissue samples were collected from patients who underwent repair operations for AAA, and from age, sex, and ethnicity-matched controls at autopsies (Table 1). RNA isolated from the tissue samples was used in global gene expression studies with two different platforms (Affymetrix and Illumina).

**Table 1:** Sample demographics

**Sample ID**	Type*	**Sex**	**Age**	**Cause of Death**	**RIN†**	**Type of Experiment‡**
A1-F	AAA	F	82	NA	7.2	MA, QRT
A2-F	AAA	F	68	NA	7.0	MA, QRT
A3-F	AAA	F	64	NA	7.9	MA, QRT
A1-M	AAA	M	64	NA	7.1	MA, QRT
A2-M	AAA	M	63	NA	6.5	MA, QRT
A3-M	AAA	M	67	NA	6.9	MA, QRT
A4-M	AAA	M	63	NA	5.9	MA, QRT
C1-F	Control	F	74	Lung cancer	6.6	MA, QRT
C2-F	Control	F	52	Metastatic breast carcinoma	7.7	MA, QRT
C3-F	Control	F	84	Aortic arch dissection	6.2	MA, QRT
C1-M	Control	M	65	Peritonitis	8.0	MA, QRT
C2-M	Control	M	59	Hepatocellular carcinoma	7.4	MA, QRT
C3-M	Control	M	52	Alcoholic liver cirrhosis	8.5	MA, QRT
C4-M	Control	M	73	Lung cancer	6.1	MA, QRT
A4-F	AAA	F	64	NA	5.5	QRT
A5-M	AAA	M	70	NA	7.8	QRT
A6-M	AAA	M	63	NA	6.3	QRT
C5-M	Control	M	41	Congestive heart failure	6.6	QRT
C6-M	Control	M	45	Ischemic heart disease	6.7	QRT
C7-M	Control	M	75	Cholangiocarcinoma	4.5	QRT

* AAA samples were obtained during aneurysmal repair operations from live patients. Control samples were obtained from autopsies. All donors were Caucasian.

† Agilent BioAnalyzer 2100 RNA integrity number.

‡ MA, microarray; QRT, quantitative real-time RT-PCR. A3-F was analyzed on Affymetrix platform only.

The Affymetrix whole-genome expression chip was run with 4 RNA pools, while the Illumina platform was run on 13 individual RNA samples and 2 pooled RNA samples (Table 1). The two independent platforms are comparable, with a total of 18,057 distinct genes (referred to as RefList) represented on both platforms (Affymetrix: 19,835; Illumina: 20,054). This subset of genes common to the two platforms was used as the basis for the analyses so that concordance between the two platforms could be determined. The genes that were excluded from the RefList were sparsely annotated (36% GO annotation; 3% KEGG annotation), consequently the loss of information due to excluding these genes was negligible.

A total of 11,542 distinct genes (64% of the RefList) passed the expression criteria of a *Detection Score *≥ 0.99 in either AAA or control groups based on Illumina arrays (AAA: 11,077; control: 10,478; both: 10,285). The Affymetrix data indicated that 11,901 (66% of the RefList) of the genes were expressed, with 9,955 genes considered expressed by both platforms (55% of the RefList). Consistent with previous findings, increasing detection stringency increased the proportion of genes in common between the platforms [32]. To be conservative in avoiding false negatives (i.e., to avoid eliminating genes unnecessarily) we used a *Detection Score *≥ 0.99 from the Illumina data as our criteria for a gene to be considered expressed.

**Figure 1:**
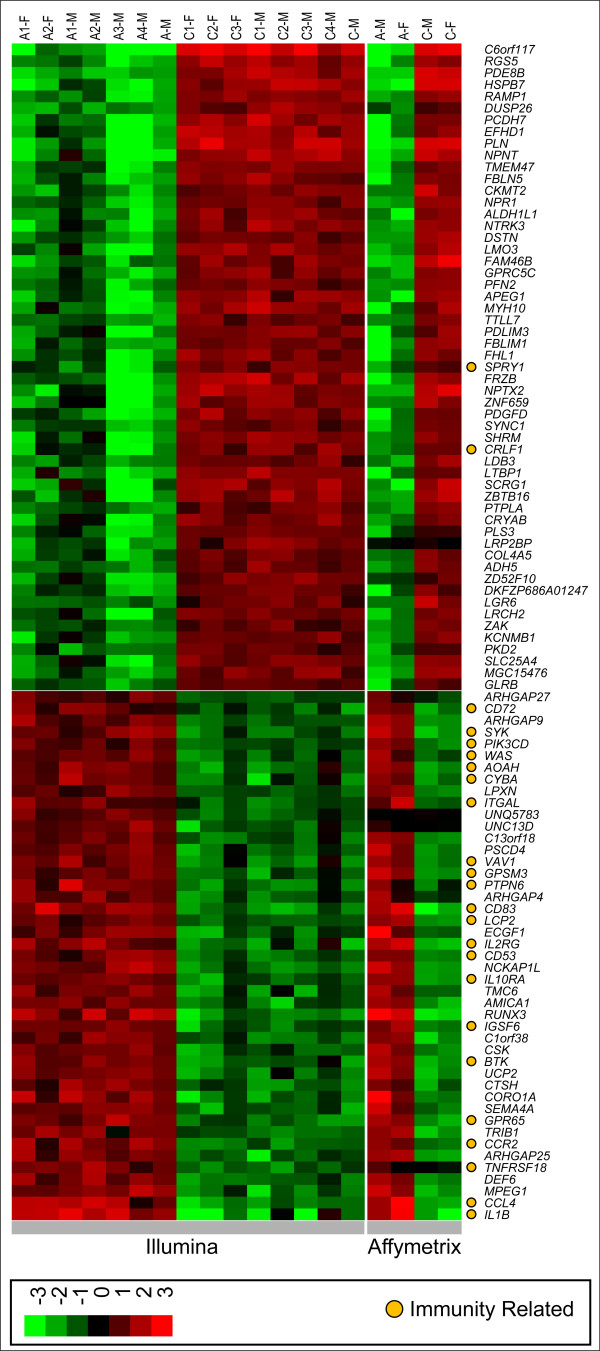
Heatmap of the 100 most differentially expressed genes in AAA. To show consistency of the results between the two platforms, both Illumina (15 left columns) and Affymetrix (4 right columns) results are presented. Sample IDs are on top (see Table 1 for details) and gene symbols on the right. A listing of these 100 genes with expression values in both conditions as well as the FDR values is provided [see the additional file 2: Table II].

Comparison of the profiles between AAA and control revealed 4,627 genes on the Illumina platform with a *Detection Score *≥ 0.99 and a |DiffScore| ≥ 13 (which equates to a nominal p-value of ≤ 0.05). Correction for multiple testing yielded 3,274 distinct differentially expressed genes (DiffList) with an FDR ≤ 0.05 [see additional file 1:Table I] [33]. The extreme differences observed between AAA and control abdominal aorta are shown in Figure 1 and additional data [see additional file 2:Table II] for illustrative purposes, and to demonstrate the concordance of the results between Illumina and Affymetrix. (The top 100 genes were chosen to provide a readable list in the figure.) Increasing stringency in the selection of the differentially expressed genes resulted in greater concordance between the two platforms [90% (1,076/1,191) at FDR ≤ 0.001; 96% (465/485) at FDR ≤ 1.0 × 10^-5^]. There were more genes with decreased expression in AAA (1,793/3,274; 55%) than genes with increased expression (1,481/3,274; 45%). Therefore, we selected genes in proportion to increased (45%) and decreased (55%) expression in AAA for visualization (Figure 1).

Isoform analysis on this dataset is limited due to the divergent nature of the arrays, i.e., the Affymetrix microarray used has probesets consisting of multiple oligos from a transcript making it difficult to calculate individual isoform contributions to the overall signal, while the Illumina platform has exact replicates with isoform information. Using the Illumina platform, 138 of the 3,274 (4%) genes on the DiffList had the largest differential signal from a particular isoform (as opposed to a general probe). An example of one such difference is in *CD86 *(*CD86 antigen*; Entrez Gene ID: 942), where the full length isoform is detected and increased significantly in AAA, while the shorter transcript variant is not detected in either condition, which indicates that *CD86 *is being transcribed by activated as opposed to nonactive monocytes [34].

**Figure 2:**
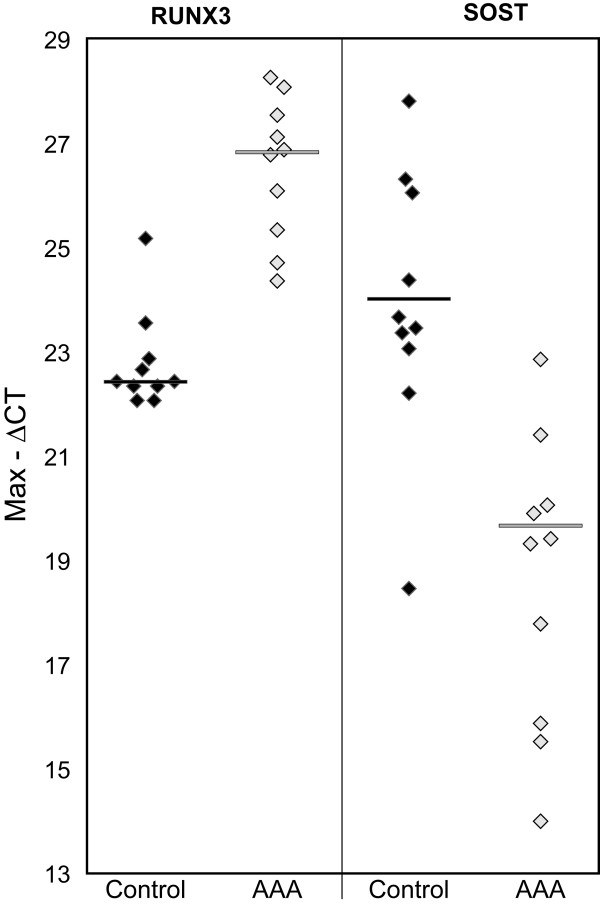
Q-RT-PCR results for *RUNX3 *and *SOST *in AAAs (n = 10) and control abdominal aortas (n = 10). Larger values of Max – ΔCT represent larger amounts of RNA present, and a change of this value by 1 represents a two-fold increase in the amount of mRNA. Medians are indicated by horizontal lines. See Table 1 for details on samples analyzed.

We carried out real-time quantitative reverse-transcriptase PCR (Q-RT-PCR) validation for two genes, *SOST *(sclerosteosis) and *RUNX3 *(runt-related transcription factor 3) which each were significantly altered according to the microarray data (Figure 2). The Pearson correlation of the expression values between the Illumina microarray and Q-RT-PCR data was 0.89 for the exact replicates (AAA n = 6; control n = 7). Consistent with prior observations, there was a bias to underestimate the fold-change of highly differential genes in the microarray data (Illumina vs. Q-RT-PCR; *SOST*: 28 vs. 38; *RUNX3*: 10 vs. 13) [35].

Inspection of the genes in Figure 1 revealed numerous genes involved in immune function with 24% having some connection to immunity (2/55 decreased; 22/45 increased). We subsequently analyzed the 3,274 genes in the DiffList for enrichment in the Kyoto Encyclopedia of Genes and Genomes (KEGG) pathways and Gene Ontology (GO) categories [36-38]. The analysis of KEGG pathways revealed 21 enriched non-metabolic pathways (Table 2). Enriched GO categories corresponded well with enriched KEGG pathways, with immune-related functions highly represented in both [e.g., the GO category "immune response" (GO:0006955) having an FDR= 2.08 x 10^-14^; not shown]. Since KEGG pathways provide more biological information in that they show molecular interactions, further analyses focused on KEGG pathways.

**Table 2:** KEGG biological pathways for differentially expressed genes

**KEGG pathway**	Probed*	**Observed**	**Expected**	**FDR**
Regulation of actin cytoskeleton (RAC)	204	76	36.99	4.71e-09
Cell adhesion molecules (CAMs)	127	51	23.03	1.63e-07
Focal adhesion (FA)	208	72	37.71	1.98e-07
Natural killer cell mediated cytotoxicity (NK)	127	47	23.03	5.94e-06
Leukocyte transendothelial migration (LTEM)	115	43	20.85	1.06e-05
Type I diabetes mellitus (T1DM)	42	20	7.62	1.29e-04
ECM-receptor interaction (ECM)	86	31	15.59	5.69e-04
T cell receptor signaling pathway (T cell)	99	34	17.95	6.93e-04
Hematopoietic cell lineage (HCL)	84	30	15.23	7.03e-04
B cell receptor signaling pathway (B cell)	70	26	12.69	8.65e-04
Adherens junction (AJ)	76	27	13.78	1.38e-03
Gap junction (GJ)	96	32	17.41	1.38e-03
Calcium signaling pathway (Ca2+)	175	50	31.73	2.12e-03
Antigen processing and presentation (AP&P)	79	27	14.32	2.12e-03
Cytokine-cytokine receptor interaction (CCRI)	247	66	44.78	2.12e-03
Tight junction (TJ)	116	36	21.03	2.12e-03
Alzheimer's disease (AD)	22	11	3.99	2.60e-03
MAPK signaling pathway (MAPK)	270	69	48.95	5.10e-03
Long-term potentiation (LTP)	68	21	12.33	2.58e-02
Wnt signaling pathway (WNT)	144	38	26.11	2.87e-02
Axon guidance (AG)	129	34	23.39	4.02e-02

* Probed indicates the number of genes with a membership in a particular pathway and for which probes were available on both microarray platforms.

**Figure 3:**
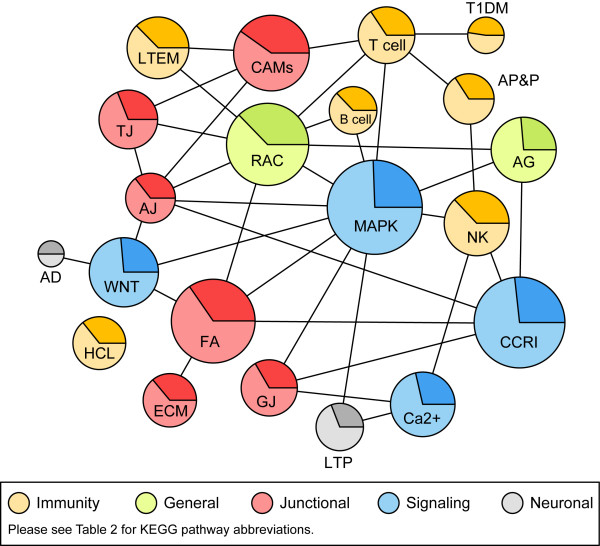
Interactions of KEGG biological pathways for genes differentially expressed in AAA. The 21 most significant pathways are shown (see Table 2 for details). Areas represent pathway size (number of genes) and darker shades indicate the proportion of differentially expressed genes of the total number of genes in the pathway. See Methods section for details on how the interactions were identified.

The enriched KEGG pathways are not independent of one another; many KEGG pathways refer to other KEGG pathways, i.e., the pathways interact. This interaction is illustrated in Figure 3. The relative size of each pathway in terms of the number of genes represented is indicated by the area of the circle, and the proportion of differentially expressed genes is indicated by the darker shaded segments. The pathway names in Figure 3 have been shortened and the abbreviations can be found in Table 2.

The top five most enriched KEGG pathways are *regulation of actin cytoskeleton *(RAC; hsa04810; FDR= 4.71e-09), *cell adhesion molecules *(CAM; hsa04514; FDR= 1.63e-07), *focal adhesion *(FA; hsa04510; FDR= 1.98e-07), *natural killer cell mediated cytotoxicity *(NK; hsa04650; FDR= 5.94e-06), and *leukocyte transendothelial migration *(LTEM; hsa04670; FDR= 1.06e-05), see Table 2.

The KEGG pathways RAC, CAM, and FA each participate in many aspects of biology and, therefore, each interact with several other KEGG pathways (see Figure 3). In the RAC pathway, 147/204 probed genes were expressed in either AAA or controls (AAA: 132; control: 134), and 76 of the expressed genes (51%) were differentially expressed between cases and controls. In the CAM pathway, 93/127 probed genes were expressed in either AAA or controls (AAA: 89; control: 73), and 51 of the expressed genes (55%) were differentially expressed between cases and controls. In the FA pathway, 158/208 probed genes were expressed in either AAA or controls (AAA: 151; control: 148), and 72 of the expressed genes (46%) were differentially expressed between cases and controls. There is considerable overlap between these three pathways and the NK and LTEM pathways in that 39 of the 127 (31%) genes in the CAM pathway are also in the NK and LTEM pathways. The RAC pathway shares 52 of its 204 (25%) genes with the NK and LTEM pathways, and the FA pathway shares 61 of its 208 (29%) genes with these two pathways.

**Figure 4:**
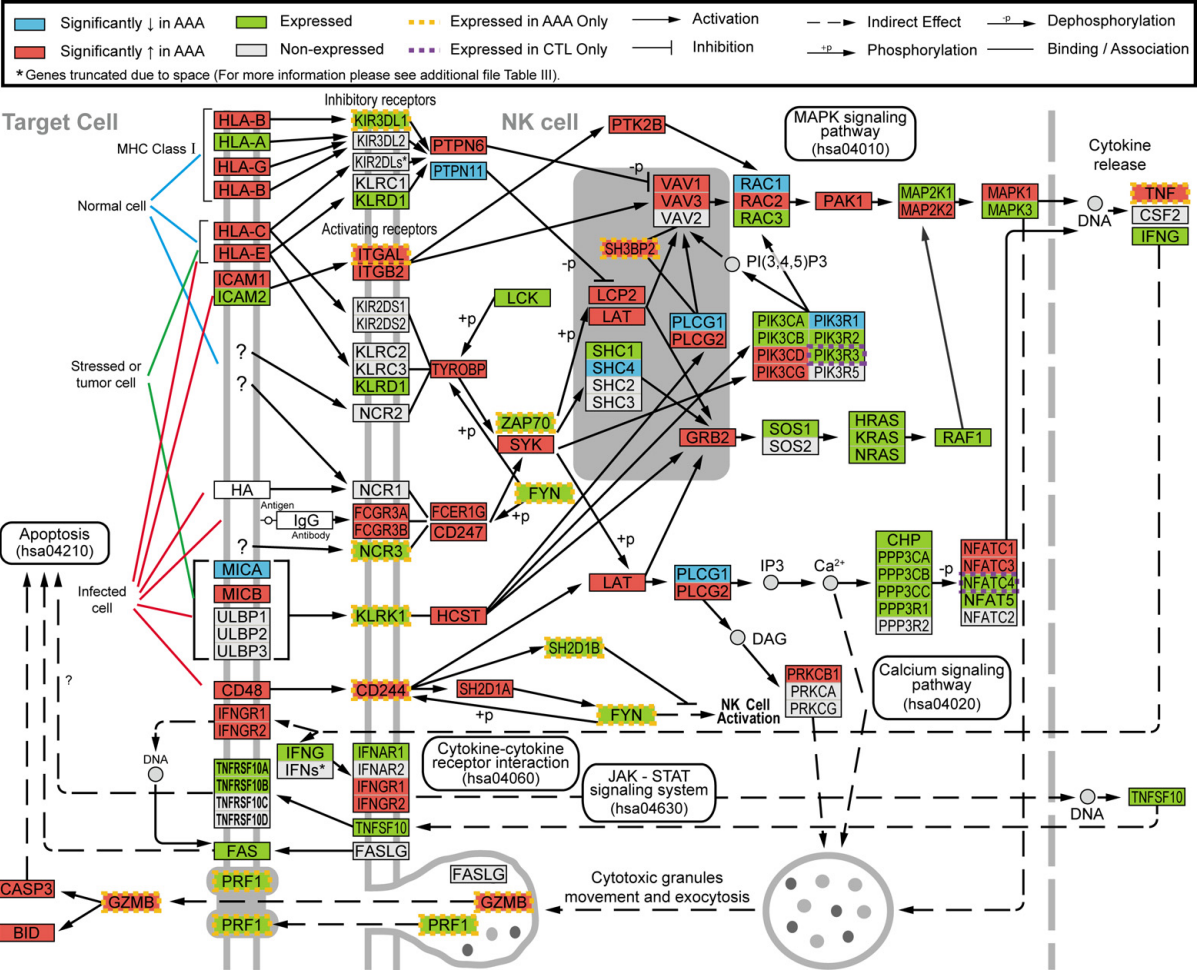
Modified "natural killer cell mediated cytotoxicity" (hsa04650) pathway from KEGG. Protein symbols were replaced by gene symbols to reflect gene-centric data. See key for explanation of colors and symbols. Gene symbols were not italicized for better legibility [see additional file 4: Table III for expression values of the genes in both conditions as well as the FDR value for those that were significantly different].

NK and LTEM pathways (Table 2) are more detailed in their annotations in the KEGG database, and of the top five enriched pathways also have the most direct biological relevance to AAA. In the NK pathway (Figure 4), 86/127 probed genes were expressed in either AAA or controls (AAA: 84; control: 75). Altogether, 47/86 (55%) of the expressed genes were differentially expressed between cases and controls. In the LTEM pathway [see additional file 3: Figure I], 89/115 probed genes were expressed in either condition (AAA: 84; control: 79), and 43/89 (48%) were differentially expressed. Taken together, 75 distinct genes from these two pathways were differentially expressed between AAA and control, since 10 genes had membership in both KEGG pathways. It is also noteworthy that 7/75 (9.3%; *CYBA*, *ITGAL*, *LCP2*, *PIK3CD*, *PTPN6*, *SYK*, *VAV1*) were among the 100 most differentially expressed genes shown in Figure 1 and additional data [see additional file 2: Table II]. The expression values for all the probed genes, and FDR values for those significantly different in these pathways, are provided as additional data [see additional file 4: Table III and additional file 5: Table IV].

To illustrate the gene-based data, KEGG pathways were redrawn with genes instead of proteins as seen in Figure 4 and additional data [see additional file 3: Figure I]. This increased the complexity of the pathways, since multiple genes occasionally coded for a particular protein type (e.g. the NFAT proteins), or protein complex. In some instances where the original KEGG protein symbol referred to two or more genes, the genes had differential expression in opposite directions, i.e., some genes had reduced, and other genes increased expression. An example of this is Rac in the NK pathway where *RAC1 *is decreased in AAA (FDR= 5.28 × 10^-4^) and *RAC2 *is increased (FDR= 1.16 × 10^-5^). In the original KEGG pathway both of the RAC genes that link to the protein in the KEGG database are represented by the symbol "Rac", and therefore some of the information of the gene enrichment was unavailable even with biological annotation, prior to our modifications of the KEGG pathways.

## Discussion

Multiple etiological factors contribute to AAA, and its pathobiology is incompletely understood. Experiments with animal models such as the angiotensin II infusion, elastase perfusion, and calcium chloride exposure models [13], in combination with more detailed analysis of the condition in humans, are beginning to elucidate the pathobiology. The current study provides a comprehensive, global view of the gene expression in AAA and control aorta, and the pathway analyses presented extend previous findings and provide novel insights into the pathogenesis of AAA.

Genes such as *MMP1*,*MMP9*,*IL1B*,*PLAU*, *PLAUR*, and several cathepsin family members previously implicated in the pathogenesis of AAA were among the 3,274 differentially expressed genes. We selected 2 genes, *RUNX3 *and *SOST*, novel to AAA for further study since their expression was substantially different in opposing directions between AAA and control. *SOST *belongs to the DAN family of antagonists of the transforming growth factor-beta (TGF-β) superfamily and also antagonizes Wnt signaling [39,40]. Its association with TGF-β is interesting since TGF-β is involved in the Marfan syndrome which has aortic dissection as a characteristic feature [41]. Wnt signaling is involved in many aspects of the vasculature including arterial response to angiotensin II, used to induce AAA in animal models [13]. Together, these lines of evidence suggest that *SOST *is important in AAA pathogenesis. *RUNX3 *also has a role in TGF-β signaling and apoptosis [42], and it is involved in the differentiation of T cells from CD4+ to CD8+ lineages [43].

The enrichment seen in biological pathways provides greater understanding of the processes that may be involved in AAA. This comprehensive approach of gene expression analysis, utilizing annotated pathways rather than isolated genes, as well as the interconnectedness of the pathways (Figure 3), gives a more global picture of the pathology involved in AAA. In addition, subtle changes can be understood, rather than overlooked, when examined in the context of biological processes. Not all pathways are equally informative; it should be noted that the enrichment of some pathways, e.g., RAC, CAM and FA, while highly interesting, lack the specificity of the smaller KEGG pathways, and consequently relevance to AAA is not as obvious. Since the number of overlapping genes in these three pathways and the NK and LTEM pathways was considerable, it is reasonable to conclude that the contribution of the immunological processes in AAA may also contribute to the observed enrichment of these pathways.

The NK pathway (Figure 4) is interesting because immune involvement has been implicated in AAA [25,28,44-47]. The biological processes involved in mediating NK cytotoxicity are not restricted to NK cells alone, but are common to several types of cytotoxic immune cells, including cytotoxic T lymphocytes, NK T cells, and γδ T cells [48]. Although NK cells have been found in AAA tissue, they represent a small fraction of the immune cells present [25]. The gene expression levels in this pathway are likely to be the result of more than one cytotoxic cell type, and further work is necessary to determine the exact composition of the cellular infiltrate and their individual contributions to the increased levels of the genes in this pathway.

Several genes shown in the NK (Figure 4) and LTEM pathways [see additional file 3: Figure I] with dashed orange and purple outlines were identified as expressed exclusively in either AAA or control aorta, but had an FDR > 0.05, i.e., the genes did not pass the FDR ≤ 0.05 threshold for differential expression, but were interesting because their expression was not detectable in one of the conditions. Since the genes failed the FDR ≤ 0.05 threshold they were not included in the DiffList and, therefore, were not included in the pathway enrichment analyses. They are nevertheless noteworthy because of the specific expression in one of the two conditions.

There is increased expression of genes in the activation signaling that begins with *KLRK1 *(NKG2D) and *HCST *(DAP10). Although this signaling is activating, it does not utilize an immunoreceptor tyrosine-based activation motif (ITAM). Interestingly, the activation is specific for certain downstream intermediates while ITAM signaling is relatively insensitive to the isoform of the intermediates [49]. In particular, *HCST *signals through *PLCG2 *in preference to *PLCG1*, and through *VAV1 *in preference to either of the other two *VAV *genes [50,51]. This phenomenon fits well with the observed expression patterns, since *PLCG2 *is significantly increased in AAA while *PLCG1 *is significantly decreased, and *VAV1 *is expressed (unlike *VAV2*) and is among the 100 most differential genes (see Figure 1).

Activation of the NK pathway is suggested by the expression of several of the end-products exclusively in AAA (*PRF1*, *GZMB*), as well as the significant increase of other end-products from this pathway (*CASP3*, *BID*). FAS ligand (*FASLG*) expression, another possible end-product of this pathway, was not detected. Since *FASLG *is not generally stored in the immune cells that utilize it [48], lack of expression indicates that the Fas/FasL system is unlikely to be involved in the pathology of AAA. Taken together, these findings fit well with the granule exocytosis pathway model of cytotoxicity, and support previous findings in AAA.

The process of immune cell infiltration represented by the LTEM pathway [see additional file 3: Figure I] is also biologically relevant to AAA. The presence of immune cells, as well as the cell types comprising the infiltrate, has previously been shown in AAA tissue [25,28,46]. We observed significantly increased expression of several molecules attributed to leukocytes (*ITGB2*, *ITGAM*, *CXCR4*) and in some cases a specificity to AAA (*ITGAL*, *ITGA4*, *ITK*, *TXK*; Figure 4). Also, the significant decrease of *ITGB1 *was seen previously [7], lending further credence to the changes demonstrated. These data not only confirm that different leukocytes are present in AAA tissue, but may help to identify the specific molecules involved in their infiltration of the aorta for future studies.

The data presented here confirm many of the findings of previous gene expression studies on AAA using smaller sets of genes. One study using spotted membrane macroarrays found that 104 of the 1,185 genes tested were differentially expressed in AAA [6]. Our data confirmed 41 (39%) of the 104 genes in that the same genes had significantly different expression and the expression was increased or decreased in the same condition, i.e., the differential expression was in the same direction [6]. Another macroarray study used the same array and found differences in 20 genes, of which our data confirmed 13 (65%) [7]. There were 13 differentially expressed genes in common between the two macroarray studies, and 10 of these (77%) were increased or decreased similarly and had an FDR ≤ 0.05 in our study. Our microarray study differs from the prior macroarray studies in array type and analytical techniques. Nevertheless, the high level of confirmation of previous observations provides validation for the current study.

Previous histological studies of AAA confirm many of the differences observed in our microarray data. For instance, the specific expression of perforin in AAA was previously seen using immunohistochemistry [52]. Also, the differential expression of *IL8*, confirmed in prior expression studies [6,7], was previously validated by immunohistochemistry [45]. *IL1B*, among the most differentially expressed genes, was also found to be highly differential in comparisons of protein levels [47]. The consistency with protein data is useful since it shows that the expression differences are likely functional.

The present study has several limitations. One specific sample-related limitation is that the aneurysm tissue used came from very late-stage disease, when the aneurysm was large enough for surgery (≥ 5.5 cm). Since several studies have shown that the risks of surgical resection are not outweighed by the risk of rupture until an aneurysm is quite large (~5.5 cm), the only tissue available is from late-stage AAA. A time course of gene expression during aneurysm development could yield valuable results, but this is not possible with humans.

Bias may have been introduced in the study since our control tissue was derived from autopsies. Extensive quality control analyses revealed no detectable differences between AAA and control RNA [see additional file 6: Figure II and file 7: Figure III]. Post mortem changes in RNA have been studied and shown to have an effect on RNA integrity, but this is both tissue and time dependent and can be limited [53]. Despite these limitations, post mortem tissues have been used successfully for expression studies [53,54].

Another sample-related limitation is that the mRNA was obtained from the entire control abdominal aorta and from the resected AAA; therefore, expression at specific sites or layers in the aorta or aneurysm cannot be identified. It is, however, impractical to obtain highly comparable, anatomically similar aneurysm and aorta tissue.

The incompleteness of gene annotation is yet another limitation of our study, since only 859 (26%) of the 3,274 differentially expressed genes had KEGG annotations and 2,615 (80%) of these genes had GO annotations. We, therefore, examined enrichment in both KEGG and GO annotations to assure consistency. Restricting the analysis to genes with GO and KEGG annotations limits our analyses, but provides greater functional interpretation. Isoforms also complicate the analysis and interpretation of microarray data. The Illumina Sentrix-6 array contains probes for various specific isoforms of only 1,935 genes. The biological information that these annotations can provide is valuable (such as the example of CD86 isoforms in the results), but limited not only by our knowledge (and the testability) of the transcript isoforms, but also by the biological understanding of what these differences indicate. Future reanalysis of the data, particularly of the genes that currently lack annotation and analysis of any new functional information concerning probed isoforms, will be useful.

## Conclusion

In summary, the expression profiles presented here extend the evidence for involvement of immunity in non-inflammatory AAA. The substantially increased evidence for the role of cell-mediated cytotoxicity in AAA and the molecular signaling behind the infiltration of leukocytes, as well as the comprehensive annotation of the genes that are involved in these two pathways, add significantly to the understanding of AAA. Future work to ascertain the contributions of these expression changes to AAA should be undertaken. The results provided by this study offer new insights into AAA pathobiology which will help to direct future research as well as aid in the development of therapeutic options for the treatment of AAA.

## Methods

### Tissue samples, RNA isolation and microarray analysis

Full thickness aortic wall tissue specimens were collected in RNAlater (Ambion) from patients undergoing AAA repair operations at the Harper University Hospital, Detroit, Michigan. Control aortas were all collected within 24 h of death and snap-frozen in liquid nitrogen. Control samples were matched to cases using sex, age, and ethnicity (Table 1). The study was approved by the Institutional Review Board of Wayne State University, Detroit, Michigan, and the research carried out was in compliance with the Helsinki Declaration.

We isolated total RNA from blood vessels as previously described [5]. Quality of RNA was checked by Agilent Bioanalyzer and RNA Integrity Scores are shown in Table 1[55]. Sentrix Human-6 Whole Genome Expression BeadChips (Sentrix Human WG-6; Illumina) were used to identify genes expressed in AAA tissue (n= 6) and from individuals without AAA (controls; n= 7). We analyzed samples both individually as well as in pools. Further QC procedures included examination of raw and adjusted intensity histograms [see additional file 6: Figure II] and principal component analysis [see additional file 7: Figure III] to detect systematic bias. We used the "Detection Score" to determine expression using the Illumina platform. It is a statistical measure in the BeadStudio software (version 1.5.0.34), which is computed based on the Z-value of a gene relative to the Z-value of the negative controls. The Illumina data were adjusted ("normalized") using a cubic spline function. We identified genes differentially expressed in AAA [see additional file 1: Table I] using the Illumina custom error model implemented in BeadStudio. The expression difference score, DiffScore, takes into account background noise and sample variability [56]. The formula for the calculation of the DiffScore is:

*DiffScore *= 10 sgn(μ_*cond *_- μ_*ref*_)log_10 _(*p*)

For the Affymetrix microarray analysis, 10 μg of each RNA pool was processed and analyzed [57]. For the Affymetrix arrays, raw data were processed with the statistical language R using some of the packages provided by the Bioconductor project [58]. We used the GC-RMA algorithm for the adjustment of the data [59]. GC-RMA takes into account the GC content of the probe sequences when comparing the expression intensities of the different probesets.

To combine the Affymetrix and Illumina data, we identified a common reference gene set (RefList) of 18,057 distinct genes represented on both platforms (Affymetrix: n= 19,835 and Illumina: n= 20,054). Heatmaps showing differential expression, with green representing lower, and red higher, were generated using the programs Cluster and TreeView (version 1.0.12) [60].

The microarray data have been deposited to the Gene Expression Omnibus (GEO) database (Series# GSE7084) [61].

### Real-time quantitative-reverse-transcription PCR

Microarray-based mRNA expression was validated with RNA isolated from 10 AAAs and 10 controls (Table 1) by Q-RT-PCR for *RUNX3 *and *SOST *using validated TaqMan^® ^assays available from Applied Biosystems. The Q-RT-PCR assays were performed using 50 ng of RNA as previously described [5].

Functional classification of genes

The differentially expressed gene set [see additional file 1: Table I] was used to identify enriched GO biological process categories using the GoHyperG function (GOStats, Bioconductor) and enrichment in KEGG biological pathways using WebGestalt (Web-based Gene SeT AnaLysis Toolkit) [36-38,62]. The reference gene set comprised the 18,057 distinct genes represented on both Affymetrix and Illumina arrays. The hypergeometric test was used for significance.

Inclusion in KEGG is limited to the proteins/genes with defined roles in biological processes. As of December 2006 only 3,768 (21%) of the RefList had KEGG annotations. A total of 13,026 (72%) had GO categories. The KEGG pathways are not entirely separate from one another. One example of this is the *MAPK signaling pathway*, which is a constituent component in several other biological pathways, such as the NK pathway. Within the pathways of the KEGG database there are multiple references to other KEGG pathways indicated by a rectangle with rounded corners inserted into the pathway diagrams (as can be seen in Figure 4). This interconnectedness information was manually extracted from the KEGG pathways. The nature and complexity of these interactions varied from pathway to pathway, and for simplicity a line connecting two KEGG pathways was used to represent these interactions. The interaction map was created using CytoScape software to generate a framework of the interactions of the enriched KEGG pathways, and the publication images were produced in Adobe Illustrator (version 12.0.1) [63].

## Authors' contributions

GML designed experiments, prepared RNA samples for microarray and Q-RT-PCR, analyzed data, and drafted the manuscript. GT contributed to the experimental design, statistical analysis, computational aspects, and drafting of the manuscript. SW contributed to the experimental design and data analysis. ZG obtained and processed the control tissue. RB obtained and processed the aneurysmal tissue. HK contributed to the experimental design, data analysis, and drafting of the manuscript. HK also obtained funding for the study. All authors read and approved the final manuscript.

## Supplementary Material

Additional file 1 Genes included in DiffList. Gene symbols, Entrez Gene IDs, gene names, signals, and significance of differential expression provided in a tabular format for all 3,274 genes on the DiffList. Gene symbols and Entrez Gene IDs contain links to the NCBI site. Click here for file

Additional file 2 Top 100 most differential genes. The 100 genes included in Figure 1 arranged alphabetically by symbol with gene symbols, Entrez gene IDs, gene names, signals, p-value, and FDR given for the top 100 genes using the group comparisons from Illumina. Click here for file

Additional file 3 Modified "leukocyte transendothelial migration" (hsa04670) pathway from KEGG. Protein symbols were replaced by gene symbols to reflect gene-centric data. See key for explanation of colors and symbols (see the additional file Table IV for expression values of the genes in both conditions as well as the FDR value for those that were significantly different). Click here for file

Additional file 4 Expression values and significance of differential expression for individual genes included in the NK cytotoxicity pathway in Figure 4. Gene symbols, Entrez Gene IDs, gene names, signals, and significance of differential expression provided in a tabular format for NK cytotoxicity pathway. Gene symbols and Entrez Gene IDs contain links to the NCBI site. Click here for file

Additional file 5 Expression values and significance of differential expression for individual genes included in the LTEM pathway in Figure 5. Gene symbols, Entrez Gene IDs, gene names, signals, and significance of differential expression provided in a tabular format for LTEM pathway. Gene symbols and Entrez Gene IDs contain links to the NCBI site. Click here for file

Additional file 6 Signal intensity histograms. Unadjusted, i.e. raw, signals from microarray experiments were converted into histograms to visualize the lack of patterns of bias. Click here for file

Additional file 7 Principal component analysis. Principal component analysis was carried out on the microarray data to show that there was no systematic bias in the samples. Click here for file
