# Supplementary material for: Whole genome expression profiling reveals a significant role for immune function in human abdominal aortic aneurysms
Source: BMC Genomics. 2007 Jul 16;8:237. doi: 10.1186/1471-2164-8-237 (PMC1934369; doi:10.1186/1471-2164-8-237)
Supplement: Additional file 1 — Genes included in DiffList. Gene symbols, Entrez Gene IDs, gene names, signals, and significance of differential expression provided in a tabular format for all 3,274 genes on the DiffList. Gene symbols and Entrez Gene IDs contain links to the NCBI site. [file 1471-2164-8-237-S1.pdf]

Table I. Differentially Expressed Genes

Symbol	GeneID	Gene Name*	Signal CNTRL	Signal AAA	P	FDR
A2M	2	alpha-2-macroglobulin	12.53	11.07	8.62e-05	8.62e-04
A4GALT	53947	alpha 1,4-galactosyltransferase (globotriaosylcera	8.23	7.00	3.02e-05	3.60e-04
AADACL1	57552	arylacetamide deacetylase-like 1	6.52	8.97	1.46e-06	2.89e-05
AADAT	51166	aminoadipate aminotransferase	6.11	4.90	5.28e-05	5.77e-04
AASS	10157	aminoadipate-semialdehyde synthase	6.94	4.88	2.02e-03	1.11e-02
ABCA1	19	ATP-binding cassette, sub-family A (ABC1), member	9.35	10.64	6.49e-03	2.73e-02
ABCA6	23460	ATP-binding cassette, sub-family A (ABC1), member	7.49	6.61	1.35e-02	4.80e-02
ABCA7	10347	ATP-binding cassette, sub-family A (ABC1), member	4.74	6.26	5.83e-04	4.09e-03
ABCA8	10351	ATP-binding cassette, sub-family A (ABC1), member	9.33	8.05	1.32e-02	4.73e-02
ABCB6	10058	ATP-binding cassette, sub-family B (MDR/TAP), memb	7.49	6.31	3.04e-03	1.54e-02
ABCC5	10057	ATP-binding cassette, sub-family C (CFTR/MRP), mem	6.55	7.44	6.57e-03	2.75e-02
ABCC9	10060	ATP-binding cassette, sub-family C (CFTR/MRP), mem	6.15	4.42	4.18e-04	3.11e-03
ABCD1	215	ATP-binding cassette, sub-family D (ALD), member 1	5.17	6.42	4.26e-03	1.99e-02
ABCF2	10061	ATP-binding cassette, sub-family F (GCN20), member	5.84	4.74	1.15e-03	7.06e-03
ABCG1	9619	ATP-binding cassette, sub-family G (WHITE), member	8.32	9.56	1.23e-03	7.39e-03
ABHD4	63874	abhydrolase domain containing 4	8.69	7.68	9.74e-06	1.41e-04
ABHD6	57406	abhydrolase domain containing 6	7.28	7.99	8.96e-03	3.50e-02
ABI2	10152	abl interactor 2	7.55	6.92	6.97e-03	2.88e-02
ABI3	51225	ABI gene family, member 3	6.79	8.53	9.15e-07	1.96e-05
ABI3BP	25890	ABI gene family, member 3 (NESH) binding protein	9.79	8.63	5.62e-04	3.98e-03
ABLIM1	3983	actin binding LIM protein 1	9.46	8.60	1.05e-02	3.96e-02
ABLIM3	22885	actin binding LIM protein family, member 3	7.26	5.86	4.03e-03	1.91e-02
ACACA	31	acetyl-Coenzyme A carboxylase alpha	7.65	7.00	4.92e-03	2.21e-02
ACAD11	84129	acyl-Coenzyme A dehydrogenase family, member 11	8.29	7.35	9.22e-05	9.08e-04
ACADL	33	acyl-Coenzyme A dehydrogenase, long chain	8.32	5.02	4.16e-06	6.90e-05
ACAT1	38	acetyl-Coenzyme A acetyltransferase 1 (acetoacetyl	10.01	9.16	2.01e-04	1.71e-03
ACE2	59272	angiotensin I converting enzyme (peptidyl-dipeptid	6.07	5.15	1.39e-02	4.91e-02
ACO1	48	aconitase 1, soluble	9.81	9.10	1.77e-03	9.93e-03
ACOX2	8309	acyl-Coenzyme A oxidase 2, branched chain	8.76	7.69	9.79e-06	1.41e-04
ACP2	53	acid phosphatase 2, lysosomal	6.31	7.87	1.39e-04	1.28e-03
ACP5	54	acid phosphatase 5, tartrate resistant	9.24	11.88	1.02e-03	6.45e-03
ACP6	51205	acid phosphatase 6, lysophosphatidic	6.56	5.96	1.16e-02	4.29e-02
ACPL2	92370	acid phosphatase-like 2	8.01	7.12	3.47e-04	2.67e-03
ACRBP	84519	acrosin binding protein	4.18	5.98	7.48e-03	3.05e-02
ACSS2	55902	acyl-CoA synthetase short-chain family member 2	10.05	9.16	7.96e-05	8.07e-04
ACTA2	59	actin, alpha 2, smooth muscle, aorta	14.86	13.08	6.41e-10	4.35e-08
ACTC	70	actin, alpha, cardiac muscle	11.04	7.94	5.57e-11	5.40e-09
ACTG2	72	actin, gamma 2, smooth muscle, enteric	13.07	11.34	1.60e-04	1.44e-03
ACTN1	87	actinin, alpha 1	12.18	10.96	8.30e-07	1.80e-05
ACTN4	81	actinin, alpha 4	10.95	9.94	3.99e-04	2.98e-03
ACTR10	55860	actin-related protein 10 homolog (S. cerevisiae)	9.02	8.43	9.22e-03	3.57e-02
ACTR2	10097	ARP2 actin-related protein 2 homolog (yeast)	7.70	8.47	9.14e-03	3.55e-02
ADA	100	adenosine deaminase	7.49	8.64	3.10e-04	2.44e-03
ADAM17	6868	ADAM metallopeptidase domain 17 (tumor necrosis fa	7.58	8.52	1.07e-03	6.65e-03
ADAM28	10863	ADAM metallopeptidase domain 28	5.23	6.90	4.70e-03	2.14e-02
ADAM8	101	ADAM metallopeptidase domain 8	6.37	9.24	4.68e-03	2.14e-02
ADAMTS5	11096	ADAM metallopeptidase with thrombospondin type 1 m	8.29	6.98	5.34e-04	3.80e-03
ADAMTSL1	92949	ADAMTS-like 1	5.68	4.78	9.71e-03	3.73e-02
ADAMTSL3	57188	ADAMTS-like 3	9.06	6.80	2.97e-12	3.94e-10
ADCK2	90956	aarF domain containing kinase 2	7.71	8.72	5.21e-04	3.73e-03
ADCY6	112	adenylate cyclase 6	7.29	6.12	3.23e-03	1.60e-02
ADCY7	113	adenylate cyclase 7	5.76	7.04	7.27e-05	7.51e-04
ADCY9	115	adenylate cyclase 9	7.46	6.72	4.49e-03	2.07e-02
ADH1A	124	alcohol dehydrogenase 1A (class I), alpha polypept	12.19	9.10	8.30e-14	1.55e-11
ADH1B	125	alcohol dehydrogenase IB (class I), beta polypepti	12.95	9.72	4.10e-08	1.41e-06
ADH5	128	alcohol dehydrogenase 5 (class III), chi polypepti	11.09	8.68	6.51e-15	1.67e-12
ADHFE1	137872	alcohol dehydrogenase, iron containing, 1	8.43	6.69	9.01e-05	8.92e-04
ADI1	55256	acireductone dioxygenase 1	11.89	10.66	1.68e-04	1.49e-03
ADM	133	adrenomedullin	10.83	9.27	1.81e-03	1.01e-02
ADORA2A	135	adenosine A2a receptor	5.62	6.95	1.02e-03	6.42e-03
ADPGK	83440	ADP-dependent glucokinase	7.15	8.81	2.83e-05	3.43e-04
ADRA2C	152	adrenergic, alpha-2C-, receptor	9.84	6.86	9.73e-07	2.05e-05
ADRBK2	157	adrenergic, beta, receptor kinase 2	4.73	6.46	1.50e-05	2.02e-04
ADSSL1	122622	adenylosuccinate synthase like 1	9.71	6.32	3.23e-10	2.42e-08
AEBP1	165	AE binding protein 1	12.36	10.68	1.36e-04	1.25e-03

Symbol	GeneID	Gene Name*	Signal CNTRL	Signal AAA	P	FDR
AEBP2	121536	AE binding protein 2	8.09	6.51	7.08e-10	4.72e-08
AER61	285203	AER61 glycosyltransferase	8.56	7.51	1.04e-03	6.53e-03
AGPAT2	10555	1-acylglycerol-3-phosphate O-acyltransferase 2 (ly	9.82	10.62	1.05e-02	3.96e-02
AGPAT3	56894	1-acylglycerol-3-phosphate O-acyltransferase 3	4.84	6.14	9.07e-05	8.96e-04
AGPAT6	137964	1-acylglycerol-3-phosphate O-acyltransferase 6 (ly	6.21	7.22	5.50e-04	3.90e-03
AGT	183	angiotensinogen (serpin peptidase inhibitor, clade	9.02	6.62	1.21e-05	1.71e-04
AGTR1	185	angiotensin II receptor, type 1	9.01	6.84	2.77e-08	1.03e-06
AHI1	54806	Abelson helper integration site 1	7.47	6.52	4.37e-05	4.90e-04
AHR	196	aryl hydrocarbon receptor	11.95	12.77	9.80e-03	3.76e-02
AIF1	199	allograft inflammatory factor 1	8.10	9.86	2.45e-06	4.46e-05
AIG1	51390	androgen-induced 1	8.61	7.62	1.60e-05	2.12e-04
AIM1	202	absent in melanoma 1	6.60	9.04	2.34e-04	1.95e-03
AK1	203	adenylate kinase 1	8.24	7.12	1.23e-05	1.73e-04
AK2	204	adenylate kinase 2	6.17	7.22	1.17e-03	7.17e-03
AK3L1	205	adenylate kinase 3-like 1	9.87	9.15	1.46e-03	8.52e-03
AKAP1	8165	A kinase (PRKA) anchor protein 1	6.89	5.24	4.47e-08	1.52e-06
AKAP12	9590	A kinase (PRKA) anchor protein (gravin) 12	6.84	5.08	3.00e-05	3.58e-04
AKAP13	11214	A kinase (PRKA) anchor protein 13	8.52	9.24	1.22e-02	4.46e-02
AKAP6	9472	A kinase (PRKA) anchor protein 6	6.26	4.75	1.04e-05	1.48e-04
AKNA	80709	AT-hook transcription factor	6.38	8.10	7.46e-05	7.69e-04
AKR1A1	10327	aldo-keto reductase family 1, member A1 (aldehyde	10.01	11.02	5.56e-04	3.94e-03
AKR1C2	1646	aldo-keto reductase family 1, member C2 (dihydrodi	9.60	8.18	8.62e-04	5.67e-03
AKR1C3	8644	aldo-keto reductase family 1, member C3 (3-alpha h	9.92	8.16	7.18e-06	1.08e-04
AKR1C4	1109	aldo-keto reductase family 1, member C4 (chlordeco	7.52	5.64	3.02e-06	5.29e-05
ALDH1A1	216	aldehyde dehydrogenase 1 family, member A1	10.03	9.33	1.94e-03	1.07e-02
ALDH1B1	219	aldehyde dehydrogenase 1 family, member B1	8.98	6.69	7.29e-07	1.62e-05
ALDH1L1	10840	aldehyde dehydrogenase 1 family, member L1	7.78	4.76	4.29e-18	3.30e-15
ALDH3A2	224	aldehyde dehydrogenase 3 family, member A2	8.76	7.53	8.26e-03	3.29e-02
ALDH3B1	221	aldehyde dehydrogenase 3 family, member B1	6.92	8.48	1.39e-03	8.18e-03
ALDH6A1	4329	aldehyde dehydrogenase 6 family, member A1	8.76	7.30	1.01e-09	6.40e-08
ALDH7A1	501	aldehyde dehydrogenase 7 family, member A1	8.68	7.69	8.79e-05	8.73e-04
ALOX5	240	arachidonate 5-lipoxygenase	9.31	11.25	1.71e-07	4.75e-06
ALOX5AP	241	arachidonate 5-lipoxygenase-activating protein	10.62	12.22	1.06e-02	3.98e-02
ALPK2	115701	alpha-kinase 2	4.56	6.38	1.25e-04	1.16e-03
ALPP	250	alkaline phosphatase, placental (Regan isozyme)	5.92	6.88	6.96e-03	2.88e-02
ALS2CR15	130026	amyotrophic lateral sclerosis 2 (juvenile) chromos	6.47	5.10	3.19e-06	5.57e-05
ALS2CR4	65062	amyotrophic lateral sclerosis 2 (juvenile) chromos	8.03	6.70	1.07e-06	2.23e-05
AMICA1	120425	adhesion molecule, interacts with CXADR antigen 1	7.26	9.83	2.93e-09	1.50e-07
AMIGO2	347902	adhesion molecule with Ig-like domain 2	8.33	7.11	1.66e-04	1.47e-03
AMOT	154796	angiomotin	6.99	5.39	1.28e-04	1.19e-03
AMPD3	272	adenosine monophosphate deaminase (isoform E)	5.95	8.33	3.70e-08	1.31e-06
AMPH	273	amphiphysin (Stiff-Man syndrome with breast cancer	5.54	4.28	5.30e-03	2.34e-02
ANGPT1	284	angiopoietin 1	8.17	6.10	1.52e-05	2.04e-04
ANGPTL1	9068	angiopoietin-like 1	7.90	6.24	9.10e-04	5.89e-03
ANKDD1A	348094	ankyrin repeat and death domain containing 1A	8.44	7.65	8.95e-04	5.81e-03
ANKMY2	57037	ankyrin repeat and MYND domain containing 2	8.78	8.10	2.68e-03	1.39e-02
ANKRD15	23189	ankyrin repeat domain 15	10.02	7.37	8.97e-09	3.89e-07
ANKRD25	25959	ankyrin repeat domain 25	10.35	8.49	1.02e-07	3.11e-06
ANKRD35	148741	ankyrin repeat domain 35	7.19	5.78	1.14e-03	7.03e-03
ANKRD46	157567	ankyrin repeat domain 46	8.85	7.80	4.34e-06	7.13e-05
ANKRD50	57182	ankyrin repeat domain 50	7.77	7.18	1.05e-02	3.97e-02
ANKRD6	22881	ankyrin repeat domain 6	8.02	6.96	9.06e-03	3.52e-02
ANP32B	10541	acidic (leucine-rich) nuclear phosphoprotein 32 fa	11.47	10.91	1.29e-02	4.64e-02
ANTXR1	84168	anthrax toxin receptor 1	12.62	10.99	2.62e-03	1.37e-02
ANTXR2	118429	anthrax toxin receptor 2	10.38	9.78	8.06e-03	3.23e-02
ANXA3	306	annexin A3	7.22	5.95	2.09e-03	1.14e-02
AOAH	313	acyloxyacyl hydrolase (neutrophil)	5.27	7.72	1.79e-08	7.11e-07
AOC3	8639	amine oxidase, copper containing 3 (vascular adhes	9.73	7.25	6.46e-11	6.11e-09
AOX1	316	aldehyde oxidase 1	8.98	7.02	1.80e-04	1.56e-03
AP1B1	162	adaptor-related protein complex 1, beta 1 subunit	7.41	8.66	4.74e-05	5.25e-04
AP1G2	8906	adaptor-related protein complex 1, gamma 2 subunit	7.02	8.31	2.78e-04	2.24e-03
AP2S1	1175	adaptor-related protein complex 2, sigma 1 subunit	9.22	10.04	3.98e-03	1.89e-02
AP3M2	10947	adaptor-related protein complex 3, mu 2 subunit	8.41	7.38	8.16e-06	1.20e-04
APBA2BP	63941	amyloid beta (A4) precursor protein-binding, famil	7.87	7.21	4.24e-03	1.99e-02
APBB1	322	amyloid beta (A4) precursor protein-binding, famil	7.47	6.31	2.13e-04	1.80e-03
APBB1IP	54518	amyloid beta (A4) precursor protein-binding, famil	7.39	8.96	1.13e-05	1.60e-04
APCDD1	147495	adenomatosis polyposis coli down-regulated 1	8.50	7.16	5.05e-03	2.26e-02

Symbol	GeneID	Gene Name*	Signal CNTRL	AAA	P	FDR
APEG1	10290	aortic preferentially expressed gene 1	10.09	6.99	3.14e-17	1.65e-14
APH1A	51107	anterior pharynx defective 1 homolog A (C. elegans	8.86	9.74	2.07e-03	1.13e-02
APH1B	83464	anterior pharynx defective 1 homolog B (C. elegans	8.29	9.50	5.31e-04	3.79e-03
APLP1	333	amyloid beta (A4) precursor-like protein 1	6.01	5.10	8.64e-03	3.41e-02
APOB	338	apolipoprotein B (including Ag(x) antigen)	7.16	4.25	3.33e-07	8.24e-06
APOBEC3A	200315	apolipoprotein B mRNA editing enzyme, catalytic po	11.65	12.43	5.15e-03	2.29e-02
APOBEC3G	60489	apolipoprotein B mRNA editing enzyme, catalytic po	5.37	6.75	2.50e-05	3.09e-04
APOC1	341	apolipoprotein C-I	8.88	12.04	3.29e-03	1.62e-02
APOE	348	apolipoprotein E	10.55	13.19	4.93e-04	3.57e-03
APP	351	amyloid beta (A4) precursor protein (peptidase nex	9.74	8.83	9.77e-03	3.75e-02
APRIN	23047	androgen-induced proliferation inhibitor	7.95	7.02	3.27e-04	2.55e-03
AQP1	358	aquaporin 1 (channel-forming integral protein, 28k	12.02	10.45	2.30e-03	1.23e-02
AQP9	366	aquaporin 9	7.51	10.22	8.71e-03	3.43e-02
AR	367	androgen receptor (dihydrotestosterone receptor; t	7.32	5.15	1.19e-07	3.52e-06
AREG	374	amphiregulin (schwannoma-derived growth factor)	7.59	9.32	2.88e-04	2.30e-03
ARHGAP1	392	Rho GTPase activating protein 1	10.89	9.85	7.62e-06	1.14e-04
ARHGAP10	79658	Rho GTPase activating protein 10	9.12	7.88	9.15e-08	2.84e-06
ARHGAP15	55843	Rho GTPase activating protein 15	7.56	9.22	1.11e-06	2.29e-05
ARHGAP20	57569	Rho GTPase activating protein 20	6.54	5.72	1.61e-03	9.20e-03
ARHGAP25	9938	Rho GTPase activating protein 25	6.30	9.03	9.32e-10	5.98e-08
ARHGAP27	201176	Rho GTPase activating protein 27	7.55	9.69	2.82e-08	1.04e-06
ARHGAP30	257106	Rho GTPase activating protein 30	6.45	9.03	3.70e-08	1.31e-06
ARHGAP4	393	Rho GTPase activating protein 4	8.14	10.58	7.31e-09	3.26e-07
ARHGAP9	64333	Rho GTPase activating protein 9	7.52	10.05	2.40e-08	9.16e-07
ARHGDIB	397	Rho GDP dissociation inhibitor (GDI) beta	12.04	13.23	8.63e-05	8.62e-04
ARHGEF10	9639	Rho guanine nucleotide exchange factor (GEF) 10	8.78	7.50	3.92e-06	6.56e-05
ARHGEF12	23365	Rho guanine nucleotide exchange factor (GEF) 12	7.02	5.32	4.51e-09	2.16e-07
ARHGEF17	9828	Rho guanine nucleotide exchange factor (GEF) 17	8.35	6.37	1.12e-08	4.74e-07
ARHGEF18	23370	rho/rac guanine nucleotide exchange factor (GEF) 1	6.79	8.09	1.21e-04	1.13e-03
ARHGEF9	23229	Cdc42 guanine nucleotide exchange factor (GEF) 9	8.47	7.04	5.83e-09	2.71e-07
ARID3A	1820	AT rich interactive domain 3A (BRIGHT- like)	6.74	8.14	2.67e-03	1.39e-02
ARID3B	10620	AT rich interactive domain 3B (BRIGHT- like)	6.88	8.16	2.10e-03	1.14e-02
ARL1	400	ADP-ribosylation factor-like 1	9.76	8.93	2.96e-04	2.35e-03
ARL3	403	ADP-ribosylation factor-like 3	8.46	7.81	4.61e-03	2.11e-02
ARL4C	10123	ADP-ribosylation factor-like 4C	5.16	7.02	9.49e-07	2.01e-05
ARMC4	55130	armadillo repeat containing 4	5.50	4.64	5.69e-03	2.47e-02
ARMCX1	51309	armadillo repeat containing, X-linked 1	8.49	7.20	4.09e-08	1.41e-06
ARMCX2	9823	armadillo repeat containing, X-linked 2	9.14	7.66	4.32e-06	7.10e-05
ARPC1B	10095	actin related protein 2/3 complex, subunit 1B, 41k	10.35	11.52	3.49e-04	2.68e-03
ARPC3	10094	actin related protein 2/3 complex, subunit 3, 21kD	10.90	11.91	7.34e-04	4.98e-03
ARPC5L	81873	actin related protein 2/3 complex, subunit 5-like	8.54	9.41	8.24e-03	3.29e-02
ARPP-21	10777	cyclic AMP-regulated phosphoprotein, 21 kD	5.24	4.22	1.37e-02	4.87e-02
ARRDC4	91947	arrestin domain containing 4	7.73	9.23	4.87e-04	3.53e-03
ARSJ	79642	arylsulfatase J	7.25	5.63	2.80e-08	1.04e-06
ARTS-1	51752	type 1 tumor necrosis factor receptor shedding ami	4.65	5.76	3.68e-03	1.78e-02
ASAH1	427	N-acylsphingosine amidohydrolase (acid ceramidase)	6.74	7.60	5.41e-03	2.37e-02
ASB16	92591	ankyrin repeat and SOCS box-containing 16	4.91	6.08	5.19e-04	3.72e-03
ASB2	51676	ankyrin repeat and SOCS box-containing 2	8.23	6.93	5.14e-05	5.64e-04
ASCC1	51008	activating signal cointegrator 1 complex subunit 1	8.98	8.31	3.13e-03	1.56e-02
ASCC3	10973	activating signal cointegrator 1 complex subunit 3	8.40	7.69	1.02e-02	3.89e-02
ASF1B	55723	ASF1 anti-silencing function 1 homolog B (S. cerev	4.45	5.35	1.32e-02	4.72e-02
ASGR1	432	asialoglycoprotein receptor 1	5.76	8.42	8.81e-03	3.46e-02
ASGR2	433	asialoglycoprotein receptor 2	4.35	6.00	7.56e-03	3.07e-02
ASPH	444	aspartate beta-hydroxylase	8.93	8.20	1.25e-03	7.50e-03
ASPHD2	57168	aspartate beta-hydroxylase domain containing 2	5.48	7.28	5.33e-07	1.24e-05
ASPN	54829	asporin (LRR class 1)	10.99	8.90	1.25e-06	2.54e-05
ASS	445	argininosuccinate synthetase	9.63	8.10	1.79e-04	1.56e-03
ASTN2	23245	astrotactin 2	6.46	5.51	1.18e-02	4.33e-02
ASXL1	171023	additional sex combs like 1 (Drosophila)	8.42	7.79	5.05e-03	2.26e-02
ATAD1	84896	ATPase family, AAA domain containing 1	9.03	7.99	1.82e-03	1.02e-02
ATBF1	463	AT-binding transcription factor 1	8.47	7.20	2.65e-03	1.38e-02
ATF5	22809	activating transcription factor 5	8.19	9.71	3.61e-04	2.74e-03
ATG16L2	89849	ATG16 autophagy related 16-like 2 (S. cerevisiae)	6.27	8.31	1.13e-02	4.19e-02
ATG5	9474	ATG5 autophagy related 5 homolog (S. cerevisiae)	8.55	7.41	1.31e-04	1.22e-03
ATG7	10533	ATG7 autophagy related 7 homolog (S. cerevisiae)	8.32	9.11	7.10e-03	2.93e-02
ATM	472	ataxia telangiectasia mutated (includes complement	4.73	6.37	3.93e-03	1.87e-02
ATN1	1822	atrophin 1	7.72	6.04	1.55e-04	1.40e-03

Symbol	GeneID	Gene Name*	Signal CNTRL	Signal AAA	P	FDR
ATOH8	84913	atonal homolog 8 (Drosophila)	10.72	8.29	1.47e-10	1.26e-08
ATOX1	475	ATX1 antioxidant protein 1 homolog (yeast)	8.65	9.55	2.90e-03	1.47e-02
ATP10D	57205	ATPase, Class V, type 10D	9.08	8.32	8.67e-04	5.69e-03
ATP13A1	57130	ATPase type 13A1	7.15	8.06	1.33e-03	7.88e-03
ATP13A2	23400	ATPase type 13A2	5.10	6.45	1.01e-04	9.78e-04
ATP1A2	477	ATPase, Na+/K+ transporting, alpha 2 (+) polypepti	8.42	5.95	1.85e-05	2.40e-04
ATP1B1	481	ATPase, Na+/K+ transporting, beta 1 polypeptide	7.25	8.40	9.05e-04	5.87e-03
ATP2A2	488	ATPase, Ca++ transporting, cardiac muscle, slow tw	9.98	9.07	5.19e-04	3.72e-03
ATP2A3	489	ATPase, Ca++ transporting, ubiquitous	5.34	7.82	3.14e-04	2.46e-03
ATP2B1	490	ATPase, Ca++ transporting, plasma membrane 1	6.60	8.21	2.71e-04	2.20e-03
ATP5G3	518	ATP synthase, H+ transporting, mitochondrial F0 co	9.97	10.66	1.29e-02	4.65e-02
ATP6V0B	533	ATPase, H+ transporting, lysosomal 21kDa, V0 subun	9.45	11.00	1.18e-03	7.19e-03
ATP6V0D1	9114	ATPase, H+ transporting, lysosomal 38kDa, V0 subun	8.55	9.73	2.39e-03	1.27e-02
ATP6V1A	523	ATPase, H+ transporting, lysosomal 70kDa, V1 subun	9.63	10.73	9.99e-03	3.82e-02
ATP6V1B2	526	ATPase, H+ transporting, lysosomal 56/58kDa, V1 su	10.22	11.45	6.34e-05	6.71e-04
ATP6V1F	9296	ATPase, H+ transporting, lysosomal 14kDa, V1 subun	8.99	10.11	4.37e-03	2.04e-02
ATP9B	374868	ATPase, Class II, type 9B	5.13	9.06	1.04e-04	1.01e-03
ATPAF1	64756	ATP synthase mitochondrial F1 complex assembly fac	9.91	9.33	1.09e-02	4.07e-02
ATRX	546	alpha thalassemia/mental retardation syndrome X-li	8.87	8.13	1.17e-03	7.18e-03
AUH	549	AU RNA binding protein/enoyl-Coenzyme A hydratase	7.58	6.93	4.41e-03	2.05e-02
AURKB	9212	aurora kinase B	5.18	6.59	2.24e-05	2.81e-04
AUTS2	26053	autism susceptibility candidate 2	8.64	7.06	2.72e-03	1.41e-02
AVEN	57099	apoptosis, caspase activation inhibitor	8.65	7.65	1.33e-05	1.83e-04
AVPI1	60370	arginine vasopressin-induced 1	10.31	9.69	1.37e-02	4.87e-02
AXUD1	64651	AXIN1 up-regulated 1	10.22	11.41	3.22e-03	1.60e-02
B3GALT6	126792	UDP-Gal:betaGal beta 1,3-galactosyltransferase pol	8.21	8.93	7.87e-03	3.18e-02
B3GNT5	84002	UDP-GlcNAc:betaGal beta-1,3-N-acetylglucosaminyltr	5.51	7.64	3.36e-05	3.91e-04
BACE1	23621	beta-site APP-cleaving enzyme 1	8.88	7.98	1.07e-03	6.70e-03
BACE2	25825	beta-site APP-cleaving enzyme 2	10.76	9.73	3.07e-03	1.54e-02
BAG2	9532	BCL2-associated athanogene 2	9.05	7.32	3.22e-05	3.77e-04
BAHCC1	57597	BAH domain and coiled-coil containing 1	7.05	5.70	7.64e-03	3.10e-02
BAMBI	25805	BMP and activin membrane-bound inhibitor homolog (	9.04	7.13	3.28e-06	5.67e-05
BARD1	580	BRCA1 associated RING domain 1	7.49	6.87	1.09e-02	4.07e-02
BATF	10538	basic leucine zipper transcription factor, ATF-lik	4.99	7.16	3.00e-08	1.10e-06
BAZ1A	11177	bromodomain adjacent to zinc finger domain, 1A	8.01	8.95	1.08e-03	6.72e-03
BBS1	582	Bardet-Biedl syndrome 1	7.24	6.24	2.23e-05	2.80e-04
BBS2	583	Bardet-Biedl syndrome 2	8.75	8.13	6.59e-03	2.76e-02
BBS7	55212	Bardet-Biedl syndrome 7	7.58	6.75	2.91e-04	2.31e-03
BBX	56987	bobby sox homolog (Drosophila)	9.38	8.67	4.53e-03	2.09e-02
BCAM	4059	basal cell adhesion molecule (Lu and Au blood grou	7.67	6.45	2.01e-03	1.10e-02
BCAP29	55973	B-cell receptor-associated protein 29	7.47	6.80	1.04e-02	3.95e-02
BCAS3	54828	breast carcinoma amplified sequence 3	6.57	5.68	2.67e-04	2.17e-03
BCCIP	56647	BRCA2 and CDKN1A interacting protein	6.31	4.90	1.75e-03	9.82e-03
BCDIN3	56257	bin3, bicoid-interacting 3, homolog (Drosophila)	8.38	9.09	1.15e-02	4.25e-02
BCKDHB	594	branched chain keto acid dehydrogenase E1, beta po	7.52	6.05	1.57e-08	6.40e-07
BCKDK	10295	branched chain ketoacid dehydrogenase kinase	7.90	8.63	1.11e-02	4.12e-02
BCL11A	53335	B-cell CLL/lymphoma 11A (zinc finger protein)	4.55	8.04	4.10e-03	1.93e-02
BCL2A1	597	BCL2-related protein A1	6.58	9.01	5.62e-03	2.45e-02
BCL2L12	83596	BCL2-like 12 (proline rich)	6.68	7.59	1.34e-03	7.95e-03
BCL2L2	599	BCL2-like 2	9.00	7.87	4.02e-06	6.69e-05
BCL6	604	B-cell CLL/lymphoma 6 (zinc finger protein 51)	10.89	9.77	4.92e-06	7.92e-05
BCL6B	255877	B-cell CLL/lymphoma 6, member B (zinc finger prote	7.30	8.28	1.08e-03	6.74e-03
BCL7A	605	B-cell CLL/lymphoma 7A	5.80	4.81	3.54e-04	2.70e-03
BDH2	56898	3-hydroxybutyrate dehydrogenase, type 2	8.60	7.52	2.74e-06	4.89e-05
BGN	633	biglycan	13.18	11.60	8.95e-03	3.50e-02
BHLHB9	80823	basic helix-loop-helix domain containing, class B,	6.89	5.22	6.70e-07	1.51e-05
BHMT2	23743	betaine-homocysteine methyltransferase 2	7.86	6.13	5.79e-09	2.70e-07
BID	637	BH3 interacting domain death agonist	6.61	8.17	1.31e-03	7.82e-03
BIK	638	BCL2-interacting killer (apoptosis-inducing)	3.85	5.75	2.90e-03	1.47e-02
BIN2	51411	bridging integrator 2	6.37	7.93	3.08e-06	5.39e-05
BIRC1	4671	baculoviral IAP repeat-containing 1	4.80	6.38	4.21e-04	3.12e-03
BIRC3	330	baculoviral IAP repeat-containing 3	9.99	11.36	1.75e-04	1.53e-03
BIRC5	332	baculoviral IAP repeat-containing 5 (survivin)	4.99	5.94	1.32e-02	4.70e-02
BIVM	54841	basic, immunoglobulin-like variable motif containi	6.99	6.25	1.77e-03	9.94e-03
BLNK	29760	B-cell linker	4.37	5.57	1.02e-03	6.46e-03
BLOC1S2	282991	biogenesis of lysosome-related organelles complex-	9.13	10.30	3.05e-03	1.54e-02
BM039	55839	uncharacterized bone marrow protein BM039	5.62	6.34	1.08e-02	4.03e-02

Symbol	GeneID	Gene Name*	Signal CNTRL	AAA	P	FDR
BMP1	649	bone morphogenetic protein 1	8.58	7.73	1.85e-04	1.60e-03
BMP2K	55589	BMP2 inducible kinase	5.40	7.88	8.04e-08	2.53e-06
BMPR1A	657	bone morphogenetic protein receptor, type IA	8.27	6.36	1.37e-10	1.18e-08
BMPR2	659	bone morphogenetic protein receptor, type II (seri	8.41	7.41	8.22e-04	5.44e-03
BNIP3	664	BCL2/adenovirus E1B 19kDa interacting protein 3	9.64	8.98	3.90e-03	1.86e-02
BOMB	80014	BH3-only member B protein	7.91	6.86	1.64e-04	1.47e-03
BRLZ	283234	brain leucine zipper protein	6.46	8.74	1.60e-07	4.52e-06
BSPRY	54836	B-box and SPRY domain containing	4.35	5.59	5.71e-04	4.02e-03
BTBD10	84280	BTB (POZ) domain containing 10	8.44	7.83	6.99e-03	2.89e-02
BTBD11	121551	BTB (POZ) domain containing 11	4.64	6.34	1.13e-04	1.08e-03
BTBD12	84464	BTB (POZ) domain containing 12	6.53	7.53	3.64e-03	1.76e-02
BTC	685	betacellulin	6.26	3.90	7.08e-06	1.07e-04
BTF3	689	basic transcription factor 3	12.53	11.95	1.11e-02	4.14e-02
BTG1	694	B-cell translocation gene 1, anti-proliferative	11.91	12.60	1.05e-02	3.96e-02
BTK	695	Bruton agammaglobulinemia tyrosine kinase	6.06	8.73	1.99e-09	1.10e-07
BTN3A1	11119	butyrophilin, subfamily 3, member A1	6.84	7.88	3.74e-04	2.83e-03
BTN3A3	10384	butyrophilin, subfamily 3, member A3	6.29	7.59	1.35e-04	1.25e-03
BUB1	699	BUB1 budding uninhibited by benzimidazoles 1 homol	4.07	5.44	4.79e-03	2.17e-02
C10orf10	11067	chromosome 10 open reading frame 10	13.11	9.44	6.82e-11	6.40e-09
C10orf116	10974	chromosome 10 open reading frame 116	14.20	11.65	2.89e-09	1.48e-07
C10orf125	282969	chromosome 10 open reading frame 125	6.03	7.27	4.16e-04	3.09e-03
C10orf128	170371	chromosome 10 open reading frame 128	8.48	9.46	7.49e-04	5.04e-03
C10orf35	219738	chromosome 10 open reading frame 35	6.83	5.53	5.06e-07	1.18e-05
C10orf65	112817	chromosome 10 open reading frame 65	7.18	4.79	2.11e-05	2.68e-04
C10orf9	219771	chromosome 10 open reading frame 9	6.62	7.65	5.01e-04	3.62e-03
C10orf97	80013	chromosome 10 open reading frame 97	9.07	8.20	4.43e-04	3.26e-03
C11orf11	747	chromosome 11 open reading frame 11	5.45	6.78	4.15e-03	1.95e-02
C11orf21	29125	chromosome 11 open reading frame 21	4.53	6.32	4.63e-06	7.51e-05
C11orf54	28970	chromosome 11 open reading frame 54	7.12	6.52	1.21e-02	4.41e-02
C12orf23	90488	chromosome 12 open reading frame 23	10.19	9.13	3.47e-06	5.93e-05
C12orf46	121506	chromosome 12 open reading frame 46	4.72	6.48	6.86e-06	1.05e-04
C12orf49	79794	chromosome 12 open reading frame 49	4.93	6.51	5.68e-04	4.01e-03
C12orf58	79738	chromosome 12 open reading frame 58	6.01	4.94	8.25e-03	3.29e-02
C13orf1	57213	chromosome 13 open reading frame 1	7.00	5.85	6.66e-06	1.02e-04
C13orf18	80183	chromosome 13 open reading frame 18	5.43	7.80	1.14e-08	4.80e-07
C14orf128	84837	chromosome 14 open reading frame 128	6.79	5.44	2.85e-07	7.27e-06
C14orf132	56967	chromosome 14 open reading frame 132	8.43	6.25	2.02e-10	1.63e-08
C14orf149	112849	chromosome 14 open reading frame 149	7.11	6.20	5.24e-04	3.75e-03
C14orf159	80017	chromosome 14 open reading frame 159	7.34	8.16	3.20e-03	1.59e-02
C14orf24	283635	chromosome 14 open reading frame 24	9.17	7.84	7.42e-08	2.37e-06
C14orf28	122525	chromosome 14 open reading frame 28	7.73	6.80	5.23e-05	5.72e-04
C14orf58	55640	chromosome 14 open reading frame 58	7.22	9.28	3.59e-04	2.73e-03
C14orf80	283643	chromosome 14 open reading frame 80	6.69	7.58	3.48e-03	1.70e-02
C15orf15	51187	chromosome 15 open reading frame 15	10.83	10.03	9.17e-04	5.92e-03
C15orf41	84529	chromosome 15 open reading frame 41	7.33	6.38	4.04e-05	4.58e-04
C15orf48	84419	chromosome 15 open reading frame 48	5.34	9.71	8.07e-03	3.23e-02
C16orf30	79652	chromosome 16 open reading frame 30	7.95	6.57	2.55e-03	1.34e-02
C16orf33	79622	chromosome 16 open reading frame 33	7.44	8.14	9.32e-03	3.60e-02
C16orf44	79786	chromosome 16 open reading frame 44	7.53	8.22	1.10e-02	4.10e-02
C16orf45	89927	chromosome 16 open reading frame 45	8.48	7.28	9.11e-04	5.89e-03
C16orf5	29965	chromosome 16 open reading frame 5	7.52	6.30	3.78e-07	9.15e-06
C16orf54	283897	chromosome 16 open reading frame 54	4.65	6.77	3.01e-06	5.29e-05
C16orf7	9605	chromosome 16 open reading frame 7	5.05	6.52	1.77e-03	9.92e-03
C17orf56	146705	chromosome 17 open reading frame 56	6.21	7.33	5.60e-03	2.44e-02
C17orf60	284021	chromosome 17 open reading frame 60	5.51	7.10	3.89e-04	2.93e-03
C17orf69	147081	chromosome 17 open reading frame 69	6.03	5.26	2.70e-03	1.40e-02
C17orf70	80233	chromosome 17 open reading frame 70	8.19	9.14	8.80e-04	5.74e-03
C17orf79	55352	chromosome 17 open reading frame 79	9.45	8.48	2.20e-05	2.77e-04
C18orf1	753	chromosome 18 open reading frame 1	8.57	7.62	1.17e-03	7.15e-03
C18orf10	25941	chromosome 18 open reading frame 10	7.98	7.32	4.31e-03	2.01e-02
C19orf24	55009	chromosome 19 open reading frame 24	9.32	10.31	9.97e-04	6.31e-03
C19orf28	126321	chromosome 19 open reading frame 28	5.69	7.71	8.30e-05	8.34e-04
C1GALT1C1	29071	C1GALT1-specific chaperone 1	8.50	7.91	9.04e-03	3.52e-02
C1orf108	79647	chromosome 1 open reading frame 108	7.12	7.95	3.16e-03	1.58e-02
C1orf162	128346	chromosome 1 open reading frame 162	9.45	11.11	8.36e-05	8.39e-04
C1orf163	65260	chromosome 1 open reading frame 163	5.33	6.15	9.85e-03	3.78e-02
C1orf165	79656	chromosome 1 open reading frame 165	7.95	6.77	8.23e-04	5.44e-03

Symbol	GeneID	Gene Name*	Signal CNTRL	AAA	P	FDR
C1orf171	127253	chromosome 1 open reading frame 171	7.25	6.45	5.39e-03	2.37e-02
C1orf181	54680	chromosome 1 open reading frame 181	8.84	8.19	4.52e-03	2.09e-02
C1orf198	84886	chromosome 1 open reading frame 198	9.76	8.23	1.88e-06	3.56e-05
C1orf2	10712	chromosome 1 open reading frame 2	8.39	7.76	5.34e-03	2.35e-02
C1orf24	116496	chromosome 1 open reading frame 24	8.85	7.20	6.15e-07	1.39e-05
C1orf38	9473	chromosome 1 open reading frame 38	7.18	9.75	2.59e-09	1.37e-07
C1orf59	113802	chromosome 1 open reading frame 59	6.39	7.73	2.33e-05	2.90e-04
C1orf85	112770	chromosome 1 open reading frame 85	9.09	9.90	7.22e-03	2.96e-02
C1orf93	127281	chromosome 1 open reading frame 93	6.40	7.89	7.25e-06	1.09e-04
C1QA	712	complement component 1, q subcomponent, A chain	10.89	12.01	9.83e-04	6.25e-03
C1QC	714	complement component 1, q subcomponent, C chain	9.35	10.83	2.26e-03	1.21e-02
C1QTNF1	114897	C1q and tumor necrosis factor related protein 1	9.15	7.57	1.06e-03	6.61e-03
C1QTNF7	114905	C1q and tumor necrosis factor related protein 7	7.57	5.19	2.96e-06	5.25e-05
C1RL	51279	complement component 1, r subcomponent-like	9.05	8.09	2.92e-05	3.52e-04
C1S	716	complement component 1, s subcomponent	12.18	10.92	5.87e-05	6.27e-04
C2	717	complement component 2	7.77	9.41	3.26e-06	5.64e-05
C20orf100	84969	chromosome 20 open reading frame 100	7.56	6.45	7.70e-04	5.16e-03
C20orf103	24141	chromosome 20 open reading frame 103	4.85	7.00	1.15e-02	4.26e-02
C20orf108	116151	chromosome 20 open reading frame 108	9.50	8.43	5.45e-05	5.91e-04
C20orf149	79144	chromosome 20 open reading frame 149	12.93	12.17	1.70e-03	9.58e-03
C20orf160	140706	chromosome 20 open reading frame 160	6.66	5.68	8.54e-03	3.38e-02
C20orf24	55969	chromosome 20 open reading frame 24	9.35	10.25	4.07e-03	1.92e-02
C20orf27	54976	chromosome 20 open reading frame 27	6.88	7.93	3.47e-04	2.67e-03
C20orf3	57136	chromosome 20 open reading frame 3	8.38	9.17	6.14e-03	2.61e-02
C20orf31	55741	chromosome 20 open reading frame 31	8.53	9.24	8.87e-03	3.47e-02
C20orf55	83541	chromosome 20 open reading frame 55	6.18	8.17	1.88e-07	5.14e-06
C20orf59	63910	chromosome 20 open reading frame 59	6.87	8.63	5.42e-07	1.25e-05
C20orf6	51575	chromosome 20 open reading frame 6	7.42	6.64	1.69e-03	9.55e-03
C21orf25	25966	chromosome 21 open reading frame 25	9.37	8.51	4.47e-03	2.07e-02
C21orf34	388815	chromosome 21 open reading frame 34	7.26	5.77	2.31e-07	6.04e-06
C21orf51	54065	chromosome 21 open reading frame 51	6.84	5.67	1.75e-04	1.53e-03
C21orf6	10069	chromosome 21 open reading frame 6	8.87	7.75	3.22e-05	3.77e-04
C21orf63	59271	chromosome 21 open reading frame 63	9.29	8.10	1.71e-04	1.50e-03
C21orf82	114036	chromosome 21 open reading frame 82	6.20	4.48	2.35e-06	4.32e-05
C22orf18	79019	chromosome 22 open reading frame 18	4.31	5.89	1.18e-03	7.21e-03
C2orf11	130132	chromosome 2 open reading frame 11	8.10	6.41	3.51e-09	1.75e-07
C2orf30	27248	chromosome 2 open reading frame 30	9.95	9.24	1.66e-03	9.41e-03
C2orf33	56947	chromosome 2 open reading frame 33	9.49	8.77	1.50e-03	8.69e-03
C3AR1	719	complement component 3a receptor 1	8.17	9.62	2.06e-04	1.75e-03
C3orf18	51161	chromosome 3 open reading frame 18	6.63	5.47	1.19e-05	1.68e-04
C3orf26	84319	chromosome 3 open reading frame 26	7.87	7.19	7.69e-03	3.12e-02
C3orf29	64419	chromosome 3 open reading frame 29	6.73	8.19	6.77e-06	1.03e-04
C3orf40	131408	chromosome 3 open reading frame 40	9.02	8.42	7.95e-03	3.20e-02
C3orf62	375341	chromosome 3 open reading frame 62	4.67	6.11	2.88e-05	3.48e-04
C4orf18	51313	chromosome 4 open reading frame 18	10.25	9.51	1.18e-03	7.20e-03
C5	727	complement component 5	7.99	6.75	4.81e-06	7.75e-05
C5AR1	728	complement component 5a receptor 1	9.06	11.00	8.76e-05	8.72e-04
C5orf20	140947	chromosome 5 open reading frame 20	4.49	6.52	2.64e-03	1.38e-02
C5orf4	10826	chromosome 5 open reading frame 4	9.61	7.92	9.87e-08	3.03e-06
C6orf1	221491	chromosome 6 open reading frame 1	8.58	9.31	7.34e-03	3.00e-02
C6orf105	84830	chromosome 6 open reading frame 105	4.17	5.84	9.48e-03	3.65e-02
C6orf115	58527	chromosome 6 open reading frame 115	7.94	9.63	3.55e-05	4.09e-04
C6orf117	112609	chromosome 6 open reading frame 117	10.42	5.48	7.51e-24	8.67e-20
C6orf145	221749	chromosome 6 open reading frame 145	10.85	9.90	1.99e-03	1.09e-02
C6orf150	115004	chromosome 6 open reading frame 150	4.26	5.83	7.28e-03	2.98e-02
C6orf151	154007	chromosome 6 open reading frame 151	6.12	5.12	1.02e-04	9.92e-04
C6orf152	167691	chromosome 6 open reading frame 152	6.95	6.16	1.06e-02	3.98e-02
C6orf162	57150	chromosome 6 open reading frame 162	5.62	4.71	3.83e-03	1.84e-02
C6orf166	55122	chromosome 6 open reading frame 166	7.97	8.84	1.85e-03	1.03e-02
C6orf168	84553	chromosome 6 open reading frame 168	5.98	4.69	3.24e-03	1.61e-02
C6orf35	55836	chromosome 6 open reading frame 35	6.45	5.10	1.16e-04	1.10e-03
C6orf62	81688	chromosome 6 open reading frame 62	9.26	10.36	2.14e-04	1.80e-03
C6orf75	60487	chromosome 6 open reading frame 75	7.71	6.77	6.47e-05	6.81e-04
C7	730	complement component 7	12.02	10.93	7.36e-03	3.01e-02
C7orf10	79783	chromosome 7 open reading frame 10	8.39	7.06	6.34e-06	9.78e-05
C7orf19	80228	chromosome 7 open reading frame 19	5.40	7.57	3.95e-08	1.37e-06
C7orf29	113763	chromosome 7 open reading frame 29	5.32	6.54	1.45e-04	1.32e-03

Symbol	GeneID	Gene Name*	Signal CNTRL	AAA	P	FDR
C8orf4	56892	chromosome 8 open reading frame 4	8.94	7.04	3.73e-03	1.80e-02
C8orf57	84257	chromosome 8 open reading frame 57	5.85	4.76	2.53e-04	2.07e-03
C8orf70	51101	chromosome 8 open reading frame 70	7.05	6.41	6.31e-03	2.67e-02
C8orf72	90362	chromosome 8 open reading frame 72	7.31	6.05	6.97e-07	1.56e-05
C8orf76	84933	chromosome 8 open reading frame 76	7.51	8.31	3.98e-03	1.89e-02
C9orf125	84302	chromosome 9 open reading frame 125	8.06	5.55	1.38e-09	8.19e-08
C9orf127	51754	chromosome 9 open reading frame 127	7.47	6.31	5.99e-06	9.32e-05
C9orf150	286343	chromosome 9 open reading frame 150	7.45	5.54	8.36e-10	5.42e-08
C9orf3	84909	chromosome 9 open reading frame 3	8.54	6.99	2.40e-04	1.99e-03
C9orf40	55071	chromosome 9 open reading frame 40	6.93	6.25	8.39e-03	3.33e-02
C9orf46	55848	chromosome 9 open reading frame 46	7.14	7.80	1.35e-02	4.81e-02
C9orf5	23731	chromosome 9 open reading frame 5	9.81	8.75	3.87e-06	6.49e-05
C9orf58	83543	chromosome 9 open reading frame 58	9.94	8.35	1.03e-05	1.48e-04
C9orf65	158471	chromosome 9 open reading frame 65	7.49	4.33	1.08e-06	2.24e-05
C9orf90	203245	chromosome 9 open reading frame 90	4.79	5.99	3.88e-03	1.86e-02
CABYR	26256	calcium binding tyrosine-(Y)-phosphorylation regul	7.20	6.21	5.11e-03	2.28e-02
CACHD1	57685	cache domain containing 1	6.51	4.98	2.74e-07	7.05e-06
CACNA1C	775	calcium channel, voltage-dependent, L type, alpha	7.22	5.61	3.02e-10	2.29e-08
CACNA2D3	55799	calcium channel, voltage-dependent, alpha 2/delta	4.34	5.81	1.30e-03	7.77e-03
CACNA2D4	93589	calcium channel, voltage-dependent, alpha 2/delta	6.48	7.93	1.83e-05	2.37e-04
CACNB2	783	calcium channel, voltage-dependent, beta 2 subunit	5.92	4.71	1.42e-03	8.35e-03
CADPS2	93664	Ca2+-dependent activator protein for secretion 2	8.02	6.94	3.57e-04	2.72e-03
CALCOCO1	57658	calcium binding and coiled-coil domain 1	7.98	7.07	3.58e-03	1.74e-02
CALCOCO2	10241	calcium binding and coiled-coil domain 2	7.58	6.11	4.36e-03	2.03e-02
CALCRL	10203	calcitonin receptor-like	8.36	7.65	2.77e-03	1.42e-02
CALD1	800	caldesmon 1	10.36	8.09	3.15e-10	2.38e-08
CALM1	801	calmodulin 1 (phosphorylase kinase, delta)	11.82	10.98	1.98e-04	1.69e-03
CALM3	808	calmodulin 3 (phosphorylase kinase, delta)	7.75	7.01	4.98e-03	2.23e-02
CALU	813	calumenin	11.46	10.61	1.95e-04	1.67e-03
CAMK1D	57118	calcium/calmodulin-dependent protein kinase ID	6.95	7.96	4.88e-04	3.54e-03
CAMK2D	817	calcium/calmodulin-dependent protein kinase (CaM k	7.14	6.28	2.38e-04	1.98e-03
CAMK2G	818	calcium/calmodulin-dependent protein kinase (CaM k	8.43	7.03	4.36e-09	2.12e-07
CAMLG	819	calcium modulating ligand	10.34	9.49	3.14e-04	2.47e-03
CAMSAP1L1	23271	calmodulin regulated spectrin-associated protein 1	9.53	8.29	1.09e-07	3.27e-06
CAND2	23066	cullin-associated and neddylation-dissociated 2 (p	7.76	5.77	6.90e-12	8.47e-10
CAP2	10486	CAP, adenylate cyclase-associated protein, 2 (yeas	7.02	5.16	4.85e-10	3.37e-08
CAPG	822	capping protein (actin filament), gelsolin-like	8.05	10.76	1.02e-03	6.45e-03
CAPN1	823	calpain 1, (mu/I) large subunit	8.99	9.68	1.05e-02	3.96e-02
CAPN7	23473	calpain 7	6.84	6.26	1.36e-02	4.85e-02
CARD11	84433	caspase recruitment domain family, member 11	4.87	7.94	9.76e-04	6.22e-03
CARD12	58484	caspase recruitment domain family, member 12	5.81	8.14	1.62e-04	1.45e-03
CARD8	22900	caspase recruitment domain family, member 8	4.78	6.37	4.00e-04	2.99e-03
CARD9	64170	caspase recruitment domain family, member 9	5.05	7.86	1.72e-06	3.31e-05
CASP3	836	caspase 3, apoptosis-related cysteine peptidase	7.61	8.43	3.03e-03	1.53e-02
CASQ2	845	calsequestrin 2 (cardiac muscle)	8.74	6.51	4.05e-05	4.58e-04
CAV1	857	caveolin 1, caveolae protein, 22kDa	9.09	8.04	2.22e-04	1.86e-03
CAV2	858	caveolin 2	9.81	8.18	1.30e-07	3.79e-06
CBFA2T3	863	core-binding factor, runt domain, alpha subunit 2;	5.08	6.76	4.88e-03	2.20e-02
CBX1	10951	chromobox homolog 1 (HP1 beta homolog Drosophila)	8.52	7.71	3.71e-04	2.81e-03
CBX5	23468	chromobox homolog 5 (HP1 alpha homolog, Drosophila	6.53	5.65	1.71e-03	9.62e-03
CBX7	23492	chromobox homolog 7	8.92	7.93	7.64e-05	7.85e-04
CCDC25	55246	coiled-coil domain containing 25	9.14	8.31	2.75e-04	2.22e-03
CCDC3	83643	coiled-coil domain containing 3	12.37	10.27	1.88e-07	5.14e-06
CCDC46	201134	coiled-coil domain containing 46	7.18	6.04	8.34e-03	3.32e-02
CCDC55	84081	coiled-coil domain containing 55	7.93	7.36	1.25e-02	4.54e-02
CCDC6	8030	coiled-coil domain containing 6	8.25	6.58	8.28e-06	1.22e-04
CCDC66	285331	coiled-coil domain containing 66	6.69	5.82	6.20e-04	4.32e-03
CCDC8	83987	coiled-coil domain containing 8	6.51	4.94	4.39e-09	2.12e-07
CCL2	6347	chemokine (C-C motif) ligand 2	9.05	11.77	1.58e-05	2.11e-04
CCL20	6364	chemokine (C-C motif) ligand 20	5.45	7.46	7.23e-04	4.91e-03
CCL22	6367	chemokine (C-C motif) ligand 22	3.66	7.95	5.50e-05	5.96e-04
CCL4	6351	chemokine (C-C motif) ligand 4	7.04	10.66	3.45e-10	2.57e-08
CCL5	6352	chemokine (C-C motif) ligand 5	8.99	11.05	8.48e-08	2.65e-06
CCL8	6355	chemokine (C-C motif) ligand 8	6.68	10.06	5.04e-03	2.25e-02
CCNB1	891	cyclin B1	5.84	7.35	5.39e-03	2.37e-02
CCNB1IP1	57820	cyclin B1 interacting protein 1	9.62	8.05	9.32e-11	8.28e-09
CCNB2	9133	cyclin B2	4.74	6.26	3.03e-05	3.60e-04

Symbol	GeneID	Gene Name*	Signal CNTRL	AAA	P	FDR
CCND2	894	cyclin D2	10.72	9.90	1.18e-02	4.33e-02
CCNE2	9134	cyclin E2	4.36	5.61	2.57e-03	1.35e-02
CCNG1	900	cyclin G1	8.09	7.19	2.57e-03	1.35e-02
CCNI	10983	cyclin I	12.67	11.69	1.72e-05	2.25e-04
CCNL1	57018	cyclin L1	9.14	10.14	2.02e-03	1.11e-02
CCPG1	9236	cell cycle progression 1	9.34	8.34	1.41e-05	1.92e-04
CCR1	1230	chemokine (C-C motif) receptor 1	8.19	10.78	6.17e-04	4.30e-03
CCR2	1231	chemokine (C-C motif) receptor 2	4.03	7.17	1.16e-09	7.10e-08
CCR5	1234	chemokine (C-C motif) receptor 5	6.23	8.58	2.86e-05	3.46e-04
CCR7	1236	chemokine (C-C motif) receptor 7	4.46	8.93	6.08e-03	2.60e-02
CCRL1	51554	chemokine (C-C motif) receptor-like 1	6.75	4.80	6.98e-06	1.06e-04
CD14	929	CD14 antigen	9.46	11.57	3.07e-07	7.68e-06
CD151	977	CD151 antigen (Raph blood group)	11.77	10.04	5.38e-10	3.69e-08
CD163L1	283316	CD163 antigen-like 1	5.57	7.31	1.33e-03	7.88e-03
CD1C	911	CD1C antigen, c polypeptide	4.54	6.90	1.19e-03	7.24e-03
CD1D	912	CD1D antigen, d polypeptide	5.94	7.85	2.16e-05	2.73e-04
CD2	914	CD2 antigen (p50), sheep red blood cell receptor	4.87	8.51	5.69e-04	4.01e-03
CD200	4345	CD200 antigen	9.04	8.13	2.41e-03	1.28e-02
CD244	51744	CD244 natural killer cell receptor 2B4	5.09	6.33	1.39e-04	1.28e-03
CD300A	11314	CD300A antigen	6.06	8.81	3.10e-03	1.55e-02
CD300C	10871	CD300C antigen	6.33	9.43	4.58e-03	2.10e-02
CD300LB	124599	CD300 antigen like family member B	4.45	7.61	1.18e-02	4.33e-02
CD33	945	CD33 antigen (gp67)	6.99	8.81	5.60e-05	6.04e-04
CD37	951	CD37 antigen	7.84	10.78	1.60e-06	3.12e-05
CD3D	915	CD3D antigen, delta polypeptide (TiT3 complex)	5.77	8.53	6.09e-03	2.60e-02
CD3E	916	CD3E antigen, epsilon polypeptide (TiT3 complex)	6.11	8.97	1.26e-02	4.55e-02
CD3G	917	CD3G antigen, gamma polypeptide (TiT3 complex)	4.95	7.04	1.07e-02	4.01e-02
CD3Z	919	CD3Z antigen, zeta polypeptide (TiT3 complex)	6.75	9.09	3.86e-03	1.85e-02
CD4	920	CD4 antigen (p55)	6.85	8.88	1.04e-07	3.15e-06
CD44	960	CD44 antigen (Indian blood group)	9.07	9.75	1.15e-02	4.25e-02
CD47	961	CD47 antigen (Rh-related antigen, integrin-associa	8.91	9.62	9.09e-03	3.53e-02
CD48	962	CD48 antigen (B-cell membrane protein)	7.18	9.95	9.39e-05	9.21e-04
CD5	921	CD5 antigen (p56-62)	4.72	6.68	8.55e-04	5.63e-03
CD52	1043	CD52 antigen (CAMPATH-1 antigen)	6.06	10.42	3.01e-05	3.59e-04
CD53	963	CD53 antigen	8.67	11.34	4.13e-09	2.01e-07
CD59	966	CD59 antigen, complement regulatory protein	11.81	10.86	3.10e-05	3.67e-04
CD68	968	CD68 antigen	6.46	9.29	5.12e-03	2.28e-02
CD7	924	CD7 antigen (p41)	4.36	6.46	5.77e-03	2.50e-02
CD72	971	CD72 antigen	5.20	7.43	2.49e-08	9.47e-07
CD74	972	CD74 antigen (invariant polypeptide of major histo	12.55	13.77	1.71e-04	1.50e-03
CD83	9308	CD83 antigen (activated B lymphocytes, immunoglobu	7.27	10.62	6.46e-09	2.91e-07
CD84	8832	CD84 antigen (leukocyte antigen)	4.86	8.11	9.08e-06	1.32e-04
CD86	942	CD86 antigen (CD28 antigen ligand 2, B7-2 antigen)	4.99	7.44	7.97e-05	8.08e-04
CD8A	925	CD8 antigen, alpha polypeptide (p32)	6.48	8.61	3.36e-03	1.65e-02
CD96	10225	CD96 antigen	4.39	6.30	1.21e-02	4.43e-02
CDC20	991	CDC20 cell division cycle 20 homolog (S. cerevisia	6.13	8.33	3.48e-04	2.67e-03
CDC37L1	55664	CDC37 cell division cycle 37 homolog (S. cerevisia	9.12	8.15	6.30e-05	6.68e-04
CDC42EP1	11135	CDC42 effector protein (Rho GTPase binding) 1	8.20	7.28	5.24e-03	2.32e-02
CDC42EP4	23580	CDC42 effector protein (Rho GTPase binding) 4	10.09	9.38	8.81e-03	3.46e-02
CDC42EP5	148170	CDC42 effector protein (Rho GTPase binding) 5	9.25	8.00	2.96e-06	5.25e-05
CDC42SE1	56882	CDC42 small effector 1	6.97	7.85	2.35e-03	1.25e-02
CDC42SE2	56990	CDC42 small effector 2	4.92	6.40	2.05e-04	1.74e-03
CDC45L	8318	CDC45 cell division cycle 45-like (S. cerevisiae)	4.48	6.00	2.52e-04	2.07e-03
CDC7	8317	CDC7 cell division cycle 7 (S. cerevisiae)	4.39	5.56	1.05e-02	3.96e-02
CDCA4	55038	cell division cycle associated 4	6.40	7.65	9.00e-04	5.85e-03
CDCA7	83879	cell division cycle associated 7	5.66	6.71	7.47e-03	3.05e-02
CDCA7L	55536	cell division cycle associated 7-like	6.68	8.07	1.23e-05	1.73e-04
CDCA8	55143	cell division cycle associated 8	4.87	5.93	1.73e-03	9.71e-03
CDGAP	57514	Cdc42 GTPase-activating protein	5.72	6.58	3.21e-03	1.60e-02
CDH13	1012	cadherin 13, H-cadherin (heart)	8.38	5.77	1.63e-07	4.57e-06
CDH2	1000	cadherin 2, type 1, N-cadherin (neuronal)	9.42	7.96	1.54e-03	8.88e-03
CDK5	1020	cyclin-dependent kinase 5	7.03	8.01	5.34e-03	2.35e-02
CDK5RAP2	55755	CDK5 regulatory subunit associated protein 2	8.58	7.37	1.53e-05	2.06e-04
CDO1	1036	cysteine dioxygenase, type I	8.73	6.38	4.03e-10	2.91e-08
CDR2	1039	cerebellar degeneration-related protein 2, 62kDa	8.93	8.13	1.37e-02	4.86e-02
CDS1	1040	CDP-diacylglycerol synthase (phosphatide cytidyl	5.56	6.50	2.75e-03	1.42e-02
CEBPA	1050	CCAAT/enhancer binding protein (C/EBP), alpha	7.07	9.34	7.38e-04	4.99e-03

Symbol	GeneID	Gene Name*	Signal CNTRL	AAA	P	FDR
CEBPD	1052	CCAAT/enhancer binding protein (C/EBP), delta	12.79	11.72	2.22e-05	2.79e-04
CECR1	51816	cat eye syndrome chromosome region, candidate 1	8.53	10.68	3.39e-08	1.22e-06
CENTA1	11033	centaurin, alpha 1	7.86	10.03	6.93e-04	4.74e-03
CENTA2	55803	centaurin, alpha 2	8.38	10.53	1.06e-03	6.64e-03
CENTB1	9744	centaurin, beta 1	5.64	8.41	4.72e-04	3.45e-03
CENTB2	23527	centaurin, beta 2	5.43	7.04	3.52e-06	5.99e-05
CENTD1	116984	centaurin, delta 1	4.26	5.83	3.18e-05	3.74e-04
CENTD2	116985	centaurin, delta 2	6.54	7.75	1.43e-03	8.35e-03
CENTG3	116988	centaurin, gamma 3	7.23	7.95	8.72e-03	3.43e-02
CEP70	80321	centrosomal protein 70kDa	5.81	4.77	2.76e-03	1.42e-02
CERK	64781	ceramide kinase	8.21	8.99	4.46e-03	2.06e-02
CETN2	1069	centrin, EF-hand protein, 2	10.20	9.49	1.56e-03	8.98e-03
CFDP1	10428	craniofacial development protein 1	9.39	7.93	5.29e-06	8.43e-05
CFH	3075	complement factor H	9.33	8.25	7.90e-04	5.26e-03
CFL2	1073	cofilin 2 (muscle)	9.68	7.73	2.40e-12	3.23e-10
CGI-09	51605	CGI-09 protein	6.02	6.79	6.03e-03	2.58e-02
CGI-14	51005	CGI-14 protein	6.13	7.58	4.10e-04	3.05e-03
CHES1	1112	checkpoint suppressor 1	11.06	10.13	4.09e-05	4.62e-04
CHFR	55743	checkpoint with forkhead and ring finger domains	7.30	8.53	6.03e-05	6.41e-04
CHKB	1120	choline kinase beta	8.48	9.27	5.10e-03	2.27e-02
CHMP4C	92421	chromatin modifying protein 4C	5.77	4.37	3.78e-05	4.32e-04
CHODL	140578	chondrolectin	7.08	5.04	1.64e-04	1.47e-03
CHPF	79586	chondroitin polymerizing factor	8.08	6.97	2.81e-03	1.44e-02
CHST1	8534	carbohydrate (keratan sulfate Gal-6) sulfotransfer	6.63	5.11	2.61e-08	9.79e-07
CHST11	50515	carbohydrate (chondroitin 4) sulfotransferase 11	5.78	7.35	1.42e-05	1.93e-04
CHST13	166012	carbohydrate (chondroitin 4) sulfotransferase 13	5.29	8.81	1.25e-02	4.53e-02
CHST3	9469	carbohydrate (chondroitin 6) sulfotransferase 3	7.95	6.56	5.34e-04	3.80e-03
CHTF18	63922	CTF18, chromosome transmission fidelity factor 18	6.43	7.33	1.56e-03	8.97e-03
CHURC1	91612	churchill domain containing 1	8.88	7.89	4.54e-03	2.09e-02
CIAS1	114548	cold autoinflammatory syndrome 1	6.16	8.95	3.32e-05	3.87e-04
CIB1	10519	calcium and integrin binding 1 (calmyrin)	9.89	11.18	5.68e-03	2.47e-02
CIITA	4261	class II, major histocompatibility complex, transa	4.59	6.33	3.49e-04	2.68e-03
CITED4	163732	Cbp/p300-interacting transactivator, with Glu/Asp-	9.34	8.74	1.15e-02	4.25e-02
CKAP2	26586	cytoskeleton associated protein 2	4.41	5.74	3.63e-03	1.76e-02
CKAP5	9793	cytoskeleton associated protein 5	8.38	7.81	1.25e-02	4.53e-02
CKLF	51192	chemokine-like factor	6.18	7.41	1.68e-04	1.49e-03
CKMT2	1160	creatine kinase, mitochondrial 2 (sarcomeric)	9.07	6.38	2.14e-18	1.90e-15
CKS2	1164	CDC28 protein kinase regulatory subunit 2	4.31	5.49	1.05e-02	3.96e-02
CLCN7	1186	chloride channel 7	9.67	11.04	5.04e-04	3.64e-03
CLDN12	9069	claudin 12	8.44	6.89	3.01e-08	1.10e-06
CLEC11A	6320	C-type lectin domain family 11, member A	7.67	8.50	2.78e-03	1.43e-02
CLEC12A	160364	C-type lectin domain family 12, member A	4.87	6.79	3.82e-03	1.84e-02
CLEC2D	29121	C-type lectin domain family 2, member D	5.99	7.25	6.49e-03	2.73e-02
CLEC4A	50856	C-type lectin domain family 4, member A	5.96	7.91	3.04e-04	2.40e-03
CLEC4F	165530	C-type lectin domain family 4, member F	6.65	8.60	1.07e-03	6.70e-03
CLEC5A	23601	C-type lectin domain family 5, member A	6.38	9.10	1.22e-02	4.45e-02
CLIC1	1192	chloride intracellular channel 1	11.17	11.90	7.42e-03	3.03e-02
CLIC4	25932	chloride intracellular channel 4	12.37	10.48	4.52e-12	5.67e-10
CLIPR-59	25999	CLIP-170-related protein	9.82	8.47	3.46e-04	2.67e-03
CLK1	1195	CDC-like kinase 1	10.36	9.74	6.39e-03	2.69e-02
CLK4	57396	CDC-like kinase 4	8.46	7.87	9.21e-03	3.57e-02
CLMN	79789	calmin (calponin-like, transmembrane)	6.62	7.72	2.72e-04	2.20e-03
CLN6	54982	ceroid-lipofuscinosis, neuronal 6, late infantile,	8.23	9.97	1.09e-06	2.25e-05
CLSTN2	64084	calsyntenin 2	6.49	5.60	4.72e-03	2.14e-02
CLU	1191	clusterin	13.15	11.68	4.29e-06	7.07e-05
CLUAP1	23059	clusterin associated protein 1	6.58	5.48	5.95e-05	6.35e-04
CMIP	80790	c-Maf-inducing protein	9.24	10.01	7.49e-03	3.05e-02
CMTM3	123920	CKLF-like MARVEL transmembrane domain containing	8.76	9.96	9.28e-05	9.12e-04
CMTM6	54918	CKLF-like MARVEL transmembrane domain containing	8.88	10.03	1.21e-04	1.14e-03
CMTM7	112616	CKLF-like MARVEL transmembrane domain containing	4.96	7.04	1.52e-07	4.36e-06
CNDP2	55748	CNDP dipeptidase 2 (metallopeptidase M20 family)	8.25	9.75	1.18e-05	1.66e-04
CNN1	1264	calponin 1, basic, smooth muscle	11.35	9.47	4.76e-04	3.48e-03
CNN2	1265	calponin 2	7.36	8.19	3.10e-03	1.55e-02
CNN3	1266	calponin 3, acidic	11.07	9.68	1.07e-05	1.53e-04
CNR1	1268	cannabinoid receptor 1 (brain)	7.75	5.73	5.43e-03	2.38e-02
CNTN1	1272	contactin 1	5.38	3.98	6.30e-03	2.66e-02
CNTN4	152330	contactin 4	6.88	4.88	8.30e-07	1.80e-05

Symbol	GeneID	Gene Name*	Signal CNTRL	AAA	P	FDR
CNTNAP3	79937	contactin associated protein-like 3	5.67	4.73	1.22e-02	4.45e-02
COBLL1	22837	COBL-like 1	9.10	8.06	1.48e-03	8.59e-03
COL13A1	1305	collagen, type XIII, alpha 1	7.27	5.77	1.31e-06	2.66e-05
COL18A1	80781	collagen, type XVIII, alpha 1	9.17	7.45	1.60e-05	2.12e-04
COL21A1	81578	collagen, type XXI, alpha 1	8.67	6.25	2.14e-07	5.74e-06
COL23A1	91522	collagen, type XXIII, alpha 1	4.93	6.77	6.23e-07	1.41e-05
COL4A1	1282	collagen, type IV, alpha 1	12.46	11.15	8.90e-06	1.30e-04
COL4A2	1284	collagen, type IV, alpha 2	11.08	9.39	1.07e-04	1.03e-03
COL4A5	1287	collagen, type IV, alpha 5 (Alport syndrome)	8.87	6.60	6.34e-15	1.66e-12
COL6A1	1291	collagen, type VI, alpha 1	10.62	9.58	7.75e-03	3.14e-02
COL6A2	1292	collagen, type VI, alpha 2	13.08	11.84	2.42e-05	3.00e-04
COL8A1	1295	collagen, type VIII, alpha 1	12.23	10.25	2.89e-09	1.48e-07
COL8A2	1296	collagen, type VIII, alpha 2	8.91	8.13	4.88e-03	2.20e-02
COMMD9	29099	COMM domain containing 9	7.72	8.94	7.01e-05	7.28e-04
COP1	114769	caspase-1 dominant-negative inhibitor pseudo-ICE	7.83	8.93	1.18e-03	7.20e-03
COPS2	9318	COP9 constitutive photomorphogenic homolog subunit	9.30	8.35	9.00e-05	8.92e-04
COPS8	10920	COP9 constitutive photomorphogenic homolog subunit	10.26	9.57	2.25e-03	1.21e-02
COPZ2	51226	coatomer protein complex, subunit zeta 2	8.76	7.88	6.78e-03	2.82e-02
COQ2	27235	coenzyme Q2 homolog, prenyltransferase (yeast)	7.65	9.03	2.83e-03	1.45e-02
CORO1A	11151	coronin, actin binding protein, 1A	6.39	9.51	1.54e-09	8.93e-08
CORO2A	7464	coronin, actin binding protein, 2A	5.28	6.39	4.83e-03	2.18e-02
CORO6	84940	coronin 6	6.44	4.81	7.37e-04	4.99e-03
CORO7	79585	coronin 7	5.97	7.50	4.10e-06	6.81e-05
COTL1	23406	coactosin-like 1 (Dictyostelium)	10.04	12.14	5.01e-08	1.69e-06
COX6A2	1339	cytochrome c oxidase subunit VIa polypeptide 2	5.81	4.86	1.06e-02	3.99e-02
COX7A1	1346	cytochrome c oxidase subunit VIIa polypeptide 1 (m	9.87	8.55	2.52e-04	2.07e-03
CP	1356	ceruloplasmin (ferroxidase)	9.83	7.68	8.34e-04	5.50e-03
CPE	1363	carboxypeptidase E	12.61	10.40	1.02e-09	6.40e-08
CPNE3	8895	copine III	10.39	9.67	1.55e-03	8.90e-03
CPS1	1373	carbamoyl-phosphate synthetase 1, mitochondrial	6.16	4.80	2.29e-05	2.87e-04
CPT2	1376	carnitine palmitoyltransferase II	7.33	8.09	5.80e-03	2.50e-02
CPVL	54504	carboxypeptidase, vitellogenic-like	9.05	11.02	1.29e-07	3.78e-06
CPXM2	119587	carboxypeptidase X (M14 family), member 2	10.26	8.90	2.76e-04	2.23e-03
CRABP2	1382	cellular retinoic acid binding protein 2	5.64	7.32	5.82e-04	4.09e-03
CRBN	51185	cereblon	9.01	8.42	9.26e-03	3.58e-02
CRELD1	78987	cysteine-rich with EGF-like domains 1	8.87	7.78	4.99e-03	2.24e-02
CRI1	23741	CREBBP/EP300 inhibitor 1	12.68	11.59	2.08e-06	3.89e-05
CRIM1	51232	cysteine rich transmembrane BMP regulator 1 (chord	7.92	5.77	1.34e-09	8.04e-08
CRIP2	1397	cysteine-rich protein 2	10.71	9.23	1.33e-02	4.74e-02
CRISPLD1	83690	cysteine-rich secretory protein LCCL domain contai	8.80	5.76	1.72e-05	2.25e-04
CRKRS	51755	Cdc2-related kinase, arginine/serine-rich	6.62	8.37	2.11e-03	1.15e-02
CRLF1	9244	cytokine receptor-like factor 1	9.69	7.16	1.22e-15	4.02e-13
CRLF3	51379	cytokine receptor-like factor 3	7.33	8.62	2.29e-04	1.91e-03
CRSP3	9439	cofactor required for Sp1 transcriptional activati	5.43	6.66	9.19e-05	9.06e-04
CRSP6	9440	cofactor required for Sp1 transcriptional activati	4.57	5.47	6.54e-03	2.74e-02
CRTAP	10491	cartilage associated protein	9.23	7.64	6.96e-11	6.43e-09
CRYAB	1410	crystallin, alpha B	12.72	10.20	3.32e-15	9.34e-13
CRYZL1	9946	crystallin, zeta (quinone reductase)-like 1	6.00	4.82	5.68e-05	6.12e-04
CSDA	8531	cold shock domain protein A	12.14	11.16	1.84e-05	2.39e-04
CSDE1	7812	cold shock domain containing E1, RNA-binding	11.06	10.42	4.83e-03	2.18e-02
CSE1L	1434	CSE1 chromosome segregation 1-like (yeast)	7.81	6.71	4.65e-05	5.16e-04
CSF1R	1436	colony stimulating factor 1 receptor, formerly McD	9.88	11.85	2.37e-07	6.16e-06
CSF2RA	1438	colony stimulating factor 2 receptor, alpha, low-a	6.17	7.86	1.26e-05	1.75e-04
CSK	1445	c-src tyrosine kinase	6.32	8.92	2.03e-09	1.12e-07
CSNK1D	1453	casein kinase 1, delta	9.25	10.16	1.40e-03	8.22e-03
CSNK1G2	1455	casein kinase 1, gamma 2	6.79	7.63	2.67e-03	1.39e-02
CSNK2A2	1459	casein kinase 2, alpha prime polypeptide	8.07	7.11	3.04e-05	3.61e-04
CSPG4	1464	chondroitin sulfate proteoglycan 4 (melanoma-assoc	8.15	5.81	3.48e-06	5.93e-05
CSPG6	9126	chondroitin sulfate proteoglycan 6 (bamacan)	8.09	7.41	2.74e-03	1.41e-02
CSRP1	1465	cysteine and glycine-rich protein 1	13.84	12.04	2.00e-09	1.10e-07
CSRP2	1466	cysteine and glycine-rich protein 2	11.01	8.71	1.17e-10	1.02e-08
CSS3	337876	chondroitin sulfate synthase 3	8.90	7.03	5.58e-07	1.28e-05
CSTA	1475	cystatin A (stefin A)	6.88	8.37	3.90e-03	1.86e-02
CTA-126B4.3	27341	CGI-96 protein	6.84	7.84	1.30e-02	4.67e-02
CTBP2	1488	C-terminal binding protein 2	9.58	8.79	4.83e-04	3.51e-03
CTDP1	9150	CTD (carboxy-terminal domain, RNA polymerase II, p	6.78	7.84	3.37e-04	2.62e-03
CTDSPL	10217	CTD (carboxy-terminal domain, RNA polymerase II, p	8.09	6.90	1.19e-03	7.24e-03

Symbol	GeneID	Gene Name*	Signal CNTRL	Signal AAA	P	FDR
CTLA4	1493	cytotoxic T-lymphocyte-associated protein 4	4.62	6.94	6.50e-04	4.49e-03
CTNNAL1	8727	catenin (cadherin-associated protein), alpha-like	8.80	7.71	6.64e-05	6.97e-04
CTNS	1497	cystinosis, nephropathic	6.60	7.40	4.05e-03	1.92e-02
CTPS	1503	CTP synthase	7.73	6.23	2.41e-08	9.19e-07
CTPS2	56474	CTP synthase II	8.05	7.38	3.33e-03	1.64e-02
CTSB	1508	cathepsin B	11.62	13.19	7.47e-04	5.04e-03
CTSC	1075	cathepsin C	9.14	10.36	1.12e-04	1.07e-03
CTSG	1511	cathepsin G	7.03	9.52	4.82e-03	2.18e-02
CTSH	1512	cathepsin H	7.56	10.21	1.60e-09	9.12e-08
CTSL	1514	cathepsin L	4.58	6.22	4.82e-03	2.18e-02
CTSS	1520	cathepsin S	9.21	11.58	3.72e-05	4.26e-04
CTSW	1521	cathepsin W (lymphopain)	5.12	6.36	3.63e-03	1.76e-02
CTTN	2017	cortactin	9.71	8.87	4.53e-03	2.09e-02
CTTNBP2NL	55917	CTTNBP2 N-terminal like	6.93	6.06	4.96e-03	2.23e-02
CUGBP1	10658	CUG triplet repeat, RNA binding protein 1	6.08	5.29	9.03e-03	3.52e-02
CWF19L1	55280	CWF19-like 1, cell cycle control (S. pombe)	8.18	8.91	7.78e-03	3.15e-02
CWF19L2	143884	CWF19-like 2, cell cycle control (S. pombe)	8.80	7.87	5.24e-05	5.73e-04
CX3CL1	6376	chemokine (C-X3-C motif) ligand 1	7.54	4.85	1.06e-04	1.02e-03
CXCL1	2919	chemokine (C-X-C motif) ligand 1 (melanoma growth	7.55	9.14	3.03e-04	2.39e-03
CXCL16	58191	chemokine (C-X-C motif) ligand 16	9.65	11.42	8.81e-04	5.74e-03
CXCL2	2920	chemokine (C-X-C motif) ligand 2	7.25	10.37	5.92e-04	4.15e-03
CXCL3	2921	chemokine (C-X-C motif) ligand 3	5.46	7.43	3.91e-05	4.45e-04
CXCL5	6374	chemokine (C-X-C motif) ligand 5	4.76	8.79	3.80e-04	2.87e-03
CXCR3	2833	chemokine (C-X-C motif) receptor 3	3.97	6.66	9.36e-03	3.61e-02
CXCR4	7852	chemokine (C-X-C motif) receptor 4	10.63	12.32	2.69e-06	4.84e-05
CXCR6	10663	chemokine (C-X-C motif) receptor 6	4.58	6.05	8.42e-04	5.55e-03
CXorf21	80231	chromosome X open reading frame 21	4.29	5.74	2.36e-03	1.25e-02
CXorf34	79979	chromosome X open reading frame 34	4.91	6.10	3.52e-04	2.70e-03
CXorf38	159013	chromosome X open reading frame 38	6.42	7.78	9.85e-04	6.26e-03
CXorf41	139212	chromosome X open reading frame 41	11.59	12.40	1.39e-02	4.90e-02
CXorf44	90736	chromosome X open reading frame 44	7.37	6.42	4.37e-05	4.90e-04
CXorf9	54440	chromosome X open reading frame 9	5.36	6.41	1.19e-03	7.24e-03
CXXC4	80319	CXXC finger 4	6.71	5.10	7.73e-04	5.18e-03
CYB561D2	11068	cytochrome b-561 domain containing 2	6.35	7.97	1.77e-06	3.38e-05
CYB5R3	1727	cytochrome b5 reductase 3	10.22	9.02	8.76e-04	5.72e-03
CYB5R4	51167	cytochrome b5 reductase 4	8.00	9.36	8.34e-03	3.32e-02
CYBA	1535	cytochrome b-245, alpha polypeptide	9.39	11.78	1.77e-08	7.07e-07
CYBASC3	220002	cytochrome b, ascorbate dependent 3	7.22	8.45	5.79e-05	6.21e-04
CYBB	1536	cytochrome b-245, beta polypeptide (chronic granul	7.51	9.52	1.53e-07	4.36e-06
CYBRD1	79901	cytochrome b reductase 1	11.92	10.09	1.93e-11	2.12e-09
CYHR1	50626	cysteine/histidine-rich 1	9.45	8.33	3.38e-06	5.82e-05
CYP26B1	56603	cytochrome P450, family 26, subfamily B, polypepti	7.13	5.47	1.12e-02	4.18e-02
CYP2S1	29785	cytochrome P450, family 2, subfamily S, polypeptid	4.39	7.08	7.00e-04	4.78e-03
CYP4B1	1580	cytochrome P450, family 4, subfamily B, polypeptid	9.78	5.60	5.57e-03	2.43e-02
CYP4F11	57834	cytochrome P450, family 4, subfamily F, polypeptid	5.69	4.67	1.01e-02	3.84e-02
CYP4F3	4051	cytochrome P450, family 4, subfamily F, polypeptid	6.68	8.06	2.81e-03	1.44e-02
CYP4X1	260293	cytochrome P450, family 4, subfamily X, polypeptid	8.18	6.30	9.73e-09	4.17e-07
CYSLTR1	10800	cysteinyl leukotriene receptor 1	5.31	6.75	2.84e-03	1.45e-02
CYTL1	54360	cytokine-like 1	8.35	6.83	1.19e-04	1.13e-03
CYYR1	116159	cysteine/tyrosine-rich 1	8.15	7.24	1.00e-02	3.82e-02
DAAM1	23002	dishevelled associated activator of morphogenesis	8.62	7.85	2.19e-03	1.18e-02
DAAM2	23500	dishevelled associated activator of morphogenesis	9.47	7.69	6.81e-09	3.06e-07
DAB2IP	153090	DAB2 interacting protein	6.79	5.82	6.24e-04	4.34e-03
DAG1	1605	dystroglycan 1 (dystrophin-associated glycoprotein	9.46	8.42	1.55e-05	2.07e-04
DAPK1	1612	death-associated protein kinase 1	7.96	9.93	9.19e-06	1.34e-04
DAPP1	27071	dual adaptor of phosphotyrosine and 3-phosphoinosi	7.28	8.64	1.07e-03	6.67e-03
DBC1	1620	deleted in bladder cancer 1	5.62	4.45	1.35e-04	1.24e-03
DBF4	10926	DBF4 homolog (S. cerevisiae)	8.19	9.23	6.53e-03	2.74e-02
DBN1	1627	drebrin 1	9.55	8.47	1.93e-05	2.48e-04
DBNL	28988	drebrin-like	8.84	9.91	2.77e-04	2.23e-03
DBR1	51163	debranching enzyme homolog 1 (S. cerevisiae)	6.06	6.76	1.08e-02	4.04e-02
DBT	1629	dihydrolipoamide branched chain transacylase E2	8.70	7.84	1.57e-04	1.42e-03
DCAL1	160365	dendritic cell-associated lectin-1	5.02	7.23	2.63e-03	1.37e-02
DCAMKL2	166614	doublecortin and CaM kinase-like 2	6.64	5.64	5.81e-03	2.51e-02
DCBLD2	131566	discoidin, CUB and LCCL domain containing 2	8.18	7.23	1.36e-04	1.25e-03
DCHS1	8642	dachsous 1 (Drosophila)	6.28	5.12	4.71e-03	2.14e-02
DCK	1633	deoxycytidine kinase	6.93	7.68	8.55e-03	3.38e-02

Symbol	GeneID	Gene Name*	Signal CNTRL	AAA	P	FDR
DCLRE1B	64858	DNA cross-link repair 1B (PSO2 homolog, S. cerevis	4.62	6.17	5.35e-03	2.36e-02
DCTN6	10671	dynactin 6	9.06	8.37	2.54e-03	1.34e-02
DCUN1D4	23142	DCN1, defective in cullin neddylation 1, domain co	9.87	8.56	2.15e-07	5.74e-06
DDAH1	23576	dimethylarginine dimethylaminohydrolase 1	9.77	8.43	9.68e-06	1.40e-04
DDEF2	8853	development and differentiation enhancing factor 2	8.81	7.55	1.74e-04	1.52e-03
DDEFL1	55616	development and differentiation enhancing factor-l	7.94	7.15	7.79e-03	3.15e-02
DDIT4	54541	DNA-damage-inducible transcript 4	11.13	9.46	1.43e-08	5.87e-07
DDIT4L	115265	DNA-damage-inducible transcript 4-like	7.82	6.86	1.13e-02	4.19e-02
DDR2	4921	discoidin domain receptor family, member 2	9.04	7.85	2.17e-03	1.17e-02
DDX1	1653	DEAD (Asp-Glu-Ala-Asp) box polypeptide 1	10.05	9.44	7.23e-03	2.96e-02
DDX10	1662	DEAD (Asp-Glu-Ala-Asp) box polypeptide 10	7.96	7.27	2.53e-03	1.33e-02
DDX24	57062	DEAD (Asp-Glu-Ala-Asp) box polypeptide 24	9.22	8.62	8.82e-03	3.46e-02
DDX28	55794	DEAD (Asp-Glu-Ala-Asp) box polypeptide 28	5.44	7.03	2.96e-06	5.25e-05
DDX39	10212	DEAD (Asp-Glu-Ala-Asp) box polypeptide 39	9.00	9.91	1.35e-03	7.98e-03
DEDD2	162989	death effector domain containing 2	6.48	7.30	1.40e-02	4.96e-02
DEF6	50619	differentially expressed in FDCP 6 homolog (mouse)	5.87	8.74	4.41e-10	3.11e-08
DELGEF	26297	deafness locus associated putative guanine nucleot	8.38	7.38	1.40e-05	1.91e-04
DENND1A	57706	DENN/MADD domain containing 1A	5.73	6.93	9.92e-03	3.80e-02
DENND1C	79958	DENN/MADD domain containing 1C	6.37	9.01	4.04e-05	4.58e-04
DENND2D	79961	DENN/MADD domain containing 2D	7.03	8.86	2.79e-04	2.24e-03
DENND4B	9909	DENN/MADD domain containing 4B	7.41	8.59	2.44e-04	2.01e-03
DERL1	79139	Der1-like domain family, member 1	6.34	7.43	4.26e-04	3.16e-03
DEXI	28955	dexamethasone-induced transcript	7.83	6.98	2.04e-04	1.73e-03
DGAT2	84649	diacylglycerol O-acyltransferase homolog 2 (mouse)	5.17	7.55	1.16e-02	4.28e-02
DGKZ	8525	diacylglycerol kinase, zeta 104kDa	6.34	7.48	3.26e-03	1.61e-02
DHRS10	51171	dehydrogenase/reductase (SDR family) member 10	5.91	6.89	6.20e-03	2.63e-02
DHRS9	10170	dehydrogenase/reductase (SDR family) member 9	5.98	9.03	1.13e-03	6.99e-03
DHX29	54505	DEAH (Asp-Glu-Ala-His) box polypeptide 29	7.94	7.27	3.28e-03	1.62e-02
DIAPH2	1730	diaphanous homolog 2 (Drosophila)	9.46	7.85	6.28e-08	2.04e-06
DIP	23151	death-inducing-protein	6.09	7.73	7.59e-04	5.09e-03
DIP2C	22982	DIP2 disco-interacting protein 2 homolog C (Drosop	7.87	6.51	6.55e-06	1.01e-04
DIRAS3	9077	DIRAS family, GTP-binding RAS-like 3	6.12	4.54	1.43e-05	1.94e-04
DISC1	27185	disrupted in schizophrenia 1	6.22	7.04	7.56e-03	3.07e-02
DIXDC1	85458	DIX domain containing 1	9.10	7.40	7.72e-11	7.07e-09
DKFZp434N035	84222	hypothetical protein DKFZp434N035	4.80	6.10	1.10e-04	1.06e-03
DKFZp451A211	400169	DKFZp451A211 protein	6.72	5.37	1.63e-03	9.27e-03
DKFZP564B147	26071	DKFZP564B147 protein	7.25	6.12	5.99e-03	2.56e-02
DKFZp564J157	54458	DKFZp564J157 protein	11.34	12.13	4.35e-03	2.03e-02
DKFZP566N034	81615	hypothetical protein DKFZp566N034	4.74	7.18	3.05e-07	7.67e-06
DKFZp586I1420	222161	hypothetical protein DKFZp586I1420	6.27	7.56	1.96e-04	1.68e-03
DKFZP686A01247	22998	hypothetical protein	8.11	5.77	1.13e-14	2.78e-12
DKFZp686L1814	132660	hypothetical protein DKFZp686L1814	4.72	5.74	6.75e-03	2.81e-02
DKFZp761E198	91056	DKFZp761E198 protein	3.89	5.33	1.69e-03	9.58e-03
DKFZp761L1417	222865	hypothetical protein DKFZp761L1417	8.51	6.27	5.01e-06	8.04e-05
DKFZp761P0423	157285	hypothetical protein DKFZp761P0423	6.89	8.49	9.71e-04	6.19e-03
DKK3	27122	dickkopf homolog 3 (Xenopus laevis)	11.45	9.09	4.51e-06	7.35e-05
DLC1	10395	deleted in liver cancer 1	7.01	5.78	4.25e-07	1.01e-05
DLG5	9231	discs, large homolog 5 (Drosophila)	9.10	7.55	1.89e-06	3.58e-05
DMD	1756	dystrophin (muscular dystrophy, Duchenne and Becke	6.78	4.80	3.18e-05	3.74e-04
DMN	23336	desmuslin	10.92	8.44	2.01e-08	7.95e-07
DMPK	1760	dystrophia myotonica-protein kinase	9.14	7.19	1.31e-07	3.81e-06
DNAJB4	11080	DnaJ (Hsp40) homolog, subfamily B, member 4	8.74	7.46	1.21e-02	4.41e-02
DNAJB5	25822	DnaJ (Hsp40) homolog, subfamily B, member 5	8.17	6.99	5.21e-04	3.73e-03
DNAJB6	10049	DnaJ (Hsp40) homolog, subfamily B, member 6	9.66	8.85	3.55e-04	2.71e-03
DNALI1	7802	dynein, axonemal, light intermediate polypeptide 1	7.32	6.16	4.68e-03	2.14e-02
DNAPTP6	26010	DNA polymerase-transactivated protein 6	10.24	9.57	3.30e-03	1.63e-02
DNER	92737	delta-notch-like EGF repeat-containing transmembra	6.79	4.43	1.58e-09	9.02e-08
DNM2	1785	dynamin 2	7.47	8.36	4.88e-03	2.20e-02
DNMT1	1786	DNA (cytosine-5-)-methyltransferase 1	8.45	9.18	7.92e-03	3.19e-02
DOC1	11259	downregulated in ovarian cancer 1	10.63	8.60	3.07e-05	3.64e-04
DOCK1	1793	dedicator of cytokinesis 1	7.10	6.18	5.27e-03	2.33e-02
DOCK10	55619	dedicator of cytokinesis 10	6.53	7.59	3.25e-04	2.54e-03
DOCK2	1794	dedicator of cytokinesis 2	7.84	9.96	3.23e-08	1.17e-06
DOCK8	81704	dedicator of cytokinesis 8	6.41	8.99	5.99e-07	1.36e-05
DOK1	1796	docking protein 1, 62kDa (downstream of tyrosine k	8.05	8.95	2.56e-03	1.34e-02
DOK2	9046	docking protein 2, 56kDa	4.96	6.07	4.73e-04	3.45e-03
DOK4	55715	docking protein 4	7.63	6.34	5.90e-03	2.54e-02

Symbol	GeneID	Gene Name*	Signal CNTRL	AAA	P	FDR
DOK5	55816	docking protein 5	6.80	5.78	7.16e-03	2.95e-02
DOPEY2	9980	dopey family member 2	4.73	6.48	9.36e-04	6.02e-03
DPEP2	64174	dipeptidase 2	5.66	8.14	5.72e-05	6.15e-04
DPH2	1802	DPH2 homolog (S. cerevisiae)	7.72	8.53	3.44e-03	1.68e-02
DPP7	29952	dipeptidyl-peptidase 7	8.36	9.21	4.69e-03	2.14e-02
DPP9	91039	dipeptidyl-peptidase 9	6.81	7.58	1.28e-02	4.61e-02
DPY19L2	283417	dpy-19-like 2 (C. elegans)	5.81	3.88	1.23e-02	4.48e-02
DPY19L4	286148	dpy-19-like 4 (C. elegans)	7.97	7.39	1.19e-02	4.37e-02
DPYSL3	1809	dihydropyrimidinase-like 3	11.84	10.44	2.99e-06	5.27e-05
DPYSL4	10570	dihydropyrimidinase-like 4	5.15	6.15	8.62e-03	3.40e-02
DR1	1810	down-regulator of transcription 1, TBP-binding (ne	7.10	8.03	1.51e-03	8.72e-03
DSC2	1824	desmocollin 2	4.76	6.62	2.11e-03	1.15e-02
DSCR1L1	10231	Down syndrome critical region gene 1-like 1	11.67	8.64	3.96e-14	7.74e-12
DSTN	11034	destrin (actin depolymerizing factor)	13.70	11.23	5.24e-18	3.56e-15
DTNA	1837	dystrobrevin, alpha	6.78	4.95	9.45e-04	6.06e-03
DTNB	1838	dystrobrevin, beta	7.22	7.89	1.26e-02	4.57e-02
DTWD1	56986	DTW domain containing 1	8.03	6.92	1.91e-06	3.60e-05
DTX2	113878	deltex homolog 2 (Drosophila)	7.86	8.85	6.10e-04	4.26e-03
DTX3	196403	deltex 3 homolog (Drosophila)	8.91	7.49	1.60e-04	1.44e-03
DUS1L	64118	dihydrouridine synthase 1-like (S. cerevisiae)	9.23	9.91	1.17e-02	4.29e-02
DUS2L	54920	dihydrouridine synthase 2-like, SMM1 homolog (S. c	6.83	7.93	2.20e-04	1.85e-03
DUS3L	56931	dihydrouridine synthase 3-like (S. cerevisiae)	6.85	7.93	2.75e-04	2.22e-03
DUSP10	11221	dual specificity phosphatase 10	6.09	7.90	2.32e-03	1.24e-02
DUSP14	11072	dual specificity phosphatase 14	7.64	8.59	8.04e-03	3.23e-02
DUSP15	128853	dual specificity phosphatase 15	5.84	3.94	3.40e-04	2.63e-03
DUSP18	150290	dual specificity phosphatase 18	5.49	6.58	2.49e-03	1.31e-02
DUSP2	1844	dual specificity phosphatase 2	6.34	8.33	4.62e-03	2.11e-02
DUSP26	78986	dual specificity phosphatase 26 (putative)	9.03	5.98	3.83e-21	6.47e-18
DUSP27	92235	dual specificity phosphatase 27 (putative)	5.67	4.57	3.81e-04	2.87e-03
DUSP4	1846	dual specificity phosphatase 4	5.84	8.25	2.28e-03	1.22e-02
DUSP5	1847	dual specificity phosphatase 5	8.96	10.08	7.75e-03	3.14e-02
DUSP6	1848	dual specificity phosphatase 6	7.56	9.05	1.06e-03	6.61e-03
DUT	1854	dUTP pyrophosphatase	7.11	6.54	1.37e-02	4.87e-02
DYNC1I1	1780	dynein, cytoplasmic 1, intermediate chain 1	7.69	4.82	6.65e-08	2.14e-06
DYNC1I2	1781	dynein, cytoplasmic 1, intermediate chain 2	9.81	9.07	9.45e-04	6.06e-03
DYNC1LI2	1783	dynein, cytoplasmic 1, light intermediate chain 2	8.93	8.09	2.10e-04	1.78e-03
DYNC2LI1	51626	dynein, cytoplasmic 2, light intermediate chain 1	6.95	6.03	1.10e-04	1.06e-03
DYNLT3	6990	dynein, light chain, Tctex-type 3	10.12	9.06	3.47e-06	5.93e-05
DZIP1	22873	DAZ interacting protein 1	6.37	5.44	7.22e-04	4.90e-03
EAF2	55840	ELL associated factor 2	4.14	5.44	4.07e-04	3.03e-03
EBF	1879	early B-cell factor	9.18	7.11	1.13e-08	4.79e-07
EBI2	1880	Epstein-Barr virus induced gene 2 (lymphocyte-spec	8.69	10.83	2.27e-07	5.98e-06
ECEL1	9427	endothelin converting enzyme-like 1	7.28	6.06	1.03e-02	3.90e-02
ECGF1	1890	endothelial cell growth factor 1 (platelet-derived	10.47	12.97	4.78e-09	2.27e-07
ECHDC2	55268	enoyl Coenzyme A hydratase domain containing 2	9.57	8.60	3.40e-04	2.63e-03
ECM2	1842	extracellular matrix protein 2, female organ and a	8.63	6.70	1.28e-09	7.75e-08
ECRG4	84417	esophageal cancer related gene 4 protein	10.05	8.24	3.60e-08	1.29e-06
EDEM1	9695	ER degradation enhancer, mannosidase alpha-like 1	7.19	8.51	2.64e-05	3.23e-04
EDG2	1902	endothelial differentiation, lysophosphatidic acid	9.40	8.27	7.70e-05	7.90e-04
EDG6	8698	endothelial differentiation, lysophosphatidic acid	4.37	8.84	1.48e-06	2.91e-05
EDIL3	10085	EGF-like repeats and discoidin I-like domains 3	7.40	5.01	4.05e-07	9.66e-06
EDN2	1907	endothelin 2	5.92	3.82	7.48e-04	5.04e-03
EDNRA	1909	endothelin receptor type A	8.37	6.47	2.76e-08	1.03e-06
EEA1	8411	early endosome antigen 1, 162kD	5.84	4.86	1.07e-02	4.00e-02
EFEMP1	2202	EGF-containing fibulin-like extracellular matrix p	12.52	10.36	1.57e-06	3.08e-05
EFEMP2	30008	EGF-containing fibulin-like extracellular matrix p	9.95	8.92	2.72e-04	2.20e-03
EFHD1	80303	EF-hand domain family, member D1	11.59	8.26	2.20e-20	3.18e-17
EFHD2	79180	EF-hand domain family, member D2	8.62	10.62	7.45e-08	2.37e-06
EFNA1	1942	ephrin-A1	7.93	6.90	1.56e-05	2.08e-04
EFS	10278	embryonal Fyn-associated substrate	7.26	6.04	3.37e-04	2.62e-03
EGFR	1956	epidermal growth factor receptor (erythroblastic l	6.47	4.68	4.78e-04	3.48e-03
EGR2	1959	early growth response 2 (Krox-20 homolog, Drosophi	6.19	9.18	1.05e-05	1.49e-04
EHD1	10938	EH-domain containing 1	8.77	10.11	6.74e-05	7.03e-04
EHD2	30846	EH-domain containing 2	9.81	8.32	2.97e-06	5.25e-05
EHD3	30845	EH-domain containing 3	5.84	4.28	1.57e-03	8.99e-03
EI24	9538	etoposide induced 2.4 mRNA	9.66	8.83	2.49e-04	2.05e-03
EIF2B2	8892	eukaryotic translation initiation factor 2B, subun	8.81	7.91	8.22e-05	8.27e-04

Symbol	GeneID	Gene Name*	Signal CNTRL	AAA	P	FDR
EIF3S6	3646	eukaryotic translation initiation factor 3, subuni	11.63	11.03	1.11e-02	4.12e-02
EIF4A2	1974	eukaryotic translation initiation factor 4A, isofo	12.03	10.96	3.45e-06	5.92e-05
EIF4ENIF1	56478	eukaryotic translation initiation factor 4E nuclea	7.17	6.60	1.30e-02	4.67e-02
EIF5	1983	eukaryotic translation initiation factor 5	4.78	6.92	4.37e-08	1.49e-06
ELF1	1997	E74-like factor 1 (ets domain transcription factor	9.65	10.44	6.19e-03	2.63e-02
ELF4	2000	E74-like factor 4 (ets domain transcription factor	6.79	7.99	2.29e-03	1.23e-02
Ells1	222166	hypothetical protein Ells1	9.40	8.73	4.18e-03	1.96e-02
ELOVL4	6785	elongation of very long chain fatty acids (FEN1/El	7.56	5.82	1.83e-09	1.03e-07
ELOVL7	79993	ELOVL family member 7, elongation of long chain fa	7.43	6.72	9.85e-03	3.78e-02
EMB	133418	embigin homolog (mouse)	5.82	6.97	4.44e-03	2.06e-02
EMCN	51705	endomucin	7.67	6.57	1.20e-03	7.28e-03
EMILIN1	11117	elastin microfibril interfacer 1	10.63	9.32	5.56e-05	6.02e-04
EMILIN2	84034	elastin microfibril interfacer 2	8.75	10.38	6.43e-03	2.71e-02
EML1	2009	echinoderm microtubule associated protein like 1	8.47	6.46	2.36e-08	9.05e-07
EML2	24139	echinoderm microtubule associated protein like 2	9.15	8.51	7.18e-03	2.95e-02
EML4	27436	echinoderm microtubule associated protein like 4	8.74	9.50	5.73e-03	2.48e-02
EMP2	2013	epithelial membrane protein 2	9.78	8.45	1.17e-04	1.11e-03
EMP3	2014	epithelial membrane protein 3	9.31	10.53	1.64e-03	9.30e-03
EMR1	2015	egf-like module containing, mucin-like, hormone re	4.90	6.51	2.11e-03	1.15e-02
EMR2	30817	egf-like module containing, mucin-like, hormone re	5.15	8.17	1.45e-04	1.33e-03
EMR3	84658	egf-like module containing, mucin-like, hormone re	5.09	6.86	5.36e-03	2.36e-02
ENAH	55740	enabled homolog (Drosophila)	6.65	5.58	4.97e-04	3.59e-03
ENC1	8507	ectodermal-neural cortex (with BTB-like domain)	6.21	7.57	2.15e-04	1.81e-03
ENDOD1	23052	endonuclease domain containing 1	10.17	8.50	5.47e-09	2.59e-07
ENPP4	22875	ectonucleotide pyrophosphatase/phosphodiesterase 4	8.88	7.75	9.40e-07	1.99e-05
ENPP5	59084	ectonucleotide pyrophosphatase/phosphodiesterase 5	5.87	3.94	6.20e-08	2.03e-06
ENTPD1	953	ectonucleoside triphosphate diphosphohydrolase 1	7.13	8.00	4.39e-03	2.04e-02
EPB41L3	23136	erythrocyte membrane protein band 4.1-like 3	10.17	11.00	1.23e-02	4.48e-02
EPB49	2039	erythrocyte membrane protein band 4.9 (dematin)	6.88	6.03	8.88e-04	5.78e-03
EPC2	26122	enhancer of polycomb homolog 2 (Drosophila)	8.77	7.72	1.50e-03	8.67e-03
EPDR1	54749	ependymin related protein 1 (zebrafish)	10.54	9.10	5.28e-08	1.76e-06
EPHA3	2042	EPH receptor A3	7.44	6.44	7.03e-03	2.90e-02
EPHB2	2048	EPH receptor B2	6.45	7.79	8.33e-04	5.50e-03
EPM2A	7957	epilepsy, progressive myoclonus type 2A, Lafora di	7.45	6.60	2.32e-04	1.94e-03
EPM2AIP1	9852	EPM2A (laforin) interacting protein 1	8.47	7.58	1.43e-04	1.31e-03
EPN2	22905	epsin 2	7.61	6.71	4.68e-03	2.14e-02
EPS8	2059	epidermal growth factor receptor pathway substrate	10.23	8.05	1.19e-07	3.53e-06
EPS8L2	64787	EPS8-like 2	7.30	6.01	2.31e-05	2.89e-04
EPSTI1	94240	epithelial stromal interaction 1 (breast)	7.76	9.71	1.10e-06	2.27e-05
ERRFI1	54206	ERBB receptor feedback inhibitor 1	11.46	10.15	5.32e-04	3.79e-03
ESAM	90952	endothelial cell adhesion molecule	11.01	9.79	4.71e-04	3.45e-03
ESD	2098	esterase D/formylglutathione hydrolase	11.02	10.00	8.82e-06	1.29e-04
ESR1	2099	estrogen receptor 1	7.45	6.49	1.25e-02	4.53e-02
ESRRA	2101	estrogen-related receptor alpha	5.33	6.16	8.71e-03	3.43e-02
ETNK2	55224	ethanolamine kinase 2	7.22	5.38	1.14e-08	4.80e-07
EVA1	10205	epithelial V-like antigen 1	10.03	8.33	5.43e-05	5.90e-04
EVC	2121	Ellis van Creveld syndrome	8.72	7.56	7.82e-03	3.16e-02
EVI1	2122	ecotropic viral integration site 1	8.97	7.36	1.30e-07	3.79e-06
EVI2A	2123	ecotropic viral integration site 2A	6.14	7.78	1.65e-03	9.37e-03
EVI2B	2124	ecotropic viral integration site 2B	8.40	10.46	1.06e-07	3.20e-06
EVL	51466	Enah/Vasp-like	7.39	8.73	5.63e-04	3.98e-03
EXDL2	55218	exonuclease 3'-5' domain-like 2	7.39	6.70	2.51e-03	1.32e-02
EXOSC4	54512	exosome component 4	7.69	8.61	1.34e-03	7.91e-03
EXOSC6	118460	exosome component 6	7.92	8.70	4.44e-03	2.06e-02
EXT1	2131	exostoses (multiple) 1	9.92	8.58	2.66e-06	4.79e-05
EXT2	2132	exostoses (multiple) 2	8.89	8.05	3.38e-04	2.63e-03
EXTL2	2135	exostoses (multiple)-like 2	7.44	6.48	3.58e-05	4.12e-04
EYA2	2139	eyes absent homolog 2 (Drosophila)	6.97	6.18	2.72e-03	1.40e-02
EZH2	2146	enhancer of zeste homolog 2 (Drosophila)	4.19	5.36	1.26e-03	7.56e-03
F11R	50848	F11 receptor	6.58	8.12	1.21e-05	1.71e-04
F2R	2149	coagulation factor II (thrombin) receptor	8.69	7.69	7.66e-03	3.11e-02
FADS1	3992	fatty acid desaturase 1	8.40	7.68	1.70e-03	9.58e-03
FADS2	9415	fatty acid desaturase 2	8.36	7.49	1.87e-04	1.61e-03
FAIM3	9214	Fas apoptotic inhibitory molecule 3	5.03	9.21	8.46e-03	3.36e-02
FAM100B	283991	family with sequence similarity 100, member B	7.94	8.84	1.73e-03	9.74e-03
FAM101A	144347	family with sequence similarity 101, member A	5.65	6.78	3.62e-03	1.76e-02
FAM101B	359845	family with sequence similarity 101, member B	6.93	8.95	4.21e-03	1.97e-02

Symbol	GeneID	Gene Name*	Signal CNTRL	AAA	P	FDR
FAM105A	54491	family with sequence similarity 105, member A	5.52	7.83	3.59e-07	8.77e-06
FAM107B	83641	family with sequence similarity 107, member B	8.01	9.84	1.56e-05	2.08e-04
FAM112B	121355	family with sequence similarity 112, member B	4.81	6.55	7.49e-03	3.05e-02
FAM113B	91523	family with sequence similarity 113, member B	7.12	8.23	3.12e-03	1.56e-02
FAM13A1	10144	family with sequence similarity 13, member A1	8.10	7.49	8.48e-03	3.36e-02
FAM19A2	338811	family with sequence similarity 19 (chemokine (C-C	6.39	4.23	4.88e-04	3.54e-03
FAM19A5	25817	family with sequence similarity 19 (chemokine (C-C	7.96	6.46	1.65e-05	2.18e-04
FAM20A	54757	family with sequence similarity 20, member A	5.91	7.18	1.47e-03	8.55e-03
FAM20B	9917	family with sequence similarity 20, member B	9.91	9.10	3.54e-04	2.70e-03
FAM44B	91272	family with sequence similarity 44, member B	8.71	7.76	3.45e-05	3.99e-04
FAM46B	115572	family with sequence similarity 46, member B	8.83	6.00	7.81e-18	4.74e-15
FAM46C	54855	family with sequence similarity 46, member C	4.87	6.85	7.83e-03	3.16e-02
FAM49A	81553	family with sequence similarity 49, member A	6.70	7.56	5.22e-03	2.31e-02
FAM49B	51571	family with sequence similarity 49, member B	8.67	10.63	8.00e-04	5.32e-03
FAM50B	26240	family with sequence similarity 50, member B	7.97	6.65	3.84e-08	1.34e-06
FAM53B	9679	family with sequence similarity 53, member B	6.23	8.29	5.16e-08	1.73e-06
FAM59A	64762	family with sequence similarity 59, member A	7.44	5.44	6.73e-04	4.63e-03
FAM62B	57488	family with sequence similarity 62 (C2 domain cont	9.67	8.68	2.07e-05	2.64e-04
FAM63B	54629	family with sequence similarity 63, member B	7.64	6.65	1.08e-02	4.03e-02
FAM78A	286336	family with sequence similarity 78, member A	5.89	6.81	7.43e-03	3.03e-02
FAM8A1	51439	family with sequence similarity 8, member A1	10.20	9.12	3.40e-06	5.83e-05
FAM96A	84191	family with sequence similarity 96, member A	7.88	9.16	1.70e-04	1.50e-03
FANCC	2176	Fanconi anemia, complementation group C	5.82	6.82	9.32e-04	6.00e-03
FANCE	2178	Fanconi anemia, complementation group E	7.40	6.29	1.14e-04	1.08e-03
FANCG	2189	Fanconi anemia, complementation group G	5.52	6.78	9.23e-05	9.08e-04
FAT	2195	FAT tumor suppressor homolog 1 (Drosophila)	9.82	8.42	8.33e-04	5.50e-03
FAT4	79633	FAT tumor suppressor homolog 4 (Drosophila)	7.93	6.43	4.27e-05	4.80e-04
FBLIM1	54751	filamin binding LIM protein 1	9.87	7.44	2.38e-16	1.06e-13
FBLN5	10516	fibulin 5	11.52	8.60	1.99e-18	1.90e-15
FBN1	2200	fibrillin 1 (Marfan syndrome)	10.92	9.96	5.10e-03	2.27e-02
FBP1	2203	fructose-1,6-bisphosphatase 1	7.99	11.24	6.57e-03	2.75e-02
FBS1	64319	fibrosin 1	5.89	6.91	7.82e-03	3.16e-02
FBXL11	22992	F-box and leucine-rich repeat protein 11	6.80	7.73	1.18e-03	7.20e-03
FBXL14	144699	F-box and leucine-rich repeat protein 14	6.82	5.56	1.25e-04	1.16e-03
FBXL17	64839	F-box and leucine-rich repeat protein 17	7.74	6.73	3.53e-03	1.72e-02
FBXL2	25827	F-box and leucine-rich repeat protein 2	6.84	4.76	9.68e-13	1.43e-10
FBXL3	26224	F-box and leucine-rich repeat protein 3	9.19	8.16	6.55e-04	4.52e-03
FBXL7	23194	F-box and leucine-rich repeat protein 7	7.75	6.60	8.13e-05	8.19e-04
FBXO17	115290	F-box protein 17	7.17	5.90	1.71e-06	3.30e-05
FBXO18	84893	F-box protein, helicase, 18	9.39	8.83	1.37e-02	4.86e-02
FBXO21	23014	F-box protein 21	9.40	8.58	3.04e-04	2.40e-03
FBXO22	26263	F-box protein 22	7.21	6.40	4.21e-03	1.97e-02
FBXO27	126433	F-box protein 27	6.36	5.07	2.08e-04	1.76e-03
FBXO3	26273	F-box protein 3	7.33	6.53	8.68e-04	5.69e-03
FBXO31	79791	F-box protein 31	7.71	6.97	4.47e-03	2.07e-02
FBXO32	114907	F-box protein 32	10.50	8.07	5.91e-10	4.03e-08
FBXO8	26269	F-box protein 8	8.78	8.03	9.29e-04	5.99e-03
FBXW4	6468	F-box and WD-40 domain protein 4	8.28	7.62	3.95e-03	1.88e-02
FCER1A	2205	Fc fragment of IgE, high affinity I, receptor for;	4.95	7.75	6.18e-06	9.59e-05
FCER1G	2207	Fc fragment of IgE, high affinity I, receptor for;	9.76	11.25	2.41e-04	1.99e-03
FCGR2A	2212	Fc fragment of IgG, low affinity IIa, receptor (CD	8.84	10.02	5.20e-04	3.72e-03
FCGR2B	2213	Fc fragment of IgG, low affinity IIb, receptor (CD	7.77	9.72	6.24e-04	4.34e-03
FCGR3B	2215	Fc fragment of IgG, low affinity IIIb, receptor (C	7.02	8.86	3.75e-03	1.81e-02
FCGRT	2217	Fc fragment of IgG, receptor, transporter, alpha	10.45	11.81	2.68e-05	3.28e-04
FCHO1	23149	FCH domain only 1	5.50	7.46	3.52e-07	8.61e-06
FCN1	2219	ficolin (collagen/fibrinogen domain containing) 1	7.59	10.61	2.77e-05	3.37e-04
FCRLM1	84824	Fc receptor-like and mucin-like 1	5.38	9.36	8.58e-03	3.39e-02
FCRLM2	127943	Fc receptor-like and mucin-like 2	6.49	7.90	2.71e-03	1.40e-02
FEM1A	55527	fem-1 homolog a (C.elegans)	7.86	8.64	4.59e-03	2.11e-02
FEN1	2237	flap structure-specific endonuclease 1	5.54	7.14	2.73e-06	4.88e-05
FER	2241	fer (fps/fes related) tyrosine kinase (phosphoprot	6.58	5.10	3.18e-06	5.57e-05
FEZ1	9638	fasciculation and elongation protein zeta 1 (zygin	6.59	4.44	3.90e-10	2.83e-08
FGD3	89846	FYVE, RhoGEF and PH domain containing 3	5.81	8.30	1.63e-05	2.16e-04
FGF13	2258	fibroblast growth factor 13	9.18	6.83	3.73e-07	9.08e-06
FGF2	2247	fibroblast growth factor 2 (basic)	6.04	4.73	2.56e-04	2.09e-03
FGFRL1	53834	fibroblast growth factor receptor-like 1	8.19	6.52	1.76e-10	1.46e-08
FGL2	10875	fibrinogen-like 2	9.69	8.95	1.15e-03	7.06e-03

Symbol	GeneID	Gene Name*	Signal CNTRL	Signal AAA	P	FDR
FGR	2268	Gardner-Rasheed feline sarcoma viral (v-fgr) oncog	7.00	9.18	1.63e-05	2.16e-04
FHL1	2273	four and a half LIM domains 1	12.50	9.72	4.26e-16	1.80e-13
FHL5	9457	four and a half LIM domains 5	9.49	5.73	6.82e-07	1.53e-05
FHOD3	80206	formin homology 2 domain containing 3	6.55	4.68	1.45e-04	1.32e-03
FIGF	2277	c-fos induced growth factor (vascular endothelial	7.26	5.19	1.31e-03	7.79e-03
FILIP1	27145	filamin A interacting protein 1	7.68	5.10	3.48e-14	7.05e-12
FKBP7	51661	FK506 binding protein 7	7.15	5.39	8.56e-11	7.72e-09
FKHL18	2307	forkhead-like 18 (Drosophila)	7.64	6.15	2.94e-05	3.53e-04
FKSG44	83786	FKSG44 gene	8.08	9.15	2.89e-04	2.30e-03
FLJ10159	55084	hypothetical protein FLJ10159	9.38	7.55	3.93e-07	9.44e-06
FLJ10781	55228	hypothetical protein FLJ10781	7.86	5.79	8.65e-09	3.78e-07
FLJ10925	55262	hypothetical protein FLJ10925	6.88	8.18	7.10e-05	7.36e-04
FLJ11200	55325	hypothetical protein FLJ11200	9.40	8.41	1.27e-05	1.77e-04
FLJ11712	79621	deleted in leukemia 8 protein	8.20	8.98	4.64e-03	2.12e-02
FLJ13149	60493	hypothetical protein FLJ13149	6.35	7.50	2.65e-04	2.15e-03
FLJ13195	64940	stromal antigen 3-like	6.07	5.08	1.50e-04	1.36e-03
FLJ13305	84140	hypothetical protein FLJ13305	5.92	5.06	1.28e-02	4.60e-02
FLJ13646	79635	hypothetical protein FLJ13646	5.95	4.99	4.78e-03	2.16e-02
FLJ14001	79730	hypothetical protein FLJ14001	6.15	4.93	3.24e-04	2.53e-03
FLJ14054	79614	hypothetical protein FLJ14054	6.26	4.33	1.35e-05	1.85e-04
FLJ14466	84876	hypothetical protein FLJ14466	5.72	6.91	1.05e-03	6.59e-03
FLJ14959	284391	hypothetical protein FLJ14959	7.45	6.38	9.91e-05	9.65e-04
FLJ20054	54530	hypothetical protein FLJ20054	6.63	8.16	3.41e-04	2.63e-03
FLJ20245	54863	hypothetical protein FLJ20245	5.88	7.13	4.36e-04	3.22e-03
FLJ20254	54867	hypothetical protein FLJ20254	8.91	7.54	1.03e-07	3.14e-06
FLJ20273	54502	RNA-binding protein	6.74	8.91	1.61e-04	1.45e-03
FLJ20323	54468	hypothetical protein FLJ20323	7.40	8.12	7.85e-03	3.17e-02
FLJ20366	55638	hypothetical protein FLJ20366	8.20	7.14	1.83e-03	1.02e-02
FLJ20534	54969	hypothetical protein FLJ20534	8.30	7.73	1.20e-02	4.38e-02
FLJ20647	55013	hypothetical protein FLJ20647	8.10	9.76	1.46e-04	1.33e-03
FLJ20701	55022	hypothetical protein FLJ20701	10.95	8.75	2.94e-10	2.25e-08
FLJ20718	55027	hypothetical protein FLJ20718	6.30	7.41	1.41e-03	8.26e-03
FLJ20920	80221	hypothetical protein FLJ20920	6.31	7.25	1.16e-03	7.14e-03
FLJ21062	79846	hypothetical protein FLJ21062	7.66	6.30	2.95e-05	3.53e-04
FLJ21159	79884	ASAP	6.42	4.96	4.26e-03	2.00e-02
FLJ21438	64926	hypothetical protein FLJ21438	7.05	10.24	1.84e-04	1.59e-03
FLJ21742	84167	hypothetical protein FLJ21742	5.99	5.25	4.84e-03	2.18e-02
FLJ21963	79611	FLJ21963 protein	7.23	5.67	1.10e-08	4.67e-07
FLJ21986	79974	hypothetical protein FLJ21986	11.16	7.99	4.71e-09	2.24e-07
FLJ22104	65084	hypothetical protein FLJ22104	8.77	7.81	3.75e-04	2.83e-03
FLJ22471	80212	limkain beta 2	8.74	8.07	2.89e-03	1.47e-02
FLJ22531	79703	hypothetical protein FLJ22531	4.73	6.02	1.32e-02	4.72e-02
FLJ22624	79866	FLJ22624 protein	4.80	5.99	8.78e-03	3.45e-02
FLJ22655	79785	hypothetical protein FLJ22655	9.19	7.32	9.08e-03	3.53e-02
FLJ22662	79887	hypothetical protein FLJ22662	8.93	9.99	5.23e-03	2.32e-02
FLJ25076	134111	similar to CG4502-PA	6.91	5.61	1.68e-05	2.21e-04
FLJ30596	133686	hypothetical protein FLJ30596	6.71	5.84	2.89e-04	2.30e-03
FLJ31033	91351	hypothetical protein FLJ31033	6.04	7.58	7.84e-04	5.23e-03
FLJ31413	155061	hypothetical protein FLJ31413	6.84	7.65	3.78e-03	1.82e-02
FLJ32028	201799	hypothetical protein FLJ32028	6.14	8.33	6.26e-04	4.35e-03
FLJ33641	202309	hypothetical protein FLJ33641	5.60	8.37	1.65e-07	4.61e-06
FLJ36031	168455	hypothetical protein FLJ36031	7.47	8.38	4.39e-03	2.04e-02
FLJ37798	401264	hypothetical gene supported by AK095117	7.08	6.32	6.28e-03	2.66e-02
FLJ38359	151009	hypothetical protein FLJ38359	7.83	5.03	2.36e-04	1.97e-03
FLJ39051	399972	hypothetical gene supported by AK096370	9.97	11.11	2.72e-03	1.40e-02
FLJ39378	353116	hypothetical protein FLJ39378	6.63	4.83	1.98e-07	5.34e-06
FLJ40432	151195	hypothetical protein FLJ40432	5.65	6.65	3.38e-03	1.66e-02
FLJ40542	200298	hypothetical protein FLJ40542	5.50	6.27	8.80e-03	3.45e-02
FLJ41603	389337	FLJ41603 protein	6.65	5.70	2.40e-04	1.99e-03
FLJ42351	400999	hypothetical gene supported by AK124342	6.04	4.68	6.95e-04	4.75e-03
FLJ90013	202018	hypothetical protein FLJ90013	7.86	6.89	6.37e-04	4.42e-03
FLJ90166	164284	hypothetical protein FLJ90166	7.22	5.51	2.32e-03	1.24e-02
FLJ90723	285580	hypothetical protein FLJ90723	6.20	5.13	2.13e-03	1.16e-02
FLNA	2316	filamin A, alpha (actin binding protein 280)	13.87	12.47	3.73e-09	1.83e-07
FLNC	2318	filamin C, gamma (actin binding protein 280)	7.78	5.36	4.95e-06	7.96e-05
FMNL1	752	formin-like 1	5.17	6.83	3.18e-04	2.49e-03
FMO2	2327	flavin containing monooxygenase 2	9.72	7.03	1.03e-09	6.40e-08

Symbol	GeneID	Gene Name*	Signal CNTRL	AAA	P	FDR
FMO3	2328	flavin containing monooxygenase 3	7.10	5.81	6.53e-03	2.74e-02
FMO4	2329	flavin containing monooxygenase 4	7.64	6.64	1.72e-05	2.25e-04
FMOD	2331	fibromodulin	10.93	8.99	2.29e-08	8.84e-07
FN3K	64122	fructosamine 3 kinase	7.90	6.92	1.24e-03	7.45e-03
FN5	56935	FN5 protein	8.17	8.95	1.05e-02	3.96e-02
FOLR2	2350	folate receptor 2 (fetal)	9.71	10.67	1.35e-02	4.81e-02
FOSB	2354	FBJ murine osteosarcoma viral oncogene homolog B	8.70	12.34	5.79e-06	9.08e-05
FOSL1	8061	FOS-like antigen 1	5.85	6.66	9.72e-03	3.73e-02
FOXC1	2296	forkhead box C1	10.21	8.17	5.72e-11	5.50e-09
FOXC2	2303	forkhead box C2 (MFH-1, mesenchyme forkhead 1)	6.68	5.45	7.34e-05	7.58e-04
FOXD1	2297	forkhead box D1	7.90	5.83	2.03e-11	2.19e-09
FOXJ2	55810	forkhead box J2	9.18	8.40	3.22e-03	1.60e-02
FOXM1	2305	forkhead box M1	4.93	6.21	4.48e-03	2.07e-02
FOXO1A	2308	forkhead box O1A (rhabdomyosarcoma)	10.00	9.28	9.88e-03	3.78e-02
FOXP1	27086	forkhead box P1	11.43	10.48	5.98e-04	4.19e-03
FPR1	2357	formyl peptide receptor 1	8.31	10.37	5.31e-03	2.34e-02
FPRL1	2358	formyl peptide receptor-like 1	4.77	6.35	6.24e-03	2.65e-02
FPRL2	2359	formyl peptide receptor-like 2	8.21	10.58	4.19e-04	3.11e-03
FRAT1	10023	frequently rearranged in advanced T-cell lymphomas	4.49	6.17	1.74e-05	2.26e-04
FRAT2	23401	frequently rearranged in advanced T-cell lymphomas	6.70	8.16	7.77e-06	1.15e-04
FREM1	158326	FRAS1 related extracellular matrix 1	6.49	5.26	7.89e-04	5.26e-03
FREQ	23413	frequenin homolog (Drosophila)	7.63	6.45	4.76e-05	5.26e-04
FRK	2444	fyn-related kinase	6.83	3.86	1.28e-05	1.78e-04
FRMD3	257019	FERM domain containing 3	6.79	5.69	4.70e-04	3.45e-03
FRMD6	122786	FERM domain containing 6	8.62	7.69	1.97e-03	1.09e-02
FRY	10129	furry homolog (Drosophila)	9.06	7.30	1.88e-11	2.09e-09
FRZB	2487	frizzled-related protein	12.04	8.68	4.52e-16	1.80e-13
FSCN1	6624	fascin homolog 1, actin-bundling protein (Strongyl	5.13	6.74	1.70e-04	1.50e-03
FSTL1	11167	follistatin-like 1	12.76	11.33	4.27e-07	1.01e-05
FTO	79068	fatso	8.69	7.94	1.66e-03	9.41e-03
FTS	64400	fused toes homolog (mouse)	9.38	8.27	1.44e-06	2.85e-05
FUCA1	2517	fucosidase, alpha-L- 1, tissue	9.77	11.78	9.94e-04	6.30e-03
FUT7	2529	fucosyltransferase 7 (alpha (1,3) fucosyltransfera	4.14	6.32	3.80e-04	2.87e-03
FVT1	2531	follicular lymphoma variant translocation 1	8.38	7.78	8.47e-03	3.36e-02
FXYD5	53827	FXYD domain containing ion transport regulator 5	9.70	10.60	2.86e-03	1.46e-02
FXYD6	53826	FXYD domain containing ion transport regulator 6	6.86	7.68	4.02e-03	1.91e-02
FYB	2533	FYN binding protein (FYB-120/130)	5.80	7.96	2.70e-06	4.86e-05
FYCO1	79443	FYVE and coiled-coil domain containing 1	8.96	7.30	1.77e-11	2.00e-09
FZD1	8321	frizzled homolog 1 (Drosophila)	9.54	8.01	4.44e-04	3.27e-03
FZD3	7976	frizzled homolog 3 (Drosophila)	7.11	5.90	1.98e-03	1.09e-02
FZD5	7855	frizzled homolog 5 (Drosophila)	4.88	5.91	3.11e-03	1.56e-02
FZD6	8323	frizzled homolog 6 (Drosophila)	7.86	6.47	1.05e-03	6.59e-03
G0S2	50486	G0/G1switch 2	9.45	11.05	2.43e-03	1.28e-02
G1P2	9636	interferon, alpha-inducible protein (clone IFI-15K	8.91	10.21	1.33e-03	7.88e-03
GAA	2548	glucosidase, alpha; acid (Pompe disease, glycogen	7.78	8.79	4.20e-03	1.97e-02
GAB3	139716	GRB2-associated binding protein 3	5.49	7.02	4.35e-04	3.21e-03
GABBR1	2550	gamma-aminobutyric acid (GABA) B receptor, 1	9.17	7.97	2.18e-07	5.82e-06
GABRR1	2569	gamma-aminobutyric acid (GABA) receptor, rho 1	6.55	5.06	4.90e-03	2.21e-02
GAD1	2571	glutamate decarboxylase 1 (brain, 67kDa)	6.41	4.16	3.50e-03	1.71e-02
GADD45A	1647	growth arrest and DNA-damage-inducible, alpha	11.52	9.27	1.87e-09	1.04e-07
GADD45B	4616	growth arrest and DNA-damage-inducible, beta	11.95	10.70	2.68e-07	6.93e-06
GADD45G	10912	growth arrest and DNA-damage-inducible, gamma	10.63	8.72	6.27e-03	2.66e-02
GAK	2580	cyclin G associated kinase	8.25	9.25	5.68e-04	4.01e-03
GALC	2581	galactosylceramidase	8.55	9.54	1.16e-03	7.15e-03
GALE	2582	UDP-galactose-4-epimerase	6.08	7.29	5.16e-03	2.29e-02
GALNAC4S-6ST	51363	B cell RAG associated protein	8.48	9.42	9.69e-04	6.18e-03
GALNT4	8693	UDP-N-acetyl-alpha-D-galactosamine:polypeptide N-a	5.94	6.74	5.45e-03	2.39e-02
GALNT6	11226	UDP-N-acetyl-alpha-D-galactosamine:polypeptide N-a	4.57	7.20	2.02e-04	1.72e-03
GALNTL4	374378	UDP-N-acetyl-alpha-D-galactosamine:polypeptide N-a	7.07	6.08	3.76e-03	1.81e-02
GARNL1	253959	GTPase activating Rap/RanGAP domain-like 1	8.88	8.22	8.72e-03	3.43e-02
GARNL3	84253	GTPase activating Rap/RanGAP domain-like 3	7.87	6.18	2.62e-07	6.79e-06
GARNL4	23108	GTPase activating Rap/RanGAP domain-like 4	4.47	5.54	3.82e-03	1.84e-02
GAS1	2619	growth arrest-specific 1	8.71	7.80	1.16e-02	4.27e-02
GAS6	2621	growth arrest-specific 6	11.68	10.36	1.78e-04	1.55e-03
GATA3	2625	GATA binding protein 3	4.59	6.58	5.16e-04	3.71e-03
GATM	2628	glycine amidinotransferase (L-arginine:glycine ami	6.76	8.15	8.05e-05	8.15e-04
GBE1	2632	glucan (1,4-alpha-), branching enzyme 1 (glycogen	9.20	8.45	6.64e-03	2.78e-02

Symbol	GeneID	Gene Name*	Signal CNTRL	AAA	P	FDR
GBGT1	26301	globoside alpha-1,3-N-acetylgalactosaminyltransfer	7.02	8.03	2.35e-03	1.25e-02
GBP2	2634	guanylate binding protein 2, interferon-inducible	7.13	6.00	9.32e-06	1.35e-04
GBP5	115362	guanylate binding protein 5	6.43	8.53	9.66e-08	2.97e-06
GCA	25801	grancalcin, EF-hand calcium binding protein	8.91	9.68	5.06e-03	2.26e-02
GCC1	79571	GRIP and coiled-coil domain containing 1	7.78	8.53	9.92e-03	3.80e-02
GCH1	2643	GTP cyclohydrolase 1 (dopa-responsive dystonia)	6.03	6.92	8.06e-03	3.23e-02
GCK	2645	glucokinase (hexokinase 4, maturity onset diabetes	5.52	4.50	4.05e-03	1.92e-02
GCLC	2729	glutamate-cysteine ligase, catalytic subunit	7.43	8.26	2.84e-03	1.45e-02
GCLM	2730	glutamate-cysteine ligase, modifier subunit	6.05	6.93	1.06e-02	3.98e-02
GCNT1	2650	glucosaminyl (N-acetyl) transferase 1, core 2 (bet	5.34	6.58	6.21e-04	4.32e-03
GDF10	2662	growth differentiation factor 10	8.20	6.27	3.34e-11	3.35e-09
GDF15	9518	growth differentiation factor 15	9.30	7.43	3.40e-04	2.63e-03
GEFT	115557	RAC/CDC42 exchange factor	8.41	6.96	5.69e-03	2.47e-02
GENX-3414	8987	genethonin 1	7.07	5.23	1.85e-11	2.07e-09
GFRA2	2675	GDNF family receptor alpha 2	6.10	7.82	1.34e-04	1.24e-03
GGA1	26088	golgi associated, gamma adaptin ear containing, AR	7.67	8.45	1.14e-02	4.22e-02
GGA2	23062	golgi associated, gamma adaptin ear containing, AR	7.56	9.40	2.19e-05	2.76e-04
GGT1	2678	gamma-glutamyltransferase 1	6.08	7.35	7.59e-05	7.82e-04
GGTLA1	2687	gamma-glutamyltransferase-like activity 1	7.82	6.93	7.82e-03	3.16e-02
GHR	2690	growth hormone receptor	8.52	6.65	9.60e-10	6.12e-08
GHRL	51738	ghrelin, growth hormone secretagogue receptor liga	3.99	6.33	1.31e-02	4.69e-02
GIMAP2	26157	GTPase, IMAP family member 2	6.48	7.29	3.77e-03	1.82e-02
GIYD2	79008	GIY-YIG domain containing 2	5.37	7.03	1.35e-04	1.25e-03
GJA1	2697	gap junction protein, alpha 1, 43kDa (connexin 43)	10.66	8.82	2.10e-05	2.67e-04
GJA4	2701	gap junction protein, alpha 4, 37kDa (connexin 37)	9.28	8.08	7.52e-03	3.06e-02
GK	2710	glycerol kinase	6.37	8.29	4.58e-03	2.10e-02
GKAP1	80318	G kinase anchoring protein 1	7.33	6.09	1.90e-03	1.05e-02
GLA	2717	galactosidase, alpha	8.12	9.86	1.40e-03	8.21e-03
GLB1	2720	galactosidase, beta 1	8.84	9.62	7.83e-03	3.16e-02
GLDN	342035	gliomedin	9.21	6.77	1.83e-07	5.06e-06
GLRB	2743	glycine receptor, beta	7.74	5.67	2.65e-14	5.57e-12
GLS	2744	glutaminase	9.95	8.51	2.78e-09	1.44e-07
GLT1D1	144423	glycosyltransferase 1 domain containing 1	4.96	8.25	1.28e-02	4.62e-02
GLT25D1	79709	glycosyltransferase 25 domain containing 1	8.07	9.27	1.29e-04	1.19e-03
GLT8D2	83468	glycosyltransferase 8 domain containing 2	7.75	6.15	7.80e-07	1.71e-05
GM2A	2760	GM2 ganglioside activator	6.83	8.99	3.40e-03	1.66e-02
GMDS	2762	GDP-mannose 4,6-dehydratase	9.91	7.96	3.68e-14	7.32e-12
GMEB2	26205	glucocorticoid modulatory element binding protein	6.53	7.42	2.56e-03	1.34e-02
GMFG	9535	glia maturation factor, gamma	7.75	9.80	6.93e-06	1.05e-04
GMIP	51291	GEM interacting protein	6.21	8.30	1.53e-07	4.36e-06
GMPPB	29925	GDP-mannose pyrophosphorylase B	6.53	7.39	3.34e-03	1.64e-02
GNA11	2767	guanine nucleotide binding protein (G protein), al	11.00	10.00	2.25e-04	1.88e-03
GNA13	10672	guanine nucleotide binding protein (G protein), al	9.28	10.22	5.10e-03	2.27e-02
GNA15	2769	guanine nucleotide binding protein (G protein), al	7.23	9.21	2.07e-03	1.13e-02
GNAI1	2770	guanine nucleotide binding protein (G protein), al	6.49	5.17	5.95e-06	9.29e-05
GNAZ	2781	guanine nucleotide binding protein (G protein), al	6.55	5.59	2.04e-03	1.12e-02
GNB5	10681	guanine nucleotide binding protein (G protein), be	7.75	6.97	3.33e-03	1.64e-02
GNG12	55970	guanine nucleotide binding protein (G protein), ga	7.78	6.77	1.26e-02	4.56e-02
GNLY	10578	granulysin	6.36	7.93	1.56e-03	8.98e-03
GNPDA1	10007	glucosamine-6-phosphate deaminase 1	8.28	9.21	9.87e-03	3.78e-02
GNPDA2	132789	glucosamine-6-phosphate deaminase 2	7.71	6.82	1.07e-04	1.03e-03
GNPTAB	79158	N-acetylglucosamine-1-phosphate transferase, alpha	7.06	8.14	3.17e-03	1.58e-02
GNRH1	2796	gonadotropin-releasing hormone 1 (luteinizing-rele	6.30	5.47	8.84e-04	5.76e-03
GOLPH3L	55204	golgi phosphoprotein 3-like	9.33	8.59	1.02e-03	6.44e-03
GPATC1	55094	G patch domain containing 1	6.78	5.95	5.42e-04	3.86e-03
GPATC3	63906	G patch domain containing 3	8.08	8.82	8.37e-03	3.33e-02
GPC1	2817	glypican 1	7.35	6.50	4.42e-03	2.05e-02
GPC4	2239	glypican 4	8.71	7.28	2.42e-04	2.00e-03
GPC6	10082	glypican 6	6.66	5.30	7.43e-03	3.03e-02
GPD1L	23171	glycerol-3-phosphate dehydrogenase 1-like	8.82	7.17	3.16e-05	3.73e-04
GPHN	10243	gephyrin	7.43	6.74	2.58e-03	1.35e-02
GPLD1	2822	glycosylphosphatidylinositol specific phospholipas	5.95	4.69	6.66e-06	1.02e-04
GPR1	2825	G protein-coupled receptor 1	10.66	11.83	5.21e-03	2.31e-02
GPR109B	8843	G protein-coupled receptor 109B	5.87	8.27	4.10e-03	1.93e-02
GPR125	166647	G protein-coupled receptor 125	6.94	5.85	3.71e-04	2.81e-03
GPR132	29933	G protein-coupled receptor 132	4.71	6.27	8.02e-06	1.18e-04
GPR137B	7107	G protein-coupled receptor 137B	9.34	10.41	8.16e-03	3.26e-02

Symbol	GeneID	Gene Name*	Signal CNTRL	AAA	P	FDR
GPR160	26996	G protein-coupled receptor 160	6.58	9.00	4.24e-03	1.99e-02
GPR162	27239	G protein-coupled receptor 162	6.60	7.96	9.61e-05	9.39e-04
GPR172A	79581	G protein-coupled receptor 172A	6.47	7.25	1.40e-02	4.94e-02
GPR177	79971	G protein-coupled receptor 177	8.91	7.95	2.61e-05	3.20e-04
GPR178	57583	G protein-coupled receptor 178	9.71	8.75	2.94e-05	3.53e-04
GPR18	2841	G protein-coupled receptor 18	4.66	6.91	1.09e-02	4.08e-02
GPR30	2852	G protein-coupled receptor 30	7.81	6.56	4.21e-03	1.97e-02
GPR34	2857	G protein-coupled receptor 34	7.54	8.42	8.98e-03	3.50e-02
GPR65	8477	G protein-coupled receptor 65	5.47	8.39	1.43e-09	8.43e-08
GPR68	8111	G protein-coupled receptor 68	5.43	7.14	5.83e-03	2.51e-02
GPR92	57121	G protein-coupled receptor 92	4.76	6.16	2.64e-03	1.37e-02
GPRASP1	9737	G protein-coupled receptor associated sorting prot	7.07	4.91	8.77e-04	5.72e-03
GPRASP2	114928	G protein-coupled receptor associated sorting prot	7.99	6.58	2.66e-08	9.95e-07
GPRC5A	9052	G protein-coupled receptor, family C, group 5, mem	8.50	7.42	7.22e-03	2.96e-02
GPRC5C	55890	G protein-coupled receptor, family C, group 5, mem	9.83	6.98	1.99e-17	1.15e-14
GPSM3	63940	G-protein signalling modulator 3 (AGS3-like, C. el	7.22	9.61	7.52e-09	3.31e-07
GPX1	2876	glutathione peroxidase 1	4.81	6.02	7.42e-03	3.03e-02
GPX3	2878	glutathione peroxidase 3 (plasma)	13.21	10.67	1.57e-07	4.45e-06
GRAMD3	65983	GRAM domain containing 3	8.29	6.85	2.90e-05	3.50e-04
GRAP	10750	GRB2-related adaptor protein	4.34	5.31	5.16e-03	2.29e-02
GRB10	2887	growth factor receptor-bound protein 10	7.43	6.53	1.20e-03	7.28e-03
GRB2	2885	growth factor receptor-bound protein 2	8.77	10.32	2.82e-06	5.02e-05
GRIA2	2891	glutamate receptor, ionotropic, AMPA 2	7.46	4.43	3.00e-12	3.94e-10
GRINL1A	81488	glutamate receptor, ionotropic, N-methyl D-asparta	10.50	9.09	2.73e-09	1.43e-07
GRK5	2869	G protein-coupled receptor kinase 5	10.51	8.46	2.21e-08	8.63e-07
GRN	2896	granulin	10.14	12.00	9.93e-05	9.65e-04
GSN	2934	gelsolin (amyloidosis, Finnish type)	12.95	11.60	1.71e-06	3.30e-05
GSTA4	2941	glutathione S-transferase A4	7.59	5.94	3.66e-06	6.17e-05
GSTM3	2947	glutathione S-transferase M3 (brain)	7.03	5.56	7.50e-06	1.12e-04
GSTO2	119391	glutathione S-transferase omega 2	6.42	5.10	2.14e-05	2.71e-04
GTF2H3	2967	general transcription factor IIH, polypeptide 3, 3	8.06	10.72	4.07e-03	1.93e-02
GUCY1A3	2982	guanylate cyclase 1, soluble, alpha 3	9.53	8.32	9.36e-04	6.02e-03
GUCY1B3	2983	guanylate cyclase 1, soluble, beta 3	8.09	6.89	1.07e-03	6.67e-03
GULP1	51454	GULP, engulfment adaptor PTB domain containing 1	7.53	5.74	4.71e-10	3.30e-08
GYG1	2992	glycogenin 1	11.16	10.20	2.25e-05	2.82e-04
GZMA	3001	granzyme A (granzyme 1, cytotoxic T-lymphocyte-ass	6.14	7.57	2.03e-03	1.11e-02
GZMB	3002	granzyme B (granzyme 2, cytotoxic T-lymphocyte-ass	5.32	7.75	2.19e-03	1.18e-02
GZMH	2999	granzyme H (cathepsin G-like 2, protein h-CCPX)	5.30	6.45	6.07e-04	4.24e-03
GZMK	3003	granzyme K (granzyme 3; tryptase II)	6.55	8.31	1.01e-02	3.85e-02
H2AFJ	55766	H2A histone family, member J	7.61	5.08	3.47e-10	2.57e-08
H2AFV	94239	H2A histone family, member V	8.28	7.64	1.25e-02	4.53e-02
H2AFX	3014	H2A histone family, member X	8.85	9.92	2.86e-04	2.29e-03
H2AFY	9555	H2A histone family, member Y	10.43	11.61	5.20e-03	2.31e-02
HABP4	22927	hyaluronan binding protein 4	8.39	7.48	1.35e-02	4.79e-02
HADHSC	3033	L-3-hydroxyacyl-Coenzyme A dehydrogenase, short ch	9.55	8.60	3.17e-05	3.73e-04
HAVCR2	84868	hepatitis A virus cellular receptor 2	8.55	10.99	2.12e-03	1.15e-02
HBLD2	81689	HESB like domain containing 2	9.12	8.42	3.61e-03	1.75e-02
HBXAP	51773	hepatitis B virus x associated protein	7.05	6.22	3.99e-04	2.98e-03
HCAP-G	64151	chromosome condensation protein G	5.46	6.79	1.64e-03	9.33e-03
HCK	3055	hemopoietic cell kinase	6.51	8.44	8.61e-07	1.85e-05
HCLS1	3059	hematopoietic cell-specific Lyn substrate 1	9.11	11.11	1.39e-07	4.01e-06
HCST	10870	hematopoietic cell signal transducer	7.36	9.56	4.53e-04	3.33e-03
HDAC11	79885	histone deacetylase 11	6.12	5.13	2.83e-04	2.27e-03
HDAC5	10014	histone deacetylase 5	6.35	5.30	7.98e-04	5.31e-03
HDAC9	9734	histone deacetylase 9	6.53	5.35	5.22e-03	2.32e-02
HDC	3067	histidine decarboxylase	4.55	5.44	1.38e-02	4.89e-02
HDDC3	374659	HD domain containing 3	6.60	7.37	5.96e-03	2.56e-02
HDGFRP3	50810	hepatoma-derived growth factor, related protein 3	9.84	8.75	2.07e-06	3.87e-05
HEAB	10978	ATP/GTP-binding protein	6.14	7.13	7.83e-04	5.23e-03
HECTD2	143279	HECT domain containing 2	4.97	3.45	1.28e-02	4.63e-02
HEPH	9843	hephaestin	9.86	7.65	2.35e-07	6.12e-06
HES1	3280	hairy and enhancer of split 1, (Drosophila)	8.15	6.54	1.46e-07	4.20e-06
HES4	57801	hairy and enhancer of split 4 (Drosophila)	10.16	9.17	1.70e-04	1.50e-03
HEXIM1	10614	hexamethylene bis-acetamide inducible 1	9.91	8.87	1.50e-04	1.36e-03
HEY2	23493	hairy/enhancer-of-split related with YRPW motif 2	8.54	5.39	1.43e-06	2.84e-05
HEYL	26508	hairy/enhancer-of-split related with YRPW motif-li	9.29	7.51	7.27e-09	3.25e-07
HGF	3082	hepatocyte growth factor (hepapoietin A; scatter f	4.96	5.85	4.06e-03	1.92e-02

Symbol	GeneID	Gene Name*	Signal CNTRL	Signal AAA	P	FDR
HIBCH	26275	3-hydroxyisobutyryl-Coenzyme A hydrolase	8.34	7.52	2.83e-04	2.27e-03
HIC2	23119	hypermethylated in cancer 2	5.04	5.89	7.24e-03	2.97e-02
HIF3A	64344	hypoxia inducible factor 3, alpha subunit	5.39	4.40	6.49e-03	2.73e-02
HIG2	29923	hypoxia-inducible protein 2	9.93	6.55	2.33e-09	1.25e-07
HIGD1A	25994	HIG1 domain family, member 1A	10.62	9.61	6.82e-04	4.68e-03
HIGD2A	192286	HIG1 domain family, member 2A	7.87	8.68	8.48e-03	3.36e-02
HIPK3	10114	homeodomain interacting protein kinase 3	6.37	5.69	1.29e-02	4.64e-02
HK3	3101	hexokinase 3 (white cell)	5.17	7.79	6.51e-03	2.74e-02
HLA-B	3106	major histocompatibility complex, class I, B	11.61	12.94	2.53e-05	3.11e-04
HLA-C	3107	major histocompatibility complex, class I, C	11.54	12.38	2.94e-03	1.49e-02
HLA-DMA	3108	major histocompatibility complex, class II, DM alp	10.45	12.31	2.91e-07	7.39e-06
HLA-DMB	3109	major histocompatibility complex, class II, DM bet	9.82	11.95	3.09e-08	1.12e-06
HLA-DOA	3111	major histocompatibility complex, class II, DO alp	4.73	6.59	2.51e-03	1.32e-02
HLA-DPA1	3113	major histocompatibility complex, class II, DP alp	12.18	13.36	9.36e-05	9.20e-04
HLA-DPB1	3115	major histocompatibility complex, class II, DP bet	12.54	13.90	1.90e-05	2.45e-04
HLA-DQA1	3117	major histocompatibility complex, class II, DQ alp	8.87	11.44	4.43e-03	2.06e-02
HLA-DQB1	3119	major histocompatibility complex, class II, DQ bet	8.69	10.92	3.18e-07	7.92e-06
HLA-DQB2	3120	major histocompatibility complex, class II, DQ bet	4.32	6.19	3.27e-03	1.62e-02
HLA-DRA	3122	major histocompatibility complex, class II, DR alp	10.44	12.49	2.20e-07	5.83e-06
HLA-DRB4	3126	major histocompatibility complex, class II, DR bet	10.44	12.70	7.72e-08	2.44e-06
HLA-E	3133	major histocompatibility complex, class I, E	10.40	11.35	4.69e-03	2.14e-02
HLA-G	3135	HLA-G histocompatibility antigen, class I, G	5.44	6.38	1.54e-03	8.90e-03
HLF	3131	hepatic leukemia factor	6.91	5.44	4.33e-04	3.20e-03
HMCN1	83872	hemicentin 1	7.84	4.98	9.34e-03	3.61e-02
HMGA1	3159	high mobility group AT-hook 1	5.86	7.34	4.53e-05	5.04e-04
HMGN1	3150	high-mobility group nucleosome binding domain 1	9.87	10.71	8.06e-03	3.23e-02
HMHA1	23526	histocompatibility (minor) HA-1	5.65	8.36	1.86e-06	3.53e-05
HMOX1	3162	heme oxygenase (decycling) 1	8.56	12.47	3.30e-03	1.63e-02
HN1	51155	hematological and neurological expressed 1	8.16	10.10	3.93e-04	2.95e-03
HNRPA1	3178	heterogeneous nuclear ribonucleoprotein A1	6.89	5.71	6.80e-03	2.82e-02
HNRPH3	3189	heterogeneous nuclear ribonucleoprotein H3 (2H9)	9.60	8.88	1.32e-03	7.87e-03
HOXA2	3199	homeobox A2	7.00	6.30	2.64e-03	1.38e-02
HOXA4	3201	homeobox A4	7.22	4.75	2.57e-09	1.37e-07
HOXA5	3202	homeobox A5	8.78	7.12	7.78e-07	1.71e-05
HOXA7	3204	homeobox A7	8.07	7.22	1.23e-02	4.48e-02
HOXB2	3212	homeobox B2	8.30	6.96	1.02e-02	3.89e-02
HOXB3	3213	homeobox B3	6.34	5.29	3.45e-05	3.99e-04
HOXB5	3215	homeobox B5	5.88	4.54	2.37e-05	2.95e-04
HOXB7	3217	homeobox B7	7.87	6.69	2.40e-03	1.27e-02
HOXC6	3223	homeobox C6	9.61	7.88	7.89e-06	1.17e-04
HOXD8	3234	homeobox D8	7.52	6.57	2.75e-03	1.41e-02
HPCAL1	3241	hippocalcin-like 1	9.53	10.42	1.62e-03	9.21e-03
HPD	3242	4-hydroxyphenylpyruvate dioxygenase	6.67	5.12	1.21e-03	7.31e-03
HPGD	3248	hydroxyprostaglandin dehydrogenase 15-(NAD)	5.68	6.81	1.31e-02	4.70e-02
HPS6	79803	Hermansky-Pudlak syndrome 6	7.44	8.25	3.47e-03	1.70e-02
HRASLS3	11145	HRAS-like suppressor 3	10.09	8.77	2.01e-05	2.58e-04
HRC	3270	histidine rich calcium binding protein	5.88	4.58	6.90e-03	2.86e-02
HRH1	3269	histamine receptor H1	7.73	5.62	5.33e-07	1.24e-05
HRSP12	10247	heat-responsive protein 12	6.72	6.10	8.15e-03	3.26e-02
HS3ST1	9957	heparan sulfate (glucosamine) 3-O-sulfotransferase	6.06	8.76	2.84e-03	1.45e-02
HS3ST3A1	9955	heparan sulfate (glucosamine) 3-O-sulfotransferase	5.05	6.94	3.90e-03	1.86e-02
HSA9761	27292	dimethyladenosine transferase	10.22	9.52	2.22e-03	1.20e-02
HSD17B12	51144	hydroxysteroid (17-beta) dehydrogenase 12	11.28	10.35	2.95e-04	2.34e-03
HSD17B8	7923	hydroxysteroid (17-beta) dehydrogenase 8	5.76	6.91	2.68e-03	1.39e-02
HSD3B7	80270	hydroxy-delta-5-steroid dehydrogenase, 3 beta- and	7.11	8.49	4.56e-03	2.10e-02
HSDL2	84263	hydroxysteroid dehydrogenase like 2	9.21	8.31	8.09e-05	8.17e-04
HSF2	3298	heat shock transcription factor 2	8.68	7.96	1.55e-03	8.92e-03
HSH2D	84941	hematopoietic SH2 domain containing	3.88	7.04	1.17e-03	7.18e-03
HSPA2	3306	heat shock 70kDa protein 2	8.57	6.96	5.76e-07	1.32e-05
HSPA4L	22824	heat shock 70kDa protein 4-like	7.20	5.29	1.19e-12	1.74e-10
HSPB1	3315	heat shock 27kDa protein 1	10.96	9.44	7.42e-06	1.11e-04
HSPB2	3316	heat shock 27kDa protein 2	9.16	6.84	1.70e-13	2.92e-11
HSPB6	126393	heat shock protein, alpha-crystallin-related, B6	9.96	7.74	7.57e-04	5.08e-03
HSPB7	27129	heat shock 27kDa protein family, member 7 (cardiov	10.28	6.76	1.14e-21	3.28e-18
HSPB8	26353	heat shock 22kDa protein 8	10.55	8.59	9.78e-06	1.41e-04
HSPG2	3339	heparan sulfate proteoglycan 2 (perlecan)	7.84	5.62	1.86e-03	1.03e-02
HSU79303	29903	protein predicted by clone 23882	8.31	9.05	8.95e-03	3.50e-02

Symbol	GeneID	Gene Name*	Signal CNTRL	AAA	P	FDR
HTR2A	3356	5-hydroxytryptamine (serotonin) receptor 2A	6.84	5.33	1.21e-04	1.13e-03
HTRA1	5654	HtrA serine peptidase 1	11.99	10.97	1.73e-04	1.52e-03
HTRA3	94031	HtrA serine peptidase 3	10.56	9.56	6.10e-03	2.60e-02
ICAM1	3383	intercellular adhesion molecule 1 (CD54), human rh	5.45	6.58	9.60e-04	6.14e-03
ICAM3	3385	intercellular adhesion molecule 3	6.97	9.41	6.77e-05	7.06e-04
ICAM4	3386	intercellular adhesion molecule 4 (LW blood group)	5.29	6.33	4.73e-03	2.14e-02
ICMT	23463	isoprenylcysteine carboxyl methyltransferase	8.91	7.89	1.11e-05	1.58e-04
ICOS	29851	inducible T-cell co-stimulator	4.64	6.89	3.59e-05	4.12e-04
ID3	3399	inhibitor of DNA binding 3, dominant negative heli	10.65	9.70	3.31e-03	1.63e-02
ID4	3400	inhibitor of DNA binding 4, dominant negative heli	9.16	7.22	7.06e-05	7.33e-04
IDH2	3418	isocitrate dehydrogenase 2 (NADP+), mitochondrial	9.32	10.48	1.14e-04	1.09e-03
IER3	8870	immediate early response 3	10.00	11.52	1.33e-03	7.88e-03
IER5	51278	immediate early response 5	7.44	8.34	1.36e-03	8.03e-03
IFI30	10437	interferon, gamma-inducible protein 30	9.79	12.91	2.14e-03	1.16e-02
IFI35	3430	interferon-induced protein 35	8.20	9.04	4.91e-03	2.21e-02
IFI44	10561	interferon-induced protein 44	7.87	8.66	4.66e-03	2.13e-02
IFIT1	3434	interferon-induced protein with tetratricopeptide	7.49	8.33	9.55e-03	3.67e-02
IFIT3	3437	interferon-induced protein with tetratricopeptide	6.35	7.64	1.90e-03	1.05e-02
IFITM3	10410	interferon induced transmembrane protein 3 (1-8U)	13.70	12.79	6.45e-03	2.72e-02
IFNGR1	3459	interferon gamma receptor 1	9.97	10.72	5.88e-03	2.53e-02
IFNGR2	3460	interferon gamma receptor 2 (interferon gamma tran	10.52	11.40	5.08e-03	2.27e-02
IFT52	51098	intraflagellar transport 52 homolog (Chlamydomonas	9.12	8.57	1.37e-02	4.87e-02
IFT81	28981	intraflagellar transport 81 homolog (Chlamydomonas	6.31	5.13	8.01e-06	1.18e-04
IGF1	3479	insulin-like growth factor 1 (somatomedin C)	9.60	8.04	1.75e-03	9.84e-03
IGFBP2	3485	insulin-like growth factor binding protein 2, 36kD	11.42	8.38	1.82e-10	1.50e-08
IGFBP3	3486	insulin-like growth factor binding protein 3	12.39	11.17	1.46e-04	1.33e-03
IGFBP4	3487	insulin-like growth factor binding protein 4	6.03	5.38	1.01e-02	3.83e-02
IGFBP5	3488	insulin-like growth factor binding protein 5	8.49	5.89	3.30e-03	1.63e-02
IGFBP6	3489	insulin-like growth factor binding protein 6	12.17	10.11	6.09e-05	6.47e-04
IGFBP7	3490	insulin-like growth factor binding protein 7	12.94	11.58	3.83e-04	2.88e-03
IGJ	3512	immunoglobulin J polypeptide, linker protein for i	8.48	11.44	2.78e-03	1.43e-02
IGSF10	285313	immunoglobulin superfamily, member 10	6.22	5.39	1.01e-02	3.85e-02
IGSF4	23705	immunoglobulin superfamily, member 4	6.09	7.96	3.01e-05	3.58e-04
IGSF6	10261	immunoglobulin superfamily, member 6	5.89	8.44	2.64e-09	1.39e-07
IKBKE	9641	inhibitor of kappa light polypeptide gene enhancer	4.68	6.48	3.98e-06	6.63e-05
IKBKG	8517	inhibitor of kappa light polypeptide gene enhancer	8.06	8.80	6.75e-03	2.81e-02
IL10	3586	interleukin 10	5.23	6.74	3.73e-04	2.82e-03
IL10RA	3587	interleukin 10 receptor, alpha	5.94	8.44	3.47e-09	1.74e-07
IL10RB	3588	interleukin 10 receptor, beta	9.71	10.88	1.28e-02	4.61e-02
IL12RB1	3594	interleukin 12 receptor, beta 1	5.12	6.68	4.58e-06	7.46e-05
IL13RA2	3598	interleukin 13 receptor, alpha 2	9.09	4.95	4.12e-07	9.81e-06
IL16	3603	interleukin 16 (lymphocyte chemoattractant factor)	5.88	7.37	8.51e-04	5.61e-03
IL17D	53342	interleukin 17D	7.76	6.80	5.02e-03	2.25e-02
IL17R	23765	interleukin 17 receptor	6.05	6.74	1.25e-02	4.54e-02
IL18	3606	interleukin 18 (interferon-gamma-inducing factor)	9.57	10.85	6.18e-03	2.63e-02
IL1B	3553	interleukin 1, beta	6.27	9.81	9.03e-11	8.08e-09
IL1R1	3554	interleukin 1 receptor, type I	10.16	9.47	1.20e-02	4.38e-02
IL1R2	7850	interleukin 1 receptor, type II	7.39	9.32	1.12e-02	4.16e-02
IL21R	50615	interleukin 21 receptor	5.41	6.79	6.32e-05	6.69e-04
IL23A	51561	interleukin 23, alpha subunit p19	4.53	5.48	4.54e-03	2.09e-02
IL27RA	9466	interleukin 27 receptor, alpha	6.14	7.24	1.38e-03	8.14e-03
IL28RA	163702	interleukin 28 receptor, alpha (interferon, lambda	4.66	6.91	4.28e-08	1.46e-06
IL2RB	3560	interleukin 2 receptor, beta	4.62	7.64	1.56e-03	8.96e-03
IL2RG	3561	interleukin 2 receptor, gamma (severe combined imm	6.04	8.69	4.44e-09	2.13e-07
IL4R	3566	interleukin 4 receptor	9.37	10.45	2.52e-04	2.07e-03
IL8	3576	interleukin 8	8.72	12.23	4.35e-03	2.03e-02
ILK	3611	integrin-linked kinase	11.78	10.61	3.98e-07	9.54e-06
IMMP2L	83943	IMP2 inner mitochondrial membrane peptidase-like (	8.02	6.69	2.17e-08	8.49e-07
IMPDH1	3614	IMP (inosine monophosphate) dehydrogenase 1	8.45	9.65	9.07e-05	8.96e-04
INADL	10207	InaD-like (Drosophila)	6.55	5.52	1.87e-03	1.04e-02
INDO	3620	indoleamine-pyrrole 2,3 dioxygenase	5.37	7.56	1.43e-03	8.35e-03
ING3	54556	inhibitor of growth family, member 3	4.98	6.17	1.41e-02	4.97e-02
INPP4B	8821	inositol polyphosphate-4-phosphatase, type II, 105	7.83	6.53	7.36e-04	4.98e-03
INPP5A	3632	inositol polyphosphate-5-phosphatase, 40kDa	10.57	8.94	3.60e-09	1.77e-07
INPP5D	3635	inositol polyphosphate-5-phosphatase, 145kDa	6.51	8.54	1.07e-06	2.23e-05
INSIG1	3638	insulin induced gene 1	5.05	6.74	2.87e-03	1.46e-02
INSIG2	51141	insulin induced gene 2	9.10	8.36	9.90e-04	6.28e-03

Symbol	GeneID	Gene Name*	Signal CNTRL	AAA	P	FDR
IPO7	10527	importin 7	8.51	7.64	1.04e-03	6.56e-03
IQCG	84223	IQ motif containing G	6.06	6.77	1.07e-02	4.01e-02
IQGAP2	10788	IQ motif containing GTPase activating protein 2	7.41	8.92	1.29e-03	7.69e-03
IQGAP3	128239	IQ motif containing GTPase activating protein 3	7.50	11.56	2.61e-03	1.36e-02
IRAK1	3654	interleukin-1 receptor-associated kinase 1	8.55	10.21	1.63e-05	2.16e-04
IRF1	3659	interferon regulatory factor 1	8.26	9.81	9.98e-06	1.43e-04
IRF4	3662	interferon regulatory factor 4	4.36	7.26	1.61e-03	9.18e-03
IRF5	3663	interferon regulatory factor 5	4.67	6.46	8.58e-05	8.58e-04
IRF7	3665	interferon regulatory factor 7	8.06	9.98	1.92e-05	2.46e-04
IRF8	3394	interferon regulatory factor 8	4.25	7.82	1.56e-07	4.43e-06
IRS1	3667	insulin receptor substrate 1	5.57	3.93	5.03e-04	3.63e-03
IRX1	79192	iroquois homeobox protein 1	6.42	4.44	3.10e-03	1.55e-02
IRXL1	283078	iroquois homeobox protein-like 1	8.73	6.56	7.55e-09	3.31e-07
ISG20	3669	interferon stimulated exonuclease gene 20kDa	8.17	10.16	8.57e-04	5.64e-03
ISG20L2	81875	interferon stimulated exonuclease gene 20kDa-like	6.62	7.33	8.73e-03	3.44e-02
ISYNA1	51477	myo-inositol 1-phosphate synthase A1	8.74	7.88	1.10e-02	4.10e-02
ITGA10	8515	integrin, alpha 10	6.79	4.32	6.83e-04	4.69e-03
ITGA11	22801	integrin, alpha 11	7.75	5.36	1.81e-12	2.52e-10
ITGA3	3675	integrin, alpha 3 (antigen CD49C, alpha 3 subunit	7.79	6.62	3.53e-04	2.70e-03
ITGA4	3676	integrin, alpha 4 (antigen CD49D, alpha 4 subunit	3.48	5.54	6.43e-04	4.45e-03
ITGA5	3678	integrin, alpha 5 (fibronectin receptor, alpha pol	9.29	8.43	4.98e-03	2.23e-02
ITGA7	3679	integrin, alpha 7	6.71	5.17	1.86e-05	2.40e-04
ITGA8	8516	integrin, alpha 8	11.05	6.57	4.61e-06	7.50e-05
ITGAL	3683	integrin, alpha L (antigen CD11A (p180), lymphocyt	5.31	7.61	1.20e-08	4.96e-07
ITGAM	3684	integrin, alpha M (complement component 3 receptor	7.42	9.40	3.75e-03	1.81e-02
ITGAX	3687	integrin, alpha X (complement component 3 receptor	7.38	9.92	1.20e-04	1.13e-03
ITGB1	3688	integrin, beta 1 (fibronectin receptor, beta polyp	10.89	9.50	1.60e-06	3.12e-05
ITGB1BP1	9270	integrin beta 1 binding protein 1	7.12	8.71	8.60e-03	3.40e-02
ITGB1BP2	26548	integrin beta 1 binding protein (melusin) 2	5.51	4.65	8.55e-03	3.38e-02
ITGB2	3689	integrin, beta 2 (antigen CD18 (p95), lymphocyte f	8.30	10.34	3.06e-07	7.67e-06
ITGB5	3693	integrin, beta 5	12.28	11.07	2.78e-04	2.24e-03
ITGB7	3695	integrin, beta 7	4.77	7.67	2.25e-06	4.15e-05
ITGBL1	9358	integrin, beta-like 1 (with EGF-like repeat domain	10.74	9.48	5.46e-03	2.39e-02
ITIH4	3700	inter-alpha (globulin) inhibitor H4 (plasma Kallik	7.14	5.09	9.82e-04	6.25e-03
ITLN1	55600	intelectin 1 (galactofuranose binding)	7.11	4.73	8.28e-04	5.47e-03
ITPKB	3707	inositol 1,4,5-trisphosphate 3-kinase B	6.86	7.59	8.78e-03	3.45e-02
ITPR1	3708	inositol 1,4,5-triphosphate receptor, type 1	9.96	8.78	3.60e-06	6.11e-05
JAG1	182	jagged 1 (Alagille syndrome)	8.63	6.53	1.97e-11	2.15e-09
JAK2	3717	Janus kinase 2 (a protein tyrosine kinase)	7.94	6.65	3.60e-04	2.74e-03
JAM2	58494	junctional adhesion molecule 2	7.49	6.56	5.68e-03	2.47e-02
JAM3	83700	junctional adhesion molecule 3	11.38	9.28	1.20e-13	2.13e-11
JARID2	3720	Jumonji, AT rich interactive domain 2	6.77	7.86	2.33e-04	1.95e-03
JAZF1	221895	juxtaposed with another zinc finger gene 1	9.39	8.77	6.67e-03	2.78e-02
JDP2	122953	jun dimerization protein 2	7.31	8.03	7.56e-03	3.07e-02
JPH2	57158	junctophilin 2	8.57	6.01	1.34e-06	2.68e-05
JRKL	8690	jerky homolog-like (mouse)	6.20	5.55	7.92e-03	3.19e-02
JUB	84962	jub, ajuba homolog (Xenopus laevis)	6.69	5.39	8.23e-04	5.44e-03
JUNB	3726	jun B proto-oncogene	4.91	6.03	1.17e-02	4.31e-02
KARCA1	126823	kelch/ankyrin repeat containing cyclin A1 interact	6.60	5.90	8.91e-03	3.49e-02
KATNAL1	84056	katanin p60 subunit A-like 1	8.07	7.24	6.66e-04	4.59e-03
KAZALD1	81621	Kazal-type serine peptidase inhibitor domain 1	7.81	6.68	3.45e-04	2.66e-03
KBTBD11	9920	kelch repeat and BTB (POZ) domain containing 11	5.42	7.47	2.37e-03	1.25e-02
KBTBD7	84078	kelch repeat and BTB (POZ) domain containing 7	6.78	6.06	1.14e-02	4.22e-02
KBTBD8	84541	kelch repeat and BTB (POZ) domain containing 8	5.31	7.02	2.27e-04	1.90e-03
KCNA5	3741	potassium voltage-gated channel, shaker-related su	10.10	5.12	2.30e-09	1.24e-07
KCNAB1	7881	potassium voltage-gated channel, shaker-related su	10.72	8.11	1.32e-07	3.83e-06
KCND1	3750	potassium voltage-gated channel, Shal-related subf	6.49	5.58	1.99e-04	1.70e-03
KCNE3	10008	potassium voltage-gated channel, Isk-related famil	6.81	8.26	8.39e-03	3.33e-02
KCNIP3	30818	Kv channel interacting protein 3, calsenilin	6.83	5.57	6.76e-05	7.06e-04
KCNJ15	3772	potassium inwardly-rectifying channel, subfamily J	5.27	6.08	7.15e-03	2.94e-02
KCNJ8	3764	potassium inwardly-rectifying channel, subfamily J	7.62	6.16	9.39e-04	6.03e-03
KCNK1	3775	potassium channel, subfamily K, member 1	6.22	4.63	2.08e-04	1.76e-03
KCNK13	56659	potassium channel, subfamily K, member 13	4.05	6.34	4.28e-03	2.00e-02
KCNK15	60598	potassium channel, subfamily K, member 15	8.65	6.30	1.69e-04	1.50e-03
KCNK17	89822	potassium channel, subfamily K, member 17	6.69	4.91	7.08e-04	4.82e-03
KCNK3	3777	potassium channel, subfamily K, member 3	6.66	5.34	1.65e-06	3.19e-05
KCNMA1	3778	potassium large conductance calcium-activated chan	10.88	8.93	5.29e-10	3.66e-08

Symbol	GeneID	Gene Name*	Signal CNTRL	AAA	P	FDR
KCNMB1	3779	potassium large conductance calcium-activated chan	10.96	8.71	2.06e-14	4.67e-12
KCNN4	3783	potassium intermediate/small conductance calcium-a	5.28	8.03	2.86e-07	7.28e-06
KCNS3	3790	potassium voltage-gated channel, delayed-rectifier	7.03	5.67	6.48e-05	6.82e-04
KCTD10	83892	potassium channel tetramerisation domain containin	9.34	8.06	4.28e-08	1.46e-06
KCTD14	65987	potassium channel tetramerisation domain containin	6.91	6.01	1.96e-03	1.08e-02
KCTD15	79047	potassium channel tetramerisation domain containin	6.14	5.34	6.70e-03	2.79e-02
KCTD3	51133	potassium channel tetramerisation domain containin	9.12	8.25	1.22e-04	1.14e-03
KCTD5	54442	potassium channel tetramerisation domain containin	5.45	6.54	4.91e-04	3.56e-03
KCTD9	54793	potassium channel tetramerisation domain containin	9.07	7.65	2.12e-09	1.16e-07
KDELC1	79070	KDEL (Lys-Asp-Glu-Leu) containing 1	6.53	5.54	5.25e-04	3.75e-03
KDELC2	143888	KDEL (Lys-Asp-Glu-Leu) containing 2	9.25	8.21	1.61e-04	1.44e-03
KHDRBS3	10656	KH domain containing, RNA binding, signal transduc	8.74	7.41	2.40e-04	1.99e-03
KIAA0101	9768	KIAA0101	4.28	5.68	5.23e-03	2.32e-02
KIAA0103	9694	KIAA0103	8.67	7.76	5.67e-05	6.12e-04
KIAA0114	57291	KIAA0114 gene product	7.32	6.61	3.76e-03	1.81e-02
KIAA0194	22993	KIAA0194 protein	7.57	8.40	2.81e-03	1.44e-02
KIAA0226	9711	KIAA0226	8.60	9.33	8.86e-03	3.47e-02
KIAA0329	9895	KIAA0329	6.52	5.85	1.04e-02	3.94e-02
KIAA0350	23274	KIAA0350 protein	6.73	7.42	1.16e-02	4.28e-02
KIAA0367	23273	KIAA0367	9.91	6.65	8.93e-08	2.78e-06
KIAA0415	9907	KIAA0415 protein	4.68	5.96	7.15e-03	2.94e-02
KIAA0446	9673	KIAA0446 gene product	8.11	9.24	2.73e-03	1.41e-02
KIAA0492	57238	KIAA0492 protein	7.70	8.86	3.82e-03	1.84e-02
KIAA0513	9764	KIAA0513	6.24	7.80	4.63e-03	2.12e-02
KIAA0649	9858	KIAA0649	6.97	6.25	2.08e-03	1.13e-02
KIAA0672	9912	KIAA0672 gene product	7.55	6.26	1.78e-04	1.55e-03
KIAA0674	23307	KIAA0674	7.25	8.14	5.16e-03	2.29e-02
KIAA0690	23223	KIAA0690	6.03	7.61	3.06e-05	3.63e-04
KIAA0746	23231	KIAA0746 protein	6.86	9.31	1.09e-03	6.74e-03
KIAA0774	23281	KIAA0774	6.44	4.28	5.81e-04	4.08e-03
KIAA0831	22863	KIAA0831	8.24	7.15	2.84e-06	5.06e-05
KIAA0907	22889	KIAA0907	8.84	8.28	1.41e-02	4.99e-02
KIAA0960	23249	KIAA0960 protein	6.59	5.76	9.95e-03	3.81e-02
KIAA1107	23285	KIAA1107	6.39	5.27	2.07e-04	1.75e-03
KIAA1128	54462	KIAA1128	8.50	7.47	8.49e-06	1.25e-04
KIAA1199	57214	KIAA1199	6.94	9.97	1.32e-02	4.71e-02
KIAA1324	57535	KIAA1324	5.17	7.30	1.30e-06	2.66e-05
KIAA1328	57536	KIAA1328	6.05	5.01	1.16e-02	4.27e-02
KIAA1345	57545	KIAA1345 protein	7.27	6.24	1.43e-03	8.35e-03
KIAA1377	57562	KIAA1377	6.63	5.02	6.30e-09	2.86e-07
KIAA1430	57587	KIAA1430	9.49	8.34	5.30e-05	5.77e-04
KIAA1509	440193	KIAA1509	5.61	7.27	1.51e-03	8.72e-03
KIAA1576	57687	KIAA1576 protein	8.27	5.11	4.72e-03	2.14e-02
KIAA1598	57698	KIAA1598	8.18	9.48	5.63e-04	3.98e-03
KIAA1600	57700	KIAA1600	9.75	8.96	1.71e-03	9.64e-03
KIAA1683	80726	KIAA1683	4.91	6.55	8.68e-04	5.69e-03
KIAA1713	80816	KIAA1713	5.71	4.32	1.25e-02	4.53e-02
KIAA1794	55215	KIAA1794	5.05	6.17	1.50e-03	8.70e-03
KIAA1912	114800	KIAA1912 protein	6.07	5.05	6.64e-03	2.78e-02
KIAA1949	170954	KIAA1949	8.29	9.27	6.15e-04	4.30e-03
KIAA1967	57805	KIAA1967	6.19	6.99	5.47e-03	2.39e-02
KIDINS220	57498	kinase D-interacting substance of 220 kDa	9.51	8.77	1.09e-03	6.77e-03
KIF11	3832	kinesin family member 11	3.31	5.32	4.88e-03	2.20e-02
KIF1C	10749	kinesin family member 1C	8.18	6.94	1.14e-07	3.40e-06
KIF2C	11004	kinesin family member 2C	5.04	6.06	7.06e-03	2.91e-02
KIF7	374654	kinesin family member 7	7.81	5.99	1.97e-07	5.31e-06
KIFAP3	22920	kinesin-associated protein 3	8.41	7.81	8.29e-03	3.30e-02
KIFC3	3801	kinesin family member C3	8.51	9.31	1.41e-02	4.97e-02
KLC2	64837	kinesin light chain 2	8.40	7.39	1.25e-05	1.74e-04
KLF10	7071	Kruppel-like factor 10	6.83	7.71	5.44e-03	2.39e-02
KLF13	51621	Kruppel-like factor 13	8.94	9.73	4.69e-03	2.14e-02
KLF15	28999	Kruppel-like factor 15	7.79	4.95	5.87e-08	1.93e-06
KLF16	83855	Kruppel-like factor 16	5.52	7.02	5.87e-05	6.27e-04
KLF4	9314	Kruppel-like factor 4 (gut)	10.03	11.10	6.27e-03	2.66e-02
KLF6	1316	Kruppel-like factor 6	9.63	10.46	7.97e-03	3.21e-02
KLF8	11279	Kruppel-like factor 8	6.79	5.26	5.96e-06	9.29e-05
KLF9	687	Kruppel-like factor 9	11.62	10.37	1.27e-04	1.18e-03

Symbol	GeneID	Gene Name*	Signal CNTRL	AAA	P	FDR
KLHDC1	122773	kelch domain containing 1	8.17	7.25	8.71e-05	8.68e-04
KLHDC2	23588	kelch domain containing 2	10.44	9.27	9.54e-05	9.34e-04
KLHDC5	57542	kelch domain containing 5	9.61	7.71	3.04e-13	5.08e-11
KLHL25	64410	kelch-like 25 (Drosophila)	6.98	6.29	3.30e-03	1.63e-02
KLHL6	89857	kelch-like 6 (Drosophila)	5.62	8.36	8.88e-07	1.91e-05
KLHL7	55975	kelch-like 7 (Drosophila)	7.47	6.79	3.34e-03	1.64e-02
KLHL9	55958	kelch-like 9 (Drosophila)	9.06	8.22	5.50e-04	3.90e-03
KLK1	3816	kallikrein 1, renal/pancreas/salivary	5.12	6.82	4.78e-03	2.16e-02
KM-HN-1	256309	KM-HN-1 protein	6.13	4.45	2.57e-03	1.35e-02
KMO	8564	kynurenine 3-monooxygenase (kynurenine 3-hydroxyla	4.54	6.90	1.88e-04	1.62e-03
KPNA2	3838	karyopherin alpha 2 (RAG cohort 1, importin alpha	8.55	9.54	1.89e-03	1.05e-02
KRT17	3872	keratin 17	8.13	6.76	9.05e-03	3.52e-02
KRT19	3880	keratin 19	7.36	6.28	4.11e-03	1.94e-02
KRTHB6	3892	keratin, hair, basic, 6 (monilethrix)	6.73	5.48	3.64e-05	4.18e-04
KTI12	112970	KTI12 homolog, chromatin associated (S. cerevisiae	6.79	7.66	2.04e-03	1.12e-02
KTN1	3895	kinectin 1 (kinesin receptor)	10.68	9.91	6.48e-04	4.48e-03
Kua	387521	ubiquitin-conjugating enzyme variant Kua	7.93	8.65	9.04e-03	3.52e-02
KYNU	8942	kynureninase (L-kynurenine hydrolase)	7.37	9.54	4.80e-04	3.49e-03
L2HGDH	79944	L-2-hydroxyglutarate dehydrogenase	6.12	5.19	2.77e-03	1.42e-02
L3MBTL	26013	l(3)mbt-like (Drosophila)	5.38	4.02	5.96e-03	2.56e-02
LACTB	114294	lactamase, beta	7.04	9.08	2.87e-04	2.29e-03
LAIR1	3903	leukocyte-associated Ig-like receptor 1	5.98	7.04	1.08e-03	6.70e-03
LAMA2	3908	laminin, alpha 2 (merosin, congenital muscular dys	9.30	6.93	2.52e-04	2.07e-03
LAMA3	3909	laminin, alpha 3	6.78	5.63	8.87e-03	3.47e-02
LAMA5	3911	laminin, alpha 5	10.71	8.85	1.65e-04	1.47e-03
LAMB1	3912	laminin, beta 1	9.84	8.83	1.16e-03	7.11e-03
LAMB2	3913	laminin, beta 2 (laminin S)	8.76	7.69	2.81e-05	3.40e-04
LAMB3	3914	laminin, beta 3	5.64	7.01	2.36e-03	1.25e-02
LAMC1	3915	laminin, gamma 1 (formerly LAMB2)	11.29	9.72	1.29e-07	3.78e-06
LAMP3	27074	lysosomal-associated membrane protein 3	4.21	6.79	1.62e-05	2.15e-04
LANCL1	10314	LanC lantibiotic synthetase component C-like 1 (ba	9.94	8.69	2.28e-07	5.99e-06
LAP3	51056	leucine aminopeptidase 3	9.73	10.51	6.23e-03	2.64e-02
LAPTM4A	9741	lysosomal-associated protein transmembrane 4 alpha	11.04	10.43	7.01e-03	2.89e-02
LAPTM4B	55353	lysosomal associated protein transmembrane 4 beta	9.09	8.25	6.92e-03	2.86e-02
LAPTM5	7805	lysosomal associated multispanning membrane protei	8.17	10.75	1.67e-04	1.48e-03
LARGE	9215	like-glycosyltransferase	8.22	7.04	3.97e-04	2.98e-03
LARP2	55132	La ribonucleoprotein domain family, member 2	6.66	6.07	1.23e-02	4.47e-02
LARP6	55323	La ribonucleoprotein domain family, member 6	9.03	7.27	6.60e-08	2.13e-06
LASP1	3927	LIM and SH3 protein 1	9.56	10.54	6.66e-04	4.58e-03
LAT	27040	linker for activation of T cells	6.63	8.54	1.14e-04	1.09e-03
LAT2	7462	linker for activation of T cells family, member 2	6.44	9.36	3.53e-04	2.70e-03
LAYN	143903	layilin	8.23	6.75	1.80e-06	3.42e-05
LBH	81606	hypothetical protein DKFZp566J091	9.87	8.77	2.25e-03	1.21e-02
LBR	3930	lamin B receptor	10.04	10.77	7.52e-03	3.06e-02
LCAT	3931	lecithin-cholesterol acyltransferase	6.27	5.49	4.13e-03	1.95e-02
LCHN	57189	LCHN protein	8.10	8.90	1.36e-02	4.84e-02
LCP1	3936	lymphocyte cytosolic protein 1 (L-plastin)	9.73	12.24	7.74e-08	2.44e-06
LCP2	3937	lymphocyte cytosolic protein 2 (SH2 domain contain	7.27	9.63	6.41e-09	2.90e-07
LDB2	9079	LIM domain binding 2	7.76	9.01	5.29e-05	5.77e-04
LDB3	11155	LIM domain binding 3	7.00	4.48	1.27e-15	4.08e-13
LDHB	3945	lactate dehydrogenase B	11.88	11.18	2.28e-03	1.22e-02
LDOC1	23641	leucine zipper, down-regulated in cancer 1	10.96	7.97	5.93e-11	5.65e-09
LEFTY2	7044	left-right determination factor 2	7.36	5.34	6.05e-09	2.77e-07
LEP	3952	leptin (obesity homolog, mouse)	5.30	7.41	4.07e-04	3.03e-03
LEPREL2	10536	leprecan-like 2	10.72	9.50	1.28e-04	1.19e-03
LEPROT	54741	leptin receptor overlapping transcript	10.16	9.51	4.42e-03	2.05e-02
LETMD1	25875	LETM1 domain containing 1	9.54	8.88	3.82e-03	1.84e-02
LGALS2	3957	lectin, galactoside-binding, soluble, 2 (galectin	4.77	7.44	1.32e-02	4.72e-02
LGALS8	3964	lectin, galactoside-binding, soluble, 8 (galectin	8.40	9.09	1.06e-02	3.99e-02
LGALS9	3965	lectin, galactoside-binding, soluble, 9 (galectin	5.40	6.70	2.44e-04	2.02e-03
LGI1	9211	leucine-rich, glioma inactivated 1	6.54	4.80	1.45e-05	1.96e-04
LGMN	5641	legumain	10.24	12.17	5.06e-04	3.65e-03
LGP2	79132	likely ortholog of mouse D11lgp2	7.55	8.37	3.47e-03	1.70e-02
LGR4	55366	leucine-rich repeat-containing G protein-coupled r	7.77	5.91	2.09e-05	2.66e-04
LGR6	59352	leucine-rich repeat-containing G protein-coupled r	7.46	5.09	1.16e-14	2.79e-12
LHCGR	3973	luteinizing hormone/choriogonadotropin receptor	6.99	4.89	1.65e-08	6.67e-07
LHFPL2	10184	lipoma HMGIC fusion partner-like 2	8.82	10.36	2.27e-03	1.22e-02

Symbol	GeneID	Gene Name*	Signal CNTRL	AAA	P	FDR
LIF	3976	leukemia inhibitory factor (cholinergic differenti	4.43	6.54	1.20e-04	1.13e-03
LIG1	3978	ligase I, DNA, ATP-dependent	6.04	7.12	6.16e-04	4.30e-03
LILRA2	11027	leukocyte immunoglobulin-like receptor, subfamily	5.80	8.14	8.87e-03	3.47e-02
LILRA5	353514	leukocyte immunoglobulin-like receptor, subfamily	7.54	9.05	1.47e-03	8.57e-03
LILRB1	10859	leukocyte immunoglobulin-like receptor, subfamily	5.87	6.63	1.27e-02	4.59e-02
LILRB2	10288	leukocyte immunoglobulin-like receptor, subfamily	8.28	10.64	3.42e-04	2.64e-03
LIMD2	80774	LIM domain containing 2	4.68	5.65	6.13e-03	2.61e-02
LIME1	54923	Lck interacting transmembrane adaptor 1	6.94	9.26	2.12e-03	1.15e-02
LIMK1	3984	LIM domain kinase 1	8.48	10.09	1.76e-06	3.37e-05
LIMS2	55679	LIM and senescent cell antigen-like domains 2	11.02	9.19	1.55e-09	8.93e-08
LIMS3	96626	LIM and senescent cell antigen-like domains 3	7.07	4.12	1.23e-05	1.72e-04
LIPA	3988	lipase A, lysosomal acid, cholesterol esterase (Wo	10.18	12.07	3.11e-03	1.56e-02
LITAF	9516	lipopolysaccharide-induced TNF factor	10.17	11.05	9.97e-03	3.81e-02
LIX1L	128077	Lix1 homolog (mouse) like	7.64	6.96	3.14e-03	1.57e-02
LLGL2	3993	lethal giant larvae homolog 2 (Drosophila)	6.43	7.24	1.22e-02	4.46e-02
LMBR1	64327	limb region 1 homolog (mouse)	8.17	7.52	4.33e-03	2.02e-02
LMCD1	29995	LIM and cysteine-rich domains 1	11.95	9.62	1.54e-04	1.39e-03
LMNB1	4001	lamin B1	4.37	6.35	1.48e-04	1.35e-03
LMNB2	84823	lamin B2	6.70	7.53	4.93e-03	2.22e-02
LMO3	55885	LIM domain only 3 (rhombotin-like 2)	9.89	6.98	6.45e-18	4.13e-15
LMO7	4008	LIM domain 7	6.09	4.71	1.12e-04	1.07e-03
LMOD1	25802	leiomodin 1 (smooth muscle)	10.48	7.20	5.12e-11	5.01e-09
LNK	10019	lymphocyte adaptor protein	8.17	9.46	5.15e-05	5.64e-04
LNPEP	4012	leucyl/cystinyl aminopeptidase	5.16	6.04	8.30e-03	3.31e-02
LNX2	222484	ligand of numb-protein X 2	7.27	6.34	3.02e-03	1.52e-02
LOC113179	113179	hypothetical protein BC011824	5.44	6.36	2.74e-03	1.41e-02
LOC116236	116236	hypothetical protein LOC116236	6.08	7.27	3.89e-03	1.86e-02
LOC116412	116412	hypothetical protein BC012365	6.04	7.00	4.66e-03	2.13e-02
LOC119710	119710	HEPIS	6.72	5.90	3.59e-03	1.74e-02
LOC120376	120376	hypothetical protein LOC120376	7.43	6.04	7.21e-04	4.90e-03
LOC123688	123688	similar to RIKEN cDNA C630028N24 gene	6.92	5.47	2.97e-07	7.48e-06
LOC124220	124220	similar to common salivary protein 1	4.58	6.22	3.08e-03	1.55e-02
LOC129607	129607	hypothetical protein LOC129607	6.30	7.55	5.68e-03	2.47e-02
LOC134147	134147	similar to mouse 2310016A09Rik gene	8.66	7.03	3.22e-11	3.29e-09
LOC134466	134466	hypothetical protein LOC134466	7.09	5.81	1.61e-07	4.54e-06
LOC144363	144363	hypothetical protein LOC144363	8.94	8.18	7.04e-04	4.80e-03
LOC145474	145474	hypothetical protein LOC145474	4.78	6.74	1.03e-02	3.89e-02
LOC145820	145820	hypothetical protein LOC145820	6.07	5.25	1.35e-02	4.79e-02
LOC147727	147727	hypothetical protein LOC147727	7.96	7.00	7.35e-04	4.98e-03
LOC148898	148898	hypothetical protein BC007899	6.01	4.96	7.82e-05	7.98e-04
LOC149832	149832	hypothetical protein LOC149832	7.35	6.72	6.28e-03	2.66e-02
LOC150223	150223	hypothetical protein LOC150223	7.07	7.76	1.10e-02	4.11e-02
LOC151534	151534	hypothetical protein BC009264	4.16	5.36	1.35e-02	4.81e-02
LOC152485	152485	hypothetical protein LOC152485	7.22	6.21	1.05e-03	6.59e-03
LOC157562	157562	hypothetical protein LOC157562	5.75	4.88	9.83e-03	3.77e-02
LOC158376	158376	hypothetical protein LOC158376	6.63	5.54	2.86e-03	1.46e-02
LOC158830	158830	similar to Ab2-183	3.84	6.75	3.48e-04	2.68e-03
LOC168850	168850	hypothetical protein LOC168850	7.28	8.01	7.44e-03	3.04e-02
LOC196394	196394	hypothetical protein LOC196394	6.97	6.06	6.90e-04	4.72e-03
LOC201175	201175	hypothetical protein LOC201175	5.42	6.90	2.42e-03	1.28e-02
LOC201484	201484	hypothetical LOC201484	6.82	5.15	2.31e-10	1.82e-08
LOC221091	221091	similar to hypothetical protein	7.63	6.22	6.89e-05	7.17e-04
LOC221955	221955	KCCR13L	7.99	9.14	5.53e-03	2.42e-02
LOC254128	254128	hypothetical protein LOC254128	7.61	6.66	8.50e-05	8.52e-04
LOC255374	255374	similar to hypothetical protein MGC49416	8.48	9.60	5.81e-03	2.51e-02
LOC255783	255783	hypothetical protein LOC255783	9.59	8.77	1.13e-02	4.20e-02
LOC283130	283130	hypothetical protein LOC283130	5.47	7.62	3.42e-08	1.23e-06
LOC283439	283439	hypothetical protein LOC283439	5.76	4.53	5.01e-03	2.24e-02
LOC283663	283663	hypothetical protein LOC283663	5.75	9.34	8.70e-03	3.43e-02
LOC284454	284454	hypothetical protein LOC284454	7.44	9.33	1.21e-04	1.14e-03
LOC285535	285535	hypothetical protein LOC285535	7.28	5.68	3.17e-03	1.58e-02
LOC285958	285958	hypothetical protein LOC285958	7.54	8.30	1.29e-02	4.63e-02
LOC285989	285989	hypothetical protein LOC285989	4.85	6.47	3.69e-06	6.20e-05
LOC286126	286126	hypothetical protein LOC286126	8.37	9.49	1.22e-02	4.46e-02
LOC286208	286208	hypothetical protein LOC286208	4.74	5.97	2.18e-03	1.18e-02
LOC286437	286437	hypothetical protein LOC286437	7.14	6.13	1.21e-04	1.13e-03
LOC286440	286440	hypothetical protein LOC286440	8.36	7.19	1.18e-04	1.12e-03

Symbol	GeneID	Gene Name*	Signal CNTRL	AAA	P	FDR
LOC339524	339524	hypothetical protein LOC339524	6.49	5.57	1.96e-04	1.68e-03
LOC339745	339745	hypothetical protein LOC339745	7.97	7.26	4.47e-03	2.07e-02
LOC340109	340109	hypothetical protein LOC340109	5.46	4.46	3.95e-03	1.88e-02
LOC344405	344405	similar to RIKEN cDNA 2010316F05	6.11	5.47	1.02e-02	3.87e-02
LOC346887	346887	similar to solute carrier family 16 (monocarboxyli	7.86	6.20	2.28e-06	4.20e-05
LOC349236	349236	hypothetical protein LOC349236	5.58	7.55	1.51e-04	1.37e-03
LOC387648	387648	similar to supervillin isoform 2; membrane-associa	8.57	6.89	3.99e-04	2.98e-03
LOC387758	387758	similar to RIKEN cDNA 1110018M03	8.19	4.95	5.60e-09	2.63e-07
LOC387763	387763	hypothetical LOC387763	13.55	12.17	2.07e-04	1.75e-03
LOC388489	388489	hypothetical gene supported by AK091795	4.87	6.27	9.63e-03	3.71e-02
LOC388564	388564	hypothetical gene supported by BC052596	6.69	7.59	9.23e-03	3.57e-02
LOC388969	388969	hypothetical LOC388969	6.35	7.82	5.56e-04	3.94e-03
LOC389289	389289	similar to annexin II receptor	5.74	7.07	4.80e-03	2.17e-02
LOC389641	389641	hypothetical gene supported by AK124295	4.33	6.33	2.10e-04	1.78e-03
LOC399818	399818	similar to CG9643-PA	6.27	5.30	3.90e-03	1.86e-02
LOC399959	399959	hypothetical gene supported by BX647608	10.55	8.09	1.53e-09	8.93e-08
LOC400027	400027	hypothetical gene supported by BC047417	8.94	8.18	7.74e-04	5.18e-03
LOC400043	400043	hypothetical gene supported by BC009385	8.68	6.72	4.91e-11	4.84e-09
LOC401048	401048	hypothetical LOC401048	7.11	5.46	7.29e-10	4.84e-08
LOC401093	401093	hypothetical LOC401093	9.95	7.16	3.30e-04	2.57e-03
LOC401233	401233	similar to HIV TAT specific factor 1; cofactor req	4.94	6.60	1.11e-03	6.88e-03
LOC401237	401237	hypothetical gene supported by AK026189	6.23	5.23	1.22e-03	7.39e-03
LOC51252	51252	hypothetical protein LOC51252	5.50	4.64	8.73e-03	3.44e-02
LOC51334	51334	mesenchymal stem cell protein DSC54	7.66	6.07	1.02e-09	6.40e-08
LOC56901	56901	NADH:ubiquinone oxidoreductase MLRQ subunit hom.	8.96	7.71	1.29e-02	4.64e-02
LOC57146	57146	promethin	9.74	8.07	3.26e-06	5.64e-05
LOC57149	57149	hypothetical protein A-211C6.1	9.72	8.95	7.54e-04	5.07e-03
LOC63920	63920	transposon-derived Buster3 transposase-like	7.50	5.43	1.92e-10	1.56e-08
LOC92249	92249	hypothetical protein LOC92249	6.43	5.21	1.13e-02	4.19e-02
LOC92270	92270	hypothetical protein LOC92270	5.91	4.98	1.10e-03	6.79e-03
LOC93349	93349	hypothetical protein BC004921	7.72	8.53	3.65e-03	1.77e-02
LOC93622	93622	hypothetical protein BC006130	7.29	6.32	3.96e-05	4.49e-04
LONRF3	79836	LON peptidase N-terminal domain and ring finger 3	4.79	6.56	1.04e-02	3.94e-02
LOX	4015	lysyl oxidase	8.15	6.67	1.14e-04	1.09e-03
LOXL1	4016	lysyl oxidase-like 1	8.94	8.01	3.26e-03	1.61e-02
LOXL4	84171	lysyl oxidase-like 4	8.62	7.34	1.01e-05	1.45e-04
LPHN2	23266	latrophilin 2	8.98	7.40	1.09e-09	6.70e-08
LPHN3	23284	latrophilin 3	8.09	6.30	8.27e-10	5.39e-08
LPP	4026	LIM domain containing preferred translocation part	10.30	7.56	2.19e-06	4.06e-05
LPXN	9404	leupaxin	8.30	10.58	1.34e-08	5.49e-07
LR8	28959	LR8 protein	7.18	8.59	1.64e-03	9.33e-03
LRCH2	57631	leucine-rich repeats and calponin homology (CH) do	8.51	6.15	1.65e-14	3.89e-12
LRCH4	4034	leucine-rich repeats and calponin homology (CH) do	6.09	7.82	2.79e-03	1.43e-02
LRFN3	79414	leucine rich repeat and fibronectin type III domai	7.42	6.36	1.39e-04	1.27e-03
LRIG1	26018	leucine-rich repeats and immunoglobulin-like domai	8.46	6.95	9.34e-08	2.89e-06
LRMP	4033	lymphoid-restricted membrane protein	6.07	8.12	1.34e-05	1.85e-04
LRP10	26020	low density lipoprotein receptor-related protein 1	11.01	10.27	1.09e-03	6.74e-03
LRP11	84918	low density lipoprotein receptor-related protein 1	7.60	6.59	3.05e-03	1.54e-02
LRP2BP	55805	LRP2 binding protein	9.78	7.12	4.39e-15	1.18e-12
LRP5	4041	low density lipoprotein receptor-related protein 5	8.13	6.52	3.36e-07	8.27e-06
LRRC1	55227	leucine rich repeat containing 1	7.91	6.31	3.52e-07	8.61e-06
LRRC17	10234	leucine rich repeat containing 17	10.01	8.26	1.22e-03	7.35e-03
LRRC25	126364	leucine rich repeat containing 25	5.47	6.77	3.71e-03	1.79e-02
LRRC28	123355	leucine rich repeat containing 28	6.48	5.81	1.01e-02	3.85e-02
LRRC32	2615	leucine rich repeat containing 32	9.77	8.37	1.10e-04	1.06e-03
LRRC33	375387	leucine rich repeat containing 33	5.18	7.35	9.19e-07	1.96e-05
LRRC45	201255	leucine rich repeat containing 45	5.32	6.40	1.36e-03	8.01e-03
LRRC49	54839	leucine rich repeat containing 49	6.80	4.93	1.24e-10	1.08e-08
LRRC51	220074	leucine rich repeat containing 51	6.21	5.42	1.58e-03	9.08e-03
LRRC54	25987	leucine rich repeat containing 54	7.21	6.17	3.25e-05	3.80e-04
LRRC8A	56262	leucine rich repeat containing 8 family, member A	7.17	6.02	3.37e-06	5.80e-05
LRRC8C	84230	leucine rich repeat containing 8 family, member C	7.67	8.58	5.98e-03	2.56e-02
LRRC8D	55144	leucine rich repeat containing 8 family, member D	6.88	8.28	1.24e-05	1.73e-04
LRRCC1	85444	leucine rich repeat and coiled-coil domain contain	7.69	7.07	9.37e-03	3.62e-02
LRRFIP2	9209	leucine rich repeat (in FLII) interacting protein	9.00	7.40	2.83e-10	2.18e-08
LSP1	4046	lymphocyte-specific protein 1	7.03	9.29	3.52e-06	5.99e-05
LTB	4050	lymphotoxin beta (TNF superfamily, member 3)	5.82	10.26	2.95e-03	1.50e-02

Symbol	GeneID	Gene Name*	Signal CNTRL	AAA	P	FDR
LTBP1	4052	latent transforming growth factor beta binding pro	12.15	9.33	1.41e-15	4.36e-13
LTBP2	4053	latent transforming growth factor beta binding pro	11.01	8.79	1.35e-05	1.85e-04
LTBP3	4054	latent transforming growth factor beta binding pro	9.22	7.87	2.85e-03	1.46e-02
LTBP4	8425	latent transforming growth factor beta binding pro	10.00	8.72	1.30e-03	7.77e-03
LTC4S	4056	leukotriene C4 synthase	8.17	9.03	6.16e-03	2.62e-02
LTF	4057	lactotransferrin	6.10	9.64	2.79e-04	2.24e-03
LUC7L	55692	LUC7-like (S. cerevisiae)	5.57	6.59	1.00e-02	3.83e-02
LW-1	51402	LW-1	6.13	5.17	2.00e-04	1.70e-03
LY86	9450	lymphocyte antigen 86	7.68	10.00	8.59e-07	1.85e-05
LY9	4063	lymphocyte antigen 9	4.29	7.41	5.44e-03	2.39e-02
LY96	23643	lymphocyte antigen 96	8.00	9.11	6.39e-04	4.42e-03
LYL1	4066	lymphoblastic leukemia derived sequence 1	8.81	10.38	6.46e-05	6.81e-04
LYN	4067	v-yes-1 Yamaguchi sarcoma viral related oncogene h	8.69	10.83	1.41e-07	4.08e-06
LYNX1	66004	Ly6/neurotoxin 1	6.57	3.76	7.47e-10	4.93e-08
LYPLA2	11313	lysophospholipase II	6.97	7.94	4.72e-03	2.14e-02
LYPLA3	23659	lysophospholipase 3 (lysosomal phospholipase A2)	7.35	8.63	4.79e-04	3.48e-03
LYSMD1	388695	LysM, putative peptidoglycan-binding, domain conta	5.74	4.81	1.84e-03	1.02e-02
LYSMD4	145748	LysM, putative peptidoglycan-binding, domain conta	6.83	5.53	1.05e-06	2.19e-05
LYZ	4069	lysozyme (renal amyloidosis)	8.05	11.27	1.19e-06	2.44e-05
M6PR	4074	mannose-6-phosphate receptor (cation dependent)	9.12	10.24	1.68e-04	1.49e-03
MACF1	23499	microtubule-actin crosslinking factor 1	9.72	8.94	1.26e-03	7.56e-03
MAD1L1	8379	MAD1 mitotic arrest deficient-like 1 (yeast)	5.72	7.00	9.51e-05	9.32e-04
MAD2L1BP	9587	MAD2L1 binding protein	8.06	9.00	9.90e-04	6.28e-03
MAF	4094	v-maf musculoaponeurotic fibrosarcoma oncogene hom	8.14	9.65	1.39e-05	1.90e-04
MAFB	9935	v-maf musculoaponeurotic fibrosarcoma oncogene hom	8.92	10.29	3.40e-04	2.63e-03
MAGED2	10916	melanoma antigen family D, 2	5.74	4.23	2.28e-03	1.22e-02
MAGEH1	28986	melanoma antigen family H, 1	6.49	5.30	9.52e-04	6.10e-03
MAGI1	9223	membrane associated guanylate kinase, WW and PDZ d	8.84	7.67	2.36e-03	1.25e-02
MAGI2	9863	membrane associated guanylate kinase, WW and PDZ d	7.27	5.76	4.65e-06	7.53e-05
MALT1	10892	mucosa associated lymphoid tissue lymphoma translo	7.01	7.93	2.23e-03	1.20e-02
MAMDC2	256691	MAM domain containing 2	9.39	7.66	1.66e-05	2.18e-04
MAN2B1	4125	mannosidase, alpha, class 2B, member 1	6.72	8.08	1.88e-05	2.43e-04
MANBAL	63905	mannosidase, beta A, lysosomal-like	9.98	9.09	1.56e-03	8.95e-03
MANSC1	54682	MANSC domain containing 1	7.63	6.24	3.08e-08	1.12e-06
MAOA	4128	monoamine oxidase A	11.51	8.53	6.48e-14	1.25e-11
MAOB	4129	monoamine oxidase B	10.15	8.13	1.74e-10	1.46e-08
MAP1B	4131	microtubule-associated protein 1B	9.23	6.69	8.65e-12	1.02e-09
MAP2	4133	microtubule-associated protein 2	6.79	4.87	3.76e-03	1.81e-02
MAP2K2	5605	mitogen-activated protein kinase kinase 2	10.33	11.13	3.56e-03	1.73e-02
MAP2K3	5606	mitogen-activated protein kinase kinase 3	4.85	6.28	1.22e-03	7.36e-03
MAP3K1	4214	mitogen-activated protein kinase kinase kinase 1	8.28	9.69	2.73e-04	2.21e-03
MAP3K11	4296	mitogen-activated protein kinase kinase kinase 11	6.82	7.71	2.94e-03	1.49e-02
MAP3K12	7786	mitogen-activated protein kinase kinase kinase 12	6.23	4.99	8.59e-04	5.65e-03
MAP3K14	9020	mitogen-activated protein kinase kinase kinase 14	6.33	8.07	4.44e-06	7.26e-05
MAP3K5	4217	mitogen-activated protein kinase kinase kinase 5	9.00	8.02	1.45e-04	1.32e-03
MAP3K6	9064	mitogen-activated protein kinase kinase kinase 6	9.41	8.44	5.81e-05	6.22e-04
MAP3K8	1326	mitogen-activated protein kinase kinase kinase 8	8.31	9.77	2.87e-04	2.29e-03
MAP4	4134	microtubule-associated protein 4	7.02	5.38	2.91e-04	2.31e-03
MAP4K1	11184	mitogen-activated protein kinase kinase kinase kin	7.45	9.11	1.48e-03	8.59e-03
MAP4K2	5871	mitogen-activated protein kinase kinase kinase kin	7.91	9.37	4.14e-04	3.07e-03
MAP4K4	9448	mitogen-activated protein kinase kinase kinase kin	7.93	7.15	6.01e-03	2.57e-02
MAPK1	5594	mitogen-activated protein kinase 1	6.53	7.64	3.19e-04	2.50e-03
MAPK13	5603	mitogen-activated protein kinase 13	5.23	8.26	1.46e-06	2.88e-05
MAPK8IP3	23162	mitogen-activated protein kinase 8 interacting pro	8.37	9.31	1.12e-02	4.18e-02
MAPKAP1	79109	mitogen-activated protein kinase associated protei	8.36	9.20	2.64e-03	1.37e-02
MAPKAPK2	9261	mitogen-activated protein kinase-activated protein	8.25	9.03	4.46e-03	2.06e-02
MAPKAPK3	7867	mitogen-activated protein kinase-activated protein	7.27	9.45	1.27e-05	1.76e-04
MARCH1	55016	membrane-associated ring finger (C3HC4) 1	6.36	8.04	5.66e-03	2.46e-02
MARCKS	4082	myristoylated alanine-rich protein kinase C substr	5.09	7.28	3.61e-08	1.29e-06
MARCKSL1	65108	MARCKS-like 1	6.91	8.08	7.37e-03	3.02e-02
MARCO	8685	macrophage receptor with collagenous structure	8.06	9.71	1.24e-02	4.51e-02
MARK1	4139	MAP/microtubule affinity-regulating kinase 1	6.17	5.26	3.98e-04	2.98e-03
MARK2	2011	MAP/microtubule affinity-regulating kinase 2	9.26	10.06	4.66e-03	2.13e-02
MARVELD1	83742	MARVEL domain containing 1	9.43	8.73	5.82e-03	2.51e-02
MAT2A	4144	methionine adenosyltransferase II, alpha	9.43	10.80	1.44e-03	8.42e-03
MATK	4145	megakaryocyte-associated tyrosine kinase	4.97	7.09	1.09e-02	4.08e-02
MATR3	9782	matrin 3	11.17	10.60	1.17e-02	4.30e-02

Symbol	GeneID	Gene Name*	Signal CNTRL	AAA	P	FDR
MBIP	51562	MAP3K12 binding inhibitory protein 1	8.50	7.81	2.25e-03	1.21e-02
MBNL1	4154	muscleblind-like (Drosophila)	10.52	9.74	6.15e-03	2.62e-02
MBNL2	10150	muscleblind-like 2 (Drosophila)	10.47	9.26	1.62e-03	9.21e-03
MCFD2	90411	multiple coagulation factor deficiency 2	9.42	8.76	3.96e-03	1.88e-02
MCL1	4170	myeloid cell leukemia sequence 1 (BCL2-related)	7.29	8.43	7.66e-04	5.13e-03
MCM2	4171	MCM2 minichromosome maintenance deficient 2, mitot	6.98	8.43	1.44e-05	1.95e-04
MCM5	4174	MCM5 minichromosome maintenance deficient 5, cell	7.07	8.54	6.72e-05	7.03e-04
MCOLN1	57192	mucolipin 1	8.17	9.28	1.30e-03	7.77e-03
MCOLN2	255231	mucolipin 2	4.55	5.68	1.18e-02	4.33e-02
MDC1	9656	mediator of DNA damage checkpoint 1	7.26	6.45	3.04e-03	1.53e-02
ME2	4200	malic enzyme 2, NAD(+)-dependent, mitochondrial	7.58	8.99	8.02e-03	3.22e-02
MEIS1	4211	Meis1, myeloid ecotropic viral integration site 1	8.28	6.81	8.58e-07	1.85e-05
MEIS2	4212	Meis1, myeloid ecotropic viral integration site 1	8.58	7.11	9.26e-07	1.96e-05
MEOX2	4223	mesenchyme homeobox 2	8.49	6.95	6.08e-03	2.60e-02
MESDC1	59274	mesoderm development candidate 1	6.10	7.18	3.40e-04	2.63e-03
METAP1	23173	methionyl aminopeptidase 1	8.53	7.74	2.97e-03	1.50e-02
METT5D1	196074	methyltransferase 5 domain containing 1	6.64	5.79	3.02e-03	1.52e-02
METTL5	29081	methyltransferase like 5	8.26	7.62	4.87e-03	2.20e-02
METTL7A	25840	methyltransferase like 7A	11.79	10.69	1.61e-06	3.13e-05
MFAP2	4237	microfibrillar-associated protein 2	9.35	8.51	8.20e-03	3.28e-02
MFAP4	4239	microfibrillar-associated protein 4	12.28	10.24	8.96e-06	1.31e-04
MFGE8	4240	milk fat globule-EGF factor 8 protein	13.93	11.90	1.19e-09	7.21e-08
MFNG	4242	manic fringe homolog (Drosophila)	7.03	9.03	3.28e-07	8.15e-06
MFSD1	64747	major facilitator superfamily domain containing 1	9.69	10.73	2.10e-03	1.14e-02
MFSD3	113655	major facilitator superfamily domain containing 3	6.22	7.38	4.83e-04	3.51e-03
MFSD5	84975	major facilitator superfamily domain containing 5	6.54	7.77	7.78e-05	7.95e-04
MFSD7	84179	major facilitator superfamily domain containing 7	8.39	9.88	1.09e-04	1.05e-03
MGAT1	4245	mannosyl (alpha-1,3-)-glycoprotein beta-1,2-N-acet	8.64	9.65	2.85e-03	1.46e-02
MGAT4B	11282	mannosyl (alpha-1,3-)-glycoprotein beta-1,4-N-acet	7.80	8.89	2.29e-04	1.91e-03
MGC10992	92922	hypothetical protein LOC92922	8.26	6.94	5.58e-06	8.82e-05
MGC14376	84981	hypothetical protein MGC14376	8.23	9.89	7.33e-04	4.97e-03
MGC15416	84331	hypothetical protein MGC15416	5.74	7.45	5.47e-03	2.39e-02
MGC15476	147906	thymus expressed gene 3-like	8.82	6.15	2.48e-14	5.31e-12
MGC15619	84329	hypothetical protein MGC15619	6.57	8.35	1.12e-04	1.07e-03
MGC16028	112752	MGC16028 similar to RIKEN cDNA 1700019E19 gene	7.71	7.00	7.19e-03	2.96e-02
MGC16169	93627	hypothetical protein MGC16169	8.91	7.85	5.59e-05	6.04e-04
MGC18216	145815	hypothetical protein MGC18216	8.10	7.13	2.62e-03	1.37e-02
MGC19764	162394	likely ortholog of mouse schlafen 5	6.74	7.58	2.81e-03	1.44e-02
MGC20255	90324	hypothetical protein MGC20255	7.25	7.92	1.25e-02	4.53e-02
MGC20983	115948	hypothetical protein MGC20983	7.25	6.64	8.94e-03	3.49e-02
MGC21881	389741	hypothetical protein MGC21881	7.12	6.41	9.51e-03	3.66e-02
MGC23244	126259	hypothetical protein MGC23244	4.65	5.63	2.68e-03	1.39e-02
MGC24665	116028	hypothetical protein MGC24665	5.05	6.19	7.43e-04	5.02e-03
MGC24975	163154	hypothetical protein MGC24975	4.99	6.33	6.84e-05	7.12e-04
MGC26885	124044	hypothetical protein MGC26885	7.06	8.51	1.73e-05	2.26e-04
MGC31967	203260	hypothetical protein MGC31967	8.63	7.25	5.86e-09	2.72e-07
MGC33846	220382	hypothetical protein MGC33846	6.59	5.53	4.45e-05	4.96e-04
MGC3731	79159	hypothetical protein MGC3731	5.73	6.94	9.07e-04	5.87e-03
MGC39821	284440	hypothetical protein MGC39821	7.26	6.41	2.79e-04	2.24e-03
MGC39900	286527	hypothetical protein MGC39900	6.46	5.10	3.90e-05	4.44e-04
MGC40405	257415	hypothetical protein MGC40405	7.21	6.41	6.37e-04	4.42e-03
MGC4399	84275	PNC1 protein	7.27	8.17	1.50e-03	8.68e-03
MGC4618	84286	hypothetical protein MGC4618	8.03	8.71	1.25e-02	4.54e-02
MGC72075	340277	hypothetical protein MGC72075	5.81	4.61	9.18e-03	3.56e-02
MGC9913	386759	hypothetical protein MGC9913	9.30	8.11	2.21e-05	2.78e-04
MGEA5	10724	meningioma expressed antigen 5 (hyaluronidase)	10.76	10.05	1.06e-02	3.98e-02
MGP	4256	matrix Gla protein	14.75	12.83	1.90e-10	1.55e-08
MGST3	4259	microsomal glutathione S-transferase 3	11.70	10.99	2.05e-03	1.12e-02
MIB1	57534	mindbomb homolog 1 (Drosophila)	6.29	5.53	8.10e-03	3.24e-02
MICA	4276	MHC class I polypeptide-related sequence A	9.45	8.28	3.89e-07	9.38e-06
MICAL1	64780	microtubule associated monoxygenase, calponin and	8.86	9.77	1.30e-03	7.75e-03
MICB	4277	MHC class I polypeptide-related sequence B	6.40	7.55	1.62e-04	1.45e-03
MID1	4281	midline 1 (Opitz/BBB syndrome)	7.76	5.97	6.46e-06	9.94e-05
MID2	11043	midline 2	6.57	5.05	1.51e-05	2.03e-04
MIER1	57708	mesoderm induction early response 1 homolog (Xenop	9.59	8.74	1.83e-04	1.58e-03
mimitin	91942	Myc-induced mitochondria protein	7.88	7.12	9.55e-04	6.11e-03
MIPOL1	145282	mirror-image polydactyly 1	6.32	4.94	6.95e-07	1.56e-05

Symbol	GeneID	Gene Name*	Signal CNTRL	AAA	P	FDR
MLSTD1	55711	male sterility domain containing 1	5.53	6.55	1.23e-03	7.40e-03
MLYCD	23417	malonyl-CoA decarboxylase	8.74	7.94	4.93e-03	2.22e-02
MMD	23531	monocyte to macrophage differentiation-associated	8.14	9.54	1.18e-03	7.19e-03
MME	4311	membrane metallo-endopeptidase (neutral endopeptid	5.71	7.63	1.30e-02	4.66e-02
MMP1	4312	matrix metallopeptidase 1 (interstitial collagenas	5.47	8.64	1.16e-02	4.29e-02
MMP17	4326	matrix metallopeptidase 17 (membrane-inserted)	5.80	6.81	2.17e-03	1.17e-02
MMP19	4327	matrix metallopeptidase 19	7.99	9.89	6.86e-03	2.85e-02
MMP25	64386	matrix metallopeptidase 25	5.11	8.28	1.19e-03	7.24e-03
MMP9	4318	matrix metallopeptidase 9 (gelatinase B, 92kDa gel	7.62	12.31	1.31e-02	4.68e-02
MN1	4330	meningioma (disrupted in balanced translocation) 1	7.54	5.51	3.14e-05	3.70e-04
MNAT1	4331	menage a trois homolog 1, cyclin H assembly factor	7.81	6.80	2.41e-05	2.99e-04
MNDA	4332	myeloid cell nuclear differentiation antigen	7.85	10.44	4.28e-07	1.01e-05
MOBKL2A	126308	MOB1, Mps One Binder kinase activator-like 2A (yea	7.23	8.61	7.75e-05	7.93e-04
MOBKL2B	79817	MOB1, Mps One Binder kinase activator-like 2B (yea	8.21	7.10	6.27e-06	9.70e-05
MOCS1	4337	molybdenum cofactor synthesis 1	6.51	5.29	4.54e-03	2.09e-02
MOCS2	4338	molybdenum cofactor synthesis 2	7.47	6.70	5.86e-03	2.52e-02
MORC2	22880	MORC family CW-type zinc finger 2	8.55	7.81	1.19e-03	7.24e-03
MORC4	79710	MORC family CW-type zinc finger 4	8.67	7.63	1.24e-04	1.16e-03
MORF4L2	9643	mortality factor 4 like 2	10.58	9.49	2.44e-05	3.02e-04
MPDU1	9526	mannose-P-dolichol utilization defect 1	6.80	7.92	1.06e-02	3.97e-02
MPDZ	8777	multiple PDZ domain protein	9.13	7.43	4.50e-05	5.00e-04
MPEG1	219972	macrophage expressed gene 1	6.87	9.80	4.37e-10	3.11e-08
MPL	4352	myeloproliferative leukemia virus oncogene	6.18	4.92	5.54e-06	8.79e-05
MPP1	4354	membrane protein, palmitoylated 1, 55kDa	8.79	10.42	7.50e-03	3.05e-02
MPP6	51678	membrane protein, palmitoylated 6 (MAGUK p55 subfa	6.40	4.66	2.27e-07	5.98e-06
MPPED2	744	metallophosphoesterase domain containing 2	7.80	6.15	1.75e-10	1.46e-08
MR-1	25953	myofibrillogenesis regulator 1	7.13	9.07	5.55e-05	6.01e-04
MRFAP1	93621	Mof4 family associated protein 1	11.34	10.59	7.32e-03	3.00e-02
MRGPRF	219928	MAS-related GPR, member F	9.40	7.66	4.72e-05	5.23e-04
M-RIP	23164	myosin phosphatase-Rho interacting protein	10.16	9.28	9.23e-04	5.95e-03
MRPL10	124995	mitochondrial ribosomal protein L10	9.78	8.99	5.29e-04	3.78e-03
MRPL33	9553	mitochondrial ribosomal protein L33	9.84	9.01	3.05e-04	2.40e-03
MRPL49	740	mitochondrial ribosomal protein L49	8.73	8.02	1.62e-03	9.21e-03
MRPS31	10240	mitochondrial ribosomal protein S31	6.00	4.56	8.55e-07	1.85e-05
MRPS6	64968	mitochondrial ribosomal protein S6	11.52	10.55	3.45e-03	1.69e-02
MRVI1	10335	murine retrovirus integration site 1 homolog	8.76	6.13	1.90e-04	1.63e-03
MS4A6A	64231	membrane-spanning 4-domains, subfamily A, member 6	9.24	10.96	1.16e-06	2.37e-05
MS4A7	58475	membrane-spanning 4-domains, subfamily A, member 7	7.94	9.37	1.30e-05	1.80e-04
MSC	9242	musculin (activated B-cell factor-1)	9.43	8.72	5.90e-03	2.54e-02
MSTO1	55154	misato homolog 1 (Drosophila)	6.99	7.77	4.59e-03	2.10e-02
MSX1	4487	msh homeobox homolog 1 (Drosophila)	8.51	7.65	2.99e-03	1.51e-02
MT1X	4501	metallothionein 1X	12.62	11.00	4.78e-04	3.48e-03
MTA3	57504	metastasis associated 1 family, member 3	8.04	6.65	5.94e-09	2.74e-07
MTAC2D1	123036	membrane targeting (tandem) C2 domain containing 1	8.18	5.84	6.30e-09	2.86e-07
MTCH2	23788	mitochondrial carrier homolog 2 (C. elegans)	8.28	8.98	9.52e-03	3.67e-02
MTF2	22823	metal response element binding transcription facto	7.82	8.64	3.16e-03	1.58e-02
MTFR1	9650	mitochondrial fission regulator 1	8.12	7.39	8.23e-03	3.29e-02
MTHFD2	10797	methylenetetrahydrofolate dehydrogenase (NADP+ dep	10.43	8.83	2.80e-05	3.39e-04
MTMR11	10903	myotubularin related protein 11	9.42	7.96	2.24e-08	8.68e-07
MTP18	51537	mitochondrial protein 18 kDa	7.56	8.66	3.69e-04	2.80e-03
MTSS1	9788	metastasis suppressor 1	6.63	7.66	5.20e-04	3.72e-03
MTUS1	57509	mitochondrial tumor suppressor 1	8.83	6.97	6.96e-11	6.43e-09
MUT	4594	methylmalonyl Coenzyme A mutase	8.77	8.04	1.30e-03	7.75e-03
MX2	4600	myxovirus (influenza virus) resistance 2 (mouse)	6.65	8.80	3.71e-08	1.31e-06
MXI1	4601	MAX interactor 1	9.10	7.42	7.07e-10	4.72e-08
MXRA7	439921	matrix-remodelling associated 7	11.79	10.31	1.82e-04	1.58e-03
MXRA8	54587	matrix-remodelling associated 8	9.07	8.00	6.16e-03	2.62e-02
MYBL2	4605	v-myb myeloblastosis viral oncogene homolog (avian	3.95	6.41	1.22e-02	4.46e-02
MYD88	4615	myeloid differentiation primary response gene (88)	7.31	8.83	4.22e-06	6.98e-05
MYH10	4628	myosin, heavy polypeptide 10, non-muscle	13.02	9.85	9.61e-17	4.82e-14
MYH11	4629	myosin, heavy polypeptide 11, smooth muscle	13.99	11.52	4.83e-13	7.54e-11
MYH9	4627	myosin, heavy polypeptide 9, non-muscle	11.65	10.96	1.18e-02	4.33e-02
MYL5	4636	myosin, light polypeptide 5, regulatory	5.53	6.70	1.32e-03	7.83e-03
MYL9	10398	myosin, light polypeptide 9, regulatory	12.30	9.89	2.70e-07	6.97e-06
MYLK	4638	myosin, light polypeptide kinase	9.96	7.60	5.15e-08	1.73e-06
MYO10	4651	myosin X	7.85	7.08	1.30e-02	4.66e-02
MYO18B	84700	myosin XVIIIB	7.88	5.51	1.07e-02	4.01e-02

Symbol	GeneID	Gene Name*	Signal CNTRL	AAA	P	FDR
MYO1B	4430	myosin IB	8.39	7.06	2.31e-07	6.04e-06
MYO1C	4641	myosin IC	6.24	4.89	3.96e-06	6.61e-05
MYO1F	4542	myosin IF	5.51	8.15	3.37e-03	1.65e-02
MYO1G	64005	myosin IG	4.67	7.72	2.28e-05	2.85e-04
MYO5A	4644	myosin VA (heavy polypeptide 12, myoxin)	7.86	9.16	6.94e-03	2.87e-02
MYO6	4646	myosin VI	7.54	6.51	1.26e-02	4.55e-02
MYO7A	4647	myosin VIIA	5.51	6.52	4.53e-03	2.09e-02
MYO9A	4649	myosin IXA	7.61	6.83	6.81e-04	4.68e-03
MYOC	4653	myocilin, trabecular meshwork inducible glucocorti	8.57	5.63	2.35e-09	1.26e-07
MYOM1	8736	myomesin 1 (skelemin) 185kDa	7.95	5.99	3.66e-10	2.69e-08
MYOZ1	58529	myozenin 1	7.19	5.73	4.31e-06	7.09e-05
MYOZ2	51778	myozenin 2	5.98	2.45	1.62e-03	9.22e-03
MYOZ3	91977	myozenin 3	6.01	4.57	6.54e-04	4.51e-03
MYST2	11143	MYST histone acetyltransferase 2	8.08	7.39	2.47e-03	1.30e-02
N4BP2	55728	Nedd4 binding protein 2	9.60	10.44	1.00e-02	3.83e-02
NAB1	4664	NGFI-A binding protein 1 (EGR1 binding protein 1)	10.00	9.15	1.85e-04	1.60e-03
NAG	51594	neuroblastoma-amplified protein	7.50	6.90	9.14e-03	3.55e-02
NAGA	4668	N-acetylgalactosaminidase, alpha-	6.98	7.92	1.09e-03	6.74e-03
NAGK	55577	N-acetylglucosamine kinase	8.65	10.06	1.39e-05	1.90e-04
NAGPA	51172	N-acetylglucosamine-1-phosphodiester alpha-N-acety	6.14	7.31	1.20e-04	1.13e-03
NALP2	55655	NACHT, leucine rich repeat and PYD containing 2	4.16	5.60	6.01e-03	2.57e-02
NAP1L2	4674	nucleosome assembly protein 1-like 2	6.48	5.07	6.07e-06	9.43e-05
NAP1L3	4675	nucleosome assembly protein 1-like 3	7.96	5.68	6.61e-10	4.46e-08
NAP1L5	266812	nucleosome assembly protein 1-like 5	8.38	6.82	1.76e-10	1.46e-08
NAPB	63908	N-ethylmaleimide-sensitive factor attachment prote	7.57	6.83	1.29e-03	7.72e-03
NAPE-PLD	222236	N-acyl-phosphatidylethanolamine-hydrolyzing phosph	6.62	5.15	3.09e-04	2.43e-03
NASP	4678	nuclear autoantigenic sperm protein (histone-bindi	6.17	6.90	8.98e-03	3.50e-02
NAT1	9	N-acetyltransferase 1 (arylamine N-acetyltransfera	5.59	6.56	1.34e-02	4.79e-02
NAV2	89797	neuron navigator 2	9.13	6.99	4.02e-06	6.69e-05
NBR1	4077	neighbor of BRCA1 gene 1	10.64	9.90	1.18e-03	7.20e-03
NCALD	83988	neurocalcin delta	7.64	5.92	3.23e-06	5.61e-05
NCF1	4687	neutrophil cytosolic factor 1, (chronic granulomat	8.32	11.46	4.66e-06	7.53e-05
NCF2	4688	neutrophil cytosolic factor 2 (65kDa, chronic gran	7.51	10.21	1.74e-03	9.77e-03
NCF4	4689	neutrophil cytosolic factor 4, 40kDa	8.69	10.76	5.36e-08	1.77e-06
NCKAP1	10787	NCK-associated protein 1	11.55	9.61	8.93e-13	1.36e-10
NCKAP1L	3071	NCK-associated protein 1-like	6.53	9.62	3.87e-09	1.89e-07
NCKIPSD	51517	NCK interacting protein with SH3 domain	7.82	6.80	4.35e-03	2.03e-02
NDE1	54820	nudE nuclear distribution gene E homolog 1 (A. nid	7.90	9.11	1.64e-03	9.31e-03
NDFIP1	80762	Nedd4 family interacting protein 1	8.30	7.35	3.42e-05	3.96e-04
NDN	4692	necdin homolog (mouse)	10.41	9.14	6.94e-06	1.05e-04
NDRG3	57446	NDRG family member 3	9.91	8.87	5.07e-06	8.12e-05
NDUFA4	4697	NADH dehydrogenase (ubiquinone) 1 alpha subcomplex	11.58	10.69	7.86e-05	8.02e-04
NDUFC2	4718	NADH dehydrogenase (ubiquinone) 1, subcomplex unkn	8.14	8.83	1.10e-02	4.10e-02
NDUFS4	4724	NADH dehydrogenase (ubiquinone) Fe-S protein 4, 18	9.55	8.93	6.52e-03	2.74e-02
NECAP2	55707	NECAP endocytosis associated 2	8.29	9.21	2.17e-03	1.17e-02
NEDD4	4734	neural precursor cell expressed, developmentally d	7.79	6.48	7.08e-08	2.26e-06
NEDD4L	23327	neural precursor cell expressed, developmentally d	5.11	5.87	1.15e-02	4.26e-02
NEDD9	4739	neural precursor cell expressed, developmentally d	9.01	5.51	1.00e-03	6.35e-03
NEGR1	257194	neuronal growth regulator 1	5.64	4.61	2.88e-03	1.46e-02
NEK1	4750	NIMA (never in mitosis gene a)-related kinase 1	7.71	7.05	6.08e-03	2.60e-02
NEK6	10783	NIMA (never in mitosis gene a)-related kinase 6	6.51	7.49	8.19e-04	5.43e-03
NENF	29937	neuron derived neurotrophic factor	8.44	7.61	2.62e-04	2.14e-03
NEO1	4756	neogenin homolog 1 (chicken)	7.23	5.56	3.54e-09	1.76e-07
NET1	10276	neuroepithelial cell transforming gene 1	11.64	9.57	1.52e-12	2.17e-10
NETO2	81831	neuropilin (NRP) and tolloid (TLL)-like 2	4.64	6.74	3.08e-03	1.55e-02
NEU1	4758	sialidase 1 (lysosomal sialidase)	9.55	10.25	9.42e-03	3.63e-02
NEXN	91624	nexilin (F actin binding protein)	10.09	7.63	8.78e-12	1.02e-09
NFATC1	4772	nuclear factor of activated T-cells, cytoplasmic,	7.40	9.00	3.30e-04	2.57e-03
NFATC3	4775	nuclear factor of activated T-cells, cytoplasmic,	4.96	5.76	1.03e-02	3.89e-02
NFE2L1	4779	nuclear factor (erythroid-derived 2)-like 1	8.68	8.01	9.13e-03	3.55e-02
NFE2L3	9603	nuclear factor (erythroid-derived 2)-like 3	6.16	7.84	3.22e-06	5.61e-05
NFIA	4774	nuclear factor I/A	10.84	8.57	1.93e-07	5.22e-06
NFIB	4781	nuclear factor I/B	10.23	9.23	8.50e-03	3.36e-02
NFIC	4782	nuclear factor I/C (CCAAT-binding transcription fa	5.66	4.48	3.69e-03	1.78e-02
NFKBIE	4794	nuclear factor of kappa light polypeptide gene enh	7.65	9.71	4.85e-08	1.64e-06
NFKBIZ	64332	nuclear factor of kappa light polypeptide gene enh	7.80	9.74	1.19e-07	3.52e-06
NFYB	4801	nuclear transcription factor Y, beta	7.31	6.27	9.77e-05	9.54e-04

Symbol	GeneID	Gene Name*	Signal CNTRL	AAA	P	FDR
NGFRAP1	27018	nerve growth factor receptor (TNFRSF16) associated	11.32	10.33	1.40e-05	1.91e-04
NGFRAP1L1	340542	NGFRAP1-like 1	7.11	6.31	1.94e-03	1.07e-02
NGLY1	55768	N-glycanase 1	8.70	9.46	5.85e-03	2.52e-02
NHS	4810	Nance-Horan syndrome (congenital cataracts and den	7.73	5.95	1.79e-03	1.00e-02
NID1	4811	nidogen 1	6.84	5.32	5.14e-04	3.69e-03
NIFUN	23479	NifU-like N-terminal domain containing	11.31	10.60	1.82e-03	1.02e-02
NINJ1	4814	ninjurin 1	10.66	11.93	3.87e-03	1.86e-02
NINJ2	4815	ninjurin 2	6.98	7.94	4.93e-03	2.22e-02
NIPA2	81614	non imprinted in Prader-Willi/Angelman syndrome 2	8.92	9.84	1.28e-03	7.68e-03
NIPSNAP3B	55335	nipsnap homolog 3B (C. elegans)	6.07	4.42	1.03e-06	2.16e-05
NKD1	85407	naked cuticle homolog 1 (Drosophila)	6.31	4.56	4.71e-07	1.11e-05
NKG7	4818	natural killer cell group 7 sequence	5.23	7.17	1.63e-04	1.46e-03
NKIRAS1	28512	NFKB inhibitor interacting Ras-like 1	7.38	6.44	7.09e-04	4.83e-03
NKX3-1	4824	NK3 transcription factor related, locus 1 (Drosoph	5.41	6.38	1.41e-02	4.99e-02
NLGN1	22871	neuroligin 1	7.91	4.75	4.00e-07	9.57e-06
NLK	51701	nemo-like kinase	5.86	6.68	7.19e-03	2.96e-02
NMD3	51068	NMD3 homolog (S. cerevisiae)	6.91	5.91	2.95e-04	2.34e-03
NME7	29922	non-metastatic cells 7, protein expressed in (nucl	7.89	6.79	2.31e-06	4.24e-05
NMI	9111	N-myc (and STAT) interactor	7.66	8.68	4.95e-03	2.22e-02
NMNAT1	64802	nicotinamide nucleotide adenylyltransferase 1	8.08	9.07	1.19e-02	4.35e-02
NMNAT3	349565	nicotinamide nucleotide adenylyltransferase 3	7.84	6.92	1.24e-03	7.45e-03
NNMT	4837	nicotinamide N-methyltransferase	12.12	10.83	2.35e-05	2.93e-04
NOL1	4839	nucleolar protein 1, 120kDa	5.28	6.07	9.18e-03	3.56e-02
NOL3	8996	nucleolar protein 3 (apoptosis repressor with CARD	7.20	5.97	7.42e-04	5.01e-03
NOLA3	55505	nucleolar protein family A, member 3 (H/ACA small	9.63	10.44	4.11e-03	1.94e-02
NOSTRIN	115677	nitric oxide synthase trafficker	8.67	7.39	3.31e-03	1.63e-02
NOTCH3	4854	Notch homolog 3 (Drosophila)	7.96	5.89	5.32e-05	5.78e-04
NOV	4856	nephroblastoma overexpressed gene	12.74	9.76	1.76e-08	7.05e-07
NOVA1	4857	neuro-oncological ventral antigen 1	5.87	4.95	1.38e-02	4.88e-02
NOX4	50507	NADPH oxidase 4	5.92	3.55	2.45e-06	4.46e-05
NP	4860	nucleoside phosphorylase	8.38	9.73	3.05e-04	2.41e-03
N-PAC	84656	cytokine-like nuclear factor n-pac	5.98	7.05	1.21e-03	7.33e-03
NPC2	10577	Niemann-Pick disease, type C2	12.05	12.87	3.46e-03	1.69e-02
NPDC1	56654	neural proliferation, differentiation and control,	9.27	8.46	5.36e-03	2.36e-02
NPHP3	27031	nephronophthisis 3 (adolescent)	7.62	6.70	6.22e-05	6.60e-04
NPHP4	261734	nephronophthisis 4	6.21	6.90	1.22e-02	4.46e-02
NPL	80896	N-acetylneuraminate pyruvate lyase (dihydrodipicol	6.92	9.06	4.23e-04	3.13e-03
NPNT	255743	nephronectin	9.78	6.44	4.75e-19	5.49e-16
NPR1	4881	natriuretic peptide receptor A/guanylate cyclase A	8.82	6.17	3.73e-18	3.08e-15
NPR2	4882	natriuretic peptide receptor B/guanylate cyclase B	7.55	6.09	2.73e-06	4.88e-05
NPTX2	4885	neuronal pentraxin II	8.80	5.65	5.65e-16	2.17e-13
NPTXR	23467	neuronal pentraxin receptor	6.32	5.22	5.13e-05	5.63e-04
NPY1R	4886	neuropeptide Y receptor Y1	6.83	5.34	1.84e-03	1.02e-02
NPY5R	4889	neuropeptide Y receptor Y5	6.44	5.34	3.75e-03	1.81e-02
NQO1	1728	NAD(P)H dehydrogenase, quinone 1	9.88	8.30	1.18e-03	7.21e-03
NR1H3	10062	nuclear receptor subfamily 1, group H, member 3	7.39	8.93	5.32e-03	2.35e-02
NR2C2	7182	nuclear receptor subfamily 2, group C, member 2	5.87	6.64	6.13e-03	2.61e-02
NR2F2	7026	nuclear receptor subfamily 2, group F, member 2	10.83	9.24	7.91e-07	1.74e-05
NR3C2	4306	nuclear receptor subfamily 3, group C, member 2	7.40	6.26	3.76e-04	2.84e-03
NRBP2	340371	nuclear receptor binding protein 2	7.93	6.34	2.60e-08	9.79e-07
NRIP1	8204	nuclear receptor interacting protein 1	7.48	8.18	9.97e-03	3.81e-02
NRK	203447	Nik related kinase	5.72	4.80	2.17e-03	1.17e-02
NRXN3	9369	neurexin 3	7.22	5.79	8.70e-04	5.69e-03
NSD1	64324	nuclear receptor binding SET domain protein 1	4.83	5.73	5.96e-03	2.56e-02
NT5C2	22978	5'-nucleotidase, cytosolic II	10.62	11.43	8.47e-03	3.36e-02
NT5C3	51251	5'-nucleotidase, cytosolic III	8.23	8.92	1.03e-02	3.91e-02
NT5DC1	221294	5'-nucleotidase domain containing 1	8.23	7.33	2.57e-04	2.10e-03
NT5DC3	51559	5'-nucleotidase domain containing 3	8.63	7.34	5.65e-06	8.89e-05
NT5E	4907	5'-nucleotidase, ecto (CD73)	8.87	8.29	1.11e-02	4.12e-02
NT5M	56953	5',3'-nucleotidase, mitochondrial	5.14	6.42	1.67e-03	9.48e-03
NTAN1	123803	N-terminal asparagine amidase	9.38	10.16	4.50e-03	2.08e-02
NTE	10908	neuropathy target esterase	7.93	8.80	2.13e-03	1.16e-02
NTF3	4908	neurotrophin 3	8.06	6.04	8.14e-10	5.34e-08
NTN4	59277	netrin 4	5.96	4.29	3.61e-04	2.74e-03
NTNG2	84628	netrin G2	4.98	6.08	1.30e-02	4.67e-02
NTRK3	4916	neurotrophic tyrosine kinase, receptor, type 3	7.74	4.67	4.92e-18	3.55e-15
NUCKS1	64710	nuclear casein kinase and cyclin-dependent kinase	11.69	11.11	1.09e-02	4.08e-02

Symbol	GeneID	Gene Name*	Signal CNTRL	AAA	P	FDR
NUDCD1	84955	NudC domain containing 1	6.12	4.83	2.85e-04	2.28e-03
NUDT1	4521	nudix (nucleoside diphosphate linked moiety X)-typ	7.06	7.92	3.12e-03	1.56e-02
NUDT11	55190	nudix (nucleoside diphosphate linked moiety X)-typ	6.19	4.73	1.39e-04	1.27e-03
NUDT14	256281	nudix (nucleoside diphosphate linked moiety X)-typ	6.32	7.80	1.21e-04	1.13e-03
NUDT4	11163	nudix (nucleoside diphosphate linked moiety X)-typ	10.05	8.98	3.32e-06	5.73e-05
NUDT9	53343	nudix (nucleoside diphosphate linked moiety X)-typ	8.96	8.32	4.58e-03	2.10e-02
NUP210	23225	nucleoporin 210kDa	5.09	8.06	5.48e-06	8.70e-05
NUP50	10762	nucleoporin 50kDa	5.28	6.69	1.77e-03	9.92e-03
NUP62	23636	nucleoporin 62kDa	8.60	9.37	5.32e-03	2.35e-02
NUSAP1	51203	nucleolar and spindle associated protein 1	5.37	6.95	1.14e-04	1.08e-03
NXPH3	11248	neurexophilin 3	7.35	5.05	1.05e-10	9.22e-09
NY-SAR-48	93323	sarcoma antigen NY-SAR-48	5.54	6.38	8.22e-03	3.28e-02
OACT2	129642	O-acyltransferase (membrane bound) domain containi	7.06	6.33	4.35e-03	2.03e-02
OAS1	4938	2',5'-oligoadenylate synthetase 1, 40/46kDa	5.98	7.52	6.32e-04	4.38e-03
OAS2	4939	2'-5'-oligoadenylate synthetase 2, 69/71kDa	5.27	6.81	2.86e-05	3.45e-04
OAS3	4940	2'-5'-oligoadenylate synthetase 3, 100kDa	6.47	7.85	6.35e-04	4.41e-03
OASL	8638	2'-5'-oligoadenylate synthetase-like	5.19	7.59	1.59e-03	9.11e-03
OAT	4942	ornithine aminotransferase (gyrate atrophy)	9.87	9.10	6.89e-04	4.72e-03
OCRL	4952	oculocerebrorenal syndrome of Lowe	8.63	7.77	1.50e-04	1.36e-03
OGFRL1	79627	opioid growth factor receptor-like 1	5.51	6.70	5.93e-03	2.55e-02
OGN	4969	osteoglycin (osteoinductive factor, mimecan)	12.76	9.94	1.17e-07	3.49e-06
OLFM1	10439	olfactomedin 1	8.81	7.51	8.75e-05	8.71e-04
OMD	4958	osteomodulin	9.82	7.63	3.71e-08	1.31e-06
OPN3	23596	opsin 3 (encephalopsin, panopsin)	5.85	7.07	8.04e-03	3.23e-02
OPRL1	4987	opiate receptor-like 1	5.19	7.14	6.35e-05	6.72e-04
OPTN	10133	optineurin	10.16	9.31	1.77e-04	1.55e-03
ORMDL1	94101	ORM1-like 1 (S. cerevisiae)	7.39	6.74	5.33e-03	2.35e-02
OSBP	5007	oxysterol binding protein	9.42	8.76	3.97e-03	1.89e-02
OSBPL1A	114876	oxysterol binding protein-like 1A	10.26	9.46	4.68e-04	3.43e-03
OSBPL3	26031	oxysterol binding protein-like 3	4.53	5.58	9.46e-03	3.65e-02
OSBPL5	114879	oxysterol binding protein-like 5	7.27	6.10	6.65e-05	6.98e-04
OSCAR	126014	osteoclast-associated receptor	5.15	7.69	4.48e-04	3.30e-03
OSM	5008	oncostatin M	7.80	10.41	3.09e-03	1.55e-02
OSRF	23548	osmosis responsive factor	7.39	6.15	1.85e-03	1.03e-02
OSTF1	26578	osteoclast stimulating factor 1	8.49	9.64	1.20e-04	1.13e-03
OTOA	146183	otoancorin	5.44	7.05	2.27e-03	1.22e-02
OXCT1	5019	3-oxoacid CoA transferase 1	8.07	7.04	4.36e-05	4.89e-04
OXER1	165140	oxoeicosanoid (OXE) receptor 1	5.64	4.85	9.37e-03	3.62e-02
P15RS	55197	hypothetical protein FLJ10656	9.80	8.76	5.83e-06	9.12e-05
P2RX1	5023	purinergic receptor P2X, ligand-gated ion channel,	5.73	7.08	1.17e-02	4.30e-02
P2RX4	5025	purinergic receptor P2X, ligand-gated ion channel,	6.90	8.36	4.35e-03	2.03e-02
P2RX7	5027	purinergic receptor P2X, ligand-gated ion channel,	6.02	7.73	9.63e-04	6.15e-03
P2RY10	27334	purinergic receptor P2Y, G-protein coupled, 10	4.84	6.53	6.90e-03	2.86e-02
P2RY11	5032	purinergic receptor P2Y, G-protein coupled, 11	6.18	7.09	6.67e-03	2.78e-02
P2RY13	53829	purinergic receptor P2Y, G-protein coupled, 13	5.71	7.36	3.23e-06	5.61e-05
P2RY8	286530	purinergic receptor P2Y, G-protein coupled, 8	6.37	8.56	2.14e-04	1.80e-03
PABPC3	5042	poly(A) binding protein, cytoplasmic 3	10.04	10.71	1.24e-02	4.49e-02
PABPC4	8761	poly(A) binding protein, cytoplasmic 4 (inducible	7.11	7.86	1.08e-02	4.03e-02
PACAP	51237	proapoptotic caspase adaptor protein	7.58	11.06	8.14e-03	3.26e-02
PACSIN2	11252	protein kinase C and casein kinase substrate in ne	10.45	9.68	6.57e-04	4.53e-03
PADI4	23569	peptidyl arginine deiminase, type IV	6.14	8.52	4.07e-03	1.93e-02
PAFAH1B3	5050	platelet-activating factor acetylhydrolase, isofor	6.51	7.82	8.04e-05	8.14e-04
PAG1	55824	phosphoprotein associated with glycosphingolipid m	7.32	8.61	7.25e-05	7.50e-04
PAIP1	10605	poly(A) binding protein interacting protein 1	9.94	9.19	9.24e-04	5.95e-03
PAK1	5058	p21/Cdc42/Rac1-activated kinase 1 (STE20 homolog,	7.65	8.61	4.16e-03	1.96e-02
PAK3	5063	p21 (CDKN1A)-activated kinase 3	6.82	5.34	6.57e-06	1.01e-04
PAK4	10298	p21(CDKN1A)-activated kinase 4	7.44	6.43	5.74e-05	6.17e-04
PALLD	23022	palladin, cytoskeletal associated protein	11.82	9.86	9.24e-13	1.38e-10
PALM2-AKAP2	445815	PALM2-AKAP2 protein	9.60	8.62	4.27e-04	3.16e-03
PAM	5066	peptidylglycine alpha-amidating monooxygenase	11.89	10.18	3.69e-12	4.73e-10
PANK1	53354	pantothenate kinase 1	6.70	5.84	1.14e-03	7.01e-03
PAPPA	5069	pregnancy-associated plasma protein A, pappalysin	8.08	6.69	1.30e-02	4.67e-02
PAQR4	124222	progestin and adipoQ receptor family member IV	6.70	7.70	5.38e-03	2.36e-02
PARP1	142	poly (ADP-ribose) polymerase family, member 1	9.41	10.11	1.17e-02	4.31e-02
PARP10	84875	poly (ADP-ribose) polymerase family, member 10	8.56	9.78	1.95e-03	1.08e-02
PARP12	64761	poly (ADP-ribose) polymerase family, member 12	7.17	8.26	2.13e-04	1.80e-03
PARP14	54625	poly (ADP-ribose) polymerase family, member 14	7.83	7.01	9.34e-03	3.61e-02

Symbol	GeneID	Gene Name*	Signal CNTRL	AAA	P	FDR
PARVA	55742	parvin, alpha	8.50	7.12	1.07e-07	3.21e-06
PARVG	64098	parvin, gamma	6.97	8.78	2.98e-05	3.57e-04
PASK	23178	PAS domain containing serine/threonine kinase	4.04	6.71	8.27e-03	3.30e-02
PAWR	5074	PRKC, apoptosis, WT1, regulator	7.77	6.16	2.14e-06	3.98e-05
PAX8	7849	paired box gene 8	4.50	5.79	1.13e-02	4.19e-02
PBX1	5087	pre-B-cell leukemia transcription factor 1	7.13	5.22	2.70e-11	2.86e-09
PCBP4	57060	poly(rC) binding protein 4	7.62	6.27	7.62e-05	7.83e-04
PCDH10	57575	protocadherin 10	7.41	5.95	1.95e-05	2.50e-04
PCDH18	54510	protocadherin 18	8.68	7.01	1.78e-07	4.96e-06
PCDH7	5099	BH-protocadherin (brain-heart)	9.95	6.40	3.92e-21	6.47e-18
PCDH9	5101	protocadherin 9	5.70	4.40	1.06e-02	3.98e-02
PCDHB12	56124	protocadherin beta 12	6.32	4.83	2.62e-05	3.21e-04
PCDHB14	56122	protocadherin beta 14	6.20	5.34	2.68e-03	1.39e-02
PCDHB15	56121	protocadherin beta 15	6.94	5.38	6.34e-08	2.06e-06
PCDHB4	56131	protocadherin beta 4	7.93	5.44	1.53e-09	8.93e-08
PCDHB5	26167	protocadherin beta 5	6.24	5.14	1.92e-05	2.46e-04
PCDHB9	56127	protocadherin beta 9	6.39	7.45	1.01e-02	3.85e-02
PCF11	51585	PCF11, cleavage and polyadenylation factor subunit	7.15	8.16	1.47e-03	8.58e-03
PCGF2	7703	polycomb group ring finger 2	9.31	8.21	2.45e-06	4.46e-05
PCGF4	648	polycomb group ring finger 4	9.34	8.78	1.35e-02	4.81e-02
PCK2	5106	phosphoenolpyruvate carboxykinase 2 (mitochondrial	8.22	8.95	6.98e-03	2.88e-02
PCMTD1	115294	protein-L-isoaspartate (D-aspartate) O-methyltrans	9.86	9.04	3.62e-03	1.76e-02
PCNP	57092	PEST-containing nuclear protein	10.19	9.43	7.85e-04	5.23e-03
PCOLCE2	26577	procollagen C-endopeptidase enhancer 2	10.53	9.02	2.97e-06	5.25e-05
PCSK1	5122	proprotein convertase subtilisin/kexin type 1	6.25	4.13	2.04e-06	3.82e-05
PCSK5	5125	proprotein convertase subtilisin/kexin type 5	9.45	8.15	3.97e-08	1.37e-06
PCTP	58488	phosphatidylcholine transfer protein	6.88	7.66	8.33e-03	3.32e-02
PCYOX1	51449	prenylcysteine oxidase 1	8.85	7.90	2.80e-05	3.39e-04
PCYT1A	5130	phosphate cytidylyltransferase 1, choline, alpha	5.32	6.72	7.19e-05	7.44e-04
PCYT2	5833	phosphate cytidylyltransferase 2, ethanolamine	7.50	6.16	2.14e-05	2.71e-04
PDCD1LG2	80380	programmed cell death 1 ligand 2	4.72	5.62	8.37e-03	3.33e-02
PDE1A	5136	phosphodiesterase 1A, calmodulin-dependent	8.19	6.88	1.48e-04	1.35e-03
PDE1B	5153	phosphodiesterase 1B, calmodulin-dependent	6.29	5.36	2.32e-03	1.24e-02
PDE4B	5142	phosphodiesterase 4B, cAMP-specific (phosphodieste	6.67	7.95	4.78e-04	3.48e-03
PDE6B	5158	phosphodiesterase 6B, cGMP-specific, rod, beta (co	5.57	7.62	5.05e-06	8.10e-05
PDE6G	5148	phosphodiesterase 6G, cGMP-specific, rod, gamma	4.64	7.98	3.70e-05	4.24e-04
PDE7A	5150	phosphodiesterase 7A	4.55	5.51	4.11e-03	1.94e-02
PDE8B	8622	phosphodiesterase 8B	8.40	4.48	3.17e-22	1.22e-18
PDGFA	5154	platelet-derived growth factor alpha polypeptide	9.67	8.00	1.88e-07	5.14e-06
PDGFC	56034	platelet derived growth factor C	9.46	8.13	1.26e-06	2.57e-05
PDGFD	80310	platelet derived growth factor D	10.75	7.88	7.61e-16	2.74e-13
PDGFRA	5156	platelet-derived growth factor receptor, alpha pol	9.90	8.02	1.72e-06	3.30e-05
PDGFRB	5159	platelet-derived growth factor receptor, beta poly	11.14	9.59	4.09e-04	3.04e-03
PDK4	5166	pyruvate dehydrogenase kinase, isozyme 4	11.61	10.42	3.26e-03	1.61e-02
PDLIM2	64236	PDZ and LIM domain 2 (mystique)	10.45	9.71	1.40e-02	4.94e-02
PDLIM3	27295	PDZ and LIM domain 3	11.49	8.86	2.27e-16	1.05e-13
PDLIM7	9260	PDZ and LIM domain 7 (enigma)	11.45	9.89	2.79e-09	1.44e-07
PDXK	8566	pyridoxal (pyridoxine, vitamin B6) kinase	6.95	8.42	1.04e-03	6.52e-03
PDZD2	23037	PDZ domain containing 2	6.06	5.01	8.74e-04	5.71e-03
PDZD4	57595	PDZ domain containing 4	6.41	5.49	8.69e-04	5.69e-03
PDZD6	27152	PDZ domain containing 6	6.88	5.49	2.89e-07	7.35e-06
PDZRN3	23024	PDZ domain containing RING finger 3	7.73	5.24	8.69e-05	8.67e-04
PDZRN4	29951	PDZ domain containing RING finger 4	7.89	5.40	4.62e-04	3.39e-03
PEBP1	5037	phosphatidylethanolamine binding protein 1	11.95	11.16	9.03e-04	5.86e-03
PEBP4	157310	phosphatidylethanolamine-binding protein 4	6.67	4.73	7.18e-12	8.72e-10
PEF1	553115	penta-EF-hand domain containing 1	8.97	8.38	9.13e-03	3.55e-02
PEG3	5178	paternally expressed 3	6.99	5.62	3.58e-08	1.28e-06
PELI1	57162	pellino homolog 1 (Drosophila)	8.42	9.19	1.38e-02	4.90e-02
PELI2	57161	pellino homolog 2 (Drosophila)	7.68	7.05	1.39e-02	4.93e-02
PELO	53918	pelota homolog (Drosophila)	9.62	8.96	3.88e-03	1.86e-02
PERP	64065	PERP, TP53 apoptosis effector	9.00	6.95	4.08e-13	6.54e-11
PEX11A	8800	peroxisomal biogenesis factor 11A	7.02	6.13	2.56e-03	1.34e-02
PEX12	5193	peroxisomal biogenesis factor 12	5.47	4.70	8.78e-03	3.45e-02
PEX7	5191	peroxisomal biogenesis factor 7	7.39	6.73	4.12e-03	1.94e-02
PFC	5199	properdin P factor, complement	5.53	7.81	1.20e-04	1.13e-03
PFDN4	5203	prefoldin 4	6.23	5.35	1.77e-03	9.94e-03
PFKM	5213	phosphofructokinase, muscle	7.79	6.47	7.88e-06	1.17e-04

Symbol	GeneID	Gene Name*	Signal CNTRL	Signal AAA	P	FDR
PFN2	5217	profilin 2	10.45	8.04	3.10e-17	1.65e-14
PGCP	10404	plasma glutamate carboxypeptidase	10.70	9.76	3.16e-05	3.73e-04
PGD	5226	phosphogluconate dehydrogenase	6.93	8.69	1.67e-04	1.48e-03
PGK1	5230	phosphoglycerate kinase 1	8.10	9.11	1.01e-02	3.85e-02
PGLS	25796	6-phosphogluconolactonase	8.33	9.02	1.30e-02	4.67e-02
PGM1	5236	phosphoglucomutase 1	9.88	9.27	6.65e-03	2.78e-02
PGM5	5239	phosphoglucomutase 5	5.82	4.26	6.34e-03	2.67e-02
PGRMC1	10857	progesterone receptor membrane component 1	9.32	8.08	8.39e-08	2.63e-06
PGRMC2	10424	progesterone receptor membrane component 2	10.30	9.39	6.72e-05	7.03e-04
PH-4	54681	hypoxia-inducible factor prolyl 4-hydroxylase	8.66	7.85	3.64e-04	2.76e-03
PHACTR1	221692	phosphatase and actin regulator 1	5.35	7.18	3.22e-03	1.60e-02
PHCA	55331	phytoceramidase, alkaline	7.47	8.43	1.49e-03	8.64e-03
PHF17	79960	PHD finger protein 17	8.06	7.05	1.08e-05	1.54e-04
PHGDH	26227	phosphoglycerate dehydrogenase	10.68	8.69	7.97e-07	1.74e-05
PHKA1	5255	phosphorylase kinase, alpha 1 (muscle)	5.96	4.86	6.53e-05	6.87e-04
PHLDA1	22822	pleckstrin homology-like domain, family A, member	6.73	8.02	1.06e-04	1.03e-03
PHLDB2	90102	pleckstrin homology-like domain, family B, member	7.90	6.66	3.01e-04	2.38e-03
PHYH	5264	phytanoyl-CoA 2-hydroxylase	8.52	7.77	9.51e-04	6.09e-03
PIAS3	10401	protein inhibitor of activated STAT, 3	8.29	7.11	6.95e-06	1.05e-04
PIGK	10026	phosphatidylinositol glycan, class K	8.48	7.79	2.53e-03	1.33e-02
PIGW	284098	phosphatidylinositol glycan, class W	4.89	5.97	7.19e-03	2.96e-02
PIK3AP1	118788	phosphoinositide-3-kinase adaptor protein 1	5.57	7.47	3.14e-07	7.84e-06
PIK3CD	5293	phosphoinositide-3-kinase, catalytic, delta polype	7.83	10.00	2.14e-08	8.41e-07
PIK3CG	5294	phosphoinositide-3-kinase, catalytic, gamma polype	6.19	8.47	5.03e-05	5.53e-04
PIK3R1	5295	phosphoinositide-3-kinase, regulatory subunit 1 (p	10.23	9.38	1.13e-03	6.99e-03
PIK4CB	5298	phosphatidylinositol 4-kinase, catalytic, beta pol	6.04	7.14	3.31e-04	2.58e-03
PILRA	29992	paired immunoglobin-like type 2 receptor alpha	5.10	7.25	3.25e-04	2.54e-03
PIM1	5292	pim-1 oncogene	7.56	8.64	9.64e-04	6.15e-03
PIM2	11040	pim-2 oncogene	6.93	9.27	5.94e-04	4.16e-03
PINK1	65018	PTEN induced putative kinase 1	10.08	9.42	3.90e-03	1.86e-02
PIP3-E	26034	phosphoinositide-binding protein PIP3-E	4.74	6.85	8.77e-03	3.45e-02
PIR	8544	pirin (iron-binding nuclear protein)	8.11	7.06	9.79e-06	1.41e-04
PISD	23761	phosphatidylserine decarboxylase	6.60	7.44	2.60e-03	1.36e-02
PITPNB	23760	phosphatidylinositol transfer protein, beta	11.28	10.34	3.36e-05	3.91e-04
PITPNC1	26207	phosphatidylinositol transfer protein, cytoplasmic	4.65	5.49	8.98e-03	3.50e-02
PITPNM1	9600	phosphatidylinositol transfer protein, membrane-as	6.84	8.21	1.20e-03	7.26e-03
PJA2	9867	praja 2, RING-H2 motif containing	12.07	11.11	2.34e-05	2.92e-04
PKD1	5310	polycystic kidney disease 1 (autosomal dominant)	7.63	6.15	2.46e-03	1.30e-02
PKD2	5311	polycystic kidney disease 2 (autosomal dominant)	10.52	8.38	2.11e-14	4.68e-12
PKIA	5569	protein kinase (cAMP-dependent, catalytic) inhibit	4.93	5.98	1.13e-02	4.19e-02
PKN1	5585	protein kinase N1	7.35	8.06	9.10e-03	3.54e-02
PLA2G12A	81579	phospholipase A2, group XIIA	6.42	5.81	1.23e-02	4.48e-02
PLA2G7	7941	phospholipase A2, group VII (platelet-activating f	6.70	9.68	1.13e-02	4.19e-02
PLA2R1	22925	phospholipase A2 receptor 1, 180kDa	6.61	5.59	1.10e-04	1.05e-03
PLAGL2	5326	pleiomorphic adenoma gene-like 2	5.81	6.63	8.29e-03	3.30e-02
PLAU	5328	plasminogen activator, urokinase	9.64	11.47	9.15e-06	1.33e-04
PLAUR	5329	plasminogen activator, urokinase receptor	8.28	10.69	6.83e-03	2.84e-02
PLCB2	5330	phospholipase C, beta 2	5.58	8.08	3.87e-05	4.42e-04
PLCB4	5332	phospholipase C, beta 4	8.12	7.30	6.29e-03	2.66e-02
PLCD1	5333	phospholipase C, delta 1	8.55	7.29	3.35e-07	8.25e-06
PLCE1	51196	phospholipase C, epsilon 1	7.72	5.21	1.15e-08	4.81e-07
PLCG1	5335	phospholipase C, gamma 1	8.98	8.18	7.96e-03	3.21e-02
PLCG2	5336	phospholipase C, gamma 2 (phosphatidylinositol-spe	6.79	8.51	2.53e-04	2.07e-03
PLCL1	5334	phospholipase C-like 1	7.73	5.76	8.97e-07	1.92e-05
PLCL2	23228	phospholipase C-like 2	7.49	8.27	5.39e-03	2.37e-02
PLCXD1	55344	phosphatidylinositol-specific phospholipase C, X d	5.69	7.03	6.70e-05	7.02e-04
PLD5	200150	phospholipase D family, member 5	7.10	5.12	1.37e-05	1.89e-04
PLEK	5341	pleckstrin	7.97	10.86	2.45e-06	4.46e-05
PLEKHA3	65977	pleckstrin homology domain containing, family A (p	8.17	7.51	3.28e-03	1.62e-02
PLEKHA5	54477	pleckstrin homology domain containing, family A me	8.21	6.90	4.39e-05	4.91e-04
PLEKHB2	55041	pleckstrin homology domain containing, family B (e	6.86	7.96	1.68e-03	9.48e-03
PLEKHC1	10979	pleckstrin homology domain containing, family C (w	12.40	10.42	3.93e-12	4.98e-10
PLEKHH2	130271	pleckstrin homology domain containing, family H (w	7.31	5.85	1.54e-06	3.03e-05
PLEKHM1	9842	pleckstrin homology domain containing, family M (w	5.91	7.08	2.62e-04	2.14e-03
PLEKHO1	51177	pleckstrin homology domain containing, family O me	8.53	9.20	1.26e-02	4.57e-02
PLEKHQ1	80301	pleckstrin homology domain containing, family Q me	7.79	9.46	7.41e-06	1.11e-04
PLK3	1263	polo-like kinase 3 (Drosophila)	7.21	8.23	4.79e-04	3.48e-03

Symbol	GeneID	Gene Name*	Signal CNTRL	AAA	P	FDR
PLN	5350	phospholamban	10.36	6.24	9.97e-20	1.28e-16
PLOD2	5352	procollagen-lysine, 2-oxoglutarate 5-dioxygenase 2	9.69	8.87	7.20e-04	4.90e-03
PLS3	5358	plastin 3 (T isoform)	11.93	9.59	4.09e-15	1.12e-12
PLSCR4	57088	phospholipid scramblase 4	10.30	8.94	3.48e-05	4.03e-04
PLVAP	83483	plasmalemma vesicle associated protein	11.76	10.69	3.89e-03	1.86e-02
PLXNB1	5364	plexin B1	6.69	5.27	1.52e-03	8.78e-03
PLXNC1	10154	plexin C1	4.76	5.62	1.16e-02	4.28e-02
PLXND1	23129	plexin D1	5.57	7.17	4.19e-03	1.97e-02
PMAIP1	5366	phorbol-12-myristate-13-acetate-induced protein 1	5.42	7.11	1.32e-06	2.67e-05
PMM2	5373	phosphomannomutase 2	7.14	7.97	2.86e-03	1.46e-02
PNMA1	9240	paraneoplastic antigen MA1	10.60	9.94	9.91e-03	3.80e-02
PNMA2	10687	paraneoplastic antigen MA2	7.72	6.08	6.87e-07	1.54e-05
PNRC2	55629	proline-rich nuclear receptor coactivator 2	10.97	10.39	1.06e-02	3.98e-02
POLD1	5424	polymerase (DNA directed), delta 1, catalytic subu	6.16	7.27	3.84e-04	2.88e-03
POLR1C	9533	polymerase (RNA) I polypeptide C, 30kDa	7.56	8.36	9.10e-03	3.54e-02
POLR2K	5440	polymerase (RNA) II (DNA directed) polypeptide K,	8.84	8.27	1.08e-02	4.05e-02
POMC	5443	proopiomelanocortin (adrenocorticotropin/ beta-lip	5.34	6.55	1.27e-04	1.18e-03
POU2F2	5452	POU domain, class 2, transcription factor 2	5.60	6.64	1.19e-03	7.24e-03
POU6F1	5463	POU domain, class 6, transcription factor 1	7.50	6.37	3.25e-03	1.61e-02
PPAP2A	8611	phosphatidic acid phosphatase type 2A	7.77	6.05	2.54e-06	4.60e-05
PPAP2B	8613	phosphatidic acid phosphatase type 2B	9.53	8.82	1.52e-03	8.79e-03
PPAPDC3	84814	phosphatidic acid phosphatase type 2 domain contai	6.84	5.35	9.19e-07	1.96e-05
PPFIBP2	8495	PTPRF interacting protein, binding protein 2 (lipr	7.83	8.91	2.51e-04	2.06e-03
PPGB	5476	protective protein for beta-galactosidase (galacto	11.53	12.47	8.74e-03	3.44e-02
PPIC	5480	peptidylprolyl isomerase C (cyclophilin C)	9.78	8.70	2.98e-05	3.57e-04
PPIE	10450	peptidylprolyl isomerase E (cyclophilin E)	7.88	7.21	3.49e-03	1.71e-02
PPIF	10105	peptidylprolyl isomerase F (cyclophilin F)	7.13	9.64	7.95e-05	8.07e-04
PPIL5	122769	peptidylprolyl isomerase (cyclophilin)-like 5	5.30	6.45	1.03e-03	6.51e-03
PPL	5493	periplakin	7.22	5.01	3.56e-09	1.76e-07
PPM1F	9647	protein phosphatase 1F (PP2C domain containing)	7.70	8.63	1.61e-03	9.17e-03
PPM1J	333926	protein phosphatase 1J (PP2C domain containing)	6.58	5.80	1.35e-03	7.97e-03
PPM1M	132160	protein phosphatase 1M (PP2C domain containing)	8.78	9.53	6.30e-03	2.67e-02
PPP1CA	5499	protein phosphatase 1, catalytic subunit, alpha is	8.26	9.79	1.12e-05	1.59e-04
PPP1CB	5500	protein phosphatase 1, catalytic subunit, beta iso	10.54	9.46	6.19e-04	4.32e-03
PPP1R12A	4659	protein phosphatase 1, regulatory (inhibitor) subu	8.74	7.74	6.61e-04	4.55e-03
PPP1R12B	4660	protein phosphatase 1, regulatory (inhibitor) subu	6.79	4.61	5.29e-03	2.34e-02
PPP1R14A	94274	protein phosphatase 1, regulatory (inhibitor) subu	11.21	8.51	3.25e-11	3.29e-09
PPP1R16B	26051	protein phosphatase 1, regulatory (inhibitor) subu	6.15	9.05	1.38e-03	8.14e-03
PPP1R1A	5502	protein phosphatase 1, regulatory (inhibitor) subu	6.05	4.51	5.52e-04	3.92e-03
PPP1R3B	79660	protein phosphatase 1, regulatory (inhibitor) subu	7.21	6.38	1.59e-03	9.13e-03
PPP1R3C	5507	protein phosphatase 1, regulatory (inhibitor) subu	12.03	7.31	2.09e-05	2.66e-04
PPP2R2B	5521	protein phosphatase 2 (formerly 2A), regulatory su	7.91	5.45	1.75e-12	2.46e-10
PPP2R3A	5523	protein phosphatase 2 (formerly 2A), regulatory su	7.19	6.07	2.18e-03	1.18e-02
PPP2R3B	28227	protein phosphatase 2 (formerly 2A), regulatory su	6.89	7.91	1.49e-03	8.67e-03
PPP4C	5531	protein phosphatase 4 (formerly X), catalytic subu	8.11	9.27	2.38e-04	1.98e-03
PQLC1	80148	PQ loop repeat containing 1	8.97	9.83	4.69e-03	2.14e-02
PRAF1	64425	polymerase (RNA) I associated factor 1	9.12	8.42	2.12e-03	1.15e-02
PRAF2	11230	PRA1 domain family, member 2	7.76	7.01	1.09e-03	6.76e-03
PRDM1	639	PR domain containing 1, with ZNF domain	6.02	8.68	7.68e-08	2.44e-06
PRDM2	7799	PR domain containing 2, with ZNF domain	6.95	7.77	3.12e-03	1.56e-02
PRDM6	93166	PR domain containing 6	6.07	4.30	1.17e-04	1.10e-03
PRDM7	11105	PR domain containing 7	6.82	7.92	1.99e-04	1.70e-03
PRDX6	9588	peroxiredoxin 6	11.20	10.29	5.81e-05	6.22e-04
PRELP	5549	proline/arginine-rich end leucine-rich repeat prot	12.60	10.11	9.06e-14	1.66e-11
PREX1	57580	phosphatidylinositol 3,4,5-trisphosphate-dependent	6.09	7.30	3.13e-03	1.56e-02
PRG4	10216	proteoglycan 4	9.09	6.71	5.08e-04	3.66e-03
PRIC285	85441	peroxisomal proliferator-activated receptor A inte	5.88	7.44	6.30e-06	9.74e-05
PRICKLE2	166336	prickle-like 2 (Drosophila)	7.58	6.37	3.93e-05	4.47e-04
PRKCB1	5579	protein kinase C, beta 1	7.46	10.20	1.48e-05	2.01e-04
PRKCD	5580	protein kinase C, delta	7.64	8.77	2.41e-04	1.99e-03
PRKCDBP	112464	protein kinase C, delta binding protein	10.34	8.98	3.13e-04	2.46e-03
PRKCH	5583	protein kinase C, eta	8.40	9.27	3.42e-03	1.67e-02
PRKCI	5584	protein kinase C, iota	6.18	5.36	1.23e-02	4.49e-02
PRKCQ	5588	protein kinase C, theta	4.41	6.02	1.21e-02	4.42e-02
PRKCZ	5590	protein kinase C, zeta	5.30	6.49	1.54e-04	1.40e-03
PRKD1	5587	protein kinase D1	7.91	6.55	4.90e-07	1.15e-05
PRKG1	5592	protein kinase, cGMP-dependent, type I	6.21	4.46	5.20e-05	5.69e-04

Symbol	GeneID	Gene Name*	Signal CNTRL	Signal AAA	P	FDR
PRKRA	8575	protein kinase, interferon-inducible double strand	9.29	8.58	1.68e-03	9.51e-03
PRKX	5613	protein kinase, X-linked	5.46	6.94	1.53e-05	2.05e-04
PRKY	5616	protein kinase, Y-linked	6.13	7.63	9.14e-05	9.03e-04
PRLHR	2834	prolactin releasing hormone receptor	5.09	5.96	4.45e-03	2.06e-02
PROCR	10544	protein C receptor, endothelial (EPCR)	8.55	7.58	7.77e-04	5.19e-03
PROS1	5627	protein S (alpha)	11.64	9.71	9.72e-05	9.50e-04
PRR11	55771	proline rich 11	6.44	7.36	5.69e-03	2.47e-02
PRRG1	5638	proline rich Gla (G-carboxyglutamic acid) 1	7.58	6.26	8.50e-08	2.65e-06
PRRT1	80863	proline-rich transmembrane protein 1	6.34	5.04	2.60e-06	4.69e-05
PRRT2	112476	proline-rich transmembrane protein 2	8.22	5.69	3.90e-07	9.40e-06
PRRX1	5396	paired related homeobox 1	8.07	6.77	5.77e-03	2.50e-02
PRRX2	51450	paired related homeobox 2	9.92	7.96	5.57e-06	8.82e-05
PRSS35	167681	protease, serine, 35	6.48	5.73	8.77e-03	3.45e-02
PSAP	5660	prosaposin (variant Gaucher disease and variant me	12.13	13.27	2.72e-03	1.40e-02
PSCD1	9267	pleckstrin homology, Sec7 and coiled-coil domains	8.36	10.04	1.33e-06	2.68e-05
PSCD3	9265	pleckstrin homology, Sec7 and coiled-coil domains	10.20	8.81	1.21e-05	1.70e-04
PSCD4	27128	pleckstrin homology, Sec7 and coiled-coil domains	7.15	9.57	9.52e-09	4.11e-07
PSCDBP	9595	pleckstrin homology, Sec7 and coiled-coil domains,	8.38	9.96	1.07e-05	1.52e-04
PSD3	23362	pleckstrin and Sec7 domain containing 3	6.69	7.70	8.18e-04	5.42e-03
PSD4	23550	pleckstrin and Sec7 domain containing 4	4.69	7.11	5.35e-06	8.51e-05
PSEN1	5663	presenilin 1 (Alzheimer disease 3)	6.83	7.52	1.04e-02	3.94e-02
PSEN2	5664	presenilin 2 (Alzheimer disease 4)	7.28	8.08	5.39e-03	2.37e-02
PSIP1	11168	PC4 and SFRS1 interacting protein 1	9.74	8.64	8.69e-06	1.28e-04
PSMB10	5699	proteasome (prosome, macropain) subunit, beta type	9.29	11.16	2.19e-07	5.82e-06
PSMB8	5696	proteasome (prosome, macropain) subunit, beta type	8.06	9.33	1.04e-02	3.95e-02
PSMB9	5698	proteasome (prosome, macropain) subunit, beta type	8.04	9.71	2.58e-04	2.10e-03
PSME2	5721	proteasome (prosome, macropain) activator subunit	8.31	9.41	2.69e-04	2.18e-03
PSME4	23198	proteasome (prosome, macropain) activator subunit	5.98	6.98	1.61e-03	9.17e-03
PSTPIP1	9051	proline-serine-threonine phosphatase interacting p	7.98	9.32	2.11e-05	2.69e-04
PTAFR	5724	platelet-activating factor receptor	6.10	7.89	2.64e-04	2.14e-03
PTBP2	58155	polypyrimidine tract binding protein 2	8.29	7.08	1.93e-07	5.22e-06
PTCHD1	139411	patched domain containing 1	5.70	4.24	5.56e-03	2.43e-02
PTGER2	5732	prostaglandin E receptor 2 (subtype EP2), 53kDa	5.20	7.34	5.29e-05	5.77e-04
PTGER3	5733	prostaglandin E receptor 3 (subtype EP3)	7.08	5.43	5.58e-04	3.95e-03
PTGFRN	5738	prostaglandin F2 receptor negative regulator	7.62	6.82	1.41e-02	4.98e-02
PTGIR	5739	prostaglandin I2 (prostacyclin) receptor (IP)	6.82	4.87	4.02e-05	4.56e-04
PTGIS	5740	prostaglandin I2 (prostacyclin) synthase	10.14	8.25	3.70e-06	6.21e-05
PTHR1	5745	parathyroid hormone receptor 1	7.92	6.61	2.19e-05	2.76e-04
PTK2	5747	PTK2 protein tyrosine kinase 2	9.95	8.47	2.08e-08	8.20e-07
PTK2B	2185	PTK2B protein tyrosine kinase 2 beta	6.96	8.48	5.32e-06	8.48e-05
PTK9L	11344	PTK9L protein tyrosine kinase 9-like (A6-related p	7.28	8.65	2.71e-03	1.40e-02
PTPDC1	138639	protein tyrosine phosphatase domain containing 1	6.90	6.12	9.09e-04	5.88e-03
PTPLA	9200	protein tyrosine phosphatase-like (proline instead	8.39	6.06	1.91e-15	5.50e-13
PTPN11	5781	protein tyrosine phosphatase, non-receptor type 11	8.73	7.96	6.95e-04	4.75e-03
PTPN13	5783	protein tyrosine phosphatase, non-receptor type 13	6.91	6.08	1.98e-03	1.09e-02
PTPN2	5771	protein tyrosine phosphatase, non-receptor type 2	5.83	6.64	6.28e-03	2.66e-02
PTPN21	11099	protein tyrosine phosphatase, non-receptor type 21	6.47	4.75	5.74e-04	4.04e-03
PTPN22	26191	protein tyrosine phosphatase, non-receptor type 22	5.17	7.49	1.74e-05	2.26e-04
PTPN6	5777	protein tyrosine phosphatase, non-receptor type 6	6.46	9.37	7.42e-09	3.29e-07
PTPN7	5778	protein tyrosine phosphatase, non-receptor type 7	5.17	7.25	6.13e-08	2.01e-06
PTPNS1	140885	protein tyrosine phosphatase, non-receptor type su	10.00	11.23	8.50e-04	5.60e-03
PTPRC	5788	protein tyrosine phosphatase, receptor type, C	5.93	8.63	2.25e-06	4.15e-05
PTPRCAP	5790	protein tyrosine phosphatase, receptor type, C-ass	6.19	9.81	1.29e-02	4.64e-02
PTPRE	5791	protein tyrosine phosphatase, receptor type, E	4.12	6.34	2.58e-05	3.18e-04
PTPRG	5793	protein tyrosine phosphatase, receptor type, G	7.93	5.76	2.31e-10	1.82e-08
PTPRK	5796	protein tyrosine phosphatase, receptor type, K	8.19	6.84	4.96e-05	5.46e-04
PTPRM	5797	protein tyrosine phosphatase, receptor type, M	8.48	7.77	1.01e-02	3.84e-02
PTRF	284119	polymerase I and transcript release factor	7.38	5.61	1.15e-06	2.36e-05
PTTG1	9232	pituitary tumor-transforming 1	5.29	6.98	6.87e-06	1.05e-04
PTTG2	10744	pituitary tumor-transforming 2	6.36	7.20	3.34e-03	1.64e-02
PTTG3	26255	pituitary tumor-transforming 3	5.29	6.33	9.76e-03	3.75e-02
PUM1	9698	pumilio homolog 1 (Drosophila)	9.44	8.87	1.17e-02	4.30e-02
PUS1	80324	pseudouridylate synthase 1	7.61	8.53	1.59e-03	9.12e-03
PVR	5817	poliovirus receptor	6.84	6.16	6.50e-03	2.73e-02
PVRL3	25945	poliovirus receptor-related 3	6.98	5.49	3.62e-06	6.13e-05
PWWP2	170394	PWWP domain containing 2	6.48	7.57	2.83e-04	2.27e-03
PXDN	7837	peroxidasin homolog (Drosophila)	9.74	8.63	1.19e-03	7.24e-03

Symbol	GeneID	Gene Name*	Signal CNTRL	AAA	P	FDR
PXMP2	5827	peroxisomal membrane protein 2, 22kDa	7.91	6.29	2.57e-04	2.10e-03
PYCARD	29108	PYD and CARD domain containing	9.05	11.18	4.64e-08	1.57e-06
PYGB	5834	phosphorylase, glycogen; brain	9.52	8.85	2.99e-03	1.51e-02
QPCT	25797	glutaminyl-peptide cyclotransferase (glutaminyl cy	6.42	8.06	3.08e-05	3.64e-04
QRICH1	54870	glutamine-rich 1	7.61	8.28	1.23e-02	4.47e-02
QTRTD1	79691	queuine tRNA-ribosyltransferase domain containing	6.90	7.66	1.14e-02	4.21e-02
RAB11FIP1	80223	RAB11 family interacting protein 1 (class I)	5.36	6.35	2.83e-03	1.45e-02
RAB11FIP2	22841	RAB11 family interacting protein 2 (class I)	8.26	7.67	9.48e-03	3.65e-02
RAB11FIP5	26056	RAB11 family interacting protein 5 (class I)	6.94	6.25	3.23e-03	1.60e-02
RAB23	51715	RAB23, member RAS oncogene family	9.13	7.39	4.14e-10	2.97e-08
RAB34	83871	RAB34, member RAS oncogene family	10.49	9.67	2.87e-04	2.29e-03
RAB37	326624	RAB37, member RAS oncogene family	6.22	8.39	1.38e-03	8.15e-03
RAB3C	115827	RAB3C, member RAS oncogene family	6.35	5.40	6.00e-04	4.20e-03
RAB4A	5867	RAB4A, member RAS oncogene family	8.10	7.33	7.37e-04	4.99e-03
RAB6A	5870	RAB6A, member RAS oncogene family	9.27	8.70	1.20e-02	4.40e-02
RAB6IP2	23085	RAB6 interacting protein 2	6.66	5.73	4.47e-04	3.29e-03
RAB7	7879	RAB7, member RAS oncogene family	8.28	9.23	7.90e-03	3.19e-02
RAB8A	4218	RAB8A, member RAS oncogene family	8.98	9.72	6.22e-03	2.64e-02
RAB8B	51762	RAB8B, member RAS oncogene family	5.31	6.71	1.01e-02	3.85e-02
RAB9B	51209	RAB9B, member RAS oncogene family	6.85	5.61	7.44e-06	1.11e-04
RABEP2	79874	rabaptin, RAB GTPase binding effector protein 2	7.52	8.62	1.42e-03	8.35e-03
RABGAP1	23637	RAB GTPase activating protein 1	10.27	9.01	6.46e-08	2.09e-06
RABGGTA	5875	Rab geranylgeranyltransferase, alpha subunit	7.59	8.34	5.98e-03	2.56e-02
RAC1	5879	ras-related C3 botulinum toxin substrate 1 (rho fa	7.28	5.96	4.78e-05	5.28e-04
RAC2	5880	ras-related C3 botulinum toxin substrate 2 (rho fa	7.21	9.69	4.96e-07	1.16e-05
RAD51AP1	10635	RAD51 associated protein 1	4.09	6.14	2.30e-03	1.23e-02
RAI2	10742	retinoic acid induced 2	9.32	7.88	5.35e-08	1.77e-06
RALA	5898	v-ral simian leukemia viral oncogene homolog A (ra	9.32	10.40	1.01e-02	3.83e-02
RAMP1	10267	receptor (calcitonin) activity modifying protein 1	10.85	7.68	1.63e-21	3.77e-18
RANBP5	3843	RAN binding protein 5	8.52	7.24	2.49e-05	3.07e-04
RANGAP1	5905	Ran GTPase activating protein 1	7.40	8.30	2.45e-03	1.29e-02
RAPGEF1	2889	Rap guanine nucleotide exchange factor (GEF) 1	8.14	9.71	1.90e-04	1.63e-03
RAPGEF5	9771	Rap guanine nucleotide exchange factor (GEF) 5	7.97	5.97	1.42e-05	1.94e-04
RARB	5915	retinoic acid receptor, beta	7.08	5.51	8.95e-07	1.92e-05
RASA2	5922	RAS p21 protein activator 2	4.82	5.93	4.54e-03	2.09e-02
RASGEF1A	221002	RasGEF domain family, member 1A	5.40	6.23	5.00e-03	2.24e-02
RASGEF1B	153020	RasGEF domain family, member 1B	5.16	7.45	1.09e-03	6.77e-03
RASGRP3	25780	RAS guanyl releasing protein 3 (calcium and DAG-re	4.43	6.90	2.36e-07	6.12e-06
RASL10B	91608	RAS-like, family 10, member B	6.18	3.97	1.35e-04	1.24e-03
RASL11B	65997	RAS-like, family 11, member B	7.80	6.27	6.02e-05	6.41e-04
RASL12	51285	RAS-like, family 12	10.57	7.82	6.67e-13	1.03e-10
RASSF2	9770	Ras association (RalGDS/AF-6) domain family 2	6.68	9.40	6.24e-07	1.41e-05
RASSF4	83937	Ras association (RalGDS/AF-6) domain family 4	8.17	9.53	1.74e-05	2.27e-04
RASSF7	8045	Ras association (RalGDS/AF-6) domain family 7	7.33	8.73	1.24e-05	1.73e-04
RAVER2	55225	hypothetical protein FLJ10770	7.68	5.93	1.03e-09	6.40e-08
RBBP6	5930	retinoblastoma binding protein 6	6.80	5.40	2.18e-03	1.18e-02
RBBP7	5931	retinoblastoma binding protein 7	10.26	9.69	1.26e-02	4.55e-02
RBJ	51277	Ras-associated protein Rap1	6.62	5.82	3.96e-03	1.88e-02
RBM9	23543	RNA binding motif protein 9	5.82	4.35	8.14e-04	5.40e-03
RBMS3	27303	RNA binding motif, single stranded interacting pro	8.96	6.77	1.07e-13	1.92e-11
RBMXL1	494115	RNA binding motif protein, X-linked-like 1	5.97	4.97	7.24e-03	2.97e-02
RBPMS	11030	RNA binding protein with multiple splicing	10.56	8.70	8.33e-07	1.81e-05
RCC2	55920	regulator of chromosome condensation 2	7.63	9.04	1.30e-05	1.80e-04
RCN1	5954	reticulocalbin 1, EF-hand calcium binding domain	9.95	9.22	1.25e-03	7.48e-03
RCN2	5955	reticulocalbin 2, EF-hand calcium binding domain	9.73	8.62	1.34e-06	2.68e-05
RCOR3	55758	REST corepressor 3	8.11	7.21	8.56e-05	8.57e-04
RCSD1	92241	RCSD domain containing 1	7.12	9.14	5.38e-07	1.24e-05
RDH5	5959	retinol dehydrogenase 5 (11-cis and 9-cis)	8.25	7.35	5.42e-04	3.85e-03
RDX	5962	radixin	8.71	7.93	6.75e-04	4.64e-03
REC8L1	9985	REC8-like 1 (yeast)	5.79	7.57	8.04e-07	1.76e-05
RECK	8434	reversion-inducing-cysteine-rich protein with kaza	9.25	7.42	7.93e-06	1.17e-04
REEP1	65055	receptor accessory protein 1	7.70	5.64	1.86e-09	1.04e-07
REEP2	51308	receptor accessory protein 2	6.49	3.82	4.79e-06	7.74e-05
REEP4	80346	receptor accessory protein 4	6.85	8.23	6.33e-03	2.67e-02
REEP5	7905	receptor accessory protein 5	11.31	10.37	5.05e-04	3.64e-03
REL	5966	v-rel reticuloendotheliosis viral oncogene homolog	6.24	8.12	1.41e-03	8.28e-03
RELB	5971	v-rel reticuloendotheliosis viral oncogene homolog	7.20	8.31	4.31e-04	3.19e-03

Symbol	GeneID	Gene Name*	Signal CNTRL	AAA	P	FDR
RENBP	5973	renin binding protein	5.93	7.85	1.63e-06	3.17e-05
RERE	473	arginine-glutamic acid dipeptide (RE) repeats	9.57	8.55	2.03e-03	1.11e-02
RERG	85004	RAS-like, estrogen-regulated, growth inhibitor	10.19	7.91	8.52e-12	1.01e-09
REXO2	25996	REX2, RNA exonuclease 2 homolog (S. cerevisiae)	10.34	9.71	6.07e-03	2.59e-02
RFC5	5985	replication factor C (activator 1) 5, 36.5kDa	5.25	6.11	3.86e-03	1.85e-02
RFWD2	64326	ring finger and WD repeat domain 2	8.08	8.85	4.85e-03	2.19e-02
RFWD3	55159	ring finger and WD repeat domain 3	4.49	5.52	1.24e-02	4.51e-02
RGC32	28984	response gene to complement 32	10.55	11.82	1.98e-03	1.09e-02
RGL3	57139	ral guanine nucleotide dissociation stimulator-lik	6.99	5.76	4.24e-06	7.01e-05
RGMA	56963	RGM domain family, member A	9.58	7.39	4.23e-11	4.21e-09
RGN	9104	regucalcin (senescence marker protein-30)	7.66	5.73	8.22e-12	9.89e-10
RGNEF	64283	Rho-guanine nucleotide exchange factor	7.39	6.12	5.17e-06	8.26e-05
Rgr	266747	Ral-GDS related protein Rgr	4.99	7.41	5.11e-04	3.67e-03
RGS10	6001	regulator of G-protein signalling 10	7.61	8.81	7.96e-05	8.07e-04
RGS17	26575	regulator of G-protein signalling 17	7.17	4.64	5.50e-07	1.26e-05
RGS18	64407	regulator of G-protein signalling 18	6.26	8.82	1.16e-02	4.27e-02
RGS19	10287	regulator of G-protein signalling 19	6.51	9.01	2.37e-05	2.95e-04
RGS2	5997	regulator of G-protein signalling 2, 24kDa	9.72	10.66	2.22e-03	1.20e-02
RGS3	5998	regulator of G-protein signalling 3	11.46	10.44	1.60e-03	9.17e-03
RGS5	8490	regulator of G-protein signalling 5	11.84	8.40	1.23e-22	7.10e-19
RHBDF1	64285	rhomboid family 1 (Drosophila)	9.78	8.04	3.86e-10	2.82e-08
RHBDL6	79651	rhomboid, veinlet-like 6 (Drosophila)	6.50	7.57	5.46e-03	2.39e-02
RHOBTB3	22836	Rho-related BTB domain containing 3	8.61	7.57	1.30e-05	1.80e-04
RHOD	29984	ras homolog gene family, member D	6.89	5.47	1.43e-04	1.31e-03
RHOG	391	ras homolog gene family, member G (rho G)	9.56	10.83	4.08e-05	4.61e-04
RHOH	399	ras homolog gene family, member H	5.61	8.30	5.26e-03	2.32e-02
RHOJ	57381	ras homolog gene family, member J	7.36	6.57	1.99e-03	1.10e-02
RIC8B	55188	resistance to inhibitors of cholinesterase 8 homol	8.73	7.66	3.67e-06	6.18e-05
RIMS3	9783	regulating synaptic membrane exocytosis 3	7.63	6.15	7.44e-09	3.29e-07
RIPK1	8737	receptor (TNFRSF)-interacting serine-threonine kin	4.37	5.68	1.99e-04	1.70e-03
RIPK2	8767	receptor-interacting serine-threonine kinase 2	7.41	8.53	5.09e-03	2.27e-02
RIPK3	11035	receptor-interacting serine-threonine kinase 3	4.63	6.12	2.70e-05	3.30e-04
RIPK4	54101	receptor-interacting serine-threonine kinase 4	6.79	4.39	3.11e-11	3.23e-09
RIS1	25907	Ras-induced senescence 1	6.78	8.39	1.83e-05	2.37e-04
RNASE1	6035	ribonuclease, RNase A family, 1 (pancreatic)	10.91	12.51	1.20e-03	7.27e-03
RNASE4	6038	ribonuclease, RNase A family, 4	10.98	9.63	9.67e-09	4.16e-07
RNASE6	6039	ribonuclease, RNase A family, k6	6.50	8.30	1.33e-06	2.68e-05
RNASEH2A	10535	ribonuclease H2, large subunit	6.58	7.60	1.29e-02	4.64e-02
RNASET2	8635	ribonuclease T2	11.01	13.02	4.97e-05	5.47e-04
RNF14	9604	ring finger protein 14	9.00	8.27	1.33e-03	7.88e-03
RNF146	81847	ring finger protein 146	8.09	7.19	9.18e-05	9.06e-04
RNF149	284996	ring finger protein 149	9.56	10.79	6.09e-05	6.47e-04
RNF166	115992	ring finger protein 166	5.93	8.23	5.26e-03	2.32e-02
RNF185	91445	ring finger protein 185	7.06	6.40	4.14e-03	1.95e-02
RNF24	11237	ring finger protein 24	8.67	7.20	8.39e-03	3.33e-02
RNF38	152006	ring finger protein 38	9.39	8.72	9.49e-03	3.66e-02
RNF44	22838	ring finger protein 44	6.82	7.82	1.14e-03	7.05e-03
RNPEPL1	57140	arginyl aminopeptidase (aminopeptidase B)-like 1	7.59	8.89	4.68e-05	5.18e-04
ROCK1	6093	Rho-associated, coiled-coil containing protein kin	7.38	5.19	1.70e-03	9.61e-03
ROCK2	9475	Rho-associated, coiled-coil containing protein kin	9.23	8.02	1.89e-07	5.17e-06
ROR1	4919	receptor tyrosine kinase-like orphan receptor 1	9.22	7.64	5.82e-05	6.23e-04
RP1-112K5.2	90121	hypothetical protein DT1P1A10	9.31	8.46	1.72e-04	1.51e-03
RP11-130N24.1	340533	KIAA2022 protein	5.89	4.88	6.95e-04	4.75e-03
RP13-297E16.1	8227	DNA segment on chromosome X and Y (unique) 155	9.04	9.82	4.59e-03	2.11e-02
RP1-32F7.2	286499	hypothetical protein FLJ37659	6.08	4.25	2.82e-04	2.27e-03
RP13-360B22.2	84187	hypothetical protein FLJ22679	6.75	7.76	4.10e-03	1.93e-02
RP3-402G11.12	80305	hypothetical protein PP2447	6.18	7.92	5.45e-04	3.87e-03
RP3-477H23.1	23331	KIAA1043 protein	6.64	5.76	4.01e-03	1.90e-02
RP4-691N24.2	9837	DNA replication complex GINS protein PSF1	7.22	8.16	6.86e-03	2.85e-02
RP5-1104E15.5	54471	hypothetical protein FLJ20232	5.01	6.02	8.89e-03	3.48e-02
RP5-821D11.2	150365	similar to mouse meiosis defective 1 gene	4.67	6.14	9.06e-04	5.87e-03
RP5-860F19.3	57593	KIAA1442 protein	6.62	5.56	1.55e-04	1.40e-03
RPA4	29935	replication protein A4, 34kDa	5.71	4.73	1.39e-03	8.18e-03
RPESP	157869	RPE-spondin	9.12	7.30	6.46e-06	9.94e-05
RPIB9	154661	Rap2-binding protein 9	6.99	5.74	1.08e-04	1.04e-03
RPS6KA1	6195	ribosomal protein S6 kinase, 90kDa, polypeptide 1	5.28	7.57	1.55e-05	2.07e-04
RPS6KA4	8986	ribosomal protein S6 kinase, 90kDa, polypeptide 4	6.87	7.65	5.53e-03	2.42e-02

Symbol	GeneID	Gene Name*	Signal CNTRL	AAA	P	FDR
RPUSD1	113000	RNA pseudouridylate synthase domain containing 1	6.30	7.06	8.92e-03	3.49e-02
RPUSD2	27079	RNA pseudouridylate synthase domain containing 2	7.41	8.14	7.24e-03	2.97e-02
RRAD	6236	Ras-related associated with diabetes	8.47	7.13	1.20e-07	3.56e-06
RRAGA	10670	Ras-related GTP binding A	10.10	9.48	6.35e-03	2.68e-02
RRAGB	10325	Ras-related GTP binding B	8.07	7.11	2.60e-05	3.20e-04
RRAS	6237	related RAS viral (r-ras) oncogene homolog	10.95	9.22	7.05e-06	1.06e-04
RRAS2	22800	related RAS viral (r-ras) oncogene homolog 2	6.96	5.86	1.40e-03	8.21e-03
RTN1	6252	reticulon 1	9.60	8.19	2.24e-07	5.90e-06
RTN4IP1	84816	reticulon 4 interacting protein 1	6.23	7.13	2.00e-03	1.10e-02
RUFY3	22902	RUN and FYVE domain containing 3	7.21	5.84	5.21e-08	1.74e-06
RUNX1	861	runt-related transcription factor 1 (acute myeloid	6.82	8.00	8.88e-04	5.78e-03
RUNX3	864	runt-related transcription factor 3	6.12	9.71	2.75e-09	1.44e-07
RWDD2	112611	RWD domain containing 2	7.54	6.86	9.30e-03	3.60e-02
RWDD4A	201965	RWD domain containing 4A	8.81	7.96	1.64e-04	1.47e-03
RYK	6259	RYK receptor-like tyrosine kinase	10.33	9.47	1.69e-04	1.50e-03
RYR2	6262	ryanodine receptor 2 (cardiac)	6.88	4.23	4.79e-05	5.29e-04
RYR3	6263	ryanodine receptor 3	6.61	5.27	3.64e-05	4.18e-04
S100A10	6281	S100 calcium binding protein A10 (annexin II ligan	11.46	12.36	4.39e-03	2.04e-02
S100A8	6279	S100 calcium binding protein A8 (calgranulin A)	5.72	6.76	3.67e-03	1.78e-02
SAMD9	54809	sterile alpha motif domain containing 9	7.83	8.67	2.52e-03	1.33e-02
SAMSN1	64092	SAM domain, SH3 domain and nuclear localisation si	7.01	8.74	2.44e-05	3.02e-04
SAP18	10284	Sin3A-associated protein, 18kDa	8.43	7.08	2.95e-07	7.47e-06
SAP30	8819	Sin3A-associated protein, 30kDa	9.91	8.97	3.08e-04	2.43e-03
SAPS1	22870	SAPS domain family, member 1	6.18	7.03	5.74e-03	2.49e-02
SAT	6303	spermidine/spermine N1-acetyltransferase	11.64	12.75	1.93e-04	1.66e-03
SATB1	6304	special AT-rich sequence binding protein 1 (binds	8.31	9.15	2.57e-03	1.35e-02
SAV1	60485	salvador homolog 1 (Drosophila)	8.59	7.27	2.56e-08	9.70e-07
SBDS	51119	Shwachman-Bodian-Diamond syndrome	10.67	9.41	1.31e-06	2.66e-05
SBF1	6305	SET binding factor 1	8.57	9.31	6.66e-03	2.78e-02
SCAMP1	9522	secretory carrier membrane protein 1	10.11	9.42	2.30e-03	1.23e-02
SCAMP2	10066	secretory carrier membrane protein 2	7.23	8.12	2.22e-03	1.19e-02
SCAMP4	113178	secretory carrier membrane protein 4	8.34	9.15	4.39e-03	2.04e-02
SCAP1	8631	src family associated phosphoprotein 1	5.86	8.10	5.66e-03	2.46e-02
SCARA3	51435	scavenger receptor class A, member 3	8.36	5.96	1.85e-07	5.12e-06
SCARB1	949	scavenger receptor class B, member 1	8.89	10.20	1.72e-03	9.71e-03
SCD	6319	stearoyl-CoA desaturase (delta-9-desaturase)	9.09	11.08	9.91e-04	6.28e-03
SCHIP1	29970	schwannomin interacting protein 1	8.18	6.95	1.24e-03	7.43e-03
SCMH1	22955	sex comb on midleg homolog 1 (Drosophila)	7.17	6.06	3.63e-06	6.13e-05
SCO2	9997	SCO cytochrome oxidase deficient homolog 2 (yeast)	7.35	9.48	1.69e-05	2.22e-04
SCOC	60592	short coiled-coil protein	9.08	8.18	8.31e-05	8.34e-04
SCRG1	11341	scrapie responsive protein 1	10.44	7.67	1.44e-15	4.36e-13
SCRN1	9805	secernin 1	8.14	7.48	3.95e-03	1.88e-02
SDC1	6382	syndecan 1	5.20	7.86	2.09e-03	1.14e-02
SDC3	9672	syndecan 3 (N-syndecan)	9.29	10.41	1.88e-04	1.62e-03
SDC4	6385	syndecan 4 (amphiglycan, ryudocan)	8.95	7.93	2.77e-05	3.37e-04
SDFR1	27020	stromal cell derived factor receptor 1	11.69	10.86	2.59e-04	2.11e-03
SDS	10993	serine dehydratase	6.01	9.62	9.04e-03	3.52e-02
SDSL	113675	serine dehydratase-like	7.67	9.13	6.44e-03	2.72e-02
SEC15L2	23233	SEC15-like 2 (S. cerevisiae)	9.28	8.19	1.77e-06	3.38e-05
SEC22L3	9117	SEC22 vesicle trafficking protein-like 3 (S. cerev	8.75	8.01	1.08e-03	6.71e-03
SEC23B	10483	Sec23 homolog B (S. cerevisiae)	8.04	8.94	1.44e-03	8.44e-03
SEC31L1	22872	SEC31-like 1 (S. cerevisiae)	10.87	10.18	2.35e-03	1.25e-02
SELENBP1	8991	selenium binding protein 1	8.52	7.23	8.29e-03	3.30e-02
SELL	6402	selectin L (lymphocyte adhesion molecule 1)	6.81	9.31	1.03e-03	6.47e-03
SELM	140606	selenoprotein M	11.43	10.03	2.31e-03	1.24e-02
SELPLG	6404	selectin P ligand	6.46	8.50	3.31e-07	8.19e-06
SEMA4A	64218	sema domain, immunoglobulin domain (Ig), transmemb	6.24	8.90	1.43e-09	8.43e-08
SEMA4D	10507	sema domain, immunoglobulin domain (Ig), transmemb	7.07	9.52	3.27e-05	3.82e-04
SEMA5B	54437	sema domain, seven thrombospondin repeats (type 1	5.16	5.99	5.60e-03	2.44e-02
SEPP1	6414	selenoprotein P, plasma, 1	10.70	11.61	6.56e-03	2.75e-02
SEPT10	151011	septin 10	10.25	8.91	7.63e-06	1.14e-04
SEPT4	5414	septin 4	8.38	6.88	7.60e-06	1.13e-04
SEPT6	23157	septin 6	4.42	6.58	1.75e-04	1.53e-03
SEPT8	23176	septin 8	7.56	6.27	3.25e-05	3.80e-04
SEPT9	10801	septin 9	9.39	10.14	1.09e-02	4.08e-02
SERINC1	57515	serine incorporator 1	11.05	10.10	2.94e-05	3.53e-04
SERPINA1	5265	serpin peptidase inhibitor, clade A (alpha-1 antip	7.43	10.00	2.37e-03	1.25e-02

Symbol	GeneID	Gene Name*	Signal CNTRL	Signal AAA	P	FDR
SERPINB1	1992	serpin peptidase inhibitor, clade B (ovalbumin), m	8.47	9.39	1.22e-03	7.36e-03
SERPING1	710	serpin peptidase inhibitor, clade G (C1 inhibitor)	12.20	10.65	1.49e-05	2.01e-04
SERPINI1	5274	serpin peptidase inhibitor, clade I (neuroserpin),	6.78	5.68	9.60e-05	9.39e-04
SERTAD1	29950	SERTA domain containing 1	8.11	9.37	3.22e-03	1.60e-02
SESN1	27244	sestrin 1	8.14	7.56	1.12e-02	4.15e-02
SESTD1	91404	SEC14 and spectrin domains 1	9.96	8.52	1.91e-07	5.19e-06
SETBP1	26040	SET binding protein 1	8.55	7.29	3.56e-04	2.71e-03
SETMAR	6419	SET domain and mariner transposase fusion gene	9.27	8.03	2.12e-07	5.70e-06
SFRS6	6431	splicing factor, arginine/serine-rich 6	10.04	10.98	9.61e-04	6.14e-03
SFRS9	8683	splicing factor, arginine/serine-rich 9	9.67	10.39	7.90e-03	3.19e-02
SFXN1	94081	sideroflexin 1	7.14	8.05	1.31e-03	7.80e-03
SFXN2	118980	sideroflexin 2	5.63	6.89	1.01e-02	3.84e-02
SFXN5	94097	sideroflexin 5	4.73	5.77	1.96e-03	1.08e-02
SGCA	6442	sarcoglycan, alpha (50kDa dystrophin-associated gl	7.73	4.90	1.66e-08	6.70e-07
SGCB	6443	sarcoglycan, beta (43kDa dystrophin-associated gly	9.43	7.38	2.66e-13	4.51e-11
SGCD	6444	sarcoglycan, delta (35kDa dystrophin-associated gl	7.20	5.48	2.97e-03	1.51e-02
SGCE	8910	sarcoglycan, epsilon	9.39	7.95	6.68e-05	7.00e-04
SGEF	26084	Src homology 3 domain-containing guanine nucleotid	6.20	4.94	2.69e-05	3.29e-04
SGIP1	84251	SH3-domain GRB2-like (endophilin) interacting prot	8.80	6.39	3.76e-13	6.11e-11
SGK	6446	serum/glucocorticoid regulated kinase	8.95	11.54	3.37e-05	3.91e-04
SGPL1	8879	sphingosine-1-phosphate lyase 1	6.54	8.33	3.74e-07	9.09e-06
SH2D1A	4068	SH2 domain protein 1A, Duncan's disease (lymphopro	5.86	7.78	2.73e-03	1.41e-02
SH2D2A	9047	SH2 domain protein 2A	6.55	8.55	4.55e-03	2.09e-02
SH2D3C	10044	SH2 domain containing 3C	6.53	7.84	5.12e-03	2.28e-02
SH3BGR	6450	SH3 domain binding glutamic acid-rich protein	8.74	5.93	2.78e-10	2.15e-08
SH3BGRL2	83699	SH3 domain binding glutamic acid-rich protein like	8.89	7.86	2.34e-04	1.95e-03
SH3BGRL3	83442	SH3 domain binding glutamic acid-rich protein like	11.77	12.92	2.20e-04	1.84e-03
SH3BP1	23616	SH3-domain binding protein 1	7.37	10.49	9.97e-05	9.68e-04
SH3BP2	6452	SH3-domain binding protein 2	4.94	6.54	1.01e-05	1.44e-04
SH3D19	152503	SH3 domain protein D19	9.75	8.02	1.06e-07	3.20e-06
SH3KBP1	30011	SH3-domain kinase binding protein 1	8.89	10.02	2.88e-04	2.29e-03
SH3PX3	257364	SH3 and PX domain containing 3	7.44	6.48	7.96e-05	8.07e-04
SH3RF2	153769	SH3 domain containing ring finger 2	6.14	4.52	2.06e-06	3.85e-05
SH3TC1	54436	SH3 domain and tetratricopeptide repeats 1	5.94	7.94	4.68e-07	1.10e-05
SHC4	399694	SHC (Src homology 2 domain containing) family, mem	7.54	5.72	1.02e-07	3.10e-06
SHKBP1	92799	SH3KBP1 binding protein 1	6.82	7.83	7.22e-03	2.96e-02
SHRM	57619	shroom	8.56	5.80	1.15e-15	3.90e-13
SIGLEC10	89790	sialic acid binding Ig-like lectin 10	6.02	8.18	3.31e-08	1.20e-06
SIGLEC11	114132	sialic acid binding Ig-like lectin 11	5.49	6.67	1.78e-03	9.99e-03
SIGLEC5	8778	sialic acid binding Ig-like lectin 5	5.51	6.92	2.95e-03	1.50e-02
SIGLEC7	27036	sialic acid binding Ig-like lectin 7	4.90	6.34	4.68e-03	2.14e-02
SIGLEC9	27180	sialic acid binding Ig-like lectin 9	5.39	6.45	9.02e-03	3.52e-02
SIGLECP3	284367	sialic acid binding Ig-like lectin, pseudogene 3	5.28	6.30	5.57e-03	2.43e-02
SIPA1	6494	signal-induced proliferation-associated gene 1	6.67	8.03	1.87e-04	1.61e-03
SIPA1L2	57568	signal-induced proliferation-associated 1 like 2	5.54	6.63	8.78e-03	3.45e-02
SIRPB2	284759	signal-regulatory protein beta 2	6.03	6.99	5.79e-03	2.50e-02
SIRT7	51547	sirtuin (silent mating type information regulation	7.40	8.38	6.92e-04	4.73e-03
SIT1	27240	signaling threshold regulating transmembrane adapt	4.43	6.78	6.87e-03	2.85e-02
SIX5	147912	sine oculis homeobox homolog 5 (Drosophila)	8.53	7.45	5.87e-03	2.53e-02
SKIV2L2	23517	superkiller viralicidic activity 2-like 2 (S. cere	9.11	8.32	5.14e-04	3.69e-03
SKP1A	6500	S-phase kinase-associated protein 1A (p19A)	12.35	11.67	2.70e-03	1.40e-02
SLA	6503	Src-like-adaptor	7.90	9.27	1.50e-05	2.02e-04
SLA2	84174	Src-like-adaptor 2	4.06	5.69	1.78e-03	9.94e-03
SLAMF1	6504	signaling lymphocytic activation molecule family m	4.56	6.89	6.82e-03	2.83e-02
SLAMF6	114836	SLAM family member 6	5.12	6.90	8.83e-04	5.75e-03
SLAMF7	57823	SLAM family member 7	5.89	8.87	1.50e-03	8.69e-03
SLAMF8	56833	SLAM family member 8	7.43	9.64	4.35e-05	4.89e-04
SLC12A2	6558	solute carrier family 12 (sodium/potassium/chlorid	4.76	5.68	8.39e-03	3.33e-02
SLC12A4	6560	solute carrier family 12 (potassium/chloride trans	8.82	7.65	4.01e-07	9.58e-06
SLC12A9	56996	solute carrier family 12 (potassium/chloride trans	7.64	8.93	3.90e-05	4.44e-04
SLC14A1	6563	solute carrier family 14 (urea transporter), membe	6.11	4.57	1.79e-04	1.56e-03
SLC15A3	51296	solute carrier family 15, member 3	7.90	9.92	5.69e-06	8.95e-05
SLC16A10	117247	solute carrier family 16 (monocarboxylic acid tran	4.91	6.33	3.39e-03	1.66e-02
SLC16A2	6567	solute carrier family 16 (monocarboxylic acid tran	6.59	5.79	6.51e-03	2.74e-02
SLC16A4	9122	solute carrier family 16 (monocarboxylic acid tran	6.78	5.10	3.50e-06	5.97e-05
SLC16A9	220963	solute carrier family 16 (monocarboxylic acid tran	7.74	6.08	3.94e-03	1.88e-02
SLC20A2	6575	solute carrier family 20 (phosphate transporter),	7.64	6.44	2.17e-06	4.03e-05

Symbol	GeneID	Gene Name*	Signal CNTRL	Signal AAA	P	FDR
SLC22A17	51310	solute carrier family 22 (organic cation transport	7.08	5.55	1.60e-04	1.44e-03
SLC22A18	5002	solute carrier family 22 (organic cation transport	8.89	10.22	6.97e-05	7.24e-04
SLC22A18AS	5003	solute carrier family 22 (organic cation transport	4.87	6.27	5.37e-03	2.36e-02
SLC22A3	6581	solute carrier family 22 (extraneuronal monoamine	7.03	5.47	2.17e-06	4.03e-05
SLC22A5	6584	solute carrier family 22 (organic cation transport	6.51	5.87	9.24e-03	3.58e-02
SLC24A3	57419	solute carrier family 24 (sodium/potassium/calcium	8.37	6.68	1.25e-05	1.75e-04
SLC24A6	80024	solute carrier family 24 (sodium/potassium/calcium	6.84	8.26	5.61e-04	3.97e-03
SLC25A12	8604	solute carrier family 25 (mitochondrial carrier, A	7.76	6.33	1.67e-08	6.70e-07
SLC25A16	8034	solute carrier family 25 (mitochondrial carrier; G	5.06	5.94	1.14e-02	4.24e-02
SLC25A19	60386	solute carrier family 25 (mitochondrial deoxynucle	7.34	8.87	3.91e-04	2.93e-03
SLC25A23	79085	solute carrier family 25 (mitochondrial carrier; p	6.44	5.28	4.18e-05	4.70e-04
SLC25A30	253512	solute carrier family 25, member 30	8.52	7.75	7.63e-04	5.11e-03
SLC25A4	291	solute carrier family 25 (mitochondrial carrier; a	10.33	7.93	2.44e-14	5.31e-12
SLC27A1	376497	solute carrier family 27 (fatty acid transporter),	6.82	7.71	3.45e-03	1.69e-02
SLC27A3	11000	solute carrier family 27 (fatty acid transporter),	9.88	8.94	3.19e-05	3.74e-04
SLC2A10	81031	solute carrier family 2 (facilitated glucose trans	8.61	7.41	9.83e-05	9.58e-04
SLC2A12	154091	solute carrier family 2 (facilitated glucose trans	7.14	4.90	3.08e-07	7.69e-06
SLC2A5	6518	solute carrier family 2 (facilitated glucose/fruct	6.94	9.20	9.04e-03	3.52e-02
SLC31A2	1318	solute carrier family 31 (copper transporters), me	7.32	9.17	3.53e-05	4.07e-04
SLC35A4	113829	solute carrier family 35, member A4	5.92	6.98	2.18e-03	1.18e-02
SLC35B1	10237	solute carrier family 35, member B1	8.37	9.16	1.05e-02	3.97e-02
SLC35C2	51006	solute carrier family 35, member C2	6.70	7.46	6.69e-03	2.79e-02
SLC37A1	54020	solute carrier family 37 (glycerol-3-phosphate tra	5.73	6.93	1.75e-04	1.53e-03
SLC37A2	219855	solute carrier family 37 (glycerol-3-phosphate tra	5.50	8.89	1.15e-03	7.06e-03
SLC37A3	84255	solute carrier family 37 (glycerol-3-phosphate tra	8.61	7.50	4.17e-06	6.90e-05
SLC37A4	2542	solute carrier family 37 (glycerol-6-phosphate tra	6.12	7.01	3.90e-03	1.86e-02
SLC39A14	23516	solute carrier family 39 (zinc transporter), membe	10.23	8.34	2.18e-10	1.74e-08
SLC3A2	6520	solute carrier family 3 (activators of dibasic and	8.70	9.46	5.79e-03	2.50e-02
SLC41A3	54946	solute carrier family 41, member 3	9.20	8.17	6.65e-06	1.02e-04
SLC44A1	23446	solute carrier family 44, member 1	10.32	9.57	9.61e-04	6.14e-03
SLC44A2	57153	solute carrier family 44, member 2	8.94	8.26	5.78e-03	2.50e-02
SLC45A3	85414	solute carrier family 45, member 3	5.38	6.61	3.50e-04	2.68e-03
SLC5A3	6526	solute carrier family 5 (inositol transporters), m	6.51	5.09	3.55e-03	1.73e-02
SLC6A12	6539	solute carrier family 6 (neurotransmitter transpor	4.59	5.94	7.94e-05	8.07e-04
SLC6A16	28968	solute carrier family 6, member 16	6.70	5.70	5.61e-03	2.44e-02
SLC7A5	8140	solute carrier family 7 (cationic amino acid trans	4.12	7.60	4.12e-03	1.94e-02
SLC7A7	9056	solute carrier family 7 (cationic amino acid trans	7.73	9.80	2.38e-06	4.35e-05
SLCO2B1	11309	solute carrier organic anion transporter family, m	9.07	10.70	3.02e-06	5.29e-05
SLITL2	114990	slit-like 2 (Drosophila)	10.78	9.05	1.11e-06	2.29e-05
SLPI	6590	secretory leukocyte peptidase inhibitor	10.09	8.62	1.28e-02	4.60e-02
SMAD3	4088	SMAD, mothers against DPP homolog 3 (Drosophila)	8.61	7.63	1.44e-03	8.40e-03
SMAD4	4089	SMAD, mothers against DPP homolog 4 (Drosophila)	9.52	8.90	6.10e-03	2.60e-02
SMAD6	4091	SMAD, mothers against DPP homolog 6 (Drosophila)	9.05	7.52	4.54e-07	1.07e-05
SMAD7	4092	SMAD, mothers against DPP homolog 7 (Drosophila)	9.51	8.08	5.35e-03	2.36e-02
SMAD9	4093	SMAD, mothers against DPP homolog 9 (Drosophila)	6.52	5.02	8.13e-05	8.19e-04
SMAP1	60682	stromal membrane-associated protein 1	8.65	7.89	9.18e-04	5.93e-03
SMAP1L	64744	stromal membrane-associated protein 1-like	8.77	10.66	1.70e-03	9.62e-03
SMARCA1	6594	SWI/SNF related, matrix associated, actin dependen	9.32	7.66	2.46e-10	1.92e-08
SMARCA2	6595	SWI/SNF related, matrix associated, actin dependen	10.39	9.68	2.14e-03	1.16e-02
SMARCA3	6596	SWI/SNF related, matrix associated, actin dependen	8.14	7.39	1.08e-03	6.74e-03
SMARCC2	6601	SWI/SNF related, matrix associated, actin dependen	7.87	7.17	6.66e-03	2.78e-02
SMARCD3	6604	SWI/SNF related, matrix associated, actin dependen	9.04	7.70	1.47e-08	6.01e-07
SMC5L1	23137	SMC5 structural maintenance of chromosomes 5-like	6.92	5.90	1.17e-02	4.31e-02
SMCR7	125170	Smith-Magenis syndrome chromosome region, candidat	5.89	5.02	1.41e-02	4.99e-02
SMOC2	64094	SPARC related modular calcium binding 2	11.97	9.48	1.24e-12	1.78e-10
SMPDL3A	10924	sphingomyelin phosphodiesterase, acid-like 3A	8.05	9.38	1.05e-03	6.59e-03
SMTN	6525	smoothelin	10.22	7.93	1.01e-07	3.08e-06
SNAI2	6591	snail homolog 2 (Drosophila)	7.65	6.85	1.12e-02	4.16e-02
SNAI3	333929	snail homolog 3 (Drosophila)	4.37	6.84	1.31e-06	2.66e-05
SNAPC1	6617	small nuclear RNA activating complex, polypeptide	6.75	7.88	1.02e-02	3.88e-02
SNAPC3	6619	small nuclear RNA activating complex, polypeptide	8.78	7.65	9.90e-07	2.08e-05
SNAPC4	6621	small nuclear RNA activating complex, polypeptide	7.38	8.12	8.15e-03	3.26e-02
SNED1	25992	sushi, nidogen and EGF-like domains 1	8.18	6.96	8.98e-03	3.50e-02
SNF1LK	150094	SNF1-like kinase	7.78	9.14	6.19e-04	4.32e-03
SNN	8303	stannin	6.63	7.84	1.33e-04	1.23e-03
SNRPB	6628	small nuclear ribonucleoprotein polypeptides B and	8.47	9.23	7.99e-03	3.21e-02
SNRPN	6638	small nuclear ribonucleoprotein polypeptide N	9.22	8.29	4.63e-03	2.12e-02

Symbol	GeneID	Gene Name*	Signal CNTRL	AAA	P	FDR
SNTA1	6640	syntrophin, alpha 1 (dystrophin-associated protein	9.20	7.74	2.01e-06	3.77e-05
SNTB2	6645	syntrophin, beta 2 (dystrophin-associated protein	8.93	8.24	7.97e-03	3.21e-02
SNX10	29887	sorting nexin 10	8.10	10.58	7.61e-05	7.83e-04
SNX2	6643	sorting nexin 2	8.77	9.47	1.31e-02	4.69e-02
SNX25	83891	sorting nexin 25	7.32	6.39	1.11e-03	6.85e-03
SNX30	401548	sorting nexin family member 30	5.63	7.15	3.88e-03	1.86e-02
SNX7	51375	sorting nexin 7	8.87	7.36	3.93e-08	1.37e-06
SNX8	29886	sorting nexin 8	5.94	7.95	1.25e-07	3.67e-06
SOCS2	8835	suppressor of cytokine signaling 2	9.46	8.66	8.12e-03	3.25e-02
SOCS5	9655	suppressor of cytokine signaling 5	8.26	7.56	2.18e-03	1.18e-02
SOD1	6647	superoxide dismutase 1, soluble (amyotrophic later	11.49	10.80	2.23e-03	1.20e-02
SOD2	6648	superoxide dismutase 2, mitochondrial	9.30	10.46	3.14e-04	2.47e-03
SOD3	6649	superoxide dismutase 3, extracellular	13.37	11.05	1.88e-12	2.58e-10
SORBS1	10580	sorbin and SH3 domain containing 1	11.29	8.15	3.29e-14	6.77e-12
SORBS2	8470	sorbin and SH3 domain containing 2	8.85	6.79	2.30e-05	2.88e-04
SORBS3	10174	sorbin and SH3 domain containing 3	8.41	7.40	8.14e-05	8.19e-04
SOST	50964	sclerosteosis	8.88	3.51	3.09e-03	1.55e-02
SOX13	9580	SRY (sex determining region Y)-box 13	7.10	6.23	7.42e-03	3.03e-02
SOX15	6665	SRY (sex determining region Y)-box 15	6.85	5.58	3.69e-06	6.20e-05
SP110	3431	SP110 nuclear body protein	7.69	8.71	4.84e-04	3.52e-03
SP140	11262	SP140 nuclear body protein	5.54	7.41	8.90e-04	5.78e-03
SPAG4	6676	sperm associated antigen 4	6.63	8.38	2.25e-04	1.88e-03
SPAG5	10615	sperm associated antigen 5	6.30	7.98	1.01e-06	2.12e-05
SPARCL1	8404	SPARC-like 1 (mast9, hevin)	11.51	8.58	9.93e-12	1.15e-09
SPATA13	221178	spermatogenesis associated 13	5.07	6.10	1.83e-03	1.02e-02
SPATA18	132671	spermatogenesis associated 18 homolog (rat)	8.12	6.29	1.64e-06	3.19e-05
SPBC24	147841	spindle pole body component 24 homolog (S. cerevis	4.89	6.11	5.12e-04	3.68e-03
SPCS3	60559	signal peptidase complex subunit 3 homolog (S. cer	7.29	8.14	2.34e-03	1.25e-02
SPEN	23013	spen homolog, transcriptional regulator (Drosophil	8.93	8.20	1.09e-02	4.06e-02
SPG20	23111	spastic paraplegia 20, spartin (Troyer syndrome)	8.30	6.88	8.64e-04	5.67e-03
SPG3A	51062	spastic paraplegia 3A (autosomal dominant)	7.73	6.48	1.53e-07	4.36e-06
SPHAR	10638	S-phase response (cyclin-related)	10.23	8.82	5.77e-07	1.32e-05
SPHK1	8877	sphingosine kinase 1	6.25	7.83	2.73e-05	3.33e-04
SPI1	6688	spleen focus forming virus (SFFV) proviral integra	7.14	9.29	2.44e-04	2.02e-03
SPIN1	83985	spinster	8.07	9.00	1.11e-03	6.85e-03
SPINT1	6692	serine peptidase inhibitor, Kunitz type 1	4.47	5.96	7.52e-04	5.06e-03
SPINT2	10653	serine peptidase inhibitor, Kunitz type, 2	9.66	8.46	1.79e-06	3.42e-05
SPN	6693	sialophorin (gpL115, leukosialin, CD43)	5.54	7.45	4.88e-07	1.14e-05
SPOCK1	6695	sparc/osteonectin, cwcv and kazal-like domains pro	9.84	8.41	2.41e-04	1.99e-03
SPOCK2	9806	sparc/osteonectin, cwcv and kazal-like domains pro	5.73	8.50	2.16e-04	1.82e-03
SPOP	8405	speckle-type POZ protein	10.58	9.91	3.05e-03	1.54e-02
SPRED2	200734	sprouty-related, EVH1 domain containing 2	7.74	7.17	1.31e-02	4.69e-02
SPRY1	10252	sprouty homolog 1, antagonist of FGF signaling (Dr	11.13	8.65	4.45e-16	1.80e-13
SPRY2	10253	sprouty homolog 2 (Drosophila)	10.31	9.52	1.06e-02	3.98e-02
SPRY4	81848	sprouty homolog 4 (Drosophila)	6.29	5.48	1.21e-02	4.43e-02
SPRYD3	84926	SPRY domain containing 3	9.63	8.62	1.92e-05	2.46e-04
SPTY2D1	144108	SPT2, Suppressor of Ty, domain containing 1 (S. ce	5.91	6.83	4.81e-03	2.17e-02
SRCRB4D	136853	scavenger receptor cysteine rich domain containing	4.62	5.98	1.31e-02	4.69e-02
SRGAP2	23380	SLIT-ROBO Rho GTPase activating protein 2	6.60	7.50	1.99e-03	1.10e-02
SRPX	8406	sushi-repeat-containing protein, X-linked	10.65	8.69	8.78e-05	8.73e-04
SSBP2	23635	single-stranded DNA binding protein 2	8.88	7.94	3.54e-05	4.08e-04
SSH2	85464	slingshot homolog 2 (Drosophila)	6.61	7.97	2.46e-03	1.30e-02
SSH3	54961	slingshot homolog 3 (Drosophila)	8.28	7.48	4.31e-04	3.19e-03
SSPN	8082	sarcospan (Kras oncogene-associated gene)	7.87	5.81	6.62e-03	2.77e-02
ST13	6767	suppression of tumorigenicity 13 (colon carcinoma)	10.08	8.96	3.75e-05	4.28e-04
ST14	6768	suppression of tumorigenicity 14 (colon carcinoma)	5.42	7.65	1.19e-02	4.37e-02
ST3GAL2	6483	ST3 beta-galactoside alpha-2,3-sialyltransferase 2	5.13	6.80	1.70e-06	3.28e-05
ST5	6764	suppression of tumorigenicity 5	7.95	6.84	1.28e-03	7.65e-03
ST6GALNAC3	256435	ST6 (alpha-N-acetyl-neuraminyl-2,3-beta-galactosyl	6.10	5.17	1.90e-03	1.05e-02
ST6GALNAC6	30815	ST6 (alpha-N-acetyl-neuraminyl-2,3-beta-galactosyl	9.16	7.89	6.31e-04	4.38e-03
ST7	7982	suppression of tumorigenicity 7	6.91	5.46	4.39e-06	7.20e-05
ST7L	54879	suppression of tumorigenicity 7 like	6.45	5.45	3.18e-04	2.49e-03
ST8SIA4	7903	ST8 alpha-N-acetyl-neuraminide alpha-2,8-sialyltra	5.50	6.88	9.81e-04	6.24e-03
STAC	6769	SH3 and cysteine rich domain	5.85	4.13	3.87e-05	4.42e-04
STAC2	342667	SH3 and cysteine rich domain 2	7.15	5.15	5.63e-06	8.88e-05
STAG3	10734	stromal antigen 3	4.09	6.53	3.06e-03	1.54e-02
STARD3	10948	START domain containing 3	6.63	7.42	1.08e-02	4.03e-02

Symbol	GeneID	Gene Name*	Signal CNTRL	AAA	P	FDR
STARD8	9754	START domain containing 8	7.31	8.06	6.09e-03	2.60e-02
STAT1	6772	signal transducer and activator of transcription 1	8.63	10.08	2.78e-05	3.38e-04
STAU2	27067	staufen, RNA binding protein, homolog 2 (Drosophil	7.13	6.27	2.58e-04	2.10e-03
STEAP1	26872	six transmembrane epithelial antigen of the prosta	8.06	6.72	5.85e-03	2.52e-02
STEAP2	261729	six transmembrane epithelial antigen of the prosta	8.91	6.70	3.39e-13	5.60e-11
STEAP4	79689	STEAP family member 4	7.24	5.93	7.85e-03	3.17e-02
STK10	6793	serine/threonine kinase 10	7.13	9.44	9.62e-08	2.97e-06
STK11IP	114790	serine/threonine kinase 11 interacting protein	7.00	7.66	1.38e-02	4.88e-02
STK17B	9262	serine/threonine kinase 17b (apoptosis-inducing)	7.08	8.56	1.49e-05	2.01e-04
STK3	6788	serine/threonine kinase 3 (STE20 homolog, yeast)	9.68	8.57	1.43e-06	2.84e-05
STK32C	282974	serine/threonine kinase 32C	5.12	6.37	1.35e-02	4.81e-02
STK38L	23012	serine/threonine kinase 38 like	9.19	7.23	7.80e-11	7.09e-09
STK4	6789	serine/threonine kinase 4	8.97	10.38	1.16e-05	1.64e-04
STK6	6790	serine/threonine kinase 6	4.72	6.15	1.81e-03	1.01e-02
STMN1	3925	stathmin 1/oncoprotein 18	6.57	7.35	4.82e-03	2.18e-02
STOM	2040	stomatin	12.76	11.78	1.70e-05	2.23e-04
STRN3	29966	striatin, calmodulin binding protein 3	8.51	7.65	1.59e-04	1.43e-03
STS	412	steroid sulfatase (microsomal), arylsulfatase C, i	7.84	6.96	3.55e-03	1.73e-02
STX11	8676	syntaxin 11	7.63	9.53	9.96e-05	9.68e-04
STX3A	6809	syntaxin 3A	7.50	8.17	1.28e-02	4.60e-02
STXBP1	6812	syntaxin binding protein 1	7.65	6.76	1.67e-04	1.48e-03
STXBP2	6813	syntaxin binding protein 2	6.62	9.17	2.61e-03	1.36e-02
SUCLA2	8803	succinate-CoA ligase, ADP-forming, beta subunit	8.65	7.46	2.80e-07	7.18e-06
SUHW2	140883	suppressor of hairy wing homolog 2 (Drosophila)	4.91	5.84	1.18e-02	4.33e-02
SULT1A1	6817	sulfotransferase family, cytosolic, 1A, phenol-pre	8.71	10.08	1.65e-05	2.18e-04
SULT1A2	6799	sulfotransferase family, cytosolic, 1A, phenol-pre	5.68	6.97	3.07e-03	1.54e-02
SUMO2	6613	SMT3 suppressor of mif two 3 homolog 2 (yeast)	6.11	5.28	1.11e-03	6.85e-03
SURB7	9412	SRB7 suppressor of RNA polymerase B homolog (yeast	8.02	6.64	8.68e-03	3.42e-02
SUSD1	64420	sushi domain containing 1	7.06	8.00	1.06e-03	6.64e-03
SUSD5	26032	sushi domain containing 5	8.11	4.25	7.94e-07	1.74e-05
SUV420H2	84787	suppressor of variegation 4-20 homolog 2 (Drosophi	5.90	6.62	1.02e-02	3.86e-02
SV2A	9900	synaptic vesicle glycoprotein 2A	6.79	5.45	2.81e-07	7.18e-06
SVIL	6840	supervillin	9.18	6.91	7.52e-07	1.67e-05
SYDE1	85360	synapse defective 1, Rho GTPase, homolog 1 (C. ele	8.12	6.55	5.02e-07	1.17e-05
SYF2	25949	SYF2 homolog, RNA splicing factor (S. cerevisiae)	8.42	7.65	4.78e-03	2.16e-02
SYK	6850	spleen tyrosine kinase	7.37	10.01	2.24e-08	8.68e-07
SYNC1	81493	syncoilin, intermediate filament 1	8.19	5.83	9.83e-16	3.44e-13
SYNGR2	9144	synaptogyrin 2	7.01	8.02	8.38e-04	5.53e-03
SYNPO2	171024	synaptopodin 2	10.80	7.94	1.44e-06	2.85e-05
SYT11	23208	synaptotagmin XI	9.70	8.49	2.15e-07	5.74e-06
SYTL1	84958	synaptotagmin-like 1	5.12	7.66	1.46e-03	8.52e-03
SYTL3	94120	synaptotagmin-like 3	6.08	8.34	7.45e-04	5.03e-03
SYTL4	94121	synaptotagmin-like 4 (granuphilin-a)	6.91	5.52	1.32e-05	1.82e-04
TACC1	6867	transforming, acidic coiled-coil containing protei	11.03	10.43	7.47e-03	3.05e-02
TACC2	10579	transforming, acidic coiled-coil containing protei	8.89	6.76	3.68e-12	4.73e-10
TACC3	10460	transforming, acidic coiled-coil containing protei	4.82	7.56	2.93e-08	1.08e-06
TAF15	8148	TAF15 RNA polymerase II, TATA box binding protein	9.78	9.10	5.78e-03	2.50e-02
TAF9B	51616	TAF9B RNA polymerase II, TATA box binding protein	9.11	8.03	2.53e-06	4.58e-05
TAGAP	117289	T-cell activation GTPase activating protein	4.72	6.63	5.38e-03	2.36e-02
TAGLN	6876	transgelin	13.30	11.28	6.09e-06	9.46e-05
TANC1	85461	tetratricopeptide repeat, ankyrin repeat and coile	8.44	6.59	9.22e-07	1.96e-05
TA-NFKBH	84807	T-cell activation NFKB-like protein	4.16	5.29	4.17e-03	1.96e-02
TAP1	6890	transporter 1, ATP-binding cassette, sub-family B	9.22	10.49	1.10e-04	1.06e-03
TAPBP	6892	TAP binding protein (tapasin)	10.38	11.52	1.86e-04	1.60e-03
TA-PP2C	160760	T-cell activation protein phosphatase 2C	5.39	6.74	2.03e-03	1.11e-02
TARBP1	6894	Tar (HIV-1) RNA binding protein 1	6.94	7.79	2.56e-03	1.34e-02
TARBP2	6895	Tar (HIV-1) RNA binding protein 2	7.61	8.42	3.54e-03	1.73e-02
TASP1	55617	taspase, threonine aspartase, 1	7.25	5.69	1.92e-06	3.61e-05
TBC1D10C	374403	TBC1 domain family, member 10C	6.13	9.16	1.69e-04	1.50e-03
TBC1D19	55296	TBC1 domain family, member 19	6.67	5.87	7.92e-04	5.27e-03
TBC1D22A	25771	TBC1 domain family, member 22A	7.23	8.94	4.44e-06	7.26e-05
TBCC	6903	tubulin-specific chaperone c	6.93	7.82	1.64e-03	9.33e-03
TBL1X	6907	transducin (beta)-like 1X-linked	8.43	7.67	8.15e-04	5.41e-03
TBX2	6909	T-box 2	7.84	6.32	3.32e-03	1.63e-02
TBXA2R	6915	thromboxane A2 receptor	8.72	6.71	4.51e-06	7.35e-05
TBXAS1	6916	thromboxane A synthase 1 (platelet, cytochrome P45	6.55	8.99	5.30e-08	1.76e-06
TCEA1	6917	transcription elongation factor A (SII), 1	10.94	10.32	6.29e-03	2.66e-02

Symbol	GeneID	Gene Name*	Signal CNTRL	AAA	P	FDR
TCEA3	6920	transcription elongation factor A (SII), 3	5.49	4.07	1.48e-03	8.60e-03
TCEAL1	9338	transcription elongation factor A (SII)-like 1	9.70	8.26	5.76e-05	6.19e-04
TCEAL2	140597	transcription elongation factor A (SII)-like 2	10.57	5.56	5.98e-12	7.42e-10
TCEAL3	85012	transcription elongation factor A (SII)-like 3	10.25	8.34	7.85e-14	1.49e-11
TCEAL4	79921	transcription elongation factor A (SII)-like 4	11.65	9.79	2.74e-11	2.88e-09
TCEAL7	56849	transcription elongation factor A (SII)-like 7	6.94	5.33	1.88e-07	5.14e-06
TCEAL8	90843	transcription elongation factor A (SII)-like 8	10.45	9.72	1.38e-03	8.14e-03
TCEB3	6924	transcription elongation factor B (SIII), polypept	6.67	7.53	2.33e-03	1.24e-02
TCF12	6938	transcription factor 12 (HTF4, helix-loop-helix tr	8.99	8.31	7.14e-03	2.94e-02
TCF7	6932	transcription factor 7 (T-cell specific, HMG-box)	7.49	10.35	3.27e-03	1.62e-02
TCF7L1	83439	transcription factor 7-like 1 (T-cell specific, HM	8.71	7.33	3.49e-04	2.68e-03
TCIRG1	10312	T-cell, immune regulator 1, ATPase, H+ transportin	9.22	10.87	3.22e-06	5.61e-05
TCN2	6948	transcobalamin II; macrocytic anemia	8.90	9.68	6.70e-03	2.79e-02
TDG	6996	thymine-DNA glycosylase	8.46	9.27	3.57e-03	1.74e-02
TDO2	6999	tryptophan 2,3-dioxygenase	4.26	7.07	1.41e-06	2.82e-05
TDP1	55775	tyrosyl-DNA phosphodiesterase 1	6.19	7.37	1.07e-04	1.04e-03
TEAD2	8463	TEA domain family member 2	9.32	8.32	4.53e-03	2.09e-02
TEAD3	7005	TEA domain family member 3	7.96	5.95	6.25e-08	2.04e-06
TEAD4	7004	TEA domain family member 4	8.67	7.88	6.59e-03	2.76e-02
TEF	7008	thyrotrophic embryonic factor	9.55	8.19	1.81e-05	2.36e-04
TENC1	23371	tensin like C1 domain containing phosphatase (tens	8.66	7.28	2.43e-03	1.28e-02
TERF1	7013	telomeric repeat binding factor (NIMA-interacting)	7.87	7.08	6.19e-03	2.63e-02
TES	26136	testis derived transcript (3 LIM domains)	8.45	7.00	7.28e-06	1.09e-04
TFAM	7019	transcription factor A, mitochondrial	7.40	8.14	6.87e-03	2.85e-02
TFB2M	64216	transcription factor B2, mitochondrial	6.83	7.78	1.03e-03	6.47e-03
TFEB	7942	transcription factor EB	6.40	7.46	8.69e-04	5.69e-03
TFEC	22797	transcription factor EC	5.05	6.61	6.73e-03	2.80e-02
TFIP11	24144	tuftelin interacting protein 11	8.91	9.66	5.81e-03	2.51e-02
TFRC	7037	transferrin receptor (p90, CD71)	9.25	10.85	8.62e-03	3.41e-02
TGFA	7039	transforming growth factor, alpha	4.65	6.33	2.95e-06	5.25e-05
TGFB1I1	7041	transforming growth factor beta 1 induced transcri	9.74	7.80	1.56e-09	8.96e-08
TGFBR3	7049	transforming growth factor, beta receptor III (bet	10.46	8.51	1.55e-06	3.04e-05
TGIF2	60436	TGFB-induced factor 2 (TALE family homeobox)	7.22	8.03	3.68e-03	1.78e-02
THAP10	56906	THAP domain containing 10	6.66	5.32	3.42e-04	2.64e-03
THBD	7056	thrombomodulin	10.86	11.91	2.50e-03	1.32e-02
THBS2	7058	thrombospondin 2	12.59	10.59	1.45e-06	2.87e-05
THOC4	10189	THO complex 4	8.65	9.46	8.09e-03	3.24e-02
THOP1	7064	thimet oligopeptidase 1	5.84	6.77	6.18e-03	2.63e-02
THRA	7067	thyroid hormone receptor, alpha (erythroblastic le	8.67	7.20	1.04e-09	6.41e-08
THRAP2	23389	thyroid hormone receptor associated protein 2	7.85	6.78	1.06e-03	6.64e-03
THRB	7068	thyroid hormone receptor, beta (erythroblastic leu	9.01	6.15	1.33e-08	5.47e-07
THSD4	79875	thrombospondin, type I, domain containing 4	6.10	5.06	5.23e-03	2.32e-02
THUMPD2	80745	THUMP domain containing 2	7.80	6.36	3.02e-09	1.54e-07
TIAL1	7073	TIA1 cytotoxic granule-associated RNA binding prot	6.96	7.71	8.48e-03	3.36e-02
TIGA1	114915	TIGA1	10.15	9.40	9.56e-04	6.11e-03
TIGD7	91151	tigger transposable element derived 7	7.47	6.24	2.49e-06	4.52e-05
TIMELESS	8914	timeless homolog (Drosophila)	4.85	6.31	7.65e-05	7.85e-04
TIMP4	7079	TIMP metallopeptidase inhibitor 4	8.60	5.92	1.72e-06	3.30e-05
TINAGL1	64129	tubulointerstitial nephritis antigen-like 1	8.63	6.19	7.09e-07	1.58e-05
TJP1	7082	tight junction protein 1 (zona occludens 1)	9.90	8.54	5.90e-07	1.34e-05
TJP2	9414	tight junction protein 2 (zona occludens 2)	9.10	7.43	3.91e-08	1.36e-06
TK1	7083	thymidine kinase 1, soluble	6.09	7.54	2.66e-03	1.38e-02
TLE1	7088	transducin-like enhancer of split 1 (E(sp1) homolo	8.11	6.86	8.13e-05	8.19e-04
TLE2	7089	transducin-like enhancer of split 2 (E(sp1) homolo	9.19	8.24	5.71e-03	2.48e-02
TLN2	83660	talin 2	7.51	5.67	1.24e-06	2.54e-05
TLOC1	7095	translocation protein 1	7.60	6.93	5.66e-03	2.46e-02
TLR1	7096	toll-like receptor 1	5.44	7.44	7.47e-03	3.05e-02
TLR2	7097	toll-like receptor 2	9.04	10.41	1.71e-04	1.50e-03
TLR6	10333	toll-like receptor 6	4.04	5.77	1.38e-02	4.89e-02
TLR7	51284	toll-like receptor 7	4.73	6.78	7.04e-06	1.06e-04
TLR8	51311	toll-like receptor 8	5.90	7.23	4.69e-03	2.14e-02
TM2D2	83877	TM2 domain containing 2	5.26	6.12	1.01e-02	3.85e-02
TM2D3	80213	TM2 domain containing 3	10.04	9.48	1.27e-02	4.60e-02
TM6SF1	53346	transmembrane 6 superfamily member 1	6.17	7.68	1.18e-03	7.20e-03
TMBIM1	64114	transmembrane BAX inhibitor motif containing 1	10.23	9.38	1.65e-04	1.47e-03
TMC6	11322	transmembrane channel-like 6	5.94	8.55	3.08e-09	1.56e-07
TMC8	147138	transmembrane channel-like 8	4.32	6.35	2.20e-07	5.83e-06

Symbol	GeneID	Gene Name*	Signal CNTRL	Signal AAA	P	FDR
TMCC3	57458	transmembrane and coiled-coil domain family 3	6.78	7.65	2.07e-03	1.13e-02
TMCO4	255104	transmembrane and coiled-coil domains 4	8.72	7.86	1.53e-04	1.39e-03
TMEM1	7109	transmembrane protein 1	6.00	7.20	3.98e-04	2.98e-03
TMEM100	55273	transmembrane protein 100	7.27	5.92	2.75e-04	2.22e-03
TMEM106A	113277	transmembrane protein 106A	6.08	7.02	1.19e-03	7.24e-03
TMEM106B	54664	transmembrane protein 106B	8.41	7.51	8.91e-05	8.83e-04
TMEM106C	79022	transmembrane protein 106C	9.86	8.46	3.37e-09	1.70e-07
TMEM117	84216	transmembrane protein 117	6.85	5.88	4.02e-04	3.00e-03
TMEM14A	28978	transmembrane protein 14A	8.22	7.31	7.06e-05	7.33e-04
TMEM16A	55107	transmembrane protein 16A	7.82	5.51	3.18e-11	3.28e-09
TMEM22	80723	transmembrane protein 22	7.58	6.51	2.03e-04	1.72e-03
TMEM25	84866	transmembrane protein 25	6.86	5.12	1.63e-07	4.57e-06
TMEM29	29057	transmembrane protein 29	8.16	7.46	9.97e-03	3.81e-02
TMEM30B	161291	transmembrane protein 30B	8.93	6.97	6.25e-06	9.69e-05
TMEM32	93380	transmembrane protein 32	10.01	9.39	6.67e-03	2.78e-02
TMEM35	59353	transmembrane protein 35	7.27	6.15	2.54e-04	2.08e-03
TMEM37	140738	transmembrane protein 37	5.12	6.53	4.16e-05	4.69e-04
TMEM43	79188	transmembrane protein 43	10.54	9.58	2.45e-05	3.03e-04
TMEM47	83604	transmembrane protein 47	11.89	9.07	6.93e-19	7.27e-16
TMEM50B	757	transmembrane protein 50B	7.22	6.48	1.45e-03	8.46e-03
TMEM51	55092	transmembrane protein 51	8.15	10.00	8.72e-04	5.70e-03
TMEM54	113452	transmembrane protein 54	7.11	5.48	1.95e-04	1.67e-03
TMEM55A	55529	transmembrane protein 55A	8.53	7.45	5.72e-06	8.98e-05
TMEM58	149345	transmembrane protein 58	8.72	7.07	1.32e-04	1.22e-03
TMEM59	9528	transmembrane protein 59	11.46	10.75	1.60e-03	9.17e-03
TMEM70	54968	transmembrane protein 70	6.28	7.76	2.99e-05	3.58e-04
TMEM71	137835	transmembrane protein 71	6.82	8.35	4.47e-05	4.98e-04
TMEM77	128338	transmembrane protein 77	7.53	8.45	1.23e-03	7.39e-03
TMEM8	58986	transmembrane protein 8 (five membrane-spanning do	6.98	7.77	7.15e-03	2.94e-02
TMEM86A	144110	transmembrane protein 86A	4.81	6.57	7.01e-03	2.89e-02
TMEM87A	25963	transmembrane protein 87A	7.69	7.11	1.26e-02	4.56e-02
TMEM9	252839	transmembrane protein 9	10.50	9.72	1.58e-03	9.06e-03
TMEM98	26022	transmembrane protein 98	10.52	9.12	5.08e-06	8.12e-05
TMEM9B	56674	TMEM9 domain family, member B	8.46	9.28	3.19e-03	1.59e-02
TMEPAI	56937	transmembrane, prostate androgen induced RNA	9.97	8.28	1.09e-08	4.67e-07
TMOD1	7111	tropomodulin 1	7.72	5.62	1.33e-13	2.33e-11
TMTC1	83857	transmembrane and tetratricopeptide repeat contain	7.22	5.84	3.41e-07	8.38e-06
TNF	7124	tumor necrosis factor (TNF superfamily, member 2)	4.20	6.46	7.72e-05	7.90e-04
TNFAIP2	7127	tumor necrosis factor, alpha-induced protein 2	8.73	9.91	4.65e-04	3.41e-03
TNFAIP3	7128	tumor necrosis factor, alpha-induced protein 3	8.69	10.14	3.30e-03	1.63e-02
TNFAIP8L1	126282	tumor necrosis factor, alpha-induced protein 8-lik	6.05	7.29	2.37e-03	1.26e-02
TNFAIP8L2	79626	tumor necrosis factor, alpha-induced protein 8-lik	4.70	6.55	1.79e-05	2.33e-04
TNFAIP8L3	388121	tumor necrosis factor, alpha-induced protein 8-lik	9.18	8.38	5.72e-03	2.48e-02
TNFRSF13B	23495	tumor necrosis factor receptor superfamily, member	4.98	6.57	1.30e-02	4.65e-02
TNFRSF14	8764	tumor necrosis factor receptor superfamily, member	7.97	9.47	3.32e-05	3.87e-04
TNFRSF18	8784	tumor necrosis factor receptor superfamily, member	5.29	8.01	8.84e-10	5.70e-08
TNFRSF19L	84957	tumor necrosis factor receptor superfamily, member	4.76	6.24	2.77e-03	1.42e-02
TNFRSF1B	7133	tumor necrosis factor receptor superfamily, member	9.09	11.04	1.59e-03	9.11e-03
TNFRSF21	27242	tumor necrosis factor receptor superfamily, member	6.99	9.18	1.06e-03	6.61e-03
TNFRSF25	8718	tumor necrosis factor receptor superfamily, member	7.46	8.69	1.04e-02	3.94e-02
TNFRSF4	7293	tumor necrosis factor receptor superfamily, member	6.34	8.18	5.57e-03	2.43e-02
TNFSF13	8741	tumor necrosis factor (ligand) superfamily, member	8.15	9.03	4.72e-03	2.14e-02
TNFSF13B	10673	tumor necrosis factor (ligand) superfamily, member	8.01	9.81	9.54e-07	2.01e-05
TNFSF8	944	tumor necrosis factor (ligand) superfamily, member	4.32	5.35	3.66e-03	1.77e-02
TNFSF9	8744	tumor necrosis factor (ligand) superfamily, member	6.05	6.76	1.23e-02	4.47e-02
TNKS1BP1	85456	tankyrase 1 binding protein 1, 182kDa	7.56	6.39	8.24e-05	8.28e-04
TNRC15	26058	trinucleotide repeat containing 15	6.99	6.21	4.62e-03	2.12e-02
TNRC5	10695	trinucleotide repeat containing 5	6.98	7.99	1.28e-03	7.65e-03
TNS1	7145	tensin 1	8.02	6.63	1.97e-03	1.09e-02
TOM1	10043	target of myb1 (chicken)	8.48	9.88	7.26e-03	2.97e-02
TOM1L1	10040	target of myb1-like 1 (chicken)	6.74	5.75	1.14e-04	1.09e-03
TOMM20	9804	translocase of outer mitochondrial membrane 20 hom	10.23	9.38	1.82e-04	1.58e-03
TOP2A	7153	topoisomerase (DNA) II alpha 170kDa	4.59	6.02	6.36e-03	2.68e-02
TOR1AIP1	26092	torsin A interacting protein 1	9.89	9.33	1.29e-02	4.63e-02
TOR2A	27433	torsin family 2, member A	4.67	6.26	1.94e-03	1.07e-02
TPBG	7162	trophoblast glycoprotein	8.88	7.65	1.23e-04	1.15e-03
TPCN2	219931	two pore segment channel 2	6.58	7.78	3.50e-03	1.71e-02

Symbol	GeneID	Gene Name*	Signal CNTRL	AAA	P	FDR
TPD52L1	7164	tumor protein D52-like 1	7.57	6.44	2.74e-03	1.41e-02
TPM1	7168	tropomyosin 1 (alpha)	7.31	5.95	7.73e-06	1.15e-04
TPM2	7169	tropomyosin 2 (beta)	13.25	10.78	2.06e-09	1.13e-07
TPM3	7170	tropomyosin 3	9.77	11.15	1.49e-05	2.01e-04
TPM4	7171	tropomyosin 4	11.64	11.02	9.17e-03	3.56e-02
TPP1	1200	tripeptidyl peptidase I	10.57	11.68	6.85e-04	4.70e-03
TPST2	8459	tyrosylprotein sulfotransferase 2	8.61	9.51	1.54e-03	8.90e-03
TRAF3	7187	TNF receptor-associated factor 3	4.46	5.99	1.28e-03	7.64e-03
TRAF3IP3	80342	TRAF3 interacting protein 3	4.43	7.81	2.97e-07	7.48e-06
TRAK2	66008	trafficking protein, kinesin binding 2	8.78	7.87	3.41e-04	2.63e-03
TRAPPC5	126003	trafficking protein particle complex 5	8.59	9.46	5.21e-03	2.31e-02
TRAPPC6A	79090	trafficking protein particle complex 6A	5.93	7.25	1.91e-03	1.06e-02
TRAT1	50852	T cell receptor associated transmembrane adaptor 1	4.64	6.06	7.69e-03	3.12e-02
TREM1	54210	triggering receptor expressed on myeloid cells 1	6.77	9.54	2.61e-03	1.36e-02
TREX2	11219	three prime repair exonuclease 2	9.39	8.32	5.66e-04	4.00e-03
TRIB1	10221	tribbles homolog 1 (Drosophila)	7.95	10.59	1.36e-09	8.11e-08
TRIB3	57761	tribbles homolog 3 (Drosophila)	6.51	7.82	4.59e-05	5.10e-04
TRIM2	23321	tripartite motif-containing 2	8.71	7.07	3.43e-05	3.97e-04
TRIM23	373	tripartite motif-containing 23	7.64	6.63	1.17e-03	7.18e-03
TRIM3	10612	tripartite motif-containing 3	6.78	5.78	7.88e-05	8.03e-04
TRIM32	22954	tripartite motif-containing 32	6.99	6.06	5.74e-04	4.04e-03
TRIM50B	375593	tripartite motif-containing 50B	5.77	7.14	3.28e-05	3.83e-04
TRIM56	81844	tripartite motif-containing 56	7.67	6.76	4.91e-03	2.21e-02
TRIP10	9322	thyroid hormone receptor interactor 10	10.99	10.34	9.29e-03	3.59e-02
TRNT1	51095	tRNA nucleotidyl transferase, CCA-adding, 1	7.93	7.35	1.12e-02	4.18e-02
TRO	7216	trophinin	8.60	7.29	2.21e-06	4.10e-05
TRPC1	7220	transient receptor potential cation channel, subfa	8.21	6.63	2.14e-09	1.16e-07
TRPV2	51393	transient receptor potential cation channel, subfa	6.31	7.66	1.77e-04	1.55e-03
TSC22D1	8848	TSC22 domain family, member 1	11.42	10.15	1.42e-06	2.83e-05
TSC22D3	1831	TSC22 domain family, member 3	12.46	11.68	8.02e-03	3.22e-02
TSC22D4	81628	TSC22 domain family, member 4	9.79	10.71	1.23e-03	7.40e-03
TSPAN12	23554	tetraspanin 12	6.14	5.09	7.52e-04	5.06e-03
TSPAN17	26262	tetraspanin 17	6.14	7.21	9.40e-04	6.04e-03
TSPAN2	10100	tetraspanin 2	7.18	4.86	2.36e-08	9.05e-07
TSPAN32	10077	tetraspanin 32	5.64	7.33	9.50e-06	1.38e-04
TSPAN33	340348	tetraspanin 33	7.67	9.48	7.72e-07	1.70e-05
TSPAN4	7106	tetraspanin 4	9.35	10.35	1.24e-03	7.46e-03
TSPAN6	7105	tetraspanin 6	8.01	6.90	4.31e-04	3.19e-03
TSPAN8	7103	tetraspanin 8	7.15	5.86	1.50e-03	8.69e-03
TSPAN9	10867	tetraspanin 9	9.49	8.45	4.02e-04	3.00e-03
TSPYL5	85453	TSPY-like 5	7.91	7.09	1.23e-03	7.41e-03
TTC23	64927	tetratricopeptide repeat domain 23	6.96	5.52	9.56e-06	1.39e-04
TTC25	83538	tetratricopeptide repeat domain 25	6.13	5.14	1.34e-04	1.24e-03
TTLL11	158135	tubulin tyrosine ligase-like family, member 11	6.11	4.75	7.55e-07	1.67e-05
TTLL7	79739	tubulin tyrosine ligase-like family, member 7	7.08	4.49	1.43e-16	6.89e-14
TTYH2	94015	tweety homolog 2 (Drosophila)	5.80	6.70	4.41e-03	2.05e-02
TUB	7275	tubby homolog (mouse)	7.01	5.95	3.76e-03	1.81e-02
TUBB3	10381	tubulin, beta 3	4.53	6.09	5.99e-03	2.57e-02
TUBE1	51175	tubulin, epsilon 1	8.06	6.81	5.78e-07	1.32e-05
TUBG2	27175	tubulin, gamma 2	8.24	6.83	1.28e-05	1.78e-04
TUBGCP2	10844	tubulin, gamma complex associated protein 2	8.02	9.03	7.78e-04	5.20e-03
TWIST1	7291	twist homolog 1 (acrocephalosyndactyly 3; Saethre-	8.32	6.66	2.09e-10	1.68e-08
TWIST2	117581	twist homolog 2 (Drosophila)	8.53	7.49	4.53e-03	2.09e-02
TWSG1	57045	twisted gastrulation homolog 1 (Drosophila)	8.51	7.13	5.55e-09	2.61e-07
TXNDC12	51060	thioredoxin domain containing 12 (endoplasmic reti	8.45	9.75	3.34e-04	2.60e-03
TXNRD1	7296	thioredoxin reductase 1	10.32	9.62	8.57e-03	3.39e-02
TYK2	7297	tyrosine kinase 2	7.17	8.52	7.01e-04	4.78e-03
TYROBP	7305	TYRO protein tyrosine kinase binding protein	9.68	12.28	7.67e-05	7.87e-04
TYRP1	7306	tyrosinase-related protein 1	5.99	4.96	7.99e-04	5.31e-03
U2AF1L2	8233	U2(RNU2) small nuclear RNA auxiliary factor 1-like	6.29	5.47	1.06e-02	3.99e-02
U2AF1L4	79713	U2(RNU2) small nuclear RNA auxiliary factor 1-like	6.68	8.40	2.76e-05	3.36e-04
UACA	55075	uveal autoantigen with coiled-coil domains and ank	9.14	8.21	2.39e-03	1.26e-02
UAP1	6675	UDP-N-acteylglucosamine pyrophosphorylase 1	10.97	9.98	2.68e-03	1.39e-02
UAP1L1	91373	UDP-N-acteylglucosamine pyrophosphorylase 1-like 1	5.27	6.56	3.80e-04	2.87e-03
UBA2	10054	SUMO-1 activating enzyme subunit 2	8.02	7.25	2.07e-03	1.13e-02
UBC	7316	ubiquitin C	13.53	12.91	6.84e-03	2.84e-02
UBD	10537	ubiquitin D	4.54	7.86	5.99e-03	2.56e-02

Symbol	GeneID	Gene Name*	Signal CNTRL	AAA	P	FDR
UBE1DC1	79876	ubiquitin-activating enzyme E1-domain containing 1	6.89	5.97	1.65e-04	1.47e-03
UBE2C	11065	ubiquitin-conjugating enzyme E2C	4.60	6.17	1.77e-04	1.55e-03
UBE2D2	7322	ubiquitin-conjugating enzyme E2D 2 (UBC4/5 homolog	4.27	5.59	6.88e-03	2.85e-02
UBE2J1	51465	ubiquitin-conjugating enzyme E2, J1 (UBC6 homolog,	7.47	8.70	5.96e-05	6.35e-04
UBE2L3	7332	ubiquitin-conjugating enzyme E2L 3	8.55	7.87	3.07e-03	1.54e-02
UBN1	29855	ubinuclein 1	8.04	8.87	2.98e-03	1.51e-02
UBXD5	91544	UBX domain containing 5	5.86	7.00	1.04e-03	6.52e-03
UCP2	7351	uncoupling protein 2 (mitochondrial, proton carrie	7.78	10.46	1.81e-09	1.02e-07
UGCG	7357	UDP-glucose ceramide glucosyltransferase	8.64	9.47	4.99e-03	2.24e-02
UGCGL2	55757	UDP-glucose ceramide glucosyltransferase-like 2	8.14	6.97	5.48e-07	1.26e-05
UGDH	7358	UDP-glucose dehydrogenase	10.26	9.27	1.41e-03	8.26e-03
UGP2	7360	UDP-glucose pyrophosphorylase 2	11.06	10.25	3.49e-04	2.68e-03
ULK2	9706	unc-51-like kinase 2 (C. elegans)	6.62	5.60	4.85e-04	3.52e-03
ULK3	25989	unc-51-like kinase 3 (C. elegans)	8.38	9.33	9.89e-04	6.28e-03
UNC119	9094	unc-119 homolog (C. elegans)	7.49	8.23	8.00e-03	3.22e-02
UNC13B	10497	unc-13 homolog B (C. elegans)	7.30	6.64	3.96e-03	1.88e-02
UNC13D	201294	unc-13 homolog D (C. elegans)	5.72	8.11	1.16e-08	4.83e-07
UNC84A	23353	unc-84 homolog A (C. elegans)	9.45	8.54	6.39e-05	6.75e-04
UNC84B	25777	unc-84 homolog B (C. elegans)	6.37	5.15	1.89e-03	1.05e-02
UNC93B1	81622	unc-93 homolog B1 (C. elegans)	4.69	6.14	8.38e-03	3.33e-02
UNG2	10309	uracil-DNA glycosylase 2	6.29	5.17	9.47e-04	6.07e-03
UNQ5783	388325	DTFT5783	5.34	7.64	1.18e-08	4.90e-07
UPF2	26019	UPF2 regulator of nonsense transcripts homolog (ye	7.17	6.50	3.55e-03	1.73e-02
UROS	7390	uroporphyrinogen III synthase (congenital erythrop	8.30	9.10	3.90e-03	1.86e-02
URP2	83706	UNC-112 related protein 2	7.08	9.22	3.72e-08	1.31e-06
USF1	7391	upstream transcription factor 1	7.31	8.18	1.35e-02	4.81e-02
USF2	7392	upstream transcription factor 2, c-fos interacting	7.07	7.76	1.25e-02	4.54e-02
USP13	8975	ubiquitin specific peptidase 13 (isopeptidase T-3)	7.21	6.44	2.90e-03	1.47e-02
USP14	9097	ubiquitin specific peptidase 14 (tRNA-guanine tran	8.37	9.48	2.99e-03	1.51e-02
USP18	11274	ubiquitin specific peptidase 18	5.38	6.51	5.10e-03	2.27e-02
USP35	57558	ubiquitin specific peptidase 35	6.80	4.84	2.24e-06	4.14e-05
USP36	57602	ubiquitin specific peptidase 36	5.44	7.24	1.83e-04	1.59e-03
USP4	7375	ubiquitin specific peptidase 4 (proto-oncogene)	8.00	8.73	7.95e-03	3.20e-02
USP54	159195	ubiquitin specific peptidase 54	6.98	6.23	1.66e-03	9.40e-03
USP7	7874	ubiquitin specific peptidase 7 (herpes virus-assoc	6.44	7.18	8.05e-03	3.23e-02
UST	10090	uronyl-2-sulfotransferase	8.09	6.99	2.08e-05	2.66e-04
UTP14C	9724	UTP14, U3 small nucleolar ribonucleoprotein, homol	8.29	7.59	1.98e-03	1.09e-02
VAC14	55697	Vac14 homolog (S. cerevisiae)	7.02	8.00	8.73e-04	5.71e-03
VAMP2	6844	vesicle-associated membrane protein 2 (synaptobrev	8.57	7.90	3.17e-03	1.58e-02
VAMP4	8674	vesicle-associated membrane protein 4	7.86	7.00	1.65e-04	1.47e-03
VAMP8	8673	vesicle-associated membrane protein 8 (endobrevin)	9.03	11.33	4.62e-06	7.51e-05
VAPA	9218	VAMP (vesicle-associated membrane protein)-associa	4.97	5.94	1.34e-02	4.77e-02
VASH1	22846	vasohibin 1	5.88	7.98	1.14e-02	4.22e-02
VAV1	7409	vav 1 oncogene	5.54	8.09	8.86e-09	3.86e-07
VAV3	10451	vav 3 oncogene	5.60	7.53	1.57e-07	4.45e-06
VCL	7414	vinculin	11.07	9.23	2.19e-12	2.97e-10
VDP	8615	vesicle docking protein p115	10.76	9.91	1.79e-04	1.56e-03
VENTX	27287	VENT homeobox homolog (Xenopus laevis)	5.48	7.88	3.49e-04	2.68e-03
VGLL3	389136	vestigial like 3 (Drosophila)	7.57	6.00	5.68e-05	6.12e-04
VHL	7428	von Hippel-Lindau tumor suppressor	8.16	9.15	1.19e-02	4.37e-02
VIL2	7430	villin 2 (ezrin)	8.94	10.66	1.30e-04	1.21e-03
VLDLR	7436	very low density lipoprotein receptor	8.01	6.65	1.79e-07	4.96e-06
VMO1	284013	vitelline membrane outer layer 1 homolog (chicken)	6.59	7.95	1.33e-02	4.75e-02
VNN1	8876	vanin 1	6.60	7.96	6.54e-03	2.74e-02
VNN2	8875	vanin 2	7.89	9.65	2.79e-03	1.43e-02
VPS13A	23230	vacuolar protein sorting 13A (yeast)	6.96	5.92	4.78e-03	2.16e-02
VPS13D	55187	vacuolar protein sorting 13D (yeast)	6.86	6.06	7.27e-04	4.94e-03
VPS16	64601	vacuolar protein sorting 16 (yeast)	8.32	9.00	1.20e-02	4.40e-02
VPS18	57617	vacuolar protein sorting protein 18	7.65	8.60	9.11e-04	5.89e-03
VPS37B	79720	vacuolar protein sorting 37B (yeast)	8.93	10.29	1.89e-05	2.44e-04
VPS37D	155382	vacuolar protein sorting 37D (yeast)	6.40	5.20	2.73e-06	4.88e-05
VWF	7450	von Willebrand factor	12.21	10.96	4.08e-03	1.93e-02
WARS	7453	tryptophanyl-tRNA synthetase	9.31	10.08	5.09e-03	2.27e-02
WAS	7454	Wiskott-Aldrich syndrome (eczema-thrombocytopenia)	7.72	10.02	1.80e-08	7.15e-07
WASF1	8936	WAS protein family, member 1	6.73	5.89	4.06e-04	3.03e-03
WASF3	10810	WAS protein family, member 3	8.51	6.79	1.21e-11	1.39e-09
WASL	8976	Wiskott-Aldrich syndrome-like	10.03	8.49	4.40e-10	3.11e-08

Symbol	GeneID	Gene Name*	Signal CNTRL	AAA	P	FDR
WBP4	11193	WW domain binding protein 4 (formin binding protei	7.97	7.20	7.55e-04	5.07e-03
WBP5	51186	WW domain binding protein 5	8.80	7.73	6.31e-03	2.67e-02
WBSCR17	64409	Williams-Beuren syndrome chromosome region 17	6.28	4.99	1.10e-04	1.06e-03
WDR19	57728	WD repeat domain 19	8.72	7.54	3.77e-07	9.14e-06
WDR20	91833	WD repeat domain 20	7.60	6.91	2.79e-03	1.43e-02
WDR37	22884	WD repeat domain 37	4.18	5.81	7.91e-03	3.19e-02
WDR50	51096	WD repeat domain 50	6.77	7.50	7.65e-03	3.11e-02
WDR58	79228	WD repeat domain 58	6.21	7.23	7.00e-03	2.89e-02
WDR60	55112	WD repeat domain 60	7.32	6.21	2.37e-06	4.34e-05
WDR68	10238	WD repeat domain 68	7.69	8.78	3.76e-04	2.84e-03
WDR75	84128	WD repeat domain 75	9.25	8.23	9.07e-06	1.32e-04
WDR79	55135	WD repeat domain 79	4.54	5.56	6.72e-03	2.80e-02
WDR81	124997	WD repeat domain 81	6.34	7.27	4.08e-03	1.93e-02
WFDC1	58189	WAP four-disulfide core domain 1	7.75	6.18	1.46e-06	2.88e-05
WFDC3	140686	WAP four-disulfide core domain 3	6.03	5.06	6.73e-03	2.80e-02
WFS1	7466	Wolfram syndrome 1 (wolframin)	8.16	6.68	3.61e-06	6.11e-05
WISP2	8839	WNT1 inducible signaling pathway protein 2	10.19	8.62	7.50e-03	3.05e-02
WNT2B	7482	wingless-type MMTV integration site family, member	5.83	4.81	1.40e-02	4.94e-02
WTIP	126374	Wilms tumor 1 interacting protein	5.77	4.44	1.04e-03	6.56e-03
WWP2	11060	WW domain containing E3 ubiquitin protein ligase 2	8.29	7.53	7.78e-04	5.20e-03
XAB2	56949	XPA binding protein 2	7.97	7.38	1.04e-02	3.94e-02
XBP1	7494	X-box binding protein 1	9.42	10.64	5.69e-03	2.47e-02
XG	7499	Xg blood group	6.67	5.25	9.98e-06	1.43e-04
XKR8	55113	XK, Kell blood group complex subunit-related famil	7.81	8.80	7.44e-04	5.02e-03
XPA	7507	xeroderma pigmentosum, complementation group A	8.74	7.91	2.63e-04	2.14e-03
XPNPEP2	7512	X-prolyl aminopeptidase (aminopeptidase P) 2, memb	6.45	5.01	1.98e-03	1.09e-02
XPO6	23214	exportin 6	8.56	9.81	6.41e-05	6.76e-04
XX-FW81657B9.4	8270	DNA segment on chromosome X (unique) 9879	7.94	9.01	1.30e-03	7.77e-03
YAP1	10413	Yes-associated protein 1, 65kDa	10.27	7.97	4.67e-13	7.39e-11
YES1	7525	v-yes-1 Yamaguchi sarcoma viral oncogene homolog 1	8.05	7.25	8.66e-03	3.42e-02
YIF1B	90522	Yip1 interacting factor homolog B (S. cerevisiae)	7.95	8.89	2.32e-03	1.24e-02
YIPF6	286451	Yip1 domain family, member 6	8.88	8.06	3.20e-04	2.50e-03
YRDC	79693	yrdC domain containing (E. coli)	6.99	8.12	1.57e-04	1.42e-03
YTHDF3	253943	YTH domain family, member 3	7.03	8.13	1.89e-03	1.05e-02
YWHAQ	10971	tyrosine 3-monooxygenase/tryptophan 5-monooxygenas	11.39	10.75	4.78e-03	2.16e-02
ZAK	51776	sterile alpha motif and leucine zipper containing	10.20	8.21	1.80e-14	4.16e-12
ZBED4	9889	zinc finger, BED-type containing 4	4.97	6.75	7.15e-07	1.59e-05
ZBP1	81030	Z-DNA binding protein 1	5.22	6.84	1.51e-04	1.37e-03
ZBTB16	7704	zinc finger and BTB domain containing 16	10.13	7.42	1.48e-15	4.38e-13
ZBTB20	26137	zinc finger and BTB domain containing 20	8.67	7.52	2.89e-04	2.30e-03
ZBTB38	253461	zinc finger and BTB domain containing 38	7.89	6.73	2.98e-03	1.51e-02
ZBTB8	127557	zinc finger and BTB domain containing 8	6.41	5.26	5.59e-06	8.82e-05
ZC3H10	84872	zinc finger CCCH-type containing 10	7.01	6.17	1.33e-03	7.89e-03
ZC3H12A	80149	zinc finger CCCH-type containing 12A	5.77	7.26	5.83e-06	9.12e-05
ZC3H6	376940	zinc finger CCCH-type containing 6	5.93	5.22	1.36e-02	4.84e-02
ZC3HAV1	56829	zinc finger CCCH-type, antiviral 1	5.01	6.16	3.54e-04	2.70e-03
ZCCHC14	23174	zinc finger, CCHC domain containing 14	8.24	7.52	6.63e-03	2.77e-02
ZCCHC6	79670	zinc finger, CCHC domain containing 6	7.82	8.64	3.10e-03	1.55e-02
ZCRB1	85437	zinc finger CCHC-type and RNA binding motif 1	7.13	6.49	6.14e-03	2.61e-02
ZCSL3	120526	zinc finger, CSL-type containing 3	7.24	6.48	2.20e-03	1.18e-02
ZD52F10	93099	dermokine	7.76	5.18	6.82e-15	1.71e-12
ZDHHC12	84885	zinc finger, DHHC-type containing 12	6.47	7.38	1.46e-03	8.52e-03
ZDHHC18	84243	zinc finger, DHHC-type containing 18	6.29	7.38	3.05e-03	1.54e-02
ZDHHC24	254359	zinc finger, DHHC-type containing 24	5.19	6.22	8.18e-04	5.42e-03
ZDHHC7	55625	zinc finger, DHHC-type containing 7	8.56	9.27	1.04e-02	3.93e-02
ZFAND1	79752	zinc finger, AN1-type domain 1	8.65	7.53	1.31e-06	2.66e-05
ZFP2	80108	zinc finger protein 2 homolog (mouse)	5.76	4.97	7.58e-03	3.08e-02
ZFPM2	23414	zinc finger protein, multitype 2	5.97	4.88	1.04e-02	3.94e-02
ZFR	51663	zinc finger RNA binding protein	9.87	9.17	1.77e-03	9.92e-03
ZFYVE21	79038	zinc finger, FYVE domain containing 21	9.41	7.97	5.96e-09	2.74e-07
ZFYVE9	9372	zinc finger, FYVE domain containing 9	8.70	6.92	2.10e-11	2.24e-09
ZGPAT	84619	zinc finger, CCCH-type with G patch domain	8.09	8.95	2.04e-03	1.12e-02
ZHX1	11244	zinc fingers and homeoboxes 1	9.15	8.50	3.97e-03	1.89e-02
ZHX3	23051	zinc fingers and homeoboxes 3	7.59	6.27	1.11e-06	2.29e-05
ZIC1	7545	Zic family member 1 (odd-paired homolog, Drosophil	6.84	4.47	2.51e-04	2.07e-03
ZIK1	284307	zinc finger protein interacting with K protein 1	4.78	5.91	6.48e-04	4.48e-03
ZMPSTE24	10269	zinc metallopeptidase (STE24 homolog, yeast)	10.81	9.97	2.00e-04	1.71e-03

Symbol	GeneID	Gene Name*	Signal CNTRL	AAA	P	FDR
ZMYND15	84225	zinc finger, MYND-type containing 15	6.83	8.55	9.94e-04	6.30e-03
ZNF10	7556	zinc finger protein 10	5.92	4.94	1.53e-03	8.83e-03
ZNF101	94039	zinc finger protein 101	4.84	6.64	8.80e-05	8.73e-04
ZNF133	7692	zinc finger protein 133 (clone pHZ-13)	7.38	6.65	1.49e-03	8.65e-03
ZNF16	7564	zinc finger protein 16 (KOX 9)	6.82	6.24	1.33e-02	4.75e-02
ZNF165	7718	zinc finger protein 165	4.75	6.27	2.13e-04	1.80e-03
ZNF177	7730	zinc finger protein 177	6.36	5.62	4.98e-03	2.23e-02
ZNF187	7741	zinc finger protein 187	8.16	7.53	5.82e-03	2.51e-02
ZNF2	7549	zinc finger protein 2	5.38	4.48	3.58e-03	1.74e-02
ZNF211	10520	zinc finger protein 211	5.76	6.81	4.65e-04	3.41e-03
ZNF218	128553	zinc finger protein 218	6.85	5.93	9.99e-03	3.81e-02
ZNF239	8187	zinc finger protein 239	6.62	5.73	1.16e-03	7.13e-03
ZNF24	7572	zinc finger protein 24 (KOX 17)	4.94	6.21	9.90e-05	9.65e-04
ZNF248	57209	zinc finger protein 248	7.44	6.57	1.24e-02	4.49e-02
ZNF25	219749	zinc finger protein 25 (KOX 19)	9.32	8.22	1.80e-06	3.42e-05
ZNF265	9406	zinc finger protein 265	10.02	9.35	3.08e-03	1.55e-02
ZNF266	10781	zinc finger protein 266	8.61	9.50	1.49e-03	8.64e-03
ZNF267	10308	zinc finger protein 267	7.52	8.81	1.71e-03	9.64e-03
ZNF271	10778	zinc finger protein 271	7.76	7.19	1.32e-02	4.71e-02
ZNF281	23528	zinc finger protein 281	7.58	8.65	2.93e-04	2.33e-03
ZNF291	49855	zinc finger protein 291	7.51	6.70	4.74e-04	3.47e-03
ZNF319	57567	zinc finger protein 319	6.49	7.30	6.77e-03	2.81e-02
ZNF322B	387328	zinc finger protein 322B	6.42	5.37	9.39e-03	3.62e-02
ZNF323	64288	zinc finger protein 323	6.90	5.69	9.19e-04	5.93e-03
ZNF34	80778	zinc finger protein 34 (KOX 32)	7.49	6.62	1.82e-04	1.58e-03
ZNF342	162979	zinc finger protein 342	4.59	6.71	6.74e-08	2.16e-06
ZNF343	79175	zinc finger protein 343	6.89	5.97	1.10e-04	1.06e-03
ZNF358	140467	zinc finger protein 358	10.16	9.24	4.89e-05	5.39e-04
ZNF364	27246	zinc finger protein 364	9.09	8.15	2.37e-03	1.25e-02
ZNF385	25946	zinc finger protein 385	6.19	7.72	1.37e-02	4.86e-02
ZNF395	55893	zinc finger protein 395	10.27	9.23	4.70e-03	2.14e-02
ZNF415	55786	zinc finger protein 415	7.03	5.60	7.18e-07	1.60e-05
ZNF417	147687	zinc finger protein 417	11.82	10.99	5.70e-03	2.47e-02
ZNF433	163059	zinc finger protein 433	5.83	4.10	5.80e-04	4.08e-03
ZNF442	79973	zinc finger protein 442	5.72	4.34	2.94e-04	2.33e-03
ZNF447	65982	zinc finger protein 447	9.55	8.66	3.25e-03	1.61e-02
ZNF502	91392	zinc finger protein 502	6.96	5.81	4.38e-05	4.90e-04
ZNF526	116115	zinc finger protein 526	6.31	5.42	9.72e-03	3.74e-02
ZNF532	55205	zinc finger protein 532	6.86	5.55	1.19e-03	7.25e-03
ZNF540	163255	zinc finger protein 540	6.57	5.39	2.53e-04	2.07e-03
ZNF562	54811	zinc finger protein 562	5.77	6.63	5.62e-03	2.45e-02
ZNF571	51276	zinc finger protein 571	5.93	5.08	4.08e-03	1.93e-02
ZNF580	51157	zinc finger protein 580	9.88	8.95	4.32e-05	4.85e-04
ZNF585B	92285	zinc finger protein 585B	6.36	5.58	9.96e-03	3.81e-02
ZNF611	81856	zinc finger protein 611	5.12	6.09	1.82e-03	1.02e-02
ZNF615	284370	zinc finger protein 615	7.90	7.27	5.83e-03	2.51e-02
ZNF626	199777	zinc finger protein 626	5.75	4.94	4.48e-03	2.07e-02
ZNF627	199692	zinc finger protein 627	8.52	7.76	6.63e-03	2.77e-02
ZNF630	57232	zinc finger protein 630	6.87	5.84	1.58e-05	2.11e-04
ZNF649	65251	zinc finger protein 649	6.64	5.71	1.17e-04	1.11e-03
ZNF652	22834	zinc finger protein 652	10.83	11.60	5.23e-03	2.32e-02
ZNF655	79027	zinc finger protein 655	6.57	7.85	4.43e-05	4.94e-04
ZNF659	79750	zinc finger protein 659	9.16	6.47	6.24e-16	2.32e-13
ZNF668	79759	zinc finger protein 668	6.88	8.06	9.37e-05	9.20e-04
ZNF671	79891	zinc finger protein 671	6.24	7.82	5.44e-05	5.91e-04
ZNF93	81931	zinc finger protein 93 (HTF34)	6.77	5.33	9.94e-03	3.80e-02
ZNFN1A1	10320	zinc finger protein, subfamily 1A, 1 (Ikaros)	5.13	7.68	7.81e-05	7.98e-04
ZNRF2	223082	zinc and ring finger 2	7.48	8.31	2.69e-03	1.40e-02
ZSCAN5	79149	zinc finger and SCAN domain containing 5	5.22	6.28	1.84e-03	1.02e-02
ZXDC	79364	ZXD family zinc finger C	6.43	7.24	4.42e-03	2.05e-02

* Gene names have been truncated to the first 50 characters
