# Supplementary material for: Whole genome expression profiling reveals a significant role for immune function in human abdominal aortic aneurysms
Source: BMC Genomics. 2007 Jul 16;8:237. doi: 10.1186/1471-2164-8-237 (PMC1934369; doi:10.1186/1471-2164-8-237)
Supplement: Additional file 2 — Top 100 most differential genes. The 100 genes included in Figure 1 arranged alphabetically by symbol with gene symbols, Entrez gene IDs, gene names, signals, p-value, and FDR given for the top 100 genes using the group comparisons from Illumina. [file 1471-2164-8-237-S2.pdf]

Table II. Top 100 most differential genes. The 100 genes included in Figure 1 arranged alphabetically by symbol.

Symbol	Entrez Gene ID	Gene Name	Signal* CNTRL	AAA	P	FDR
ADH5	128	alcohol dehydrogenase 5 (class III), chi polypeptide	11.09	8.68	6.51e-15	1.67e-12
ALDH1L1	10840	aldehyde dehydrogenase 1 family, member L1	7.78	4.76	4.29e-18	3.30e-15
AMICA1	120425	adhesion molecule, interacts with CXADR antigen 1	7.26	9.83	2.93e-09	1.50e-07
AOAH	313	acyloxyacyl hydrolase (neutrophil)	5.27	7.72	1.79e-08	7.11e-07
APEG1	10290	aortic preferentially expressed gene 1	10.09	6.99	3.14e-17	1.65e-14
ARHGAP25	9938	Rho GTPase activating protein 25	6.30	9.03	9.32e-10	5.98e-08
ARHGAP27	201176	Rho GTPase activating protein 27	7.55	9.69	2.82e-08	1.04e-06
ARHGAP4	393	Rho GTPase activating protein 4	8.14	10.58	7.31e-09	3.26e-07
ARHGAP9	64333	Rho GTPase activating protein 9	7.52	10.05	2.40e-08	9.16e-07
BTK	695	Bruton agammaglobulinemia tyrosine kinase	6.06	8.73	1.99e-09	1.10e-07
C13orf18	80183	chromosome 13 open reading frame 18	5.43	7.80	1.14e-08	4.80e-07
C1orf38	9473	chromosome 1 open reading frame 38	7.18	9.75	2.59e-09	1.37e-07
C6orf117	112609	chromosome 6 open reading frame 117	10.42	5.48	7.51e-24	8.67e-20
CCL4	6351	chemokine (C-C motif) ligand 4	7.04	10.66	3.45e-10	2.57e-08
CCR2	1231	chemokine (C-C motif) receptor 2	4.03	7.17	1.16e-09	7.10e-08
CD53	963	CD53 antigen	8.67	11.34	4.13e-09	2.01e-07
CD72	971	CD72 antigen	5.20	7.43	2.49e-08	9.47e-07
CD83	9308	CD83 antigen (activated B lymphocytes, immunoglobulin superfamily)	7.27	10.62	6.46e-09	2.91e-07
CKMT2	1160	creatine kinase, mitochondrial 2 (sarcomeric)	9.07	6.38	2.14e-18	1.90e-15
COL4A5	1287	collagen, type IV, alpha 5 (Alport syndrome)	8.87	6.60	6.34e-15	1.66e-12
CORO1A	11151	coronin, actin binding protein, 1A	6.39	9.51	1.54e-09	8.93e-08
CRLF1	9244	cytokine receptor-like factor 1	9.69	7.16	1.22e-15	4.02e-13
CRYAB	1410	crystallin, alpha B	12.72	10.20	3.32e-15	9.34e-13
CSK	1445	c-src tyrosine kinase	6.32	8.92	2.03e-09	1.12e-07
CTSH	1512	cathepsin H	7.56	10.21	1.60e-09	9.12e-08
CYBA	1535	cytochrome b-245, alpha polypeptide	9.39	11.78	1.77e-08	7.07e-07
DEF6	50619	differentially expressed in FDCP 6 homolog (mouse)	5.87	8.74	4.41e-10	3.11e-08
DKFZP686A01247	22998	hypothetical protein	8.11	5.77	1.13e-14	2.78e-12
DSTN	11034	destrin (actin depolymerizing factor)	13.70	11.23	5.24e-18	3.56e-15
DUSP26	78986	dual specificity phosphatase 26 (putative)	9.03	5.98	3.83e-21	6.47e-18
ECGF1	1890	endothelial cell growth factor 1 (platelet-derived)	10.47	12.97	4.78e-09	2.27e-07
EFHD1	80303	EF-hand domain family, member D1	11.59	8.26	2.20e-20	3.18e-17
FAM46B	115572	family with sequence similarity 46, member B	8.83	6.00	7.81e-18	4.74e-15
FBLIM1	54751	filamin binding LIM protein 1	9.87	7.44	2.38e-16	1.06e-13
FBLN5	10516	fibulin 5	11.52	8.60	1.99e-18	1.90e-15
FHL1	2273	four and a half LIM domains 1	12.50	9.72	4.26e-16	1.80e-13
FRZB	2487	frizzled-related protein	12.04	8.68	4.52e-16	1.80e-13

Symbol	Entrez Gene ID	Gene Name	Signal* CNTRL	AAA	P	FDR
GLRB	2743	glycine receptor, beta	7.74	5.67	2.65e-14	5.57e-12
GPR65	8477	G protein-coupled receptor 65	5.47	8.39	1.43e-09	8.43e-08
GPRC5C	55890	G protein-coupled receptor, family C, group 5, member C	9.83	6.98	1.99e-17	1.15e-14
GPSM3	63940	G-protein signalling modulator 3 (AGS3-like, C. elegans)	7.22	9.61	7.52e-09	3.31e-07
HSPB7	27129	heat shock 27kDa protein family, member 7 (cardiovascular)	10.28	6.76	1.14e-21	3.28e-18
IGSF6	10261	immunoglobulin superfamily, member 6	5.89	8.44	2.64e-09	1.39e-07
IL10RA	3587	interleukin 10 receptor, alpha	5.94	8.44	3.47e-09	1.74e-07
IL1B	3553	interleukin 1, beta	6.27	9.81	9.03e-11	8.08e-09
IL2RG	3561	interleukin 2 receptor, gamma (severe combined immunodeficiency)	6.04	8.69	4.44e-09	2.13e-07
ITGAL	3683	integrin, alpha L (antigen CD11A (p180), lymphocyte function-associated antigen 1; alpha polypeptide)	5.31	7.61	1.20e-08	4.96e-07
KCNMB1	3779	potassium large conductance calcium-activated channel, subfamily M, beta member 1	10.96	8.71	2.06e-14	4.67e-12
LCP2	3937	lymphocyte cytosolic protein 2 (SH2 domain containing leukocyte protein of 76kDa)	7.27	9.63	6.41e-09	2.90e-07
LDB3	11155	LIM domain binding 3	7.00	4.48	1.27e-15	4.08e-13
LGR6	59352	leucine-rich repeat-containing G protein-coupled receptor 6	7.46	5.09	1.16e-14	2.79e-12
LMO3	55885	LIM domain only 3 (rhombotin-like 2)	9.89	6.98	6.45e-18	4.13e-15
LPXN	9404	leupaxin	8.30	10.58	1.34e-08	5.49e-07
LRCH2	57631	leucine-rich repeats and calponin homology (CH) domain containing 2	8.51	6.15	1.65e-14	3.89e-12
LRP2BP	55805	LRP2 binding protein	9.78	7.12	4.39e-15	1.18e-12
LTBP1	4052	latent transforming growth factor beta binding protein 1	12.15	9.33	1.41e-15	4.36e-13
MGC15476	147906	thymus expressed gene 3-like	8.82	6.15	2.48e-14	5.31e-12
MPEG1	219972	macrophage expressed gene 1	6.87	9.80	4.37e-10	3.11e-08
MYH10	4628	myosin, heavy polypeptide 10, non-muscle	13.02	9.85	9.61e-17	4.82e-14
NCKAP1L	3071	NCK-associated protein 1-like	6.53	9.62	3.87e-09	1.89e-07
NPNT	255743	nephronectin	9.78	6.44	4.75e-19	5.49e-16
NPR1	4881	natriuretic peptide receptor A/guanylate cyclase A (atrionatriuretic peptide receptor A)	8.82	6.17	3.73e-18	3.08e-15
NPTX2	4885	neuronal pentraxin II	8.80	5.65	5.65e-16	2.17e-13
NTRK3	4916	neurotrophic tyrosine kinase, receptor, type 3	7.74	4.67	4.92e-18	3.55e-15
PCDH7	5099	BH-protocadherin (brain-heart)	9.95	6.40	3.92e-21	6.47e-18
PDE8B	8622	phosphodiesterase 8B	8.40	4.48	3.17e-22	1.22e-18
PDGFD	80310	platelet derived growth factor D	10.75	7.88	7.61e-16	2.74e-13
PDLIM3	27295	PDZ and LIM domain 3	11.49	8.86	2.27e-16	1.05e-13
PFN2	5217	profilin 2	10.45	8.04	3.10e-17	1.65e-14
PIK3CD	5293	phosphoinositide-3-kinase, catalytic, delta polypeptide	7.83	10.00	2.14e-08	8.41e-07
PKD2	5311	polycystic kidney disease 2 (autosomal dominant)	10.52	8.38	2.11e-14	4.68e-12
PLN	5350	phospholamban	10.36	6.24	9.97e-20	1.28e-16
PLS3	5358	plastin 3 (T isoform)	11.93	9.59	4.09e-15	1.12e-12
PSCD4	27128	pleckstrin homology, Sec7 and coiled-coil domains 4	7.15	9.57	9.52e-09	4.11e-07
PTPLA	9200	protein tyrosine phosphatase-like (proline instead of catalytic arginine), member A	8.39	6.06	1.91e-15	5.50e-13

Symbol	Entrez Gene ID	Gene Name	Signal* CNTRL	AAA	P	FDR
PTPN6	5777	protein tyrosine phosphatase, non-receptor type 6	6.46	9.37	7.42e-09	3.29e-07
RAMP1	10267	receptor (calcitonin) activity modifying protein 1	10.85	7.68	1.63e-21	3.77e-18
RGS5	8490	regulator of G-protein signalling 5	11.84	8.40	1.23e-22	7.10e-19
RUNX3	864	runt-related transcription factor 3	6.12	9.71	2.75e-09	1.44e-07
SCRG1	11341	scrapie responsive protein 1	10.44	7.67	1.44e-15	4.36e-13
SEMA4A	64218	sema domain, immunoglobulin domain (Ig), transmembrane domain (TM) and short cytoplasmic domain, (semaphorin) 4A	6.24	8.90	1.43e-09	8.43e-08
SHRM	57619	shroom	8.56	5.80	1.15e-15	3.90e-13
SLC25A4	291	solute carrier family 25 (mitochondrial carrier; adenine nucleotide translocator), member 4	10.33	7.93	2.44e-14	5.31e-12
SPRY1	10252	sprouty homolog 1, antagonist of FGF signaling (Drosophila)	11.13	8.65	4.45e-16	1.80e-13
SYK	6850	spleen tyrosine kinase	7.37	10.01	2.24e-08	8.68e-07
SYNC1	81493	syncoilin, intermediate filament 1	8.19	5.83	9.83e-16	3.44e-13
TMC6	11322	transmembrane channel-like 6	5.94	8.55	3.08e-09	1.56e-07
TMEM47	83604	transmembrane protein 47	11.89	9.07	6.93e-19	7.27e-16
TNFRSF18	8784	tumor necrosis factor receptor superfamily, member 18	5.29	8.01	8.84e-10	5.70e-08
TRIB1	10221	tribbles homolog 1 (Drosophila)	7.95	10.59	1.36e-09	8.11e-08
TTLL7	79739	tubulin tyrosine ligase-like family, member 7	7.08	4.49	1.43e-16	6.89e-14
UCP2	7351	uncoupling protein 2 (mitochondrial, proton carrier)	7.78	10.46	1.81e-09	1.02e-07
UNC13D	201294	unc-13 homolog D (C. elegans)	5.72	8.11	1.16e-08	4.83e-07
UNQ5783	388325	DTFT5783	5.34	7.64	1.18e-08	4.90e-07
VAV1	7409	vav 1 oncogene	5.54	8.09	8.86e-09	3.86e-07
WAS	7454	Wiskott-Aldrich syndrome (eczema-thrombocytopenia)	7.72	10.02	1.80e-08	7.15e-07
ZAK	51776	sterile alpha motif and leucine zipper containing kinase AZK	10.20	8.21	1.80e-14	4.16e-12
ZBTB16	7704	zinc finger and BTB domain containing 16	10.13	7.42	1.48e-15	4.38e-13
ZD52F10	93099	dermokine	7.76	5.18	6.82e-15	1.71e-12
ZNF659	79750	zinc finger protein 659	9.16	6.47	6.24e-16	2.32e-13

* Signal indicates Log_2 transformed intensity value.
