# Supplementary material for: Whole genome expression profiling reveals a significant role for immune function in human abdominal aortic aneurysms
Source: BMC Genomics. 2007 Jul 16;8:237. doi: 10.1186/1471-2164-8-237 (PMC1934369; doi:10.1186/1471-2164-8-237)
Supplement: Additional file 4 — Expression values and significance of differential expression for individual genes included in the NK cytotoxicity pathway in Figure 4. Gene symbols, Entrez Gene IDs, gene names, signals, and significance of differential expression provided in a tabular format for NK cytotoxicity pathway. Gene symbols and Entrez Gene IDs contain links to the NCBI site. [file 1471-2164-8-237-S4.pdf]

Supplemental Table III: Expression values and significance of individual genes for NK cytotoxicity pathway in Figure 4.

KEGG Symbol	Gene Symbol	Entrez Gene ID	Gene Name	Control Signal	AAA Signal	P-value	Significant FDR
Bid	*BID*	637	BH3 interacting domain death agonist	6.61	8.17	1.31e-03	7.82e-03
CASP3	*CASP3*	836	caspase 3, apoptosis-related cysteine peptidase	7.61	8.43	3.03e-03	1.53e-02
2B4	*CD244*	51744	CD244 molecule, natural killer cell receptor 2B4	5.09	6.33	1.39e-04	1.28e-03
CD3z	*CD247*	919	CD247 molecule	6.75	9.09	3.86e-03	1.85e-02
CD48	*CD48*	962	CD48 molecule	7.18	9.95	9.39e-05	9.21e-04
CaN	*CHP*	11261	calcium binding protein P22	8.52	8.65	6.77e-01	
GM-CSF	*CSF2*	1437	colony stimulating factor 2 (granulocyte-macrophage)	3.25	3.13	8.66e-01	
FAS	*FAS*	355	Fas (TNF receptor superfamily, member 6)	6.12	6.15	8.97e-01	
FASL	*FASLG*	356	Fas ligand (TNF superfamily, member 6)	3.85	4.00	7.57e-01	
FceR1g	*FCER1G*	2207	Fc fragment of IgE, high affinity I, receptor for; gamma polypeptide	9.76	11.25	2.41e-04	1.99e-03
FcgRIII	*FCGR3A*	2214	Fc fragment of IgG, low affinity IIIa, receptor (CD16a)	10.56	12.31	4.68e-03	
FcgRIII	*FCGR3B*	2215	Fc fragment of IgG, low affinity IIIb, receptor (CD16b)	7.02	8.86	3.75e-03	1.81e-02
Fyn	*FYN*	2534	FYN oncogene related to SRC, FGR, YES	4.37	5.66	2.30e-02	
Grb2	*GRB2*	2885	growth factor receptor-bound protein 2	8.77	10.32	2.82e-06	5.02e-05
Granzyme	*GZMB*	3002	granzyme B (granzyme 2, cytotoxic T-lymphocyte-associated serine esterase 1)	5.32	7.75	2.19e-03	1.18e-02
DAP-10	*HCST*	10870	hematopoietic cell signal transducer	7.36	9.56	4.53e-04	3.33e-03
HLA-A3	*HLA-A*	3105	major histocompatibility complex, class I, A	8.97	10.33	2.16e-01	
HLA-B	*HLA-B*	3106	major histocompatibility complex, class I, B	11.61	12.94	2.53e-05	3.11e-04
HLA-B46	*HLA-B*	3106	major histocompatibility complex, class I, B	11.61	12.94	2.53e-05	3.11e-04
HLA-C	*HLA-C*	3107	major histocompatibility complex, class I, C	11.54	12.38	2.94e-03	1.49e-02
HLA-E	*HLA-E*	3133	major histocompatibility complex, class I, E	10.40	11.35	4.69e-03	2.14e-02
HLA-G1	*HLA-G*	3135	HLA-G histocompatibility antigen, class I, G	5.44	6.38	1.54e-03	8.90e-03
Ras	*HRAS*	3265	v-Ha-ras Harvey rat sarcoma viral oncogene homolog	7.13	6.70	1.95e-01	
ICAM 1/2	*ICAM1*	3383	intercellular adhesion molecule 1 (CD54), human rhinovirus receptor	5.45	6.58	9.60e-04	6.14e-03
ICAM 1/2	*ICAM2*	3384	intercellular adhesion molecule 2	9.16	9.70	2.20e-01	
IFNs	*IFNA1**	3439	interferon, alpha 1	3.25	3.21	9.56e-01	
IFNs	*IFNA10**	3446	interferon, alpha 10	4.63	4.51	7.58e-01	
IFNs	*IFNA13**	3447	interferon, alpha 13	3.69	3.53	7.88e-01	
IFNs	*IFNA14**	3448	interferon, alpha 14	2.43	2.91	6.72e-01	
IFNs	*IFNA16**	3449	interferon, alpha 16	3.64	3.99	5.16e-01	
IFNs	*IFNA17**	3451	interferon, alpha 17	3.43	3.46	9.67e-01	
IFNs	*IFNA2**	3440	interferon, alpha 2	3.29	4.04	1.88e-01	
IFNs	*IFNA21**	3452	interferon, alpha 21	3.01	3.31	6.81e-01	
IFNs	*IFNA4**	3441	interferon, alpha 4	3.24	3.29	9.45e-01	
IFNs	*IFNA5**	3442	interferon, alpha 5	4.32	3.91	3.41e-01	
IFNs	*IFNA6**	3443	interferon, alpha 6	3.84	3.99	7.62e-01	

KEGG Symbol	Gene Symbol	Entrez Gene ID	Gene Name	Control Signal	AAA Signal	P-value	Significant FDR
IFNs	*IFNA7**	3444	interferon, alpha 7	4.34	4.45	7.84e-01	
IFNs	*IFNA8**	3445	interferon, alpha 8	4.33	4.57	5.22e-01	
IFNsR	*IFNAR1*	3454	interferon (alpha, beta and omega) receptor 1	6.10	6.22	7.34e-01	
IFNsR	*IFNAR2*	3455	interferon (alpha, beta and omega) receptor 2	4.07	4.12	9.15e-01	
IFNs	*IFNB1*	3456	interferon, beta 1, fibroblast	3.75	3.93	7.29e-01	
IFN-g	*IFNG*	3458	interferon, gamma	6.06	6.11	8.86e-01	
IFNs	*IFNG*	3458	interferon, gamma	6.06	6.11	8.86e-01	
IFNgR	*IFNGR1*	3459	interferon gamma receptor 1	9.97	10.72	5.88e-03	2.53e-02
IFNsR	*IFNGR1*	3459	interferon gamma receptor 1	9.97	10.72	5.88e-03	2.53e-02
IFNgR	*IFNGR2*	3460	interferon gamma receptor 2 (interferon gamma transducer 1)	10.52	11.40	5.08e-03	2.27e-02
IFNsR	*IFNGR2*	3460	interferon gamma receptor 2 (interferon gamma transducer 1)	10.52	11.40	5.08e-03	2.27e-02
ITGAL	*ITGAL*	3683	integrin, alpha L (antigen CD11A (p180)	5.31	7.61	1.20e-08	4.96e-07
ITGB2	*ITGB2*	3689	integrin, beta 2 (complement component 3 receptor 3 and 4 subunit)	8.30	10.34	3.06e-07	7.67e-06
KIR2DL	*KIR2DL1**	3802	killer cell immunoglobulin-like receptor, two domains, long cytoplasmic tail, 1	3.53	3.83	5.87e-01	
KIR2DL	*KIR2DL2**	3803	killer cell immunoglobulin-like receptor, two domains, long cytoplasmic tail, 2	4.61	4.74	6.93e-01	
KIR2DL	*KIR2DL3**	3804	killer cell immunoglobulin-like receptor, two domains, long cytoplasmic tail, 3	4.45	4.55	7.77e-01	
KIR2DL	*KIR2DL4**	3805	killer cell immunoglobulin-like receptor, two domains, long cytoplasmic tail, 4	4.18	4.01	6.92e-01	
KIR2DL	*KIR2DL5A**	57292	killer cell immunoglobulin-like receptor, two domains, long cytoplasmic tail, 5A	4.52	5.10	1.76e-01	
KIR2DS	*KIR2DS1*	3806	killer cell immunoglobulin-like receptor, two domains, short cytoplasmic tail, 1	4.19	3.85	4.70e-01	
KIR2DS	*KIR2DS2*	3807	killer cell immunoglobulin-like receptor, two domains, short cytoplasmic tail, 2	4.71	4.98	4.05e-01	
KIR3DL1	*KIR3DL1*	3811	killer cell immunoglobulin-like receptor, three domains, long cytoplasmic tail, 1	4.69	5.47	3.31e-02	
KIR3DL2	*KIR3DL2*	3812	killer cell immunoglobulin-like receptor, three domains, long cytoplasmic tail, 2	4.47	4.30	6.68e-01	
NKG2 A/B	*KLRC1*	3821	killer cell lectin-like receptor subfamily C, member 1	4.01	3.55	3.83e-01	
NKG2C/E	*KLRC2*	3822	killer cell lectin-like receptor subfamily C, member 2	3.92	4.13	6.66e-01	
NKG2C/E	*KLRC3*	3823	killer cell lectin-like receptor subfamily C, member 3	4.34	4.20	7.46e-01	
CD94	*KLRD1*	3824	killer cell lectin-like receptor subfamily D, member 1	5.80	6.23	2.32e-01	
NKG2D	*KLRK1*	22914	killer cell lectin-like receptor subfamily K, member 1	4.55	6.19	3.55e-02	
Ras	*KRAS*	3845	v-Ki-ras2 Kirsten rat sarcoma viral oncogene homolog	9.06	9.41	1.72e-01	
LAT	*LAT*	27040	linker for activation of T cells	6.63	8.54	1.14e-04	1.09e-03
Lck	*LCK*	3932	lymphocyte-specific protein tyrosine kinase	6.20	9.09	1.66e-02	
SLP-76	*LCP2*	3937	lymphocyte cytosolic protein 2	7.27	9.63	6.41e-09	2.90e-07
Mek 1/2	*MAP2K1*	5604	mitogen-activated protein kinase kinase 1	9.62	9.70	7.33e-01	
Mek 1/2	*MAP2K2*	5605	mitogen-activated protein kinase kinase 2	10.33	11.13	3.56e-03	1.73e-02
ERK 1/2	*MAPK1*	5594	mitogen-activated protein kinase 1	6.53	7.64	3.19e-04	2.50e-03
ERK 1/2	*MAPK3*	5595	mitogen-activated protein kinase 3	10.20	10.02	4.31e-01	
MICA	*MICA*	4276	MHC class I polypeptide-related sequence A	9.45	8.28	3.89e-07	9.38e-06
MICB	*MICB*	4277	MHC class I polypeptide-related sequence B	6.40	7.55	1.62e-04	1.45e-03
NKp46	*NCR1*	9437	natural cytotoxicity triggering receptor 1	3.72	4.03	5.19e-01	

KEGG Symbol	Gene Symbol	Entrez Gene ID	Gene Name	Control Signal	AAA Signal	P-value	Significant FDR
NKp44	*NCR2*	9436	natural cytotoxicity triggering receptor 2	3.72	4.08	5.23e-01	
NKp30	*NCR3*	259197	natural cytotoxicity triggering receptor 3	4.14	6.36	3.90e-02	
NFAT	*NFAT5*	10725	nuclear factor of activated T-cells 5, tonicity-responsive	7.24	7.40	6.03e-01	
NFAT	*NFATC1*	4772	nuclear factor of activated T-cells, cytoplasmic, calcineurin-dependent 1	7.39	8.99	3.30e-04	2.57e-03
NFAT	*NFATC2*	4773	nuclear factor of activated T-cells, cytoplasmic, calcineurin-dependent 2	3.51	3.79	6.33e-01	
NFAT	*NFATC3*	4775	nuclear factor of activated T-cells, cytoplasmic, calcineurin-dependent 3	4.96	5.76	1.03e-02	3.89e-02
NFAT	*NFATC4*	4776	nuclear factor of activated T-cells, cytoplasmic, calcineurin-dependent 4	5.91	5.19	2.46e-02	
Ras	*NRAS*	4893	neuroblastoma RAS viral (v-ras) oncogene homolog	6.96	7.65	4.35e-02	
PAK1	*PAK1*	5058	p21/Cdc42/Rac1-activated kinase 1 (STE20 homolog, yeast)	7.65	8.61	4.16e-03	1.96e-02
PI3K	*PIK3CA*	5290	phosphoinositide-3-kinase, catalytic, alpha polypeptide	7.56	7.06	4.94e-02	
PI3K	*PIK3CB*	5291	phosphoinositide-3-kinase, catalytic, beta polypeptide	6.03	6.89	5.93e-02	
PI3K	*PIK3CD*	5293	phosphoinositide-3-kinase, catalytic, delta polypeptide	7.83	10.00	2.14e-08	8.41e-07
PI3K	*PIK3CG*	5294	phosphoinositide-3-kinase, catalytic, gamma polypeptide	6.19	8.47	5.03e-05	5.53e-04
PI3K	*PIK3R1*	5295	phosphoinositide-3-kinase, regulatory subunit 1 (p85 alpha)	10.23	9.38	1.13e-03	6.99e-03
PI3K	*PIK3R2*	5296	phosphoinositide-3-kinase, regulatory subunit 2 (p85 beta)	5.56	5.58	9.63e-01	
PI3K	*PIK3R3*	8503	phosphoinositide-3-kinase, regulatory subunit 3 (p55, gamma)	5.39	5.06	4.29e-01	
PI3K	*PIK3R5*	23533	phosphoinositide-3-kinase, regulatory subunit 5, p101	4.32	5.07	1.08e-01	
PLCg	*PLCG1*	5335	phospholipase C, gamma 1	8.98	8.18	7.96e-03	3.21e-02
PLCg	*PLCG2*	5336	phospholipase C, gamma 2 (phosphatidylinositol-specific)	6.79	8.51	2.53e-04	2.07e-03
CaN	*PPP3CA*	5530	protein phosphatase 3 (formerly 2B), catalytic subunit, alpha isoform	5.90	6.14	4.31e-01	
CaN	*PPP3CB*	5532	protein phosphatase 3 (formerly 2B), catalytic subunit, beta isoform	10.14	9.59	1.73e-02	
CaN	*PPP3CC*	5533	protein phosphatase 3 (formerly 2B), catalytic subunit, gamma isoform	8.71	8.27	9.56e-02	
CaN	*PPP3R1*	5534	protein phosphatase 3 (formerly 2B), regulatory subunit B, 19kDa, alpha isoform	9.51	9.68	4.90e-01	
CaN	*PPP3R2*	5535	protein phosphatase 3 (formerly 2B), regulatory subunit B, 19kDa, beta isoform	4.05	4.05	1.00e-00	
Perforin	*PRF1*	5551	perforin 1 (pore forming protein)	4.57	6.01	2.39e-02	
PKC	*PRKCA*	5578	protein kinase C, alpha	5.31	5.09	5.42e-01	
PKC	*PRKCB1*	5579	protein kinase C, beta 1	7.46	10.20	1.48e-05	2.01e-04
PKC	*PRKCG*	5582	protein kinase C, gamma	4.57	4.57	9.94e-01	
Pyk-2	*PTK2B*	2185	PTK2B protein tyrosine kinase 2 beta	6.96	8.48	5.32e-06	8.48e-05
SHP-2	*PTPN11*	5781	protein tyrosine phosphatase, non-receptor type 11 (Noonan syndrome 1)	8.73	7.96	6.95e-04	4.75e-03
SHP-1	*PTPN6*	5777	protein tyrosine phosphatase, non-receptor type 6	6.46	9.37	7.42e-09	3.29e-07
Rac	*RAC1*	5879	ras-related C3 botulinum toxin substrate 1	7.28	5.96	4.78e-05	5.28e-04
Rac	*RAC2*	5880	ras-related C3 botulinum toxin substrate 2	7.21	9.69	4.96e-07	1.16e-05
Rac	*RAC3*	5881	ras-related C3 botulinum toxin substrate 3	6.05	6.06	9.77e-01	
Raf-1	*RAF1*	5894	v-raf-1 murine leukemia viral oncogene homolog 1	8.32	8.34	9.38e-01	
EAT-2	*SH2D1B*	117157	SH2 domain containing 1B	4.38	5.39	2.92e-02	
3BP2	*SH3BP2*	6452	SH3-domain binding protein 2	4.94	6.54	1.01e-05	1.44e-04
Shc	*SHC1*	6464	SHC (Src homology 2 domain containing) transforming protein 1	10.30	10.15	6.03e-01	

KEGG Symbol	Gene Symbol	Entrez Gene ID	Gene Name	Control Signal	AAA Signal	P-value	Significant FDR
Shc	*SHC2*	25759	SHC (Src homology 2 domain containing) transforming protein 2	3.17	3.13	9.62e-01	
Shc	*SHC3*	53358	SHC (Src homology 2 domain containing) transforming protein 3	4.38	4.69	5.26e-01	
Shc	*SHC4*	399694	SHC (Src homology 2 domain containing) family, member 4	7.54	5.72	1.02e-07	3.10e-06
Sos	*SOS1*	6654	son of sevenless homolog 1 (Drosophila)	6.09	6.03	8.55e-01	
Sos	*SOS2*	6655	son of sevenless homolog 2 (Drosophila)	4.45	4.50	9.22e-01	
Syk	*SYK*	6850	spleen tyrosine kinase	7.37	10.01	2.24e-08	8.68e-07
TNF-a	*TNF*	7124	tumor necrosis factor (TNF superfamily, member 2)	4.20	6.46	7.72e-05	7.90e-04
TRAILR	*TNFRSF10A*	8797	tumor necrosis factor receptor superfamily, member 10a	5.73	6.68	1.24e-01	
TRAILR	*TNFRSF10B*	8795	tumor necrosis factor receptor superfamily, member 10b	8.97	9.55	3.15e-02	
TRAILR	*TNFRSF10C*	8794	tumor necrosis factor receptor superfamily, member 10c	4.88	4.76	7.05e-01	
TRAILR	*TNFRSF10D*	8793	tumor necrosis factor receptor superfamily, member 10d	4.98	5.23	3.91e-01	
TRAIL	*TNFSF10*	8743	tumor necrosis factor (ligand) superfamily, member 10	7.90	8.74	7.46e-02	
DAP-12	*TYROBP*	7305	TYRO protein tyrosine kinase binding protein	9.68	12.28	7.67e-05	7.87e-04
ULBP1-3	*ULBP1*	80329	UL16 binding protein 1	4.04	4.31	5.17e-01	
ULBP1-3	*ULBP2*	80328	UL16 binding protein 2	3.53	4.28	1.82e-01	
ULBP1-3	*ULBP3*	79465	UL16 binding protein 3	3.68	3.80	8.38e-01	
Vav	*VAV1*	7409	vav 1 oncogene	5.54	8.09	8.86e-09	3.86e-07
Vav	*VAV2*	7410	vav 2 oncogene	4.44	4.44	9.97e-01	
Vav	*VAV3*	10451	vav 3 oncogene	5.60	7.53	1.57e-07	4.45e-06
ZAP70	*ZAP70*	7535	zeta-chain (TCR) associated protein kinase 70kDa	4.97	6.63	2.67e-02	

*Excluded from Figure 4 to save space
