# Supplementary material for: Whole genome expression profiling reveals a significant role for immune function in human abdominal aortic aneurysms
Source: BMC Genomics. 2007 Jul 16;8:237. doi: 10.1186/1471-2164-8-237 (PMC1934369; doi:10.1186/1471-2164-8-237)
Supplement: Additional file 5 — Expression values and significance of differential expression for individual genes included in the LTEM pathway in Figure 5. Gene symbols, Entrez Gene IDs, gene names, signals, and significance of differential expression provided in a tabular format for LTEM pathway. Gene symbols and Entrez Gene IDs contain links to the NCBI site. [file 1471-2164-8-237-S5.pdf]

Supplemental Table IV: Expression values and significance of individual genes for LTEM pathway in Figure 5.

KEGG Symbol	Gene Symbol	Entrez Gene ID	Gene Name	Control Signal	AAA Signal	P-value	Significant FDR
Actin	*ACTB*	60	actin, beta	14.75	14.46	2.04e-01	
Actin	*ACTC*	70	actin, alpha, cardiac muscle	11.04	7.94	5.57e-11	5.40e-09
Actin	*ACTG1*	71	actin, gamma 1	12.88	12.71	4.59e-01	
a-actinin	*ACTN1*	87	actinin, alpha 1	12.18	10.96	8.30e-07	1.80e-05
a-actinin	*ACTN2*	88	actinin, alpha 2	4.24	3.50	1.24e-01	
a-actinin	*ACTN3*	89	actinin, alpha 3	4.24	4.44	6.03e-01	
a-actinin	*ACTN4*	81	actinin, alpha 4	10.95	9.94	3.99e-04	2.98e-03
RhoGAP	*ARHGAP5*	394	Rho GTPase activating protein 5	4.16	4.16	9.98e-01	
p130Cas	*BCAR1*	9564	breast cancer anti-estrogen resistance 1	7.23	6.39	1.90e-01	
CD99	*CD99*	4267	CD99 antigen	11.50	11.21	2.08e-01	
Cdc42	*CDC42*	998	cell division cycle 42 (GTP binding protein, 25kDa)	8.99	9.22	3.43e-01	
CDH5	*CDH5*	1003	cadherin 5, type 2, VE-cadherin (vascular epithelium)	7.84	7.44	2.24e-01	
CAMs	*CLDN11**	5010	claudin 11 (oligodendrocyte transmembrane protein)	8.76	8.03	1.25e-01	
CAMs	*CLDN14**	23562	claudin 14	8.67	8.35	1.55e-01	
CAMs	*CLDN15**	24146	claudin 15	7.62	7.32	4.68e-01	
CAMs	*CLDN16**	10686	claudin 16	4.06	4.28	5.83e-01	
CAMs	*CLDN17**	26285	claudin 17	4.47	4.64	6.24e-01	
CAMs	*CLDN18**	51208	claudin 18	5.32	4.82	2.41e-01	
CAMs	*CLDN19**	149461	claudin 19	4.63	4.60	9.35e-01	
CAMs	*CLDN22**	53842	claudin 22	4.42	4.57	6.62e-01	
CAMs	*CLDN23**	137075	claudin 23	7.29	7.84	1.59e-01	
CAMs	*CLDN3**	1365	claudin 3	4.73	4.86	7.22e-01	
CAMs	*CLDN4**	1364	claudin 4	4.81	4.84	9.04e-01	
CAMs	*CLDN7**	1366	claudin 7	5.17	5.37	6.52e-01	
a-Catenin	*CTNNA1*	1495	catenin (cadherin-associated protein), alpha 1, 102kDa	9.37	8.46	1.86e-02	
a-Catenin	*CTNNA2*	1496	catenin (cadherin-associated protein), alpha 2	4.32	4.54	5.46e-01	
a-Catenin	*CTNNA3*	29119	catenin (cadherin-associated protein), alpha 3	4.51	4.07	2.72e-01	
b-Catenin	*CTNNB1*	1499	catenin (cadherin-associated protein), beta 1, 88kDa	7.49	7.75	3.41e-01	
p120ctn	*CTNND1*	1500	catenin (cadherin-associated protein), delta 1	8.52	7.96	2.22e-02	
SDF-1	*CXCL12*	6387	chemokine (C-X-C motif) ligand 12 (stromal cell-derived factor 1)	11.67	11.62	8.87e-01	
SDF-1	*CXCL13*	10563	chemokine (C-X-C motif) ligand 13 (B-cell chemoattractant)	4.47	8.36	4.33e-02	
SDF-1	*CXCL14*	9547	chemokine (C-X-C motif) ligand 14	10.72	11.23	5.12e-01	
SDF-1	*CXCL16*	58191	chemokine (C-X-C motif) ligand 16	9.65	11.42	8.81e-04	5.74e-03
CXCR4	*CXCR4*	7852	chemokine (C-X-C motif) receptor 4	10.63	12.32	2.69e-06	4.84e-05
p22phax	*CYBA*	1535	cytochrome b-245, alpha polypeptide	9.39	11.78	1.77e-08	7.07e-07
Nox	*CYBB*	1536	cytochrome b-245, beta polypeptide (chronic granulomatous disease)	7.51	9.52	1.53e-07	4.36e-06

KEGG Symbol	Gene Symbol	Entrez Gene ID	Gene Name	Control Signal	AAA Signal	P-value	Significant FDR
JAM1	*F11R*	50848	F11 receptor	6.58	8.12	1.21e-05	1.71e-04
Gi	*GNAI1*	2770	guanine nucleotide binding protein (G protein), alpha inhibiting activity polypeptide 1	6.49	5.17	5.95e-06	9.29e-05
Gi	*GNAI2*	2771	guanine nucleotide binding protein (G protein), alpha inhibiting activity polypeptide 2	11.59	11.92	2.23e-01	
Gi	*GNAI3*	2773	guanine nucleotide binding protein (G protein), alpha inhibiting activity polypeptide 3	8.41	8.55	5.54e-01	
RhoGAP	*GRLF1*	2909	glucocorticoid receptor DNA binding factor 1	7.56	7.60	8.67e-01	
ICAM1	*ICAM1*	3383	intercellular adhesion molecule 1 (CD54), human rhinovirus receptor	5.45	6.58	9.60e-04	6.14e-03
SDF-1	*IL8*	3576	interleukin 8	8.72	12.23	4.35e-03	2.03e-02
ITGA4	*ITGA4*	3676	integrin, alpha 4 (antigen CD49D, alpha 4 subunit of VLA-4 receptor)	3.48	5.54	6.43e-04	4.45e-03
ITGAL	*ITGAL*	3683	integrin, alpha L (antigen CD11A (p180))	5.31	7.61	1.20e-08	4.96e-07
ITGAM	*ITGAM*	3684	integrin, alpha M (complement component 3 receptor 3 subunit)	7.42	9.40	3.75e-03	1.81e-02
ITGB1	*ITGB1*	3688	integrin, beta 1 (fibronectin receptor, beta polypeptide, antigen CD29 includes MDF2, MSK12)	10.89	9.50	1.60e-06	3.12e-05
ITGB2	*ITGB2*	3689	integrin, beta 2 (antigen CD18 (p95))	8.30	10.34	3.06e-07	7.67e-06
TEC	*ITK*	3702	IL2-inducible T-cell kinase	5.11	8.33	2.80e-02	
JAM2	*JAM2*	58494	junctional adhesion molecule 2	7.49	6.56	5.68e-03	2.47e-02
JAM3	*JAM3*	83700	junctional adhesion molecule 3	11.38	9.28	1.20e-13	2.13e-11
p38	*MAPK11*	5600	mitogen-activated protein kinase 11	5.14	5.18	9.47e-01	
p38	*MAPK12*	6300	mitogen-activated protein kinase 12	3.99	4.03	9.54e-01	
p38	*MAPK13*	5603	mitogen-activated protein kinase 13	5.23	8.26	1.46e-06	2.88e-05
p38	*MAPK14*	1432	mitogen-activated protein kinase 14	5.52	5.19	2.39e-01	
AF-6	*MLLT4*	4301	myeloid/lymphoid or mixed-lineage leukemia translocated to, 4	4.65	4.41	5.77e-01	
MMPs	*MMP2*	4313	matrix metallopeptidase 2	10.65	9.85	1.44e-01	
MMPs	*MMP9*	4318	matrix metallopeptidase 9	7.62	12.31	1.31e-02	4.68e-02
ERM	*MSN*	4478	moesin	10.84	10.80	8.66e-01	
MLC	*MYL6*	4637	myosin, light polypeptide 6, alkali, smooth muscle and non-muscle	12.37	11.79	2.08e-02	
p47phax	*NCF1*	4687	neutrophil cytosolic factor 1	8.32	11.46	4.66e-06	7.53e-05
p67phax	*NCF2*	4688	neutrophil cytosolic factor 2	7.51	10.21	1.74e-03	9.77e-03
p40phax	*NCF4*	4689	neutrophil cytosolic factor 4, 40kDa	8.69	10.76	5.36e-08	1.77e-06
Nox	*NOX1*	27035	NADPH oxidase 1	5.16	5.44	4.90e-01	
Nox	*NOX3*	50508	NADPH oxidase 3	4.54	4.31	5.25e-01	
CAMs	*OCLN**	4950	occludin	4.50	4.50	9.93e-01	
PECAM1	*PECAM1*	5175	platelet/endothelial cell adhesion molecule (CD31 antigen)	11.17	11.59	1.21e-01	
SDF-1	*PF4*	5196	platelet factor 4 (chemokine (C-X-C motif) ligand 4)	5.15	8.38	5.26e-02	
PI3K	*PIK3CA*	5290	phosphoinositide-3-kinase, catalytic, alpha polypeptide	7.56	7.06	4.94e-02	
PI3K	*PIK3CB*	5291	phosphoinositide-3-kinase, catalytic, beta polypeptide	6.03	6.89	5.93e-02	
PI3K	*PIK3CD*	5293	phosphoinositide-3-kinase, catalytic, delta polypeptide	7.83	10.00	2.14e-08	8.41e-07
PI3K	*PIK3CG*	5294	phosphoinositide-3-kinase, catalytic, gamma polypeptide	6.19	8.47	5.03e-05	5.53e-04
PI3K	*PIK3R1*	5295	phosphoinositide-3-kinase, regulatory subunit 1 (p85 alpha)	10.23	9.38	1.13e-03	6.99e-03

KEGG Symbol	Gene Symbol	Entrez Gene ID	Gene Name	Control Signal	AAA Signal	P-value	Significant FDR
PI3K	*PIK3R2*	5296	phosphoinositide-3-kinase, regulatory subunit 2 (p85 beta)	5.56	5.58	9.63e-01	
PI3K	*PIK3R3*	8503	phosphoinositide-3-kinase, regulatory subunit 3 (p55, gamma)	5.39	5.06	4.29e-01	
PI3K	*PIK3R5*	23533	phosphoinositide-3-kinase, regulatory subunit 5, p101	4.32	5.07	1.08e-01	
PLCg	*PLCG1*	5335	phospholipase C, gamma 1	8.98	8.18	7.96e-03	3.21e-02
PLCg	*PLCG2*	5336	phospholipase C, gamma 2 (phosphatidylinositol-specific)	6.79	8.51	2.53e-04	2.07e-03
SDF-1	*PPBP*	5473	pro-platelet basic protein (chemokine (C-X-C motif) ligand 7)	5.89	9.70	7.01e-02	
PKC	*PRKCA*	5578	protein kinase C, alpha	5.31	5.09	5.42e-01	
PKC	*PRKCB1*	5579	protein kinase C, beta 1	7.46	10.20	1.48e-05	2.01e-04
PKC	*PRKCG*	5582	protein kinase C, gamma	4.57	4.57	9.94e-01	
FAK	*PTK2*	5747	PTK2 protein tyrosine kinase 2	9.95	8.47	2.08e-08	8.20e-07
Pyk2	*PTK2B*	2185	PTK2B protein tyrosine kinase 2 beta	6.96	8.48	5.32e-06	8.48e-05
SHP-2	*PTPN11*	5781	protein tyrosine phosphatase, non-receptor type 11 (Noonan syndrome 1)	8.73	7.96	6.95e-04	4.75e-03
Paxillin	*PXN*	5829	paxillin	6.77	7.30	1.62e-01	
Rac1	*RAC1*	5879	ras-related C3 botulinum toxin substrate 1 (rho family, small GTP binding protein Rac1)	7.28	5.96	4.78e-05	5.28e-04
Rac2	*RAC2*	5880	ras-related C3 botulinum toxin substrate 2	7.21	9.69	4.96e-07	1.16e-05
Rap1	*RAP1A*	5906	RAP1A, member of RAS oncogene family	9.95	9.92	9.13e-01	
Rap1	*RAP1B*	5908	RAP1B, member of RAS oncogene family	10.44	10.56	6.77e-01	
EPAC	*RAPGEF3*	10411	Rap guanine nucleotide exchange factor (GEF) 3	7.67	6.51	1.91e-02	
EPAC	*RAPGEF4*	11069	Rap guanine nucleotide exchange factor (GEF) 4	4.74	4.62	8.29e-01	
RAPL	*RASSF5*	83593	Ras association (RalGDS/AF-6) domain family 5	4.83	5.64	1.70e-02	
RhoA	*RHOA*	387	ras homolog gene family, member A	11.75	11.81	8.18e-01	
RhoH	*RHOH*	399	ras homolog gene family, member H	5.61	8.30	5.26e-03	2.32e-02
ROCK	*ROCK1*	6093	Rho-associated, coiled-coil containing protein kinase 1	7.38	5.19	1.70e-03	9.61e-03
ROCK	*ROCK2*	9475	Rho-associated, coiled-coil containing protein kinase 2	9.23	8.02	1.89e-07	5.17e-06
SPA-1	*SIPA1*	6494	signal-induced proliferation-associated gene 1	6.67	8.03	1.87e-04	1.61e-03
Thy1	*THY1*	7070	Thy-1 cell surface antigen	8.30	9.59	2.06e-01	
TEC	*TXK*	7294	TXK tyrosine kinase	3.25	5.28	7.78e-02	
VASP	*VASP*	7408	vasodilator-stimulated phosphoprotein	11.96	12.70	2.11e-02	
Vav	*VAV1*	7409	vav 1 oncogene	5.54	8.09	8.86e-09	3.86e-07
Vav	*VAV2*	7410	vav 2 oncogene	4.44	4.44	9.97e-01	
Vav	*VAV3*	10451	vav 3 oncogene	5.60	7.53	1.57e-07	4.45e-06
VCAM1	*VCAM1*	7412	vascular cell adhesion molecule 1	10.64	10.76	8.04e-01	
Vinculin	*VCL*	7414	vinculin	11.07	9.23	2.19e-12	2.97e-10
ERM	*VIL2*	7430	villin 2 (ezrin)	8.94	10.66	1.30e-04	1.21e-03

* Excluded from Figure 5 to save space.
