# Supplementary material for: Whole genome expression profiling reveals a significant role for immune function in human abdominal aortic aneurysms
Source: BMC Genomics. 2007 Jul 16;8:237. doi: 10.1186/1471-2164-8-237 (PMC1934369; doi:10.1186/1471-2164-8-237)
Supplement: Additional file 6 — Signal intensity histograms. Unadjusted, i.e. raw, signals from microarray experiments were converted into histograms to visualize the lack of patterns of bias. [file 1471-2164-8-237-S6.pdf]

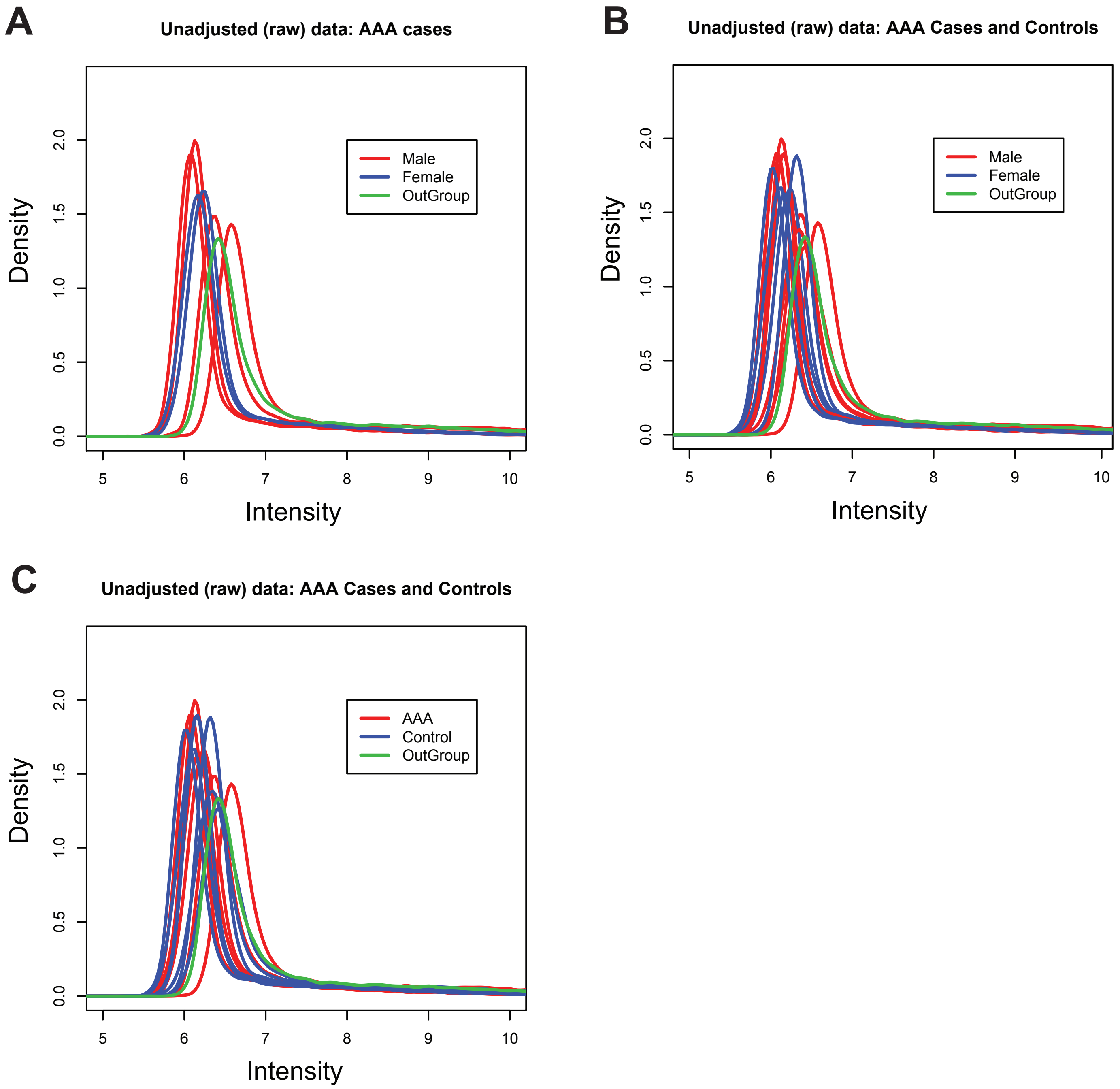

Supplemental Figure II Signal Intensity histograms. Unadjusted, i.e. raw, signals were converted into histograms using R (R: A Language and Environment for Statistical Computing; R version 2.4.1). The histograms are colored by sex (Panels **A** and **B**) and tissue type (Panel **C**; AAA vs. Control). There is no systematic bias in the observed variation, either by sex or tissue. Lack of systematic bias between AAA and control tissue suggest the difference in tissue source is not reflected in a difference in RNA signal and therefore RNA integrity at a global level.
