# Supplementary material for: Whole genome expression profiling reveals a significant role for immune function in human abdominal aortic aneurysms
Source: BMC Genomics. 2007 Jul 16;8:237. doi: 10.1186/1471-2164-8-237 (PMC1934369; doi:10.1186/1471-2164-8-237)
Supplement: Additional file 7 — Principal component analysis. Principal component analysis was carried out on the microarray data to show that there was no systematic bias in the samples. [file 1471-2164-8-237-S7.pdf]

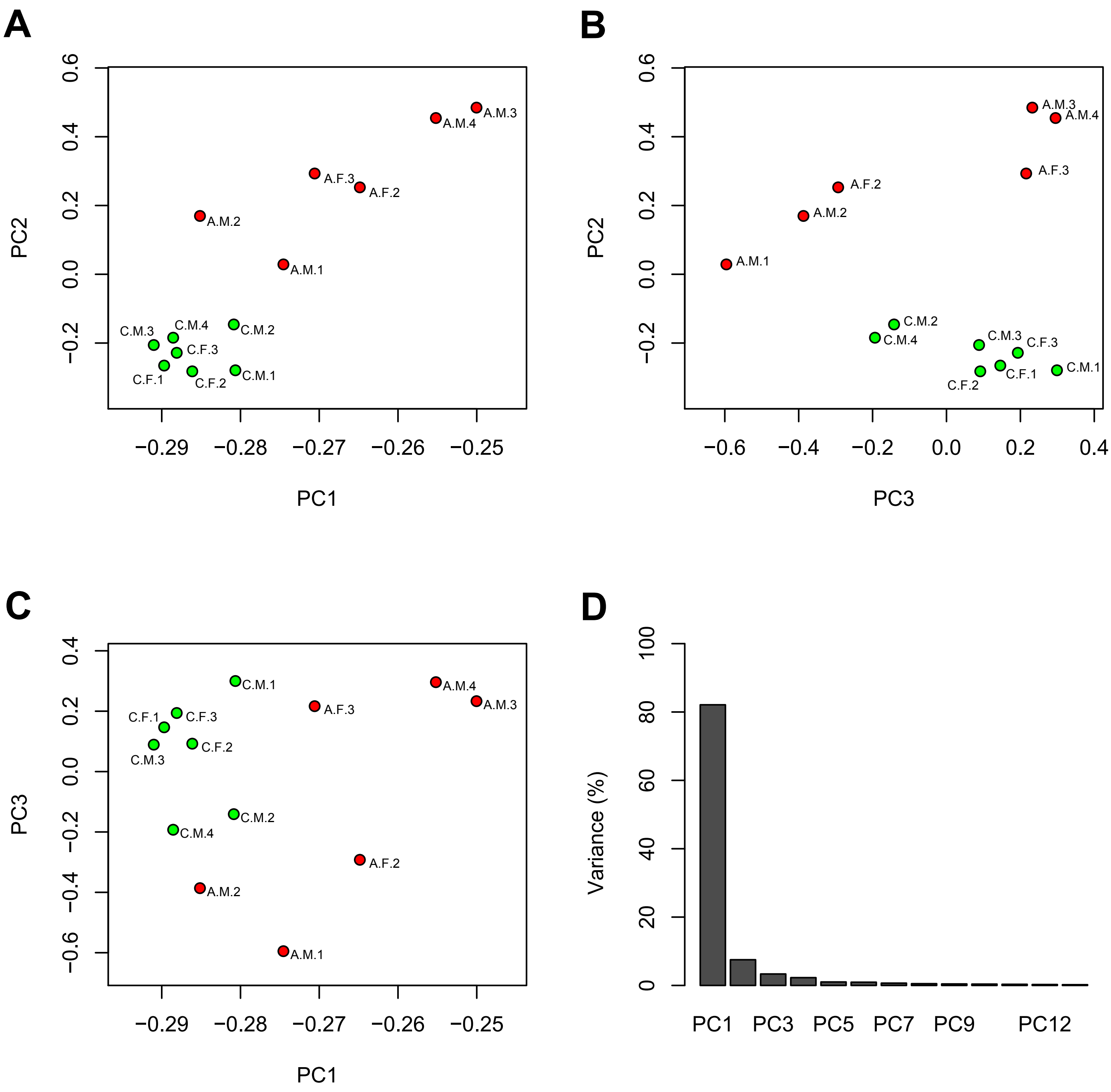

Supplemental Figure III Principal Component Analysis. Cubic-spline adjusted data were analyzed using principal components analysis (PCA) in R (function prcomp). **A-C**, The first three principal components (PCs) in pairwise plots. **D**, scree plot of the PCA analysis (illustrates the proportion of variance explained by each PC). The samples separated by disease status: completely in PC2, and nearly so in PC1. No systematic bias, other than disease status, could be detected for sex or age (not shown). See Table 1 for details on the sample characteristics.
