# Supplementary material for: Whole genome expression profiling reveals a significant role for immune function in human abdominal aortic aneurysms
Source: BMC Genomics. 2007 Jul 16;8:237. doi: 10.1186/1471-2164-8-237 (PMC1934369; doi:10.1186/1471-2164-8-237)
Supplement: Additional file 3 — Modified "leukocyte transendothelial migration" (hsa04670) pathway from KEGG. Protein symbols were replaced by gene symbols to reflect gene-centric data. See key for explanation of colors and symbols (see the additional file Table IV for expression values of the genes in both conditions as well as the FDR value for those that were significantly different). [file 1471-2164-8-237-S3.pdf]

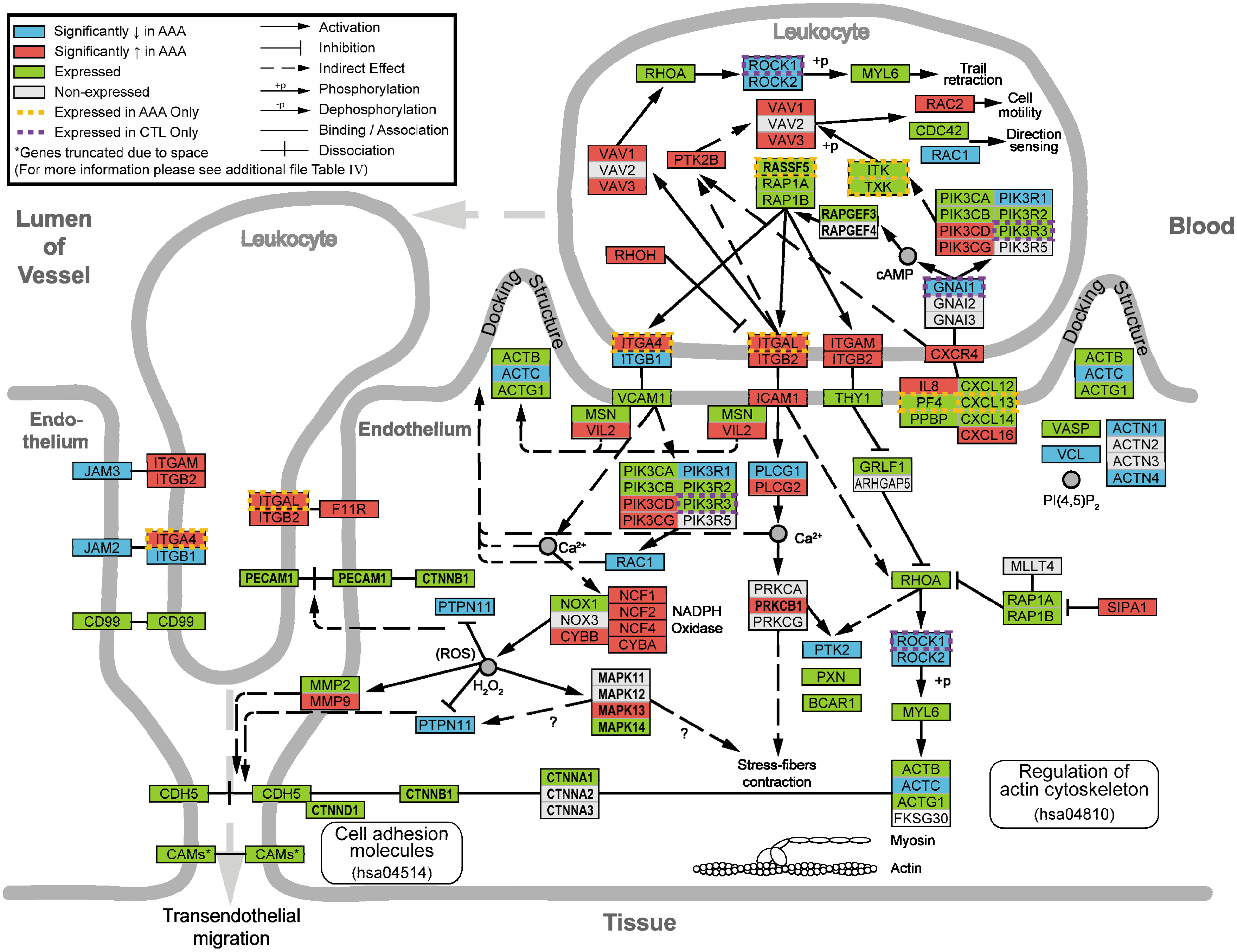

Figure I: Modified "leukocyte transendothelial migration" (hsa04670) pathway from KEGG.
